# Transcription factor 12‐mediated self‐feedback regulatory mechanism is required in *DUX4* fusion leukaemia

**DOI:** 10.1002/ctm2.1514

**Published:** 2023-12-19

**Authors:** Zhihui Li, Minghao Jiang, Junfei Wang, Zhiyi Zhuo, Shiyan Zhang, Yangxia Tan, Weiguo Hu, Hao Zhang, Guoyu Meng

**Affiliations:** ^1^ Shanghai Institute of Hematology State Key Laboratory of Medical Genomics National Research Center for Translational Medicine Rui‐Jin Hospital Shanghai Jiao Tong University School of Medicine and School of Life Sciences and Biotechnology, Shanghai Jiao Tong University Shanghai P. R. China; ^2^ Department of Geriatrics and Medical Center on Aging Ruijin Hospital, Shanghai Jiao Tong University School of Medicine Shanghai P. R. China; ^3^ Institute for Translational Brain Research State Key Laboratory of Medical Neurobiology MOE Frontiers Center for Brain Science Jinshan Hospital Fudan University Shanghai P. R. China; ^4^ State Key Laboratory of Pathogenesis Prevention and Treatment of High Incidence Diseases in Central Asia, First Affiliated Hospital of Xinjiang Medical University Xinjiang P. R. China

**Keywords:** B‐cell acute lymphoblastic leukaemia, IGH::DUX4, oncogenic driver, TCF12, transcriptional regulation

## Abstract

**Background:**

*IGH*::*DUX4* is frequently observed in 4% B‐cell acute lymphoblastic leukaemia patients. Regarding the IGH::DUX4‐driven transactivation and alternative splicing, which are the main reasons behind this acute leukaemia outbreak, it remains unclear how transcriptional cofactors contribute to this oncogenic process. Further investigation is required to elucidate their specific role in leukaemogenesis.

**Methods:**

In order to investigate the cofactors of IGH::DUX4, integrated mining of Chromatin immunoprecipitation (ChIP)‐sequencing and RNA‐sequencing of leukaemia cells and patient samples were conducted. Furthermore, to elucidate the synergistic interaction between transcription factor 12 (TCF12) and IGH::DUX4, knockdown and knockout experiment, mammalian two‐hybridisation assay, co‐immunoprecipitation and in situ proximity ligation assays were carried out. Additionally, to further investigate the direct interaction between TCF12 and IGH::DUX4, AI‐based structural simulations were utilised. Finally, to validate the synergistic role of TCF12 in promoting IGH::DUX4 leukaemia, cell proliferation, apoptosis and drug sensitivity experiments were performed.

**Results:**

In this study, we observed that the IGH::DUX4 target gene *TCF12* might be an important cofactor/helper for this oncogenic driver. The co‐expression of IGH::DUX4 and TCF12 resulted in enhanced DUX4‐driven transactivation. Supportively, knockdown and knockout of *TCF12* significantly reduced expression of IGH::DUX4‐driven target genes in leukaemia REH (a precursor B‐cell leukaemia cell line) and NALM‐6 cells (a precursor B‐cell leukaemia cell line). Consistently, in *TCF12* knockout cells, the expression of structure‐based TCF12 mutant, but not wild‐type TCF12, failed to restore the TCF12–IGH::DUX4 crosstalk and the synergistic transactivation. More importantly, the breakdown in TCF12–IGH::DUX4 cooperation impaired *IGH::DUX4*‐driven leukaemia cell survival, caused sensitivity to the chemotherapy.

**Conclusions:**

Altogether, these results helped to define a previously unrecognised TCF12‐mediated positive self‐feedback regulatory mechanism in *IGH::DUX4* leukaemia, which holds the potential to function as a pivotal drug target for the management of this particular form of leukaemia.

**Highlights:**

- Transcription factor 12 (TCF12) is a new novel cofactor in IGH::DUX4 transcriptional complexes/machinery.
- TCF12 mediates a positive self‐feedback regulatory mechanism in IGH::DUX4‐driven oncogenic transaction.
- IGH::DUX4–TCF12 structure/cooperation might represent a potent target/direction in future drug design against B‐cell acute lymphoblastic leukaemia.

## Graphical abstract

The significance of these results presented in this manuscript is three‐fold: 1) identifying a novel co‐factor in IGH::DUX4 transcriptional complexes/machinery; 2) highlighting a TCF12‐mediated self‐feedback regulatory mechanism in oncogenic transaction; 3) revealing the structural basis of IGH::DUX4‐TCF12 cooperation that might be a potent target/direction in future drug design against B‐ALL leukaemia.

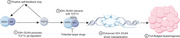

## 1 INTRODUCTION

Acute lymphoblastic leukaemia (ALL) represents a prevalent malignancy among paediatric patients, with peak incidence occurring between the ages of 3 and 5 years. The disease is characterised by genetic alterations that impede cellular differentiation and promote abnormal proliferation of lymphoid precursor cells.^1^ ALL comprises B‐cell ALL (B‐ALL), T‐cell ALL (T‐ALL) and ambiguous lineage ALL, with B‐ALL accounting for nearly 80% of all lymphoblastic leukaemia cases.^2^ Precise molecular subtyping of ALL can benefit patients by directing the course of risk‐adapted therapy. With the continuous development of modern sequencing technology, several new oncogenic fusions, including *DUX4* fusions in B‐cell progenitor acute lymphoblastic leukaemia (BCP‐ALL), have been identified.^3^, ^4^ Approximately 4% of BCP‐ALL patients harbour *IGH::DUX4*,^5^, ^6^ with most cases displaying a DUX4 reverse insertion in the enhancer region located upstream of the immunoglobulin heavy chain IGH. Analysis of *IGH::DUX4* patient samples revealed that the N‐terminal DNA‐binding domains HD1–HD2 were retained in the oncogenic fusion, while the C‐terminal domain was truncated and infused with a short tail of IGH fragment with the size up to 41 residues.^7^, ^8^

Currently, there have been some significant advances in the investigation of *IGH::DUX4* leukaemia. Research reports have demonstrated that IGH::DUX4 induced abnormal modifications in gene transcription.^9^ As an instance, the binding of IGH::DUX4 to the sixth intron of E‐26 transformation‐specific (ETS) familyrelated gene (*ERG)* leads to the conversion of *ERG* into an atypical exon 1, ultimately resulting in the transcription of ERG_alt_. This impeded the expression of wild‐type (WT) ERG and disrupted its original transcriptional activity, acting as a second‐hit event in leukaemogenesis.^5^ Moreover, CD371 expression was found to be notably higher in patients with IGH::DUX4 leukaemia than in other subtypes.^10^ This marker was associated with the C‐type lectin domain family 12 member A (*CLEC12A*), and our previous study identified a splicing variant of CLEC12A called CLEC12A_alt_ that was induced by IGH::DUX4 and may potentially correlate with disease prognosis.^2^ In general, genetic transcription disorders caused by oncogenes often exacerbate full‐blown leukaemia. Oncogenes recruited cofactors to form transcription complexes, thereby modulating downstream gene expression.^11^ Dysregulation of the cofactors (i.e., mutations or translocation) could result in the alteration of the transcription complexes, and potentially contribute to leukaemogenesis.^4^, ^12^ For example, TATA‐box binding protein (TBP)‐associated factors could facilitate transactivation by assisting oncogenic transcription factor (TF) fusions in localising to the promoter sites of their downstream aberrant transactivation, a prerequisite of leukaemogenesis.^13^ Abnormalities of transcriptional cofactors are also important in ALL. The mutations in cAMP responsive element binding protein (CREB)‐binding protein (CBP) and EP300 helped to reduce CBP acetyltransferase activity leading to downregulation of target genes.^12^ In our previous investigation, we identified distinct splicing events of transcription factor 12 (TCF12) in the IGH::DUX4 subgroup of B‐ALL.^14^ This discovery, along with transcriptomic data analysis from 1223 patients,^15^ indicated that TCF12 might act as a cofactor in the abnormal transcriptional function and disease development of *IGH::DUX4* leukaemia. TCF12 was an essential transcription factor that played a crucial role in neural development, embryonic development and cell differentiation.^16^ Prior research demonstrated that TCF12 was associated with disease, and the mutations in critical sites or changes in its transcriptome could impede its normal function and accelerate disease advancement.^17^, ^18^ However, in spite of the above breakthroughs, the role of cofactors such as TCF12 in the pathogenesis of IGH::DUX4 leukaemia remained unknown at the molecular level.

To explore TCF12's role as a cofactor in *IGH::DUX4* leukaemia, a range of experimental approaches were employed in this study. These included sequencing RNA‐sequencing (RNA‐seq) and ChIP‐sequencing (ChIP‐seq) data analysis, luciferase assays, co‐immunoprecipitation (Co‐IP) experiments, mammalian two‐hybridisation experiments, proximity ligation assay (PLA) experiments, *TCF12* knockout assays as well as AI‐based structural simulation and site‐directed mutagenesis. Through these characterisations, we discovered that IGH::DUX4 triggered abnormal upregulation of *TCF12*, which could then directly interact with IGH::DUX4 to facilitate IGH::DUX4‐driven transcriptional activation. In this leukaemia subtype, TCF12 could act as a cofactor in regulating the transcriptional mechanism of *IGH::DUX4* via a positive self‐feedback regulatory interplay between IGH::DUX4 and TCF12. These findings not only represent a significant step forward in the understanding of IGH::DUX4‐driven leukaemogenesis, but also open a new direction in future drug targeted therapy against the cofactor of oncogenic driver in ALL.

## 2 MATERIALS AND METHODS

### 2.1 RNA‐seq data analyses

All downstream analyses for sequencing data (RNA‐seq and ChIP‐seq) processing were performed with R (versions 4.0.2 and 4.1). Transcript expressions for each sample were obtained by performing Salmon (version 1.4.0) on the paired‐end fastq files without mapping. The R package tximport was used to aggregate salmon abundance estimates at the transcript level to the gene level, which was then imported into the R package DESeq2 for normalisation and differentially expressed gene (DEG) analysis.^19^, ^20^, ^21^ The screening criteria for DEGs was: both |log2(foldchange)| >1 and adjusted *p*‐value <.05. R packages, including clusterProfiler, ComplexHeatmap, gplots, ggpubr, factoextra, simplifyEnrichment, trackViewer and ggplot2, were used for visualisation. Gene set enrichment analysis (GSEA) was performed with GSEA command line tools.^22^, ^23^, ^24^, ^25^ The large‐scale bulk RNA‐seq dataset from our previous study was analysed through the Seurat basic pipeline, where each sample was treated as one single cell.^2^ RNA‐seq data of IGH::DUX4 was obtained from prior research work in our laboratory.^2^

### 2.2 ChIP‐seq data analyses

The ChIP assay was conducted based on the previously described protocols with certain modifications.^26^ In brief, we commenced by thoroughly amalgamating 1 × 10^5^ cells with a sufficient quantity of ConA magnetic beads for binding. Subsequently, incubation was performed at 4°C overnight with primary antibodies specific to IGH::DUX4 (ab124699, diluted at 1:50, Abcam) or immunoglobulin G (IgG) antibodies (12‐370, Millipore). Following this, further incubation was carried out for .5–1 h with IgG secondary antibodies (AP132, Millipore) mixed in dig wash buffer. After undergoing a washing procedure, a 100 μL pA‐Tn5 mixture was filled at 25°C for 1 h. Afterward, the cells were washed with dig‐med buffer and resuspended in tagmentation buffer (containing 10 mmol/L MgCl_2_ in dig‐med buffer). Subsequently, DNA purification was carried out through the process followed by ethanol precipitation. The extracted DNA was subjected to polymerase chain reaction (PCR) reactions and processing, and ultimately, sequencing was performed on the obtained DNA. The amplified libraries were assessed and sequenced using the Agilent 4200 TapeStation (Agilent) and Illumina Novaseq 6000 (150 bp paired‐end) (Illumina), respectively. ChIP‐seq fastq files were analysed using a modified chilin2 pipeline.^27^ Briefly, several steps were performed after quality control, including Bowtie2 mapping, fragment size estimation and peak calling.^28^ And FRiP score was calculated for peak calling evaluation. Deeptools was used for ChIP‐seq visualisation and the R package ChIPseeker was used for peak annotation and visualisation.^29^, ^30^ For motif finding, both HOMER and MDSeqPos were used. R packages, including universal motif, motifStack, ggtree and ggseqlogo, were used to perform motif analyses and visualisation.^29^, ^30^, ^31^ The vector group was designated as the control group, while the IGH::DUX4 group was designated as the treatment group. For peak calling, empty vector was used as control in MACS3. The program findMotifsGenome.pl in HOMER was used for motif discovery where the top 5000 records sorted by adjusted *p*‐values in the peak bed file outputted by MACS3 were used. The ChIP‐seq data were derived from prior research work in our laboratory.^2^

### 2.3 Integrated analyses of RNA‐seq and ChIP‐seq

The RNA‐seq and ChIP‐seq data were subjected to an integrated bioinformatics approach to identify IGH::DUX4 bound and regulated target genes (see more in Section 3). A maximum distance of 50 kb was used to define the association between each binding peak and its nearest DEG. All DEGs located from 50 kb upstream or downstream of the peak were first extracted. If multiple genes were found, the nearest gene to the peak was chosen. BETA was used to re‐check the defined targets.^32^

### 2.4 Cells culture, plasmids and transfection

Leukaemia REH cells were originally obtained from a 15‐year‐old female. NALM‐6 leukaemia cells were originally obtained from a 19‐year‐old male individual. Of note, the REH cells did not contain any endogenous IGH::DUX4. In comparison, the NALM‐6 cells displayed stable/endogenous expression of IGH::DUX4 fusion. All these cells were purchased from the Cell Bank Academy of Science (China) and grown in corresponding medium supplemented with foetal bovine serum (FBS) at 37°C in 5% CO_2_. The human *TCF12* gene was expanded from REH cells, subsequently was cloned into the LEGO‐iG2 and pBIND vector. Amplification of cDNA fragments encoding full‐length IGH::DUX4 from B‐ALL patients and cloning into the lentiviral vector LEGO‐iG2 and pACT. *TCF12* knockdown sequences were cloned into the pLKO.1 puro vector (Youbio). The primers are shown in Table S1. CRISPR/Cas9 knockout plasmids against human *TCF12* were synthesised and cloned into YKO‐LV002‐RFP vector (Ubigene Biosciences Co., Ltd.). The detailed information of primers is shown in Table S2. YCas‐LV002 plasmid was purchased from Ubigene Biosciences Co., Ltd. In this report, we also established a stable Cas9‐based *TCF12* knockout NALM‐6 cell line (termed NALM‐6*^TCF12−/−^*), and subsequently conducted *TCF12*‐gRNA infection experiments on this platform. The NALM‐6 cells subjected to *TCF12* knockout were selected/pooled by flow cytometry. Western blot was used to monitor the knockout efficiency and outcome. Mutation induction was conducted using locus‐specific mutagenesis techniques (KOD‐401, TOYOBO). The plasmids were all further validated by sequencing. Lentiviral packaging was accomplished by employing a mixture of psPAX2, pMD2.G and RSV plasmids (Youbio). Overexpression and infection were facilitated using Lipofectamine 2000 (Invitrogen). Lentiviral particles harvested 72 h later were then introduced to REH and NALM‐6 cells via infection, supplemented with 10 μg/mL polybrene (Sigma–Aldrich).

### 2.5 Quantitative real‐time reverse transcriptase‐polymerase chain reaction

Quantitative real‐time reverse transcriptase‐polymerase chain reaction (qRT‐PCR) was employed to assess the expression levels of *AGAP1*, *CLEC12A*, *ERG_alt_*, *C6orf89_alt_* and *CLEC12A_alt_*. The primers (Table S3) were devised through the software Primer Premier 5.0 (Premier). Total RNA was extracted from cellular precipitates through the RNeasy Mini Kit (RC112, Vazyme). The synthesis of the first‐strand complementary DNA was accomplished using the Invitrogen Transcription SuperScript III RT Kit (Invitrogen). The reaction included 5 μL of SYBR Premix Ex Taq (2×), .2 μL of forward primer, .2 μL of reverse primer, .2 μL of ROX reference dye (50×), 1 μL of cDNA, 3.4 μL of H_2_O (total volume 10 μL) and SYBR Premix Ex Taq RR420A‐Tli RNase H Plus (Takara Clontech). The PCR protocol consisted of an initial denaturation at 95°C for 30 s, followed by 40 cycles of denaturation at 95°C for 5 s and annealing/extension at 60°C for 31 s. In our qRT‐PCR experiments, housekeeping gene named β‐actin was used for both internal control and normalisation in analysis. Each experiment was independently replicated three times. All data were calculated using the Sequence Detector Systems software (Applied Biosystems). Gene expression fold changes were analysed using the 2^–ΔΔCt^ method, where ΔCt values were compared to those of the control group. All data were analysed using the Sequence Detector Systems software (Applied Biosystems). Fold changes in gene expression were calculated using the 2^–ΔΔCt^ method, where ΔCt values were compared with those of the control groups.

### 2.6 Alphafold protein interaction prediction

The complex structure of IGH::DUX4 and TCF12 proteins was predicted at Shanghai Jiao Tong University's Center for High‐Performance Computing, using the multimer model in Alphafold v2.3.1.^33^, ^34^ The employed versions of the databases were as follows: pdb_mmcif, pdb_seqres, uniport and uniref 90. The remaining databases were utilised in their default configurations. Visualisation was carried out using PyMOL to represent the cartoon/electrostatic surface and atomic.

### 2.7 Co‐immunoprecipitation

REH cells expressing HA–IGH::DUX4 were cultured for 48 h. Subsequently, cells were harvested and lysed with pre‐chilled radio immunoprecipitation assay (RIPA) buffer. The clear lysate was incubated overnight with beads coated with anti‐HA antibody (ab9110, 1:500 dilution; Abcam). The precipitant pulled down by anti‐HA‐antibody was further analysed using anti‐TCF12 antibody (sc‐28364, 1:500 dilution). Concurrently, the lysate was incubated overnight with beads coated with anti‐TCF12 antibody. The precipitant pulled down by the anti‐TCF12 antibody was further analysed with anti‐HA‐antibody. Anti‐mouse IgG (sc‐2025,1:500 dilution; Santa Cruz Biotechnology) and anti‐rabbit IgG (2729, 1:500 dilution; Cell Signaling Technology) were utilised as negative controls.

### 2.8 Mammalian two‐hybridisation assay

This experiment was conducted in HEK‐293T cells using the CheckMateTM Mammalian two‐hybridisation system (Promega). To examine the interaction between IGH::DUX4 and TCF12, the cDNA of *IGH::DUX4* and *IGH::DUX4*(R76A/R79A/R80A) was cloned into pACT vector. The cDNA of *RAG1*, *TCF12, TCF12*(D393A/E394A) and *PML* were constructed into the pPBIND vector. *RAG1* was served as the positive control, while *PML* was served as the negative control. HEK‐293T cells were co‐transfected with a mixture of pG5‐luc, pBIND‐*RAG1*, pBIND‐*TCF12*, pBIND‐*TCF12*(D393A/E394A), pBIND‐*PML*, pACT‐*IGH::DUX4* and pACT‐*IGH::DUX4*(R76A/R79A/R80A) using Lipofectamine 2000 (Invitrogen) to obtain transfected HEK‐293T cells for 48 h.

### 2.9 Dual‐luciferase reporter assay

HEK‐293T cells were seeded in a 48‐well plate. After 24 h incubation, the cells were co‐transfected with 200 ng/well pGL3‐basic promoters plasmids containing either ERGalt or AGAP1 promoter sequence, 200 ng/well expression LEGO‐iG2 plasmids containing IGH::DUX4 and/or TCF12, 2 ng/well internal control SV40 reporter plasmids using Lipofectamine 2000T (Invitrogen). Ensure uniform plasmid quality among the various experimental cohorts. The firefly luciferase activity was measured 24 h later using the dual‐luciferase reporter gene assay according to the manufacturer's instructions (Beyotime Biotechnology).

### 2.10 In situ proximity ligation assay

In PLA assay, we conducted experiments involving the overexpression of HA–IGH::DUX4 and HA–IGH::DUX4(R76A/R79A/R80A) in the transfected REH cells. After harvesting the cells, .5 × 10^6^ REH cells were centrifuged onto glass slides at 1000 rpm for 5 min. The cells were then fixed with 4% paraformaldehyde for 15 min, followed by permeabilisation with .5% Triton X‐100 for 20 min at room temperature. The blocking solution was thoroughly mixed and added to the cells for 90 min at 37°C. Subsequently, the cells were incubated overnight with primary antibodies against HA (diluted to 1:200; Abcam ab9110) and TCF12 (diluted to 1:50; Santa Cruz Biotechnology; sc‐28364) in a wet dark box. The aforementioned procedure was utilised in the experiment carried out on NALM‐6 cells that overexpressed HA‐TCF12 and HA‐TCF12(D393A/E394A). Primary antibodies against HA (diluted to 1:200; Abcam ab9110) and IGH::DUX4 (obtained from Santa Cruz Biotechnology; sc‐376490, diluted to 1:50) were employed. The PLA probe incubation was prepared by mixing 8 μL each of PLA probe minus and plus with 24 μL of antibody diluent, which was then added to the slides and incubated for 1 h at 37°C in a humidified chamber. Afterward, the ligation solution was applied to the slides and incubated for 30 min at 37°C in a preheated humid chamber. Simultaneously, the amplification solution was added to the slide and incubated for 100 min at 37°C. Finally, the slide was processed for imaging.

### 2.11 Western blotting

REH and NALM‐6 cells were cultivated in 75 cm^2^ tissue culture flasks to a certain density and then infected with relevant plasmid system. After incubation at 37°C for 6 h, the viral medium was substituted with a fresh medium containing 10% FBS. After 48 h of treatment, cells in each flask were lysed on ice with RIPA lysis buffer (Sangong Biotech). Samples were centrifugated for 5 min at 1.4 × 10^3^ g, 4°C, and protein concentrations were measured using the BSA Protein Assay Kit (Sangon Biotech). Proteins were denatured at 100°C for 10 min and then separated using 10% SDS‐PAGE gels. The proteins obtained from the gel were transferred onto a polyvinylidene fluoride (PVDF) membrane using the Bio‐Rad wet electro‐blotting system (Bio‐Rad). Following this, the membrane was blocked in a 1% BSA and 5% skim milk solution in 1× Tris‐buffered saline Tween (TBST) for 2 h. Each membrane was then individually incubated with one of the following primary antibodies: anti‐HA, anti‐TCF12 or mouse monoclonal anti‐GAPDH antibody. After 2 h of incubation on a rocker, the membrane was washed thrice with 1× TBST and subsequently reacted with the secondary antibody for an additional 2 h. Finally, the blot was treated with ECL reagent for 5 min to enable Chefluorescence imaging.

### 2.12 Cell proliferation analyses

For each well of a 96‐well plate, we added 5000 NALM‐6 cells in groupings with a final volume of 100 μL. These plates were then cultured at 37°C with 5% carbon dioxide for 24, 48, 72 and 96 h, respectively. Subsequently, cell counting kit‐8 (CCK‐8) reagent (10 μL per well, Vazyme) was added at 0, 24, 48, 72 and 96 h, followed by a 2‐h incubation at 37°C. Finally, we employed a microplate reader to measure the absorbance values of the various cell groups at 450 nm.

### 2.13 Apoptosis assay

Firstly, the 10× binding buffer was diluted to 1× with distilled water (mix 1 mL of 10× binding buffer with 9 mL of ddH_2_O). Following that, the cells were performed a wash with phosphate‐buffered saline and then another wash with 1× binding buffer. The cells were then re‐suspended in 1× binding buffer to reach a final concentration of ∼5 × 10^6^ cells/mL. Afterward, 5 μL of Annexin V fluorescent dye was added to 100 μL of the cell suspension, followed by incubation at 25°C for 15 min. Next, the cells were washed with 1× binding buffer, and re‐suspended in 200 μL of the same buffer. Finally, 5 μL of propidium iodide staining solution (Invitrogen) was added for flow cytometry analysis.

### 2.14 In vitro drug sensitivity experiment

The VDCLP protocol (comprising vincristine, daunomycin, cyclophosphamide, asparaginase, and dexamethasone) are frequently used in the treatment of ALL.^35^ Furthermore, an important contributing factor to relapse in patients with ALL is the development of drug resistance in tumour cells. Here, we selected vincristine, the most effective chemo agent in the clinical management of B‐ALL, to monitor the IGH4::IGH–TCF12 cooperation in drug resistance.^36^, ^37^ For in vitro drug sensitivity experiment, 10 000 cells per well were seeded in a 96‐well plate and subjected to a gradient of vincristine concentrations for 48 h at 37°C. The gradient of vincristine ranged from 0 to 7 nM and all samples were assessed in triplicate. After the 48‐h incubation, 10 μL of CCK‐8 reagent was added to each well and incubated at 37°C for 2 h. Following the CCK‐8 incubation, absorbance values at a wavelength of 450 nm (A450) were measured using an enzyme‐linked immunosorbent assay reader. Curve fitting analysis was used to determine drug sensitivity and calculate the IC50 value.^38^

### 2.15 Statistical analyses

All data from wet experiments were presented as mean ± standard deviation. Statistical analysis of wet experiments was conducted using SPSS for Windows 19.0 and Sigma Plot version 12.5 (Systat Software). One‐way analysis of variance was employed, followed by post hoc Tukey's test or unpaired two‐tailed *t‐*test to determine the significance of difference between two groups. In all statistical analyses, a significance level of *p* < .05 was considered statistically significant.

## 3 RESULTS

### 3.1 TCF12 was highly expressed in DUX4 fusion patients

**FIGURE 1:**
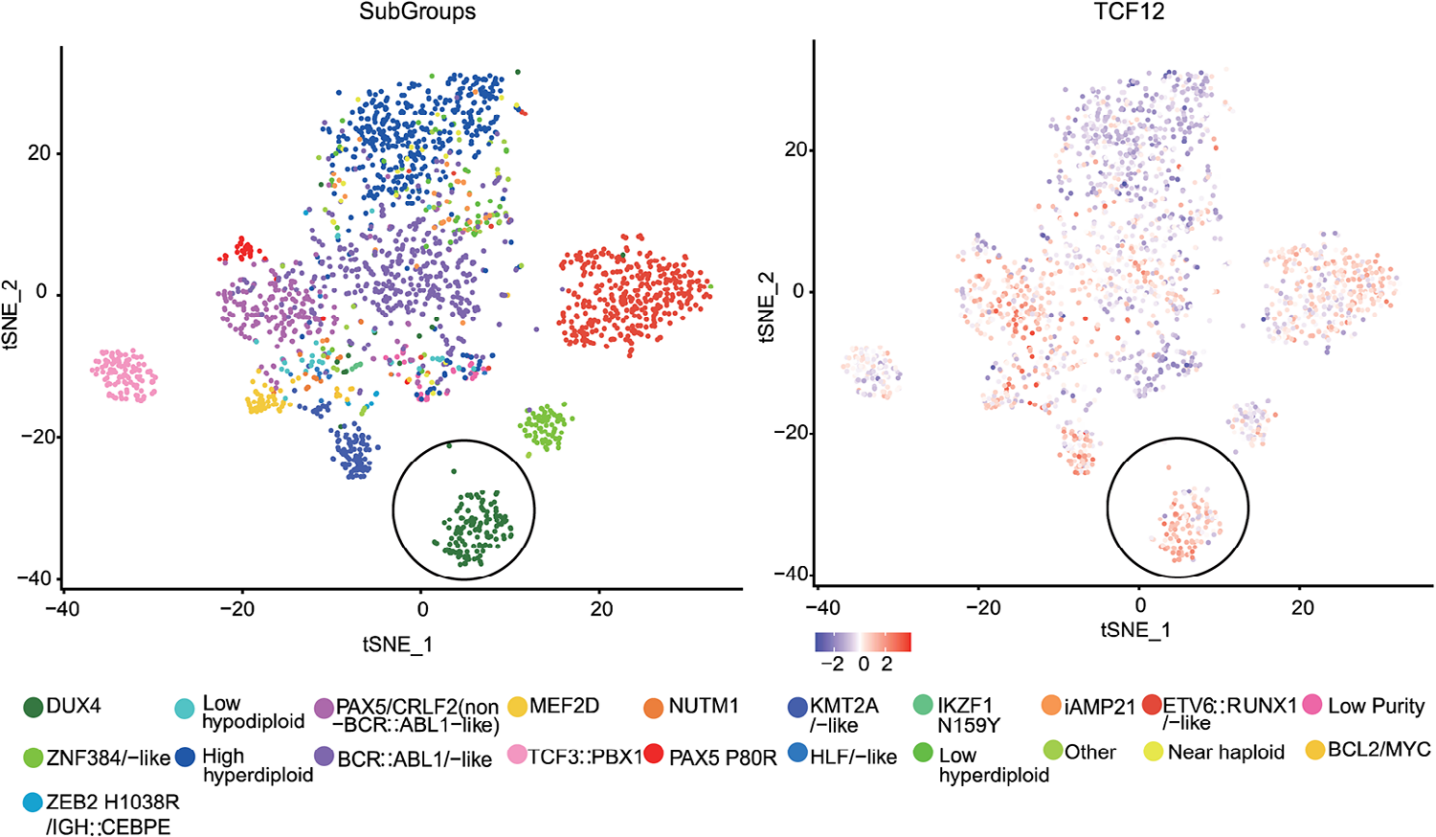
Expression levels of transcription factor 12 (TCF12) in patients with various B‐cell acute lymphoblastic leukaemia (B‐ALL) subtypes. *TCF12* upregulation in IGH::DUX4 B‐ALL patients. The *t*‐distributed stochastic neighbour embedding (*t*‐SNE) analysis was performed using bulk RNA‐sequencing (RNA‐seq) data obtained from 1223 B‐ALL patients. The B‐ALL subtypes with high expression of both TCF12 and DUX4 fusions were encircled. The different leukaemia subgroups were represented using distinct colour schemes.

Precise subtyping based on molecular abnormalities can provide clinical guidance for the diagnosis and targeted treatment of leukaemia.^15^ RNA‐seq data from multiple studies, incorporating 1223 patients, have helped to identify several subtypes of leukaemia defined by gene fusions or mutations, each characterised by distinct gene expression patterns. Using the gene expression profile from 1223 B‐ALL patients,^15^ we investigated dysregulated genes in the *DUX4* subgroup. The dysregulated genes were identified in the two‐dimensional (2D) representation of the gene expression profile following *t*‐distributed stochastic neighbour embedding dimensionality reduction (Figure 1), wherein patients with *DUX4*‐fused leukaemia exhibited pronounced upregulation of *TCF12*. Moreover, through quantitative analysis, we observed that *DUX4* patients exhibited notably high expression of TCF12 in comparison to the majority of other B‐ALL subtypes (Figures 1 and S1A). However, TCF12 expression was only moderately increased, compared to other groups such as *NUTM1*, *KMT2A/Like*, *HLF/Like*, *PAX5/CRLF2* and *IKZF1::N195Y* (Figure S1A). Based on gene expression profiling, ‘*HLF‐like*’ was also referred to the *TCF3/TCF4‐HLF* fusion. *PAX5/CRLF2* fusion exhibited distinctive gene expression profile with intermediate risk.^15^ In order to further validate the in silico results, we selected *IGH::DUX4*, *ETV6::RUNX1* and *MEF2D::HNRNPUL1* subgroups to monitor TCF12 expression at both the transcription and protein levels. The outcomes demonstrated that TCF12 was exclusively overexpressed in IGH::DUX4 compared to the *ETV6::RUNX1* and *MEF2D::HNRNPUL1* groups (Figure S1B,C).

### 3.2 IGH::DUX4 bound to genomic DNA and caused transcriptomic changes

The above investigation demonstrated the elevated expression of TCF12 in patients with *DUX4*‐type leukaemia. This observation provoked an in‐depth investigation into the significance of high TCF12 expression in the pathogenesis of *DUX4* fusion‐type leukaemia, as well as the interplay between IGH::DUX4 and TCF12.

**FIGURE 2:**
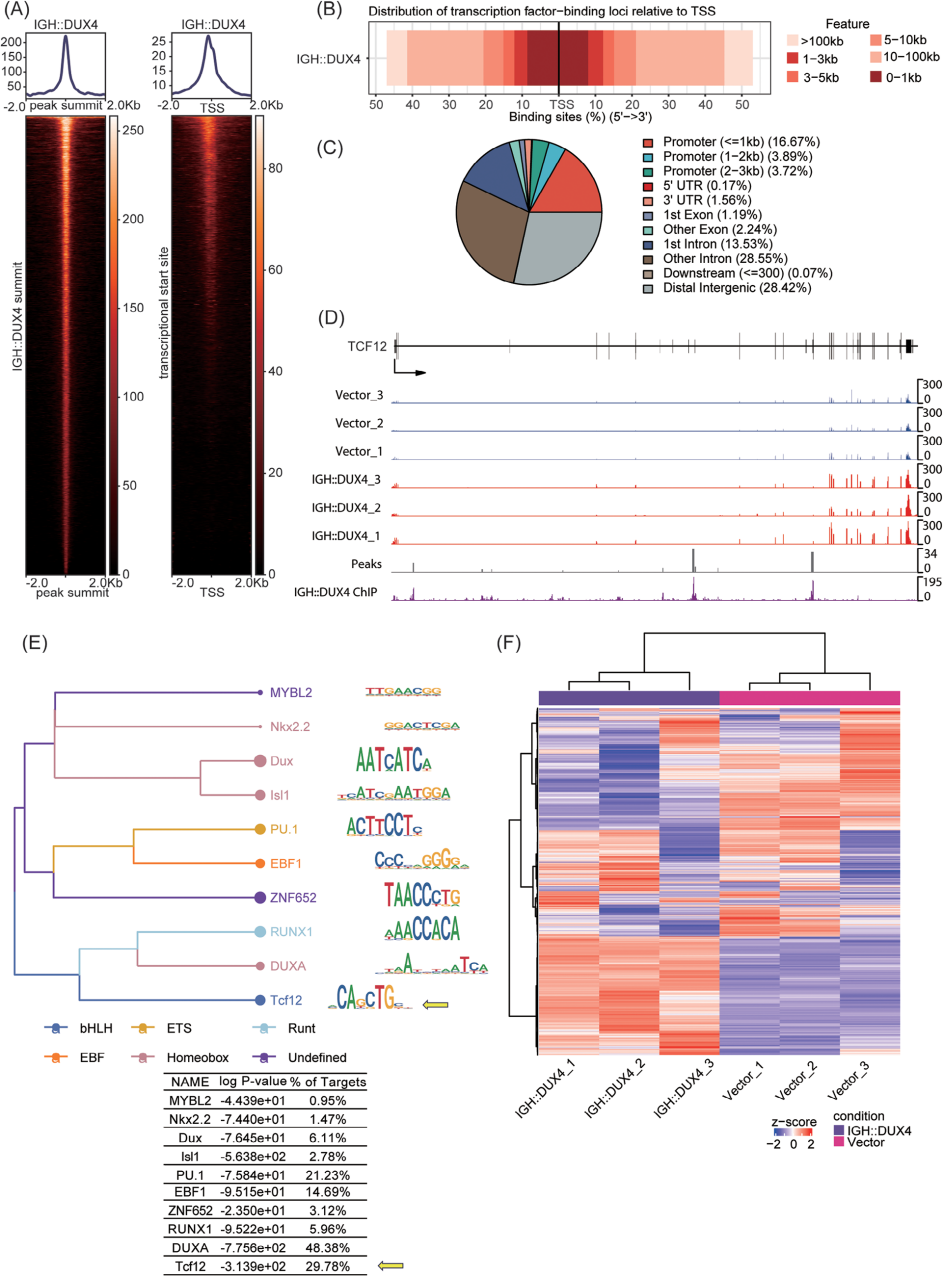
IGH::DUX4 bound directly to transcription factor 12 (TCF12) gene and overlapped with TCF12 responsive element. (A) ChIP‐sequencing (ChIP‐seq) analysis of REH cells with stable IGH::DUX4 expression. ChIP‐seq heatmaps (bottom) and profiles (top) at peak and transcription start site (TSS) regions. (B) Distribution of IGH::DUX4 binding loci relative to TSS. Darker red represents closer distance to TSS. (C) IGH::DUX4 peak annotation. Various genomic features such as promoter, intron, exon regions, etc., were coloured differently and labelled with percentages. (D) IGH::DUX4‐driven TCF12 deregulation. RNA‐sequencing (RNA‐seq), ChIP‐seq and the peak bed were plotted in the track view, in which empty vector was used as control. (E) The phylogenetic tree of de novo discovered motifs (top panel) and their statistical table (bottom panel). Colours of branches and points represent corresponding transcription factor (TF) families. TCF12 responsive element (TRE) was highlighted with yellow arrows. (F) RNA‐seq heatmap showing the expression of 5% genes including TCF12 with the greatest variance in all the six samples. Genes showing upregulations and downregulations in the heatmap are shown in red and blue, respectively.

First, we analysed the IGH::DUX4 ChIP‐seq data in REH cells. More than 92% and 85% of the reads in the treatment and control groups were uniquely mapped to the hg38 genome, with a total of 14 114 peaks identified. ChIP‐seq signals and peak profiles showed IGH::DUX4 could not only bind to positions around the transcription start site (TSS), but also to other genomic regions (Figure 2A). Almost 24.3% of IGH::DUX4 peaks were annotated as promoters, and the majority (56.97%) of the peaks were annotated as introns and distal intergenic regions (Figure 2B,C). It has been suggested that the intronic binding was important for the aberrant splicing initiated by IGH::DUX4.^2^ IGH::DUX4 could bind to several key genes of *DUX4r* B‐ALL (Figure S2) as well as *TCF12* (Figure 2D). With the peaks, 10 de novo motifs were identified by Homer2 after filtering (*p*‐value ≤ 1e‐10) (Figure 2E). Most of these motifs includin*g* DUXA and Tcf12 belonged to the TFs homeobox and bHLH families. As expected, 48.38% of the IGH::DUX4 peaks contained the DUXA (a homologue to DUX4) motif sequence. The next closely related motifs came from TFs PU.1 (a crucial factor in both hematopoiesis and leukaemia) and Tcf12, bearing 21.23% and 29.78% similarities to IGH::DUX4, respectively. Genes, directly bound/regulated by IGH::DUX4, were subjected to enrichment analysis using the signalling pathways published in Kyoto Encyclopedia of Gene and Genome (KEGG) database (Figure S3A). Enrichment analysis also suggested that IGH::DUX4 could bind and deregulate genes associated with cell adhesion, lymphocyte activation and differentiation (Figure S3A,B). Next, it was checked whether the IGH::DUX4 binding genes were also frequently observed in other human diseases. The IGH::DUX4 target genes were crosschecked against the DisGeNET database, in which the dynamics between human diseases and their causing genes were discussed. Interestingly, the IGH::DUX4 target genes were often associated with the human disease related to precursor B‐cell lymphoblastic leukaemia (Figure S3A), reiterating the driving role of this potent oncogenic driver.

IGH::DUX4 could lead to significant transcriptomic changes, monitored by RNA‐seq analysis (Figure 2F). By following the thresholds mentioned in the methods, 1988 DEGs were obtained, with 1195 upregulated and 793 downregulated. The different genes directly bound and regulated by IGH::DUX4 are listed in Table S4. For instance, *CLEC12A* (the cell lectin) was significantly upregulated, which was consistent with previous studies.^10^ Furthermore, the IGH::DUX4 cells expressed distinct patterns of T‐cell and B‐cell markers (Figure S4A,B). CD9 was upregulated in IGH::DUX4. In BCP‐ALL, CD9‐positive cases were more severe than CD9‐negative cases. CD37 was usually expressed in mature B cells, upregulated in other haematologic diseases, and downregulated in IGH::DUX4. The enrichment analysis suggested that the abnormal deregulation of CD9 and CD37 expression might be coupled with aberrant activation of Wnt signalling and GTPase functions (Figure S4C), accompanied by suppressed functions in leukocyte adhesion and B cell antigen receotor (BCR)‐related pathways (Figure S4D).^39^, ^40^ Recently, Zhang et al. demonstrated that IGH::DUX4 can recruit RAG1/2 for alternative splicing.^2^ Here, the alternative splicing pathways were also significantly upregulated in IGH::DUX4 cells (Figure S5A,B), reflecting the importance of IGH::DUX4–RAG1/2 axis in oncogenic splicing.^2^

### 3.3 TCF12 was a target of IGH::DUX4 and shared binding sites with IGH::DUX4

**FIGURE 3:**
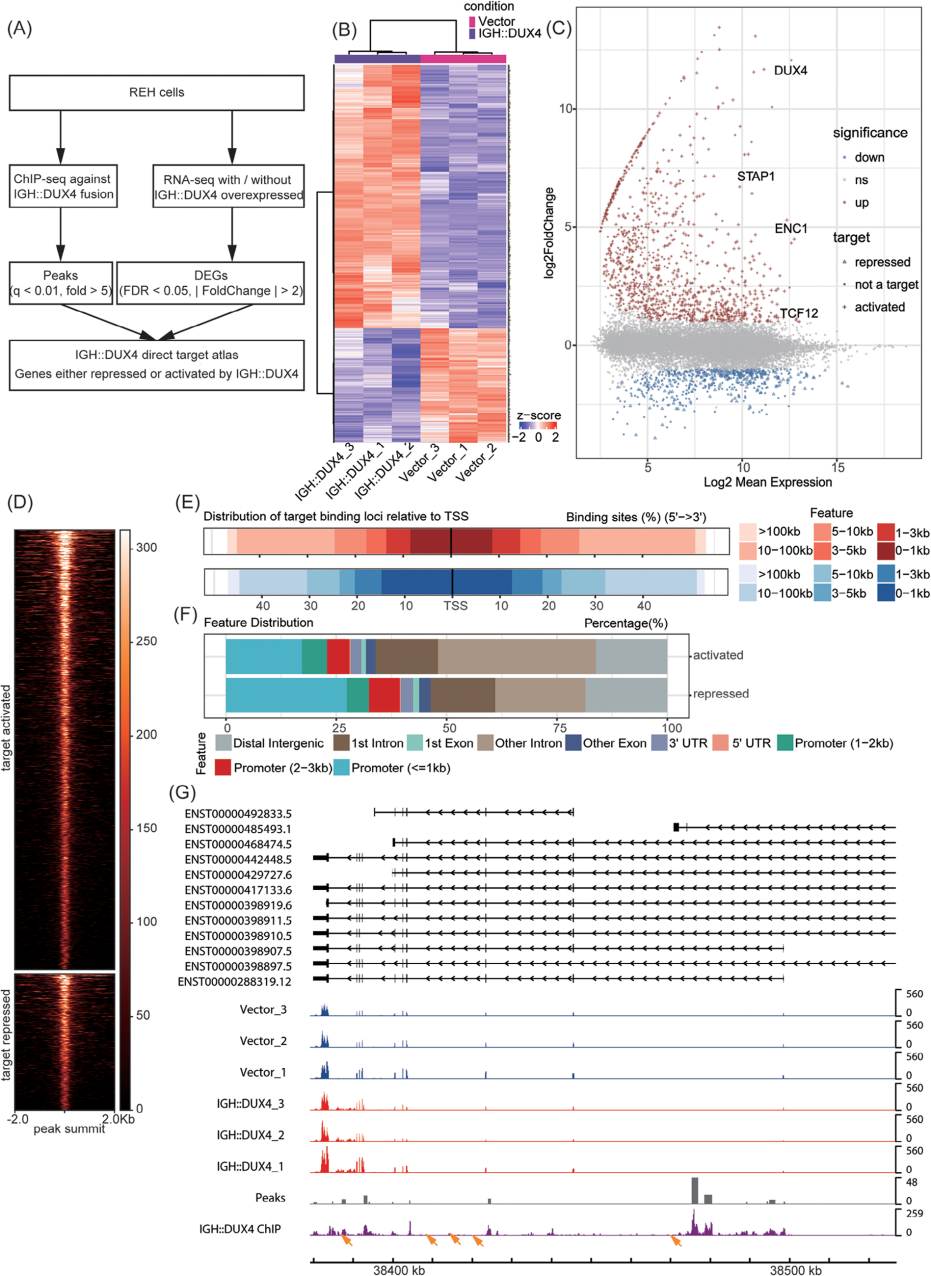
ChIP‐sequencing (ChIP‐seq) and RNA‐sequencing (RNA‐seq) integrated analyses revealed cooperation potential between IGH::DUX4 and transcription factor 12 (TCF12). (A) A schematic diagram of ChIP‐seq and RNA‐seq integrated analyses used in this study. The peak and differentially expressed gene (DEG) criteria used for this analysis were annotated. (B) Heatmap analysis of IGH::DUX4‐driven TCF12 deregulations in REH cells containing IGH::DUX4. Empty vector was used as control. All direct target genes identified by ChIP analysis were used for visualisation. (C) Minus‐versus‐add (MA) plot of IGH::DUX4 target genes. Each point represented a single gene, which was subsequently coloured according to its statistical significance. Target genes activated by IGH::DUX4 are shown with symbol ‘+’. The repressed genes are shown with symbol ‘Δ’. (D) ChIP‐seq heatmap showing signals at peaks in the vicinity of IGH::DUX4 targets. Both activated and repressed targets were included and the distribution of activated targets was broader. (E) Distribution of binding loci of the IGH::DUX4 activated targets (top and red) and repressed targets (bottom and blue) relative to transcription start site (TSS). Darker colours represent closer distance to TSS. (F) Genomic feature annotation of peaks in the vicinity of IGH::DUX4 targets. Different colours of the fractions represent different genomic features. (G) Transcriptional effects and binding pattern of IGH::DUX4 upon the *ERG* gene. The orange arrows were used to annotate the *TCF12* binding sites (i.e., TCF12 responsive element [TRE]) in these regions. RNA‐seq, ChIP‐seq and the peak bed are plotted in the track view. Empty vectors were used as control.

In order to characterise IGH::DUX4 target genes, ChIP‐seq and RNA‐seq data were subjected to integrated analysis, resulting in the identification of genes that IGH::DUX4 bound and regulated (Figure 3A). Here, we generated an IGH::DUX4 target atlas with 836 genes, including both 585 activated genes and 251 repressed genes (Figure 3B). IGH::DUX4 target genes were mapped back to all the DEGs, and ChIP‐seq profiles were delineated for peaks containing these genes (Figure 3C,D). We found that IGH::DUX4 preferred to bind to regions outside the TSS compared to repressed target genes, and intronic binding by IGH::DUX4 was increased when it came to activated targets (Figure 3E,F). Moreover, the consequences of activation mediated by IGH::DUX4 were more pervasive over the whole genome. Interestingly, we found that *TCF12* was an activated target gene for IGH::DUX4 (Figure 3C). Homer2 showed that there were 29.78% of the IGH::DUX4 peaks similar to the TCF12 motif sequence (Figure 2E). Consistently, MDSeqPos also displayed a similar result, suggestive of significant overlap between IGH::DUX4‐ and TCF12‐recognition elements. The composition of *TCF12* binding motif in *Mus musculus* and *Homo sapiens* are viewed in Figure S6. Their similarity was statistically significant (*p* = .00233239, comparison between JASPAR_mouse_1 and Cistrome in Figure S6). This, together with the observation of abnormally high expression of *TCF12* in *IGH::DUX4* patients (see results described above), led to the hypothesis that TCF12 might cooperate with IGH::DUX4 in its oncogenic transactivation activity. Supportively, the sequence scanning showed that the binding sites of *TCF12* were enriched around IGH::DUX4 peaks as observed in *ERG*, *C6of89*, *CLEC12A*, etc. (Figures 3G and S2).

### 3.4 TCF12 expression enhanced IGH::DUX4 transcriptional activity

**FIGURE 4:**
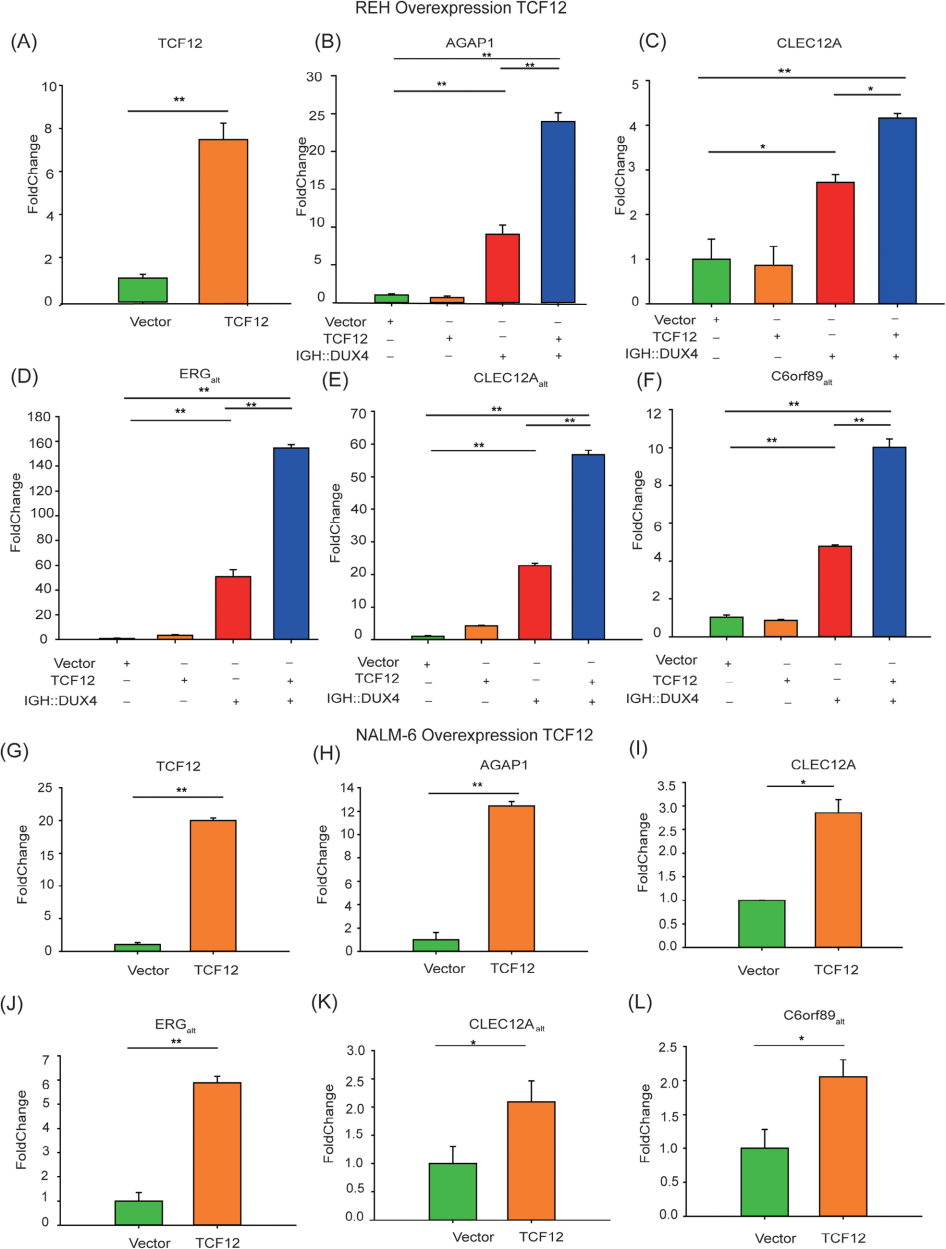
Transcription factor 12 (TCF12) enhanced IGH::DUX4‐driven transactivation in leukaemia REH and NALM‐6 cells. (A) Overexpression of TCF12 alone in leukaemia REH cells. The TCF12 expression was quantified against the control (empty vector) using quantitative real‐time reverse transcriptase‐polymerase chain reaction (qRT‐PCR). (B–F) Co‐expression of TCF12 and IGH::DUX4 in REH cells. The expression levels of IGH::DUX4 target genes such as *AGAP1* (B), *CLEC12A* (C), ERG_alt_ (D), CLEC12A_alt_ (E) and C6orf89_alt_ (F) in TCF12 infected cells (orange columns), IGH::DUX4 infected cells (red columns) and IGH::DUX4 + TCF12 infected cells (blue columns) were monitored by qRT‐PCR. The uninfected cells were used as a control (green columns). (G) Overexpression of TCF12 alone in NALM‐6 cells. The TCF12 expression was quantified against the control (empty vector, green column) using qRT‐PCR. (H–L) The expression levels of IGH::DUX4 target genes such as *AGAP1* (H), *CLEC12A* (I), ERG_alt_ (J), CLEC12A_alt_ (K) and C6orf89_alt_ (L) in TCF12 infected cells (orange columns). The data were presented as mean ± standard deviation (SD), and the experiments were independently conducted at least three times. ^*^ *p* < .05; ^**^ *p* < .01; ^***^ *p* < .001.

**FIGURE 5:**
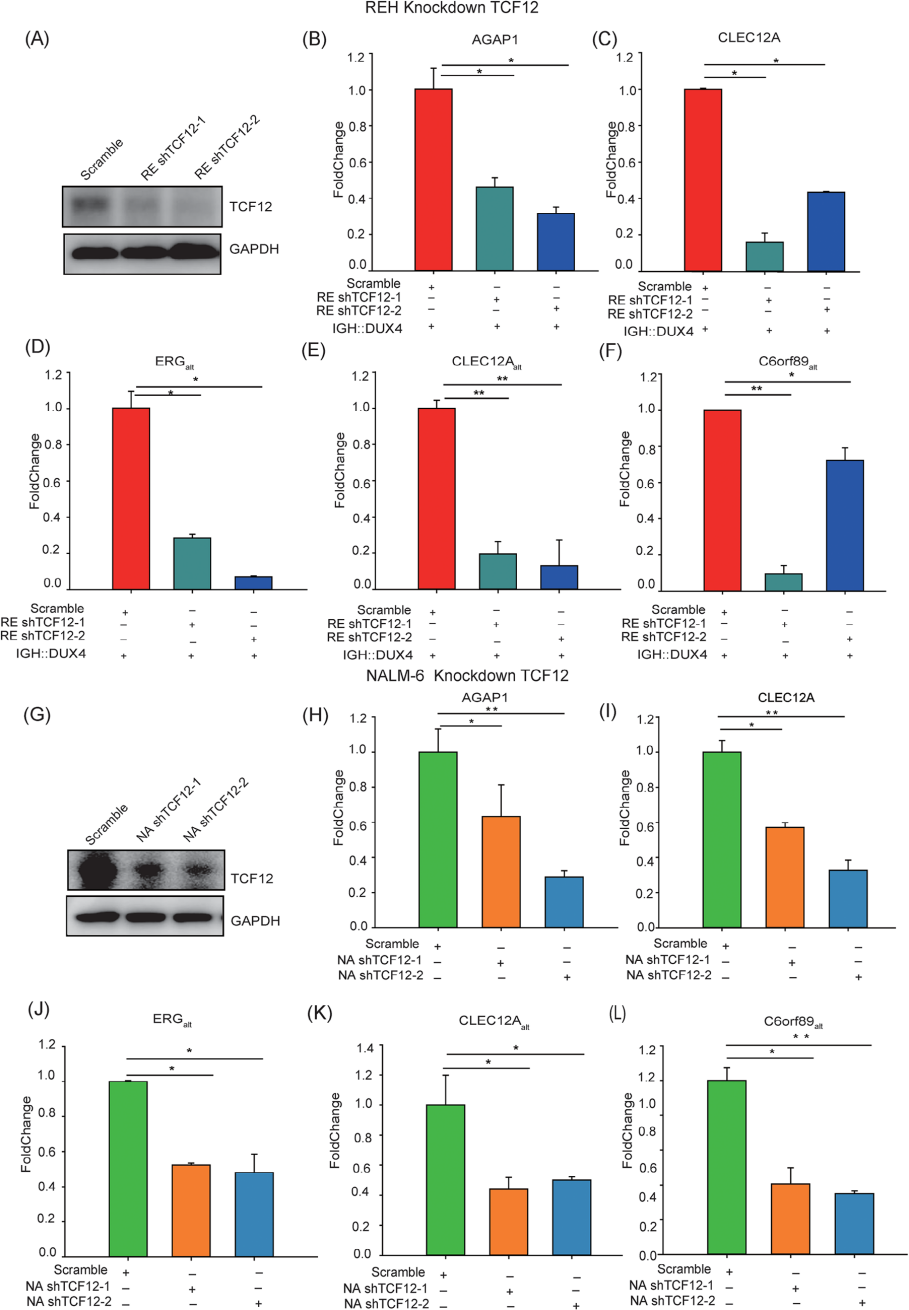
shRNA knockdown of transcription factor 12 (TCF12) in REH and NALM‐6 cells. (A) Western analysis of *TCF12* knockdown in REH cells. A randomly scrambled shRNA sequence (Scramble) was used as control. The details of shRNA sequences used in this study were shown in Table S2. (B–F) *TCF12* knockdown in REH cells with stable expression of IGH::DUX4. The IGH::DUX4‐driven transactions such as *AGAP1* (B), *CLEC12A* (C), ERG_alt_ (D), CLEC12A_alt_ (E) and C6orf89_alt_ (F) in control (red) and shTCF12 knockdown cells (dark green and dark blue) were monitored by quantitative real‐time reverse transcriptase‐polymerase chain reaction (qRT‐PCR). (G) shRNA knockdown of *TCF12* in NALM‐6 cells that contained an endogenous IGH::DUX4 fusion. Same shRNA sequences against *TCF12* described above were used. Western analysis using antibody against TCF12 was used to analyse the outcome of *TCF12* knockdown in NALM‐6 cells. (H–L) *TCF12* knockdown in NALM‐6 cells. The IGH::DUX4‐driven transactions such as *AGAP1* (H), *CLEC12A* (I), ERG_alt_ (J), CLEC12A_alt_ (K) and C6orf89_alt_ (L) were monitored by qRT‐PCR. Control and TCF12 knockdown results were shown in green, orange and light blue columns, respectively. The data were presented as mean ± standard deviation (SD), and the experiments were independently conducted at least three times. ^*^ *p* < .05; ^**^ *p* < .01; ^***^ *p* < .001.

To investigate whether and how TCF12 facilitated IGH::DUX4‐mediated transcription, the abnormal transcriptional activation of IGH::DUX4 targeted genes such as *AGAP1*, *CLEC12A*, *ERG_alt_, CLEC12A_alt_* and *C6orf89_alt_* was measured by qRT‐PCR in leukaemia cell lines REH and NALM‐6. AGAP1 is an inclusion‐related protein that activates the GTPase of ADP ribosylation factor in a phosphoinositol‐dependent manner, influencing the formation of the actin cytoskeleton.^41^ Previous studies have reported that *AGAP1* was highly expressed in *IGH::DUX4* types of leukaemia.^2^ CD371 is a protein associated with cell lectin CLEC12A expression, and studies have shown that CD371 is specifically highly expressed in *IGH::DUX4* patients.^10^ Furthermore, IGH::DUX4 triggers the aberrant transcripts such as CLEC12A_alt_ and C6orf89_alt_, which is identified in B‐ALL patients along with the secondary leukaemogenic hit ERG_alt_.^2^ In this study, we overexpressed IGH::DUX4 and TCF12 in REH and NALM‐6 cells, and measured the transcriptional expression levels of downstream target genes including *AGAP1*, *CLEC12A*, *ERG_alt_*, *CLEC12A_alt_* and *C6orf89_alt_*. First, using qRT‐PCR, we demonstrated that TCF12 was overexpressed in REH cells when compared to the control group (Figure 4A). Concerning the IGH::DUX4 target genes, co‐expression of TCF12 and IGH::DUX4 resulted in significantly enhanced expression levels of *AGAP1* and *CLEC12A*, when compared to the IGH::DUX4 alone group (Figure 4B,C). Supportively, the expressions of IGH::DUX4‐driven abnormal splicing including *ERG_alt_, CLEC12A_alt_* and *C6orf89_alt_* were consistently upregulated (Figure 4D–F). Supportively, similar results were obtained in NALM‐6 cells (Figure 4G–L), implicating that TCF12 acted as a cofactor in IGH::DUX4‐driven deregulation. Next, we wanted to check whether *TCF12* knockdown might have an impact on IGH::DUX4‐driven deregulation. The details of shRNA sequences used to knockdown *TCF12* expression in REH and NALM‐6 cells are listed in Table S1. Immunoblotting experiments with TCF12 antibody were carried out to check the protein suppression by *TCF12* knockdown in REH cells (Figure 5A). shRNA knockdown of endogenous TCF12 indeed impaired the expressions of IGH::DUX4 target genes such as *AGAP1*, *CLEC12A*, *ERG_alt_*, *CLEC12A_alt_* and *C6orf89_alt_* in REH (Figure 5B–F). The randomly selected shRNA sequence was employed as a control group. The results revealed that the scrambled shRNA sequence had little impact on the IGH::DUX4 transcriptional activity. Again, similar shRNA knockdown experiments were repeated in NALM‐6 cells that contained an endogenous IGH::DUX4 (Figure 5G). Consistent with the *TCF12* knockdown in REH cells, inhibition of *TCF12* expression caused damage to IGH::DUX4‐driven deregulations (Figure 5H–L).

### 3.5 Direct interaction between TCF12 and IGH::DUX4

**FIGURE 6:**
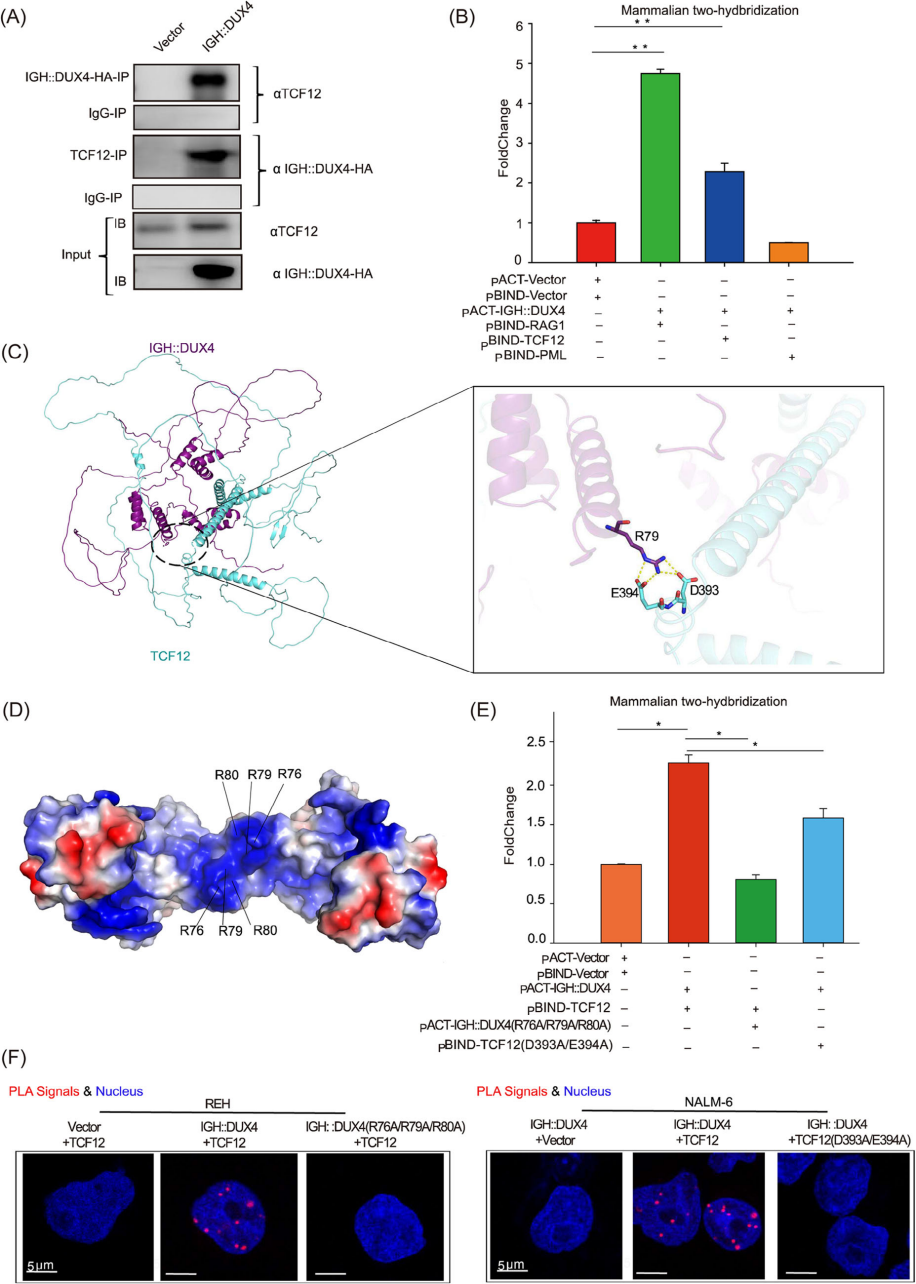
Characterisation of direct interaction between transcription factor 12 (TCF12) and IGH::DUX4. (A) Co‐immunoprecipitation (Co‐IP) assay. The interaction between IGH::DUX4 and TCF12 was detected by immunoprecipitation. HA–IGH::DUX4 was used as bait to pull down TCF12 using an antibody against HA tag (top two panels). Vice versa, when TCF12 was used as bait, the IGH::DUX4 was Co‐IP with antibody against TCF12 (middle two panels). The TCF12 and IGH::DUX4 inputs were shown in the bottom two panels. The immunoglobulin G (IgG) was used as negative control in these pull‐down assays. (B) Mammalian two‐hybridisation assay. The interaction between IGH::DUX4 and TCF12 was verified by mammalian two‐hybridisation experiments according to manufacturer's protocol. The pACT‐Vector:pBIND‐Vector interaction was normalised (= 1). In recent study, it was demonstrated that RAG1 protein interacted directly with IGH::DUX4,^2^ and hence was used as positive control. Untreated (blank, red column), RAG1 (positive control, green column), TCF12 (blue column) and nuclear protein PML (a randomly chosen protein as negative control, orange column) were used in this assay. (C) Structural simulation of IGH::DUX4–TCF12 complex. The multimer model/algorithm implemented in AlphaFold2 was used to predict the interaction between IGH::DUX4 (magenta) and TCF12 (cyan). Residues R79 (in IGH::DUX4) and D393/E394 (in TCF12) were shown in stick representation. Hydrogen bonds were shown in yellow dotted lines. (D) Electrostatic surface in IGH::DUX4 structure (PDB code: 7DW5). Blue for positively charged surfaces. Red for negatively charged surfaces. The putative binding sites delineated by R76/R79/R80 were annotated. (E) Structure‐based mammalian two‐hybridisation assay. The pACT‐Vector:pBIND‐Vector interaction was normalised (= 1). In addition to the WT IGH::DUX4–TCF12 interaction, IGH::DUX4(R76A/R79A/R80A)–TCF12 and IGH::DUX4–TCF12 (D393A/E394A) engagement were monitored. (F) In situ proximity ligation assay (PLA). The direct interaction between IGH::DUX4 and TCF12 in REH and NALM‐6 cells was visualised by fluorescently labelled complementary oligonucleotide probes. The data were presented as mean ± standard deviation (SD), and the experiments were independently conducted at least three times. ^*^ *p* < .05; ^**^ *p* < .01; ^***^ *p* < .001.

To further characterise the nature of TCF12‐mediated IGH::DUX4 transactivation, direct interactions between TCF12 and IGH::DUX4 were monitored by Co‐IP assay in REH cells. Firstly, the HA–IGH::DUX4 was used as bait to pull down TCF12 using an antibody against the HA tag. As shown in Figure 6A (two top rows), when IGH::DUX4 was pull‐down, a co‐precipitation of endogenous TCF12 was detected. Consistently, when TCF12 was used as bait, IGH::DUX4 was pull‐down with antibody against TCF12 (Figure 6A, two middle rows). To corroborate the in vitro Co‐IP results, the interaction between TCF12 and IGH::DUX4 was also monitored in leukaemia cells. The mammalian two‐hybridisation assay was carried out. The RAG1 protein was considered to interact with IGH::DUX4,^2^ and therefore, it was used as a positive control in this characterisation. An empty vector was selected as the negative control. The results were shown in Figure 6B. The basal interaction level observed between the empty pACT and pBIND vectors was set to 1 (termed control group in this assay). The interaction between RAG1 and IGH::DUX4 was 4.7, consistent with observation published elsewhere.^2^ In comparison, the mammalian two‐hybridisation reading between TCF12 and IGH::DUX4 was 2.3. This was significantly higher than that of the control group, supporting of a direct engagement between these two TFs (Figure 6B). Furthermore, the PML protein (i.e., a randomly chosen nuclear protein) was used as an extra level of control to check for the feasibility and specificity of this assay. As expected, PML mimicked the result of an empty vector, echoing a genuine crosstalk between TCF12 and IGH::DUX4. More importantly, the TCF12‐mediated IGH::DUX4 transactivation was repeatedly observed in the luciferase assay using HEK‐293T cells. The presence of TCF12 resulted in a marked enhancement of IGH::DUX4‐driven transcriptional activation (as monitored by ERG_alt_ and AGAP1 expression) when compared to IGH::DUX4 alone (Figure S7A,B).

To better understand the synergistic effect of how TCF12 might interact with IGH::DUX4, AI‐facilitated structural simulation of TCF12–IGH::DUX4 complex was used to envisage this engagement (Figure 6C). A highly positive charged pocket delineated by the conserved Arg residues (i.e., R76/R79/R80) was predicted to directly interact with the negatively charged residues, D393 and E394, in TCF12 (Figure 6C,D). More importantly, the TCF12–IGH::DUX4 simulated complex was further verified by structure‐based mutagenesis (Figures 6E,F and S7A,B). The R76A/R79A/R80A in IGH::DUX4 and D393A/E394A in TCF12 consistently disrupted IGH::DUX4–TCF12 interaction, as monitored by mammalian two‐hybridisation assay (Figure 6E). This result was echoed in PLA, in which the IGH::DUX4, but not mutant R76A/R79A/R80A, could interact with TCF12 in REH cells (Figure 6F, left panel). When mutation was introduced in TCF12, TCF12 (D393A/E394A) failed to engage the endogenous IGH::DUX4 in NALM‐6 cells (Figure 6F, right panel). Finally, luciferase assay with IGH::DUX4 and TCF12 mutants showed that perturbation of the interface between IGH::DUX4 and TCF12 abolished the TCF12‐enhanced IGH::DUX4‐mediated ERG_alt_ and AGAP1 transactivation (Figure S7A,B). Taken together, our experimental findings further supported the synergistic effect between IGH::DUX4 and TCF12.

### 3.6 Effect of TCF12 knockout on the transcriptome of IGH::DUX4 leukaemia cells

**FIGURE 7:**
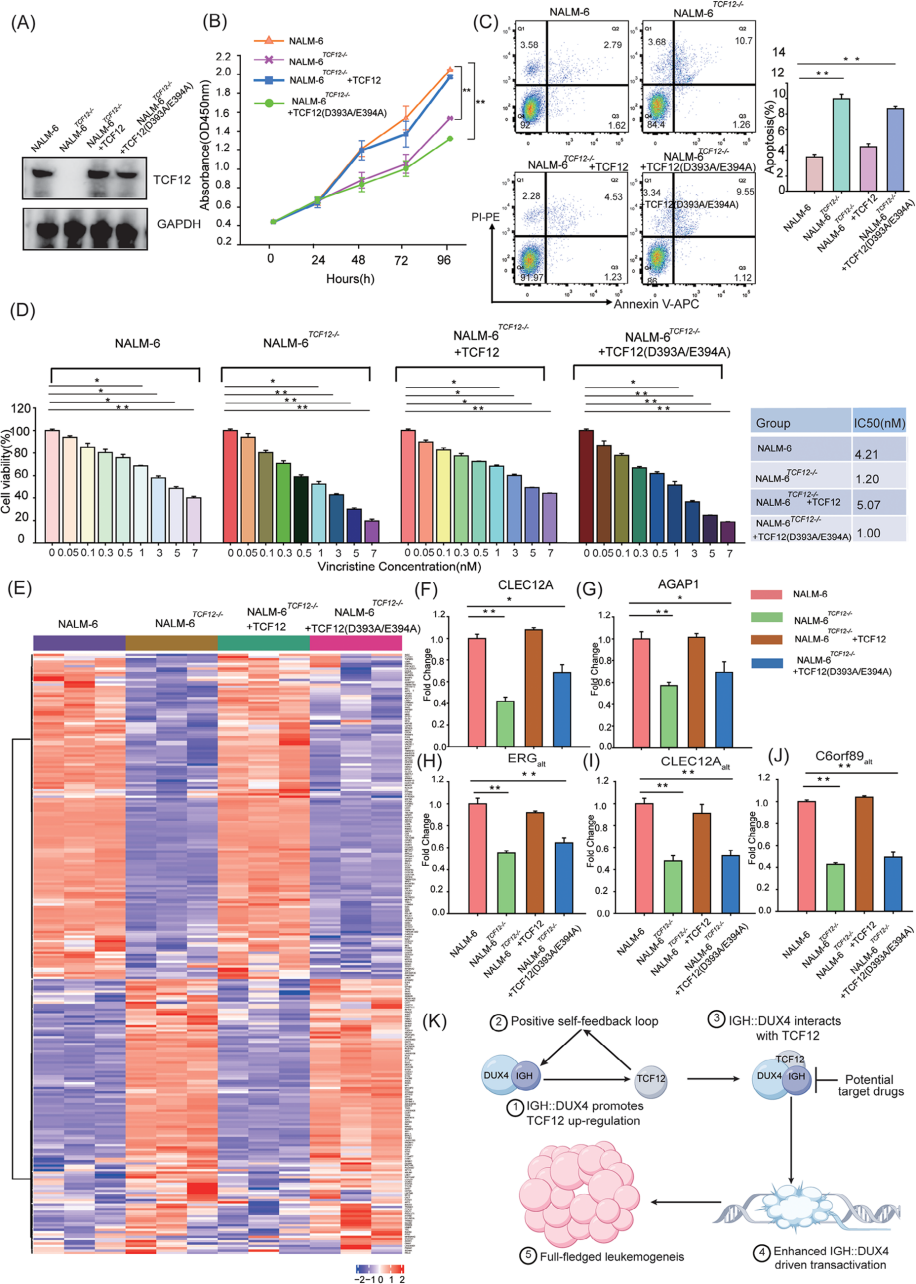
Transcription factor 12 (TCF12) knockout and rescue characterisations in NALM‐6 cells. (A) *TCF12* knockout in NALM‐6 cells. The efficacy of *TCF12* knockout was evaluated by western blot analysis using antibody against TCF12. GAPDH expression was also checked as internal control. (B) Cell proliferation assay. The cell growths of various NALM‐6 and NALM‐6*^TCF12−/−^* cells were analysed for up to 96 h. (C) Flow cytometry analysis using apoptosis markers Annexin V and PI. The statistical summary was shown in the right panel. (D) Evaluation of vincristine sensitivity in *TCF12* knockout and rescue models. The IC50 values for the NALM‐6, NALM‐6*^TCF12−/−^*, NALM‐6*^TCF12−/−^* + TCF12 and NALM‐6*^TCF12−/−^* + TCF12 (D393A/E394A) cells were 4.21, 1.20, 5.07 and 1.00 nM, respectively. (E) Heatmap analysis of differential gene expression in between TCF12 knockout and rescue cells. Different expression genes were annotated on the right side of the heatmap. (F–J) Quantitative real‐time reverse transcriptase‐polymerase chain reaction (qRT‐PCR) characterisation. The expression levels of *AGAP1* (D), *CLEC12A* (E), ERG_alt_ (F), CLEC12A_alt_ (G) and C6orf89_alt_ (H) in various NALM‐6 and NALM‐6*^TCF12−/−^* cells were monitored by qRT‐PCR. (K) Working hypothesis of a positively self‐feedback regulatory loop in between IGH::DUX4–TCF12. In the first step, IGH::DUX4 might bind to the *TCF12* promoter region, upregulating the expression of TCF12. In the second step, TCF12 might in turn enhance IGH::DUX4‐driven deregulation, likely via direct protein–protein interaction and shared DNA‐binding (due to the similarity between DUX4 responsive element [DRE] and TCF12 responsive element [TRE] motifs), to act as an important cofactor to beef up the overall oncogenic activity of IGH::DUX4, resulting in full‐fledged leukaemogenesis. The data were presented as mean ± standard deviation (SD), and the experiments were independently conducted at least three times. ^*^ *p* < .05; ^**^ *p* < .01; ^***^ *p* < .001.

The leukaemia cell line NALM‐6 can stably express IGH::DUX4 endogenously, and hence provide us with a perfect opportunity to monitor the effect of *TCF12* depletion on IGH::DUX4 in leukaemia cells. To gain insights into the transcriptomic changes induced by the interaction between TCF12 and IGH::DUX4, we carried out *TCF12* knockout experiment in NALM‐6 cells using CRISPR/Cas9 technique (Figure 7A). To verify the functional phenotypes in the NALM‐6 and NALM‐6*^TCF12−^* ^/−^ cells, cell proliferation assay was performed. The results showed that the NALM‐6*^TCF12−/−^* cells exhibited a significantly reduced cellular proliferation rate when compared to the WT NALM‐6 cells. In marked contrast, the cell growth rate in NALM‐6*^TCF12−/−^* was recovered with re‐expression of TCF12 proteins (Figure 7B). Supportively, similar results were obtained in cell apoptosis experiment using NALM‐6 TCF12 knockout and rescue models. When compared to the NALM‐6 and the NALM‐6*^TCF12−/−^* + TCF12 cells, both the NALM‐6*^TCF12−/−^* and the NALM‐6*^TCF12−/−^* + TCF12 (D393A/E394A) cells displayed a significant in increase cell death (Figure 7C), reiterating the importance to IGH::DUX4–TCF12 axis in cell survival. To characterise this further, we chose vincristine for drug sensitivity assay. It was well established that the vincristine treatment protocol was successfully used in B‐ALL patients.^36^, ^37^ The NALM‐6*^TCF12−/−^* cells, as well as the NALM‐6*^TCF12−/−^* + TC12 (D393A/E394A) cells, were more sensitive to vincristine treatment, when compared to the NALM‐6 and the NALM‐6*^TCF12−/−^* + TCF12 cells (Figure 7D). The IC50 values of NALM‐6 and NALM‐6*^TCF12−/−^* cells were 4.2 and 1.2 nM, respectively. Upon re‐expression of TCF12 in the leukaemia cells, the IC50 value was recovered to a WT NALM‐6 level (5.1 nM). The resulting NALM‐6*^TCF12−/−^* cells were then subjected to RNA‐seq analysis (Figure 7E). In order to characterise this further, we also conducted the TCF12 rescue experiments, in which the WT TCF12 and mutant (D393A/E394A) were re‐expressed in the leukaemia NALM‐6*^TCF12−/−^* cells. As monitored by western blot analysis, protein expression of TCF12 in NALM‐6*^TCF12−/−^* cells was reduced to null (Figure 7A). The rescue expression could restore WT/mut TCF12 in NALM‐6*^TCF12−/−^* (Figure 7A). Significant transcriptomic differences between control (NALM‐6 cells with endogenous IGH::DUX4), *TCF12* knockout and rescue groups were observed (Figures 7E and S8A). The upregulated and downregulated genes observed in between NALM‐6*^TCF12−/−^* and NALM‐6 cells are listed in Table S5. In additional, the principal component analysis showed that the NALM‐6*^TCF12−/−^* was closed to the NALM‐6*^TCF12−/−^* + TCF12 (D393A/E394A), but not the control and NALM‐6*^TCF12−/−^* + TCF12 cells (Figure S8A). This was also the case in RNA‐seq analysis, in which the gene expression patterns were consistent between the NALM‐6*^TCF12−/−^* group and the NALM‐6*^TCF12−/−^* + TCF12 (D393A/E394A) (Figure 7E). Supportively, the control and NALM‐6*^TCF12−/−^* + TCF12 cells shared similar RNA‐seq profiling. Consistent with the luciferase and qRT‐PCR assay presented in the report (see result above), the expressions of *AGAP1*, *CLEC12A*, *ERG_alt_, CLEC12A_alt_*, *C6orf89_alt_*, etc., which were upregulated in IGH::DUX4 cells and patients, were consistently revised downward in NALM‐6*^TCF12−/−^* (Figures 7E and 7F–J). At the same time, DEGs were enriched and analysed using the signalling pathways in KEGG and Gene Ontology. The results showed that after TCF12 knockout, B‐cell differentiation‐related, cell pro‐apoptosis and immune‐activating signalling pathways were upregulated when compared to the control. This was consistent with the phenotypes of proliferation and apoptosis mentioned above (Figures 7B,C and S8B,C). It is worth noting that we have also revisited the differential gene expression data from various NALM‐6/NALM‐6*^TCF12−/−^* cells and the *DUX4*‐rearranged B‐ALL patients. In this analysis, we have included 50 randomly chosen healthy individuals, the RNA‐seq data of which were obtained from Genotype‐Tissue Expression (GTEx) database (https://gtexportal.org). As expected, the IGH::DUX4‐driven transcriptomic signatures were consistently observed in the NALM‐6 cells, NALM‐6*^TCF12−/−^* + TCF12 cells, and B‐ALL patients (Figure S9). In summary, all these results, together with the shRNA knockdown and AI‐based structural simulation, supported the direct cross‐talk between TCF12 and IGH::DUX4. TCF12, which was one of the IGH::DUX4 target genes, might come back to work as a cofactor to enhance the transcriptional activity of IGH::DUX4 via a positive feedback regulation mechanism (Figure 7K).

## 4 DISCUSSION

B‐ALL accounts for approximately 80% of all cases, but its conventional classification is limited, making diagnosis and treatment challenging. In recent years, numerous international research teams have undertaken comprehensive typing investigations in B‐ALL, leading to the discovery of *IGH::DUX4* and other fusion variants.^3^, ^4^, ^8^ Despite recent studies have demonstrated that *IGH::DUX4* can induce changes at the transcriptomic level and exacerbate disease progression,^9^ it is still not clear whether and how this oncogenic driver might require cofactor for abnormal transactivation. It has been well established that TFs and related regulatory elements are instrumental in the maintenance of stemness, cell identities and cell type‐specific gene expression.^42^ The dysregulation of TFs often results in acute outbreaks of leukaemia and other cancers.^13^ In ALL, the abnormalities of transcriptional cofactors are frequently observed. Mutations in CBP and cofactor EP300 can significantly disrupt the overall CBP acetyltransferase activity, resulting in abnormal downregulation of target genes and leukaemia outbreak.^12^ Furthermore, it has been demonstrated that abnormal copy number variation of transcriptional cofactors such as MLLT3 contributes to the overall activity of the oncogenic transcription complex.^43^ More importantly, understanding these cofactor abnormalities provides therapeutic opportunities for blocking oncogenic TF transactivation. For example, the small molecular antagonist OICR‐9429 can target the C/EBP𝛼‐mutant and resume cell differentiation in AML.^44^ Furthermore, the dysfunctional feedback between TFs and their cofactors is also observed in other cancers beyond leukaemia. In normal cells, ANKRD11, which is frequently associated with p53, can act as a co‐regulator, enhancing the oncogenic suppression function of p53. However, in breast cancer, the expression of ANKRD11 is aberrantly downregulated, resulting in the pathogenic proliferation of tumor cells.^45^ Given the importance of cofactor in oncogenic transactivation as described above, we wanted to ask what partner the IGH::DUX4 might acquire, and what the nature of their interaction and regulatory mechanisms might be.

In this study, we aimed to investigate whether TF TCF12 was involved in IGH::DUX4‐mediated transactivation and the development of acute leukaemia. Through mining the ChIP‐seq and RNA‐seq data, we observed a strong correlation between IGH::DUX4 and TCF12. *TCF12* was the direct target gene of IGH::DUX4. The analysis of the transcriptomic data from 1223 B‐ALL patients showed that *TCF12* gene was significantly upregulated in IGH::DUX4 patients, when compared to other B‐ALL subtypes. Interestingly, it has been demonstrated that other TCF12‐like TFs can bind to GC‐rich sequences by the bi‐partite DNA recognition via the HMG‐box and the C‐clamp domains.^46^ To our surprise, the TCF12 responsive element (TRE) shared ∼30% overlap with IGH::DUX4 DNA binding motif (termed DUX4 responsive element [DRE]). More importantly, multiple TRE sites were consistently observed in genes like *ERG*, *C6of89, CLEC12A* etc. (Figures 3G and S2), which were the subjects of IGH::DUX4‐driven abnormal transactivation and alternative splicing. This observation promoted the molecular, cellular and biophysical characterisation of IGH::DUX4–TCF12 relationship.

Firstly, the available leukaemia cells REH and NALM‐6 were used for *TCF12* overexpression and knockdown analysis. It was clear in both cell lines that, when TCF12 was co‐expressed with IGH::DUX4, the overall abnormal transactivations were significantly upregulated, when compared to IGH::DUX4 alone. Supportively, when TCF12 expression was inhibited in these leukaemia cells, the IGH::DUX4‐driven transcription was significantly reduced. Notably, CLEC12A_alt_ and CLEC12A were identified as candidate genes for further investigation. As expected, the synergistic effect was clearly observed in the presence of TCF12. The expressions of CLEC12A_alt_ and CLEC12A were consistently increased in leukaemia cells that harbored both IGH::DUX4 and TCF12. However, based on the current data, it is not clear whether and how alternative splicing (such as *CLEC12A_alt_*) by IGH::DUX4 might be affected by abnormal IGH::DUX4‐driven transactivation (such as *CLEC12A*).

In order to get more insight into the synergistic effect between TCF12 and IGH::DUX4, we envisaged the TCF12‐DUX4 complex via structural simulation. To our surprise, AI algorithm implemented in AlphaFold program helped to discover a highly positively charged pocket in IGH::DUX4 that might be the binding site for TCF12. The conserved Arg residues (R76/R79/R80) were predicted to directly interact with the D393 and E394 in TCF12 (Figure 6C,D). In line with this simulation/prediction, the structure‐based mutants, R76A/R79A/R80A in IGH::DUX4 and D393A/E394A in TCF12, clearly showed that perturbations of the interface between IGH::DUX4 and TCF12 significantly impaired the interaction and transactivation cooperation between these proteins, as monitored by mammalian two‐hybridisation assay, PLA and luciferase assays. Altogether, this not only provided more evidence for the direct handshake between TCF12 and IGH::DUX4, but also highlighted an important direction for future drug and small molecular compounds design, in which the disruption of oncogenic driver–cofactor complex might represent a valuable strategy for curing IGH::DUX4 leukaemia.

Indeed, based on the results of cell proliferation, apoptosis, and drug sensitivity experiments, it was clear that the loss of *TCF12* could result in impaired leukaemia cell survival and resistance to chemo drug such as vincristine. Supportively, the bulk RNA‐seq results using NALM‐6*^TCF12−/−^* showed that depletion of *TCF12* caused a large scale deregulation of the known IGH::DUX4 target genes. The TCF12 specificity was double checked by rescue experiments, in which the WT TCF12 and mut TCF12 (D393A/E394A) were supplemented to NALM‐6*^TCF12−/−^* cells. Consistent with other results presented in this report, the pathogenic TCF12‐mediated IGH::DUX4 transactivation was observed in WT rescue, but not the mutant D393A/E394A. Supportively, enrichment analysis showed that the expressions of B‐cell differentiation‐related genes, cell pro‐apoptosis genes and immune‐activating genes were upregulated in *TCF12* knockout cells, but not in mutant D393A/E394A. Concerning the expressions of typical IGH::DUX4 target genes such as *AGAP1*, *CLEC12A*, *ERG_alt_*, *CLEC12A_alt_* and *C6orf89_alt_*, these genes were consistently downregulated in NALM‐6*^TCF12−/−^* cells, and upregulated in *TCF12* rescue (but not mutant). More importantly, similar transcriptomic signatures were observed in *DUX4‐*rearranged B‐ALL patients.

Based on these results observed in B‐ALL patients, leukaemia cells and structural simulation, an IGH::DUX4 cofactor TCF12 was proposed. In IGH::DUX4 leukaemia, the oncogenic driver might recruit TCF12 via a positively self‐regulatory feedback loop to beef up its oncogenic transcription activity (Figure 7K). First, IGH::DUX4 recognised its downstream target gene via the DRE, embarking on the aberrant transcription of the *TCF12* gene. Second, the upregulated TCF12 protein might enter the nucleus via its nuclear localisation signal and interact with IGH::DUX4, likely through R76/R79/R80‐D393/E394 hydrogen bonding and shared DNA binding/recognition between DRE and TRE motifs, to complete a positively self‐regulatory loop in the IGH::DUX4–TCF12 transcription complex, triggering the development of full‐fledged leukaemia, which held the potential to function as a pivotal drug therapeutic target for the management of this particular form of leukaemia (Figure 7K).

## AUTHOR CONTRIBUTIONS

*Conceived and designed the experiments*: Guoyu Meng. *Performed the experiments*: Zhihui Li, Minghao Jiang, Junfei Wang, Zhiyi Zhuo, Shiyan Zhang, Yangxia Tan and Hao Zhang. *Analysed the data*: Zhihui Li, Minghao Jiang, Junfei Wang, Zhiyi Zhuo, Shiyan Zhang, Yangxia Tan, Weiguo Hu and Hao Zhang. *Preparation of figures and manuscripts*: Zhihui Li, Minghao Jiang, Junfei Wang, Zhiyi Zhuo, Shiyan Zhang, Yangxia Tan and Hao Zhang. *Wrote the paper*: Zhihui Li, Minghao Jiang, Junfei Wang, Hao Zhang and Guoyu Meng. *Project supervision*: Guoyu Meng. All authors read and approved the final manuscript.

## CONFLICT OF INTEREST STATEMENT

All the authors declare they have no conflicts of interest.

## ETHICS STATEMENT

Not applicable.

## Supporting information

Supporting Information

Supporting Information

Supporting Information

Supporting Information

Supporting Information

Supporting Information

Supporting Information

Supporting Information

Supporting Information

Supporting Information

## Data Availability

The dataset from this study can be made available to the corresponding author upon their reasonable request.
