# Supplementary material for: Transcription factor 12‐mediated self‐feedback regulatory mechanism is required in DUX4 fusion leukaemia
Source: Clin Transl Med. 2023 Dec 19;13(12):e1514. doi: 10.1002/ctm2.1514 (PMC10731121; doi:10.1002/ctm2.1514)
Supplement: Supplementary file 1 — Supporting Information [file CTM2-13-e1514-s005.docx]

Transcription factor 12 mediated self-feedback regulatory mechanism is required in *DUX4* fusion leukemia

Zhihui Li^1,*^, Minghao Jiang^1,*^, Junfei Wang^1,*^, Zhiyi Zhuo^1,*^, Shiyan Zhang^1^, Yangxia Tan^1^, Weiguo Hu^1,2,#^, Hao Zhang^1,3,#^, Guoyu Meng^1,4,#^

^1^Shanghai Institute of Hematology, State Key Laboratory of Medical Genomics, National Research Center for Translational Medicine, Rui-Jin Hospital, Shanghai Jiao Tong University School of Medicine and School of Life Sciences and Biotechnology, Shanghai Jiao Tong University, 197 Ruijin Er Road, Shanghai 200025, China

^2^Department of Geriatrics and Medical Center on Aging, Ruijin Hospital, Shanghai Jiao Tong University School of Medicine, Shanghai 200025, P. R. China

^3^Institute for Translational Brain Research, State Key Laboratory of Medical Neurobiology, MOE Frontiers Center for Brain Science, Jinshan Hospital, Fudan University, Shanghai, China.

^4^State Key Laboratory of Pathogenesis, Prevention and Treatment of High Incidence Diseases in Central Asia，First Affiliated Hospital of Xinjiang Medical University，Xinjiang， P. R. China

^*^ Equal contribution

^#^ Corresponding authors

E-mails: wghu@rjh.com.cn; hao2012590@163.com; guoyumeng@shsmu.edu.cn

Tel: 0086 (0) 2164370045-610730

Fax: 0086 (0) 2164743206

**SUPPLEMENTARY INFORMATION**

**Supplementary Figure 1. Expression of TCF12 in NALM-6, REH and Kasumi-9 leukemia cells that contain endogenous IGH::DUX4, ETV6::RUNX1, MEF2D::HNRNPUL1 fusions.**

**(A)** A boxplot analysis of TCF12 gene expressions between the IGH::DUX4 patients and other subtypes. **(B)** qRT-PCR analysis. Using qRT-PCR assay, the expressions of TCF12 in various leukemia cells that contained endogenous IGH::DUX4, ETV6::RUNX1, and MEF2D::HNRNPUL1 were quantified at the mRNA level. **(C)** Western analysis using antibody against TCF12 in these leukemia cells. The data were presented as mean ± SD, and the experiments were independently conducted at least three times. *, *P* < 0.05; **, *P* < 0.01; ***, *P* < 0.001. Related to Figure 1.

**Supplementary Figure 2. IGH::DUX4-TCF12 cooperation was evident in IGH::DUX4-driven deregulations.**

**(A*)*** *C6orf89*. **(B)** *CLEC12A*. **(C)** *PTPRM*. RNA-seq data, ChIP-seq data, and peak data were plotted together. TCF12-responsive-elements (TREs) were highlighted with yellow arrows. Related to Figure 2.

**Supplementary Figure 3. Molecular functions/signaling pathways attributed to IGH::DUX4 target genes.**

**(A)** Enrichment analysis of IGH::DUX4 target genes in various molecular signaling pathways and human diseases. Significant results from different resources/databases were merged and annotated in each molecular function/signaling term. **(B)** Semantic similarity analysis of 505 significantly enriched terms. Related to Figure 3.

**Supplementary Figure 4. More enrichment analysis of IGH::DUX4-drive deregulations.**

**(A-B)** The heatmap of IGH::DUX4-mediated deregulations in REH cells. Left panel, the IGH::DUX4 cells expressed distinct patterns of T-cell markers. Right panel, the IGH::DUX4 cells expressed distinct patterns of B-cell markers.. Control, empty vector. The experiments were repeated at least three times. **(C-D)** Enrichment analyses of significant up- (left) and down-regulated (right) genes. Significant results from different resources were merged and indicated at the beginning of each term. Related to Figure 3.

**Supplementary Figure 5.** **GSEA analysis of normalized counts in RNA-seq profiling.**

**(A)** The bar plot of the normalized enrichment score (NES), with $-\lg FDR+ {10}^{-100}$ embedded in each bar. Activated pathways were colored in brown, and suppressed ones were colored in blue. **(B)** GSEA plots of different alternative splicing and immune pathways. Related to Figure 3.

**Supplementary Figure 6. The weight matrices of TCF12 responsive element (TRE) in *Mus musculus* and *Homo sapiens*.** Related to Figure 3.

**Supplementary Figure 7. Supporting evidences of synergistic cooperation between IGH::DUX4 and TCF12.**

**(A-B)** Luciferase assay. A double luciferase assay was performed to monitor the enhanced IGH::DUX4 transcription by TCF12, in the context of ERG_alt_ (A) and AGAP1 (B) biogenesis. The control (empty vector, orange), TCF12 alone (red), IGH::DUX4 alone (green), IGH::DUX4+TCF12 (blue), IGH::DUX4 (R76A/R79A/R80A) + TCF12 (yellow) and IGH::DUX4 + TCF12 (D393A/E394A) (pink) groups were subjected to luciferase characterization. The data were presented as mean ± SD, and the experiments were independently conducted at least three times. *, *P* < 0.05; **, *P* < 0.01; ***, *P* < 0.001. Related to Figure 6.

**Supplementary Figure 8. Enrichment analysis and comparison between various NALM-6 and NALM-6*^TCF12-/-^* cells.**

**(A)** PCA analysis of the NALM-6, NALM-6*^TCF12-/-^*, NALM-6*^TCF12-/-^* + TCF12, and NALM-6*^TCF12-/-^* + TCF12 (D393A/E394A) cells. **(B, C)** Analysis of upregulated and downregulated genes based on GO and KEGG pathways. The comparisons include : NALM-6*^TCF12-/-^* cells versus NALM-6 cells , NALM-6*^TCF12-/-^* *+* (D393A/E394A) versus NALM-6 cells , NALM-6*^TCF12-/-^* *+* (D393A/E394A), versus*.*NALM-6*^TCF12-/-^* + TCF12), and *.*NALM-6*^TCF12-/-^* + TCF12 versus NALM-6*^TCF12-/-^*. The data were presented as mean ± SD, and the experiments were independently conducted at least three times. *, *P* < 0.05; **, *P* < 0.01; ***, *P* < 0.001. Related to Figure 7.

**Supplementary Figure 9. Comparative transcriptional analysis between DUX4-rearranged patients and various NALM-6/NALM-6*^TCF12-/-^* cells.**

**Supplementary Table 1. Details of shRNA sequences used in this report.**

**Supplementary Table 2. Details of the gRNA sequences used to obtain NALM-6*^TCF12-/-^* cells.**

**Supplementary Table 3. Details of qRT-PCR primers used in this report.**

**Supplementary Table 4. Different genes directly bound and regulated by IGH::DUX4 in REH cells.**

**Supplementary Table 5. Differential gene expression in NALM-6*^TCF12-/-^* cells.**

**Supplementary Table 1.** **Details of shRNA sequences used in this report.**

|  | Forward sequence (5’-3’) | Reverse sequence (5’-3’) |
| --- | --- | --- |
| Scramble | CCGGGGTTCTCCGAACGTGTCACGTCTCGAGACGTGACACGTTCGGAGAACCTTTTTT | AATTCAAAAA GGTTCTCCGAACGTGTCACGTCTCGAGACGTGACACGTTCGGAGAACC |
| *shTCF12-1* | CCGGCTTACGCGTGCGGGATATTAACTCGAGTTAATATCCCGCACGCGTAAGTTTTTT | AATTCAAAAA CTTACGCGTGCGGGATATTAACTCGAGTTAATATCCCGCACGCGTAAG |
| *shTCF12-2* | AATTCAAAAA CCATCCCATAATGCACCAATTCTCGAGAATTGGTGCATTATGGG | AATTCAAAAA CTCGAATGGAGGATCGTTTAGCTCGAGCTAAACGATCCTCCATTCGAG |
| *shTCF12-3* | AATTCAAAAA CTCGAATGGAGGATCGTTTAGCTCGAGCTAAACGATCCTCCATTCGAG | AATTCAAAAA GCTGTGATTATGGTGAACATACTCGAGTATGTTCACCATAATCACAGC |

**Supplementary Table 2.** **Details of the gRNA sequences used to obtain NALM-6*^TCF12-/-^* cells.**

| GENE | Sequence |  |
| --- | --- | --- |
| gRNA1 | gAGTCGATTAGGAGCCCATGA | |
| gRNA2 | gTTCAAGTGATCACTGTAATG | |
| gRNA3 | gTTTGAGTTCATGAAAGGTGT | |

**Supplementary Table 3.** **Details of qRT-PCR primers used in this report.**

| GENE | Forward Primer (5’-3’) | Reverse Primer (5’-3’) |
| --- | --- | --- |
| *TCF12* | AGTTATCCATCTCCTAAGCCACC | AAGAATTGTGGGTCCCATCTTG |
| *CLEC12A* | TGCAGACATATGGGTGATTGGT | GGACGCCATACATGAGAGGG |
| *AGAP1* | ATACGGGCTGAATGTGGAGAG | CGGAACAGACGGAGGAATGG |
| *ERG_alt_* | ATGGAAGTTTTCAGACAAACG | CTGGTCTTCAGTTTTGGGC |
| *C6orf89_alt_* | GATGAAGACAGACCCTTTCCAG | TAATCTCTCTGACCCTTGGTTTC |
| *CLEC12A_alt_* | ATGGCTCTATTGAGAATTTACAA | TGGACAAGGCTTACATTTGTGCT |
| *β-actin* | ACTTAGTTGCGTTACACCCTTTC | GACTGCTGTCACCTTCACCGT |

**Supplementary Table 4. Different genes directly bound and regulated by IGH::DUX4 in REH cells.**

| gene symbol | gene id | baseMean | log2FoldChange | lfcSE | stat | p value | adjusted p value | target |
| --- | --- | --- | --- | --- | --- | --- | --- | --- |
| ABCA1 | ENSG00000165029.16 | 1508.239255 | 3.842822199 | 0.380782488 | 10.09190894 | 5.99913E-24 | 2.37712E-21 | UP |
| ABCA9 | ENSG00000154258.17 | 838.3192231 | -2.610858248 | 0.546620476 | -4.776363787 | 1.78493E-06 | 5.18358E-05 | DOWN |
| ABCG1 | ENSG00000160179.18 | 32.82917566 | 2.728484528 | 0.679010579 | 4.018324032 | 5.86136E-05 | 0.001081924 | UP |
| ABHD4 | ENSG00000100439.10 | 2450.237163 | -1.431941908 | 0.412771762 | -3.469088828 | 0.000522227 | 0.006796953 | DOWN |
| ABLIM1 | ENSG00000099204.20 | 255.3906903 | -1.517860929 | 0.50607696 | -2.999268981 | 0.002706283 | 0.026066977 | DOWN |
| ABLIM2 | ENSG00000163995.20 | 41.7867232 | 1.987245026 | 0.506358277 | 3.924582881 | 8.68801E-05 | 0.001501124 | UP |
| ADA | ENSG00000196839.13 | 2757.282261 | -1.026326515 | 0.266178239 | -3.855786699 | 0.000115358 | 0.001913441 | DOWN |
| ADAM19 | ENSG00000135074.15 | 192.277891 | 1.989142567 | 0.331706369 | 5.996696936 | 2.01371E-09 | 1.21306E-07 | UP |
| ADAM8 | ENSG00000151651.16 | 114.503181 | 1.717537908 | 0.498784401 | 3.443447518 | 0.000574348 | 0.007330852 | UP |
| ADAMTS14 | ENSG00000138316.11 | 64.00072366 | 6.24824778 | 0.833487526 | 7.496510251 | 6.55393E-14 | 7.53956E-12 | UP |
| ADAMTS7 | ENSG00000136378.15 | 1995.608579 | 1.444238714 | 0.40860151 | 3.534589763 | 0.000408409 | 0.005588899 | UP |
| ADARB2 | ENSG00000185736.16 | 97.98740346 | 3.361285673 | 0.517741348 | 6.492210225 | 8.45861E-11 | 6.5011E-09 | UP |
| ADCY10P1 | ENSG00000161912.18 | 71.80741994 | 2.157230108 | 0.556095676 | 3.87924273 | 0.000104782 | 0.001760953 | UP |
| ADCY3 | ENSG00000138031.14 | 1378.127417 | 2.407451416 | 0.521690141 | 4.614715183 | 3.93635E-06 | 0.000104448 | UP |
| ADRA1A | ENSG00000120907.18 | 121.1066193 | 10.32059865 | 1.379965634 | 7.478880922 | 7.49582E-14 | 8.45936E-12 | UP |
| AFF1 | ENSG00000172493.20 | 3399.362442 | 1.004794526 | 0.26257648 | 3.826673758 | 0.000129886 | 0.002128682 | UP |
| AFF2 | ENSG00000155966.14 | 1807.901703 | -2.050682023 | 0.42468484 | -4.828714921 | 1.37417E-06 | 4.15302E-05 | DOWN |
| AFF3 | ENSG00000144218.19 | 242.1893565 | 1.539534019 | 0.328204621 | 4.690774959 | 2.72172E-06 | 7.52419E-05 | UP |
| AGAP1 | ENSG00000157985.19 | 706.7752284 | 2.800342464 | 0.343648348 | 8.148860549 | 3.67369E-16 | 5.90141E-14 | UP |
| AHCYL2 | ENSG00000158467.16 | 814.7709111 | 1.002076794 | 0.277743367 | 3.607923404 | 0.000308658 | 0.004395905 | UP |
| AK5 | ENSG00000154027.19 | 76.36996175 | 3.029810033 | 0.698604276 | 4.336947451 | 1.44475E-05 | 0.00032445 | UP |
| AK7 | ENSG00000140057.9 | 786.282575 | -1.280939128 | 0.357157756 | -3.586479941 | 0.000335172 | 0.004728203 | DOWN |
| AKAP6 | ENSG00000151320.11 | 92.37261324 | 3.152584762 | 0.521068579 | 6.05023002 | 1.44639E-09 | 9.0177E-08 | UP |
| AKNAD1 | ENSG00000162641.20 | 9.087845851 | 5.916543347 | 1.550584195 | 3.815686607 | 0.000135805 | 0.002217523 | UP |
| ALCAM | ENSG00000170017.12 | 586.8127247 | 1.537257848 | 0.460786777 | 3.336158772 | 0.000849446 | 0.010165421 | UP |
| ALDH2 | ENSG00000111275.13 | 55.40645987 | 1.905275503 | 0.625304575 | 3.046955963 | 0.002311716 | 0.022874697 | UP |
| ALDH4A1 | ENSG00000159423.17 | 1432.181316 | 1.879362429 | 0.332196961 | 5.657373931 | 1.53707E-08 | 7.67715E-07 | UP |
| ALG1 | ENSG00000033011.12 | 650.3733848 | 1.075476053 | 0.234943729 | 4.577589951 | 4.70364E-06 | 0.000123158 | UP |
| AMOTL1 | ENSG00000166025.18 | 8.129986689 | 4.522666993 | 1.373510688 | 3.292778886 | 0.000992025 | 0.011561301 | UP |
| AMZ1 | ENSG00000174945.13 | 20.09461451 | 3.636214674 | 0.8915089 | 4.078719432 | 4.52844E-05 | 0.000861758 | UP |
| ANK2 | ENSG00000145362.19 | 261.6669244 | 1.088661268 | 0.347366649 | 3.134040851 | 0.001724169 | 0.017999794 | UP |
| ANK3 | ENSG00000151150.22 | 13.22171671 | 2.146390629 | 0.771121235 | 2.783467154 | 0.005378131 | 0.044874801 | UP |
| ANKRD10 | ENSG00000088448.14 | 3804.26784 | 1.297509318 | 0.321370709 | 4.037422457 | 5.40417E-05 | 0.001006014 | UP |
| ANKRD33B | ENSG00000164236.12 | 583.1911967 | -1.358750324 | 0.269394108 | -5.043726952 | 4.56551E-07 | 1.56855E-05 | DOWN |
| ANKRD50 | ENSG00000151458.12 | 1116.25088 | 1.445760333 | 0.380455706 | 3.800075301 | 0.000144652 | 0.002336315 | UP |
| ANKRD53 | ENSG00000144031.12 | 24.14233524 | 4.649975906 | 1.018143512 | 4.567112448 | 4.94489E-06 | 0.000128719 | UP |
| ANKRD7 | ENSG00000106013.15 | 9.012461504 | 4.118356176 | 1.381759096 | 2.980516784 | 0.002877625 | 0.027278536 | UP |
| ANKS1B | ENSG00000185046.18 | 45.85671907 | 4.075372172 | 0.713535589 | 5.711519139 | 1.11972E-08 | 5.8896E-07 | UP |
| ANO2 | ENSG00000047617.15 | 53.76814251 | -2.940180462 | 0.609853678 | -4.821124428 | 1.42751E-06 | 4.28518E-05 | DOWN |
| APCDD1 | ENSG00000154856.13 | 246.2418799 | 2.348902734 | 0.267773244 | 8.771984461 | 1.75545E-18 | 3.91267E-16 | UP |
| APELA | ENSG00000248329.6 | 196.6350237 | 7.869765029 | 0.713492676 | 11.02991705 | 2.74118E-28 | 1.48115E-25 | UP |
| APOL6 | ENSG00000221963.6 | 584.1820907 | -1.618206026 | 0.510817095 | -3.167877585 | 0.001535562 | 0.016405392 | DOWN |
| AQP5 | ENSG00000161798.7 | 39.17620303 | -2.104709651 | 0.628886447 | -3.346724453 | 0.000817724 | 0.009892025 | DOWN |
| ARAP2 | ENSG00000047365.12 | 225.86741 | -1.282899708 | 0.433549137 | -2.959064149 | 0.003085749 | 0.028867777 | DOWN |
| ARHGAP25 | ENSG00000163219.12 | 1911.561844 | -1.205169295 | 0.268424506 | -4.489788629 | 7.12939E-06 | 0.000178045 | DOWN |
| ARHGAP40 | ENSG00000124143.10 | 19.66469705 | 5.361404289 | 1.05646481 | 5.07485364 | 3.87794E-07 | 1.36655E-05 | UP |
| ARHGDIB | ENSG00000111348.9 | 23943.41554 | -1.036054873 | 0.31010536 | -3.340976987 | 0.000834841 | 0.010051355 | DOWN |
| ARHGEF25 | ENSG00000240771.8 | 24.27874415 | 8.002816979 | 1.368233699 | 5.849013211 | 4.94498E-09 | 2.79917E-07 | UP |
| ARHGEF28 | ENSG00000214944.9 | 15.37153443 | 2.245155615 | 0.70474338 | 3.185777515 | 0.001443656 | 0.015705811 | UP |
| ARHGEF3 | ENSG00000163947.11 | 297.2659722 | 1.649129329 | 0.479985822 | 3.435787584 | 0.000590834 | 0.00751438 | UP |
| ARHGEF37 | ENSG00000183111.12 | 29.88422606 | 1.836454982 | 0.603587917 | 3.042564192 | 0.002345718 | 0.023185418 | UP |
| ARMH4 | ENSG00000139971.15 | 84.2573808 | 3.06690798 | 0.624459537 | 4.911299766 | 9.04746E-07 | 2.90153E-05 | UP |
| ARSD | ENSG00000006756.16 | 1004.421622 | 1.070866551 | 0.278667784 | 3.842807137 | 0.000121635 | 0.002004505 | UP |
| ART3 | ENSG00000156219.16 | 68.73968053 | 8.123421254 | 1.157584927 | 7.017559629 | 2.25777E-12 | 2.1414E-10 | UP |
| ARVCF | ENSG00000099889.14 | 591.0338412 | 1.64728491 | 0.435489172 | 3.782608196 | 0.000155194 | 0.00247659 | UP |
| ASIP | ENSG00000101440.9 | 37.53010361 | 5.229940516 | 0.964762642 | 5.420960852 | 5.92795E-08 | 2.56557E-06 | UP |
| ASPH | ENSG00000198363.18 | 682.9188441 | 1.248249225 | 0.390492195 | 3.196604803 | 0.001390553 | 0.015211936 | UP |
| ATP10A | ENSG00000206190.11 | 91.12092566 | -2.930512375 | 0.593124508 | -4.940804735 | 7.78008E-07 | 2.53614E-05 | DOWN |
| ATP2C1 | ENSG00000017260.19 | 1501.638815 | 1.021624567 | 0.342100802 | 2.986326141 | 0.002823513 | 0.026875412 | UP |
| ATP6V1B1 | ENSG00000116039.13 | 8.856623761 | 3.464660892 | 1.159608928 | 2.987783906 | 0.002810082 | 0.02678281 | UP |
| ATP8A1 | ENSG00000124406.16 | 3810.795226 | 1.294108146 | 0.464495811 | 2.786049123 | 0.005335479 | 0.044604333 | UP |
| ATP8A2 | ENSG00000132932.17 | 45.15274395 | 5.045769604 | 0.736693939 | 6.849207438 | 7.42603E-12 | 6.62067E-10 | UP |
| ATP9B | ENSG00000166377.20 | 628.9804775 | 1.302087995 | 0.321546707 | 4.049452119 | 5.13377E-05 | 0.000963581 | UP |
| AUNIP | ENSG00000127423.10 | 212.5719648 | -1.376780826 | 0.406634794 | -3.385791988 | 0.000709732 | 0.008727741 | DOWN |
| AURKB | ENSG00000178999.13 | 1671.960901 | -1.054120553 | 0.377974322 | -2.788868161 | 0.005289259 | 0.044299098 | DOWN |
| B3GNT2 | ENSG00000170340.11 | 2638.614531 | -1.613939758 | 0.449886253 | -3.587439597 | 0.000333941 | 0.004714571 | DOWN |
| BAALC | ENSG00000164929.17 | 680.7392456 | -2.250217777 | 0.44582701 | -5.047289029 | 4.48123E-07 | 1.54625E-05 | DOWN |
| BAIAP3 | ENSG00000007516.13 | 135.8165188 | 2.980847904 | 0.556789094 | 5.353639167 | 8.62027E-08 | 3.63376E-06 | UP |
| BCAS1 | ENSG00000064787.13 | 656.5917886 | 3.327083895 | 0.376080525 | 8.846732745 | 9.01189E-19 | 2.08334E-16 | UP |
| BDKRB2 | ENSG00000168398.6 | 5.834555762 | 5.949144653 | 1.635713315 | 3.637033824 | 0.000275796 | 0.004024316 | UP |
| BHLHE41 | ENSG00000123095.6 | 32.15377538 | 5.82169373 | 1.334729323 | 4.361703628 | 1.29054E-05 | 0.000295778 | UP |
| BIVM | ENSG00000134897.14 | 15.90948773 | 2.943928451 | 0.774970664 | 3.798761153 | 0.000145421 | 0.002344489 | UP |
| BLACE | ENSG00000204960.6 | 324.6361253 | -1.739954503 | 0.248140664 | -7.011968421 | 2.34988E-12 | 2.21697E-10 | DOWN |
| BMERB1 | ENSG00000166780.11 | 10.94878185 | 4.255332268 | 1.329544858 | 3.200593228 | 0.00137145 | 0.015030316 | UP |
| BMF | ENSG00000104081.14 | 1355.858967 | 3.563294054 | 0.326978616 | 10.89763636 | 1.1829E-27 | 6.02638E-25 | UP |
| BMPER | ENSG00000164619.10 | 17.28120476 | 7.514531761 | 1.359933178 | 5.5256625 | 3.28245E-08 | 1.54024E-06 | UP |
| BNC2 | ENSG00000173068.18 | 90.94433453 | 6.614318001 | 0.817202971 | 8.093849675 | 5.7808E-16 | 8.96326E-14 | UP |
| BRCA2 | ENSG00000139618.15 | 671.6562605 | -1.440969811 | 0.446889964 | -3.224439859 | 0.001262194 | 0.014066359 | DOWN |
| BRI3BP | ENSG00000184992.12 | 1228.441587 | -1.625583709 | 0.479749547 | -3.388400717 | 0.000703015 | 0.008651108 | DOWN |
| BRINP2 | ENSG00000198797.7 | 33.71550351 | -3.264968418 | 0.912130332 | -3.579497693 | 0.000344255 | 0.004833398 | DOWN |
| BRINP3 | ENSG00000162670.11 | 5.483506314 | 4.861581733 | 1.724931987 | 2.818419375 | 0.004826073 | 0.041115006 | UP |
| BTG2 | ENSG00000159388.6 | 1604.67867 | 1.22778225 | 0.318947569 | 3.849479878 | 0.000118369 | 0.001956104 | UP |
| C12orf75 | ENSG00000235162.8 | 618.9001226 | -1.173180898 | 0.21496621 | -5.457513056 | 4.8285E-08 | 2.16869E-06 | DOWN |
| C13orf46 | ENSG00000283199.3 | 38.98703107 | 1.749608546 | 0.464447637 | 3.767073845 | 0.000165172 | 0.002615617 | UP |
| C20orf194 | ENSG00000088854.12 | 413.6817749 | 1.165395701 | 0.340148764 | 3.426135342 | 0.000612235 | 0.007742388 | UP |
| C20orf197 | ENSG00000176659.9 | 313.3025663 | 2.089441946 | 0.432233453 | 4.834058839 | 1.33777E-06 | 4.06368E-05 | UP |
| C2CD6 | ENSG00000155754.15 | 110.9685949 | 8.177149635 | 0.82608194 | 9.898714931 | 4.21658E-23 | 1.50372E-20 | UP |
| C2orf88 | ENSG00000187699.10 | 45.78713828 | 3.125881276 | 0.636304364 | 4.912556718 | 8.98964E-07 | 2.89154E-05 | UP |
| CABLES1 | ENSG00000134508.12 | 371.6023039 | 1.256246654 | 0.32914414 | 3.816706731 | 0.000135245 | 0.002212431 | UP |
| CACNA1A | ENSG00000141837.20 | 1471.744461 | -1.17516787 | 0.253966197 | -4.627260958 | 3.70534E-06 | 9.98034E-05 | DOWN |
| CACNA2D1 | ENSG00000153956.16 | 141.8161995 | 1.852281709 | 0.361814343 | 5.119425877 | 3.06467E-07 | 1.1062E-05 | UP |
| CADPS | ENSG00000163618.18 | 15.78657373 | 2.8244019 | 0.746738028 | 3.78231963 | 0.000155374 | 0.00247659 | UP |
| CALCOCO1 | ENSG00000012822.16 | 1976.524795 | 1.341190897 | 0.344931333 | 3.88828375 | 0.000100956 | 0.001703064 | UP |
| CALN1 | ENSG00000183166.11 | 557.5794314 | 4.05314308 | 0.514288755 | 7.881064949 | 3.24603E-15 | 4.55747E-13 | UP |
| CALU | ENSG00000128595.17 | 4631.838119 | 1.298293654 | 0.313930689 | 4.135606037 | 3.54019E-05 | 0.000694446 | UP |
| CAMK4 | ENSG00000152495.11 | 168.5395478 | 2.301006269 | 0.494452693 | 4.653642913 | 3.26121E-06 | 8.89154E-05 | UP |
| CAP2 | ENSG00000112186.12 | 563.2294363 | 1.878021015 | 0.293144058 | 6.406478189 | 1.48919E-10 | 1.10641E-08 | UP |
| CAPG | ENSG00000042493.16 | 850.491104 | -1.583900883 | 0.331865457 | -4.772719938 | 1.81754E-06 | 5.26114E-05 | DOWN |
| CARD19 | ENSG00000165233.18 | 268.660427 | 1.204475687 | 0.400205494 | 3.009643058 | 0.002615549 | 0.025346657 | UP |
| CASZ1 | ENSG00000130940.15 | 103.6433989 | -1.405402061 | 0.387858052 | -3.6234959 | 0.000290648 | 0.004192994 | DOWN |
| CAVIN2 | ENSG00000168497.5 | 44.43250599 | 7.847283495 | 1.318782328 | 5.950400855 | 2.67487E-09 | 1.56893E-07 | UP |
| CBFA2T3 | ENSG00000129993.15 | 3934.836866 | -1.195639391 | 0.430388668 | -2.778045708 | 0.005468692 | 0.045460259 | DOWN |
| CBLN3 | ENSG00000139899.11 | 44.18222592 | 1.743318056 | 0.620394945 | 2.810013315 | 0.004953945 | 0.042063711 | UP |
| CBR3-AS1 | ENSG00000236830.7 | 209.5730267 | -1.157243004 | 0.345522651 | -3.349253657 | 0.000810296 | 0.009815478 | DOWN |
| CCBE1 | ENSG00000183287.14 | 21.57735018 | 2.358830578 | 0.736900812 | 3.201015034 | 0.001369444 | 0.015026802 | UP |
| CCDC138 | ENSG00000163006.12 | 251.8105329 | -1.392778044 | 0.314585833 | -4.42733873 | 9.54029E-06 | 0.000226515 | DOWN |
| CCDC26 | ENSG00000229140.10 | 178.672991 | 1.064954049 | 0.358981998 | 2.96659458 | 0.003011177 | 0.028303797 | UP |
| CCDC26 | ENSG00000229140.10 | 178.672991 | 1.064954049 | 0.358981998 | 2.96659458 | 0.003011177 | 0.028303797 | UP |
| CCDC26 | ENSG00000229140.10 | 178.672991 | 1.064954049 | 0.358981998 | 2.96659458 | 0.003011177 | 0.028303797 | UP |
| CCDC3 | ENSG00000151468.11 | 115.9126835 | 5.477740157 | 0.58389235 | 9.381421351 | 6.50893E-21 | 1.87195E-18 | UP |
| CCDC68 | ENSG00000166510.14 | 87.9302977 | 5.673503142 | 0.611009858 | 9.285452704 | 1.61021E-20 | 4.41718E-18 | UP |
| CCDC74B | ENSG00000152076.18 | 148.6731777 | -1.353589236 | 0.431719536 | -3.135343954 | 0.001716527 | 0.017951553 | DOWN |
| CCL25 | ENSG00000131142.13 | 29.61691111 | -1.522921315 | 0.528111438 | -2.883712047 | 0.003930179 | 0.034847981 | DOWN |
| CCN2 | ENSG00000118523.6 | 96.44186471 | -2.811588365 | 0.419537924 | -6.701631019 | 2.06106E-11 | 1.71732E-09 | DOWN |
| CCND3 | ENSG00000112576.12 | 8588.928499 | -1.645081624 | 0.288267092 | -5.706796478 | 1.15122E-08 | 6.00218E-07 | DOWN |
| CCNJ | ENSG00000107443.16 | 1779.29254 | 2.163956831 | 0.470089973 | 4.603282251 | 4.15884E-06 | 0.000109861 | UP |
| CCNJL | ENSG00000135083.15 | 315.2151341 | 1.150889139 | 0.350829953 | 3.280475711 | 0.001036322 | 0.011898683 | UP |
| CCR4 | ENSG00000183813.7 | 21.80360872 | 4.650448205 | 1.010910364 | 4.600257718 | 4.21969E-06 | 0.000111304 | UP |
| CCR7 | ENSG00000126353.3 | 153.2455849 | -2.491463357 | 0.361151081 | -6.898673409 | 5.24904E-12 | 4.77529E-10 | DOWN |
| CD180 | ENSG00000134061.5 | 201.4572084 | 1.951568134 | 0.460428342 | 4.238592536 | 2.24925E-05 | 0.000471841 | UP |
| CD34 | ENSG00000174059.17 | 106.0820652 | 6.061979705 | 0.700620949 | 8.652295811 | 5.04736E-18 | 1.04651E-15 | UP |
| CD37 | ENSG00000104894.12 | 416.5515867 | -1.253117497 | 0.398055523 | -3.148097251 | 0.00164337 | 0.017348796 | DOWN |
| CD74 | ENSG00000019582.15 | 48939.80065 | -1.879025482 | 0.358063942 | -5.247737234 | 1.53979E-07 | 6.06091E-06 | DOWN |
| CD79A | ENSG00000105369.9 | 13944.71429 | -1.500426214 | 0.343020304 | -4.374161517 | 1.219E-05 | 0.000282286 | DOWN |
| CD9 | ENSG00000010278.14 | 1433.446468 | 3.316898098 | 0.399856522 | 8.295220703 | 1.08384E-16 | 1.85826E-14 | UP |
| CD93 | ENSG00000125810.10 | 373.7304815 | -1.563555216 | 0.471394351 | -3.316873044 | 0.00091031 | 0.010806746 | DOWN |
| CDC45 | ENSG00000093009.10 | 949.9415202 | -1.460283842 | 0.270063312 | -5.407190751 | 6.40209E-08 | 2.75739E-06 | DOWN |
| CDCA2 | ENSG00000184661.14 | 517.9593503 | -1.030247154 | 0.351962655 | -2.927149059 | 0.003420849 | 0.031280593 | DOWN |
| CDCA4 | ENSG00000170779.11 | 806.8222329 | -1.003071357 | 0.33531267 | -2.991450809 | 0.002776553 | 0.026598166 | DOWN |
| CDH23 | ENSG00000107736.21 | 133.253094 | -1.01463573 | 0.357911972 | -2.834875078 | 0.004584361 | 0.03947066 | DOWN |
| CDKN1C | ENSG00000129757.13 | 754.0268154 | 1.820212654 | 0.281146836 | 6.474242002 | 9.52891E-11 | 7.26111E-09 | UP |
| CDKN2D | ENSG00000129355.7 | 492.1052834 | -1.100026424 | 0.259004632 | -4.247130315 | 2.16526E-05 | 0.000457992 | DOWN |
| CDON | ENSG00000064309.14 | 118.6588841 | 2.466446249 | 0.44588666 | 5.531554251 | 3.17406E-08 | 1.50127E-06 | UP |
| CDRT4 | ENSG00000239704.11 | 17.84495545 | 2.628964418 | 0.90892723 | 2.892381623 | 0.003823332 | 0.03417235 | UP |
| CELF2 | ENSG00000048740.18 | 4576.733942 | -1.093147361 | 0.367608526 | -2.973672489 | 0.00294259 | 0.027790951 | DOWN |
| CENPA | ENSG00000115163.15 | 515.2276486 | -1.099153952 | 0.372458223 | -2.951079837 | 0.003166651 | 0.029453997 | DOWN |
| CENPK | ENSG00000123219.13 | 431.9635516 | -1.378467565 | 0.382856812 | -3.600478098 | 0.000317633 | 0.004511734 | DOWN |
| CEP55 | ENSG00000138180.16 | 853.2706948 | -1.119835981 | 0.341301743 | -3.281073135 | 0.001034129 | 0.011888819 | DOWN |
| CES5A | ENSG00000159398.16 | 60.99951722 | 5.324641181 | 0.687893821 | 7.740498637 | 9.90277E-15 | 1.32764E-12 | UP |
| CETP | ENSG00000087237.12 | 298.3243592 | 8.445562496 | 0.796196178 | 10.6073889 | 2.75336E-26 | 1.3269E-23 | UP |
| CFAP299 | ENSG00000197826.11 | 87.64185069 | 7.000546939 | 0.913110309 | 7.666704525 | 1.76472E-14 | 2.28019E-12 | UP |
| CFAP36 | ENSG00000163001.11 | 419.1993689 | 1.364609051 | 0.250716994 | 5.442826307 | 5.24418E-08 | 2.30886E-06 | UP |
| CFTR | ENSG00000001626.15 | 26.17947039 | 7.780459988 | 1.334250386 | 5.831334261 | 5.49859E-09 | 3.08319E-07 | UP |
| CHEK1 | ENSG00000149554.13 | 1225.45688 | -1.0420515 | 0.30188985 | -3.451760632 | 0.000556942 | 0.007154773 | DOWN |
| CHRD | ENSG00000090539.15 | 43.81697311 | 2.43928148 | 0.746511318 | 3.267574676 | 0.001084732 | 0.012343245 | UP |
| CHRNA1 | ENSG00000138435.16 | 440.9815553 | 12.1882818 | 1.235829292 | 9.862431552 | 6.05646E-23 | 2.07678E-20 | UP |
| CHRNA7 | ENSG00000175344.18 | 263.1938477 | 7.256515025 | 0.598910131 | 12.11620016 | 8.66859E-34 | 7.02589E-31 | UP |
| CHRNB4 | ENSG00000117971.12 | 81.18134383 | 5.643024577 | 0.565868737 | 9.972320806 | 2.01465E-23 | 7.33128E-21 | UP |
| CHST11 | ENSG00000171310.11 | 1863.175975 | -1.233988273 | 0.294166386 | -4.194864991 | 2.73034E-05 | 0.000556574 | DOWN |
| CHST15 | ENSG00000182022.18 | 1204.46579 | -1.84789253 | 0.254934288 | -7.248505273 | 4.21396E-13 | 4.3433E-11 | DOWN |
| CHST2 | ENSG00000175040.6 | 804.8567636 | 4.423176251 | 0.401744232 | 11.00993094 | 3.42266E-28 | 1.79498E-25 | UP |
| CILP2 | ENSG00000160161.9 | 44.44354375 | 2.566672977 | 0.654766753 | 3.919980613 | 8.85561E-05 | 0.001521237 | UP |
| CISD1 | ENSG00000122873.12 | 515.5297098 | 1.141647329 | 0.240882146 | 4.739443529 | 2.14306E-06 | 6.04634E-05 | UP |
| CITED2 | ENSG00000164442.10 | 3526.821508 | 2.40415468 | 0.316900122 | 7.586474459 | 3.28727E-14 | 4.01475E-12 | UP |
| CKLF | ENSG00000217555.12 | 546.387091 | -1.296029035 | 0.374024898 | -3.465087597 | 0.000530059 | 0.006873802 | DOWN |
| CLDN12 | ENSG00000157224.16 | 281.1809841 | 1.46245404 | 0.473565184 | 3.088178967 | 0.002013872 | 0.020519625 | UP |
| CLDN14 | ENSG00000159261.12 | 6.075946809 | 5.997870252 | 1.691744373 | 3.545376208 | 0.000392053 | 0.005414949 | UP |
| CLEC12A | ENSG00000172322.14 | 39.65187479 | 1.858134787 | 0.591953104 | 3.138989853 | 0.001695313 | 0.017750516 | UP |
| CLEC14A | ENSG00000176435.7 | 13.837125 | 3.900259359 | 1.08249376 | 3.603031725 | 0.000314527 | 0.004472355 | UP |
| CLEC2B | ENSG00000110852.4 | 514.0316041 | -1.017467837 | 0.338160355 | -3.008832414 | 0.002622537 | 0.025400577 | DOWN |
| CLECL1 | ENSG00000184293.7 | 41.27233357 | -1.924721579 | 0.554812566 | -3.469138405 | 0.00052213 | 0.006796953 | DOWN |
| CLIP2 | ENSG00000106665.15 | 3856.10036 | -1.443582574 | 0.267801034 | -5.390504109 | 7.02603E-08 | 3.00434E-06 | DOWN |
| CLUAP1 | ENSG00000103351.13 | 753.2688753 | 2.015776343 | 0.291990681 | 6.903563951 | 5.07138E-12 | 4.63732E-10 | UP |
| CMTM2 | ENSG00000140932.10 | 71.70088608 | -1.688730089 | 0.405418421 | -4.165400493 | 3.10806E-05 | 0.000619477 | DOWN |
| CMTM7 | ENSG00000153551.14 | 1537.071318 | -1.089907926 | 0.302043609 | -3.608445582 | 0.000308037 | 0.004390575 | DOWN |
| CNKSR3 | ENSG00000153721.19 | 363.0456134 | 2.29847622 | 0.303401866 | 7.575682542 | 3.57244E-14 | 4.30407E-12 | UP |
| CNTN2 | ENSG00000184144.12 | 74.85090191 | 5.030424151 | 0.929740124 | 5.410570139 | 6.28244E-08 | 2.7124E-06 | UP |
| CNTNAP2 | ENSG00000174469.23 | 103.3158275 | 7.05335762 | 0.836656136 | 8.430414026 | 3.44444E-17 | 6.53382E-15 | UP |
| COBLL1 | ENSG00000082438.17 | 138.3334103 | 2.445783374 | 0.514357163 | 4.755029286 | 1.98418E-06 | 5.64272E-05 | UP |
| COL17A1 | ENSG00000065618.21 | 19.98108073 | 4.718188493 | 0.967055582 | 4.878921732 | 1.06667E-06 | 3.31935E-05 | UP |
| COL20A1 | ENSG00000101203.16 | 53.84119107 | 5.555433902 | 1.907643149 | 2.912197653 | 0.003588955 | 0.032501091 | UP |
| COL4A2 | ENSG00000134871.19 | 108.4491575 | 2.065578843 | 0.600835454 | 3.43784447 | 0.000586364 | 0.007468188 | UP |
| COL5A1 | ENSG00000130635.15 | 3273.557443 | -1.222364433 | 0.301014325 | -4.060818144 | 4.89011E-05 | 0.00092298 | DOWN |
| COL9A3 | ENSG00000092758.18 | 746.7618881 | 3.359556603 | 0.413523071 | 8.124230155 | 4.50211E-16 | 7.10417E-14 | UP |
| COLEC12 | ENSG00000158270.12 | 544.6612806 | 3.474845616 | 0.344571488 | 10.08454192 | 6.46661E-24 | 2.47359E-21 | UP |
| CPNE2 | ENSG00000140848.17 | 5099.533102 | 1.718259361 | 0.431534005 | 3.981747301 | 6.84105E-05 | 0.001229665 | UP |
| CRHBP | ENSG00000145708.11 | 119.5608742 | 10.30592437 | 1.232591026 | 8.36118725 | 6.20902E-17 | 1.11831E-14 | UP |
| CRIP1 | ENSG00000213145.10 | 948.7837088 | -1.073330026 | 0.378477699 | -2.835913524 | 0.004569482 | 0.039361559 | DOWN |
| CRYBG1 | ENSG00000112297.15 | 1048.978985 | -1.345093022 | 0.273921941 | -4.910497553 | 9.08456E-07 | 2.90797E-05 | DOWN |
| CSGALNACT1 | ENSG00000147408.14 | 54.14753221 | -2.01860815 | 0.592203014 | -3.408642142 | 0.000652871 | 0.008129425 | DOWN |
| CSRNP3 | ENSG00000178662.16 | 193.1291988 | 10.09090482 | 1.253134159 | 8.052533518 | 8.10975E-16 | 1.21517E-13 | UP |
| CST7 | ENSG00000077984.6 | 27.10979522 | -2.238087369 | 0.744555275 | -3.005938502 | 0.002647625 | 0.02561574 | DOWN |
| CTIF | ENSG00000134030.14 | 605.0836126 | 1.319030393 | 0.417228922 | 3.161406897 | 0.00157009 | 0.016733374 | UP |
| CTNS | ENSG00000040531.14 | 987.0112254 | 2.046414586 | 0.375368868 | 5.451742965 | 4.98785E-08 | 2.22346E-06 | UP |
| CYBB | ENSG00000165168.7 | 2559.588812 | -1.610448921 | 0.38944503 | -4.135240653 | 3.54583E-05 | 0.000694768 | DOWN |
| CYP2W1 | ENSG00000073067.14 | 11.15652872 | 3.226709319 | 1.174457794 | 2.747403385 | 0.00600692 | 0.048796992 | UP |
| CYSLTR2 | ENSG00000152207.7 | 443.9258211 | 12.33444931 | 1.051688344 | 11.72823621 | 9.13422E-32 | 6.03231E-29 | UP |
| CYTH2 | ENSG00000105443.15 | 3570.721668 | 1.325171715 | 0.33341242 | 3.974572133 | 7.05059E-05 | 0.00125971 | UP |
| DAAM1 | ENSG00000100592.15 | 507.2500824 | 1.913050236 | 0.481691266 | 3.971527765 | 7.14132E-05 | 0.001274643 | UP |
| DAB1 | ENSG00000173406.15 | 205.3509806 | 1.635401038 | 0.33761221 | 4.844022187 | 1.27237E-06 | 3.88486E-05 | UP |
| DAPK1 | ENSG00000196730.13 | 259.4419625 | 1.223068974 | 0.32747547 | 3.734841495 | 0.000187833 | 0.002930235 | UP |
| DCBLD2 | ENSG00000057019.16 | 396.6322453 | 1.64715169 | 0.332852833 | 4.948588464 | 7.47536E-07 | 2.44575E-05 | UP |
| DCLRE1B | ENSG00000118655.7 | 515.7974167 | -1.075206607 | 0.321971907 | -3.339442304 | 0.000839468 | 0.010092662 | DOWN |
| DDAH1 | ENSG00000153904.20 | 428.5135494 | 10.31462497 | 1.086872895 | 9.490185116 | 2.30624E-21 | 7.2145E-19 | UP |
| DDIT4 | ENSG00000168209.5 | 1469.999525 | -1.585848246 | 0.397052087 | -3.994055943 | 6.49525E-05 | 0.001179713 | DOWN |
| DDIT4L | ENSG00000145358.6 | 3058.620315 | 2.045919398 | 0.48910373 | 4.182996922 | 2.87691E-05 | 0.000580954 | UP |
| DDN | ENSG00000181418.8 | 461.3988597 | 1.31795166 | 0.403842612 | 3.263527971 | 0.001100343 | 0.012496955 | UP |
| DENND2A | ENSG00000146966.13 | 50.66241694 | 3.043472353 | 0.68538851 | 4.440506818 | 8.97473E-06 | 0.000215381 | UP |
| DENND3 | ENSG00000105339.10 | 2349.971067 | -1.73349155 | 0.332794648 | -5.208892517 | 1.89971E-07 | 7.30038E-06 | DOWN |
| DIAPH1 | ENSG00000131504.17 | 10799.09728 | -1.374427671 | 0.234838705 | -5.852645425 | 4.83815E-09 | 2.74742E-07 | DOWN |
| DIP2C | ENSG00000151240.17 | 564.8032693 | 1.484352443 | 0.275602094 | 5.385853286 | 7.21018E-08 | 3.07571E-06 | UP |
| DIPK1C | ENSG00000187773.8 | 313.9657645 | -2.281568136 | 0.376199803 | -6.064777601 | 1.32136E-09 | 8.29621E-08 | DOWN |
| DLC1 | ENSG00000164741.15 | 80.6342931 | 3.244109174 | 0.5332034 | 6.084186956 | 1.17084E-09 | 7.45615E-08 | UP |
| DLGAP1 | ENSG00000170579.17 | 21.65841068 | 7.839648629 | 1.351811292 | 5.799366136 | 6.65661E-09 | 3.69763E-07 | UP |
| DNAH14 | ENSG00000185842.15 | 22.00248742 | 3.942105531 | 0.999863221 | 3.942644804 | 8.0588E-05 | 0.001407408 | UP |
| DNAH2 | ENSG00000183914.14 | 39.48697952 | 3.50407138 | 0.619281193 | 5.658288063 | 1.5289E-08 | 7.65783E-07 | UP |
| DNER | ENSG00000187957.8 | 16.87036798 | 2.470025594 | 0.706602279 | 3.495637742 | 0.00047293 | 0.006288456 | UP |
| DNM3 | ENSG00000197959.14 | 18.60209083 | 2.20567519 | 0.737352196 | 2.991345524 | 0.00277751 | 0.026598166 | UP |
| DOP1A | ENSG00000083097.14 | 915.3678249 | 1.438886352 | 0.261275601 | 5.507159276 | 3.6467E-08 | 1.68894E-06 | UP |
| DPEP1 | ENSG00000015413.9 | 112.6315947 | 1.890966757 | 0.446078912 | 4.239085744 | 2.24432E-05 | 0.00047136 | UP |
| DPF3 | ENSG00000205683.11 | 213.7239983 | -1.214357181 | 0.419050434 | -2.897878349 | 0.003756963 | 0.033714345 | DOWN |
| DTL | ENSG00000143476.18 | 1760.868706 | -1.568594188 | 0.4533226 | -3.460216163 | 0.000539742 | 0.006974013 | DOWN |
| DTNA | ENSG00000134769.21 | 46.93620959 | 5.90858261 | 0.959884852 | 6.155511882 | 7.48354E-10 | 4.92395E-08 | UP |
| DTX3L | ENSG00000163840.10 | 1409.230014 | -1.358763203 | 0.328517363 | -4.136046845 | 3.5334E-05 | 0.000693877 | DOWN |
| DTX4 | ENSG00000110042.8 | 899.5099519 | -2.248787706 | 0.349115212 | -6.441391341 | 1.18383E-10 | 8.90671E-09 | DOWN |
| DUSP6 | ENSG00000139318.8 | 4162.525201 | -1.698912985 | 0.372446164 | -4.561499486 | 5.07896E-06 | 0.000131824 | DOWN |
| DYRK3 | ENSG00000143479.17 | 1382.77347 | -1.642444412 | 0.324656095 | -5.059028417 | 4.21398E-07 | 1.46186E-05 | DOWN |
| E2F7 | ENSG00000165891.16 | 491.0601709 | -1.426296035 | 0.458129905 | -3.113300438 | 0.001850076 | 0.019068614 | DOWN |
| EBF3 | ENSG00000108001.13 | 137.6951544 | 2.850754154 | 0.49487976 | 5.760498575 | 8.38658E-09 | 4.53155E-07 | UP |
| ECE1 | ENSG00000117298.16 | 1251.509457 | 1.824624287 | 0.373767853 | 4.881704702 | 1.05173E-06 | 3.29006E-05 | UP |
| EEF2K | ENSG00000103319.12 | 823.4920872 | -1.224448127 | 0.370385883 | -3.305871478 | 0.000946815 | 0.011140396 | DOWN |
| EFCAB8 | ENSG00000215529.12 | 10.65478528 | 2.766785155 | 1.00331739 | 2.757636997 | 0.005822081 | 0.047708423 | UP |
| EFEMP1 | ENSG00000115380.20 | 898.3702953 | -1.573153843 | 0.512985083 | -3.066665866 | 0.002164607 | 0.021695955 | DOWN |
| EIF2AK3 | ENSG00000172071.14 | 1859.67791 | 1.067882162 | 0.24835987 | 4.299737161 | 1.71001E-05 | 0.000372297 | UP |
| ELFN1 | ENSG00000225968.7 | 710.9085041 | -1.98955683 | 0.384293064 | -5.177186412 | 2.25257E-07 | 8.43815E-06 | DOWN |
| ELK3 | ENSG00000111145.8 | 1032.905895 | -1.036465049 | 0.344325329 | -3.010133039 | 0.002611333 | 0.025319563 | DOWN |
| ELMO1 | ENSG00000155849.15 | 1463.385151 | -1.269300329 | 0.23368038 | -5.431779636 | 5.57948E-08 | 2.43246E-06 | DOWN |
| EMILIN2 | ENSG00000132205.11 | 44.13641651 | 1.510137206 | 0.498583751 | 3.028853637 | 0.002454836 | 0.024050647 | UP |
| ENC1 | ENSG00000171617.14 | 5262.198938 | 5.336131496 | 0.26726069 | 19.96601703 | 1.08791E-88 | 9.69922E-85 | UP |
| ENDOD1 | ENSG00000149218.5 | 1397.14941 | -1.36397005 | 0.433383243 | -3.147260707 | 0.001648079 | 0.017348796 | DOWN |
| EPHA4 | ENSG00000116106.12 | 1120.616761 | 8.168220244 | 0.607482688 | 13.4460132 | 3.24925E-41 | 4.45671E-38 | UP |
| EPHB2 | ENSG00000133216.16 | 378.768279 | -1.171874063 | 0.291076752 | -4.02599677 | 5.67345E-05 | 0.001050501 | DOWN |
| ERAP1 | ENSG00000164307.13 | 2429.601783 | -1.031275972 | 0.357225912 | -2.88690136 | 0.003890562 | 0.03461707 | DOWN |
| ERBB4 | ENSG00000178568.15 | 29.30776565 | 5.693999482 | 1.186706709 | 4.798152263 | 1.60136E-06 | 4.70409E-05 | UP |
| ESCO2 | ENSG00000171320.15 | 358.4398147 | -1.473189803 | 0.51715076 | -2.848666032 | 0.004390294 | 0.038131189 | DOWN |
| ESYT2 | ENSG00000117868.17 | 3699.864807 | -1.517445328 | 0.349163366 | -4.345946553 | 1.38676E-05 | 0.000314198 | DOWN |
| ETS1 | ENSG00000134954.14 | 1274.998409 | -1.349902021 | 0.408259068 | -3.306483865 | 0.000944748 | 0.011134038 | DOWN |
| ETS2 | ENSG00000157557.13 | 5509.830976 | -1.541275566 | 0.290128441 | -5.312390472 | 1.08196E-07 | 4.37472E-06 | DOWN |
| EVI2B | ENSG00000185862.7 | 1900.941126 | -1.161466102 | 0.358754411 | -3.237496367 | 0.001205835 | 0.013552205 | DOWN |
| EYA4 | ENSG00000112319.19 | 38.56082333 | 2.245389588 | 0.550590318 | 4.078149423 | 4.53956E-05 | 0.000862952 | UP |
| F2RL3 | ENSG00000127533.4 | 24.55718532 | 5.10973986 | 0.965570125 | 5.291940716 | 1.21025E-07 | 4.86036E-06 | UP |
| FAM111B | ENSG00000189057.11 | 953.7219049 | -1.59332268 | 0.497811978 | -3.200651555 | 0.001371172 | 0.015030316 | DOWN |
| FAM124A | ENSG00000150510.16 | 77.11349488 | 1.071781287 | 0.339274419 | 3.159039492 | 0.0015829 | 0.016830469 | UP |
| FAM166C | ENSG00000173557.15 | 15.99013977 | 6.765888602 | 1.377319859 | 4.912358273 | 8.99874E-07 | 2.89154E-05 | UP |
| FAM229A | ENSG00000225828.1 | 401.6000942 | 1.656564882 | 0.477031934 | 3.472649864 | 0.000515347 | 0.006731982 | UP |
| FAM86DP | ENSG00000244026.6 | 405.8780077 | 1.403526788 | 0.294863253 | 4.759924389 | 1.93665E-06 | 5.53405E-05 | UP |
| FAM89A | ENSG00000182118.8 | 163.2363785 | 1.160063429 | 0.330011083 | 3.515225671 | 0.00043938 | 0.0059129 | UP |
| FAM9B | ENSG00000177138.17 | 11.97112949 | 4.016544387 | 1.086126593 | 3.69804442 | 0.000217267 | 0.003311184 | UP |
| FAR2 | ENSG00000064763.11 | 602.6306432 | 1.604296015 | 0.298048069 | 5.382675416 | 7.33868E-08 | 3.11562E-06 | UP |
| FARP1 | ENSG00000152767.17 | 1628.391789 | 1.024820099 | 0.369685316 | 2.772141751 | 0.005568878 | 0.046099658 | UP |
| FAT4 | ENSG00000196159.11 | 302.6003072 | 2.570501042 | 0.401059967 | 6.40926858 | 1.46219E-10 | 1.09089E-08 | UP |
| FBXL13 | ENSG00000161040.16 | 17.51626293 | 3.638948988 | 0.996365256 | 3.652223888 | 0.000259979 | 0.003831146 | UP |
| FBXW7 | ENSG00000109670.16 | 1729.384264 | -1.302330056 | 0.368065237 | -3.53831312 | 0.000402692 | 0.005529165 | DOWN |
| FCHO1 | ENSG00000130475.14 | 1335.434309 | -1.277701624 | 0.341519826 | -3.741222398 | 0.000183127 | 0.002869371 | DOWN |
| FGD4 | ENSG00000139132.14 | 84.07477875 | 2.622173599 | 0.522067492 | 5.022671666 | 5.09576E-07 | 1.73733E-05 | UP |
| FHAD1 | ENSG00000142621.19 | 45.98811875 | 7.913554954 | 1.286365488 | 6.151871323 | 7.6574E-10 | 5.01982E-08 | UP |
| FHOD3 | ENSG00000134775.15 | 6.104640837 | 4.645638881 | 1.590060844 | 2.921673658 | 0.003481561 | 0.031705679 | UP |
| FIGNL1 | ENSG00000132436.11 | 679.9710494 | -1.287235112 | 0.405972853 | -3.170741844 | 0.001520502 | 0.016273753 | DOWN |
| FLT1 | ENSG00000102755.12 | 129.9717073 | -1.621364828 | 0.382105227 | -4.243241688 | 2.20314E-05 | 0.000464352 | DOWN |
| FMN1 | ENSG00000248905.9 | 226.1467839 | 2.726727246 | 0.583725713 | 4.671247446 | 2.99376E-06 | 8.19996E-05 | UP |
| FMNL1 | ENSG00000184922.14 | 4027.754809 | -1.743235948 | 0.228756302 | -7.620493648 | 2.52707E-14 | 3.12918E-12 | DOWN |
| FOXO6 | ENSG00000204060.7 | 264.7198539 | 2.496774276 | 0.487755413 | 5.118906335 | 3.07313E-07 | 1.10701E-05 | UP |
| FRAS1 | ENSG00000138759.19 | 731.0840792 | 10.54027477 | 0.889593927 | 11.84841134 | 2.19309E-32 | 1.5642E-29 | UP |
| FREM2 | ENSG00000150893.11 | 6.740429193 | 5.295835943 | 1.647290694 | 3.21487638 | 0.001305007 | 0.014426275 | UP |
| FRMD3 | ENSG00000172159.16 | 47.93556753 | 1.864746397 | 0.516030256 | 3.613637718 | 0.000301931 | 0.00432081 | UP |
| FRMD6 | ENSG00000139926.15 | 98.97658258 | 2.098922403 | 0.506840649 | 4.141187979 | 3.45512E-05 | 0.000680753 | UP |
| FTCD | ENSG00000160282.14 | 51.72726003 | 2.463412448 | 0.588474099 | 4.186101734 | 2.83786E-05 | 0.000574194 | UP |
| FUT5 | ENSG00000130383.7 | 8.151441317 | 6.417484247 | 1.671938576 | 3.838349291 | 0.000123864 | 0.002039356 | UP |
| FZD7 | ENSG00000155760.2 | 163.3467239 | 1.482416243 | 0.311029707 | 4.766156445 | 1.87773E-06 | 5.41414E-05 | UP |
| GAB2 | ENSG00000033327.13 | 2229.939724 | 1.026949276 | 0.348009218 | 2.950925501 | 0.003168233 | 0.029453997 | UP |
| GABARAPL1 | ENSG00000139112.11 | 221.259879 | 1.588499561 | 0.383900288 | 4.137792057 | 3.50664E-05 | 0.000689381 | UP |
| GADL1 | ENSG00000144644.14 | 25.16338632 | 1.688132333 | 0.543988862 | 3.103247973 | 0.001914092 | 0.019637613 | UP |
| GAS6-DT | ENSG00000272695.2 | 15.55604601 | 2.102889091 | 0.714239182 | 2.944236528 | 0.003237525 | 0.029926544 | UP |
| GAS7 | ENSG00000007237.18 | 3545.745865 | -1.913740555 | 0.393814444 | -4.859498137 | 1.17684E-06 | 3.62606E-05 | DOWN |
| GCSAM | ENSG00000174500.13 | 789.3015148 | 1.075647821 | 0.309359637 | 3.477014105 | 0.000507031 | 0.006637939 | UP |
| GDE1 | ENSG00000006007.12 | 1965.611732 | 1.153857853 | 0.31686802 | 3.641446222 | 0.000271111 | 0.003982023 | UP |
| GDPD5 | ENSG00000158555.15 | 446.4461067 | 1.058164008 | 0.295598979 | 3.579728222 | 0.000343952 | 0.004832942 | UP |
| GFOD1 | ENSG00000145990.11 | 820.8980458 | 2.196398231 | 0.216851683 | 10.12857361 | 4.12625E-24 | 1.67216E-21 | UP |
| GFRA1 | ENSG00000151892.14 | 12.91339865 | 3.605676198 | 0.991065654 | 3.638180965 | 0.00027457 | 0.00400994 | UP |
| GINS2 | ENSG00000131153.9 | 978.3261776 | -1.346091303 | 0.364500623 | -3.692973943 | 0.000221647 | 0.003369295 | DOWN |
| GLCCI1 | ENSG00000106415.13 | 1211.821327 | 1.266448927 | 0.338899404 | 3.736946455 | 0.000186269 | 0.002909159 | UP |
| GLDC | ENSG00000178445.9 | 356.3462654 | 1.579292962 | 0.384879378 | 4.103345241 | 4.07219E-05 | 0.000783292 | UP |
| GLYATL1 | ENSG00000166840.13 | 199.3331204 | 11.04247495 | 1.336713704 | 8.260912503 | 1.44563E-16 | 2.45496E-14 | UP |
| GMPR | ENSG00000137198.10 | 24.44736531 | 3.253728088 | 0.671654829 | 4.844345559 | 1.2703E-06 | 3.88486E-05 | UP |
| GNAI1 | ENSG00000127955.17 | 365.6798246 | 1.676072049 | 0.421583302 | 3.975660425 | 7.01842E-05 | 0.00125522 | UP |
| GNG7 | ENSG00000176533.13 | 1637.12415 | -1.150498492 | 0.26077274 | -4.411881744 | 1.02476E-05 | 0.000241381 | DOWN |
| GOLIM4 | ENSG00000173905.9 | 131.5198735 | 1.679961028 | 0.464168911 | 3.619288123 | 0.000295415 | 0.004248014 | UP |
| GPC6 | ENSG00000183098.11 | 24.17273776 | 6.168811121 | 1.171398301 | 5.266194358 | 1.39281E-07 | 5.54356E-06 | UP |
| GPER1 | ENSG00000164850.15 | 155.1959867 | 2.823162179 | 0.527542175 | 5.351538349 | 8.72097E-08 | 3.65891E-06 | UP |
| GPHB5 | ENSG00000179600.4 | 9.511200403 | 6.645954901 | 1.521298005 | 4.368608173 | 1.25041E-05 | 0.000288063 | UP |
| GPHN | ENSG00000171723.15 | 176.7588098 | 6.230154146 | 0.581424895 | 10.71532058 | 8.62579E-27 | 4.2724E-24 | UP |
| GPR155 | ENSG00000163328.13 | 757.5404966 | 4.287120162 | 0.636837834 | 6.73188673 | 1.67477E-11 | 1.4153E-09 | UP |
| GPR158 | ENSG00000151025.11 | 119.328433 | 4.93113629 | 0.642735375 | 7.67210968 | 1.6919E-14 | 2.20207E-12 | UP |
| GPR176 | ENSG00000166073.10 | 548.1584107 | 1.821665535 | 0.299904285 | 6.074156409 | 1.24641E-09 | 7.88112E-08 | UP |
| GPRIN3 | ENSG00000185477.5 | 623.5714039 | 3.247058731 | 0.418501673 | 7.758771201 | 8.57562E-15 | 1.15842E-12 | UP |
| GRAMD1B | ENSG00000023171.18 | 542.8265447 | 1.017884903 | 0.316321358 | 3.217882314 | 0.001291408 | 0.014311432 | UP |
| GRAMD1C | ENSG00000178075.20 | 24.62272701 | 2.551288478 | 0.595569511 | 4.283779528 | 1.83745E-05 | 0.000396653 | UP |
| GRAMD4 | ENSG00000075240.17 | 1237.575014 | -1.271849911 | 0.42817367 | -2.970406637 | 0.002974058 | 0.028043589 | DOWN |
| GRIN2B | ENSG00000273079.5 | 192.8869158 | 10.99141426 | 2.114726446 | 5.197558429 | 2.01923E-07 | 7.67695E-06 | UP |
| GSG1 | ENSG00000111305.19 | 13.7394649 | 2.06545993 | 0.733728035 | 2.815021143 | 0.004877402 | 0.041532457 | UP |
| GSTA4 | ENSG00000170899.11 | 252.8707328 | 1.221651645 | 0.331731145 | 3.682655856 | 0.000230817 | 0.003473158 | UP |
| GVINP1 | ENSG00000254838.5 | 64.6600334 | -2.122550047 | 0.626300726 | -3.389026964 | 0.000701411 | 0.008637336 | DOWN |
| HBEGF | ENSG00000113070.8 | 1919.613639 | 3.92639762 | 0.287091836 | 13.67645165 | 1.40378E-42 | 2.08589E-39 | UP |
| HDAC11 | ENSG00000163517.15 | 219.713065 | 2.017076503 | 0.450843987 | 4.474001116 | 7.67693E-06 | 0.000189858 | UP |
| HERC3 | ENSG00000138641.18 | 475.8292666 | 1.104966303 | 0.266682463 | 4.143378197 | 3.42227E-05 | 0.000675027 | UP |
| HEY1 | ENSG00000164683.17 | 12.11516216 | 6.203154855 | 1.426940467 | 4.347171446 | 1.37904E-05 | 0.000312847 | UP |
| HEY2 | ENSG00000135547.9 | 38.75544824 | 3.886611279 | 0.581891942 | 6.679266365 | 2.40141E-11 | 1.97325E-09 | UP |
| HGF | ENSG00000019991.18 | 15.44552561 | 4.701034625 | 1.140055106 | 4.123515259 | 3.73134E-05 | 0.000725556 | UP |
| HIVEP1 | ENSG00000095951.17 | 643.2208549 | 2.246443794 | 0.3719377 | 6.039838915 | 1.54268E-09 | 9.55123E-08 | UP |
| HJURP | ENSG00000123485.12 | 1476.618592 | -1.114171331 | 0.340144917 | -3.275578366 | 0.001054459 | 0.01208358 | DOWN |
| HMCN2 | ENSG00000148357.16 | 18.91548102 | 5.099132531 | 0.925518161 | 5.509489438 | 3.59876E-08 | 1.67108E-06 | UP |
| HMGB1 | ENSG00000189403.15 | 21324.73378 | -1.189120694 | 0.327030198 | -3.636118936 | 0.000276777 | 0.004035326 | DOWN |
| HMMR | ENSG00000072571.20 | 1308.16771 | -1.077282978 | 0.284306958 | -3.789154465 | 0.000151161 | 0.002423876 | DOWN |
| HMX2 | ENSG00000188816.3 | 905.8783957 | -1.291081438 | 0.235450174 | -5.483459257 | 4.17088E-08 | 1.90695E-06 | DOWN |
| HS3ST3B1 | ENSG00000125430.9 | 696.5041875 | 1.306159862 | 0.252894299 | 5.164845018 | 2.40638E-07 | 8.97661E-06 | UP |
| HS3ST4 | ENSG00000182601.7 | 246.3484043 | 8.936175475 | 0.903450367 | 9.891163701 | 4.54711E-23 | 1.58979E-20 | UP |
| HS6ST2 | ENSG00000171004.18 | 80.55113658 | 1.634201395 | 0.535560512 | 3.051385151 | 0.002277882 | 0.022602621 | UP |
| HTR7 | ENSG00000148680.16 | 82.08860326 | -2.742752701 | 0.610743336 | -4.490843436 | 7.09417E-06 | 0.000177663 | DOWN |
| HTRA3 | ENSG00000170801.10 | 77.75965625 | -2.335758118 | 0.417289207 | -5.597456341 | 2.1752E-08 | 1.06263E-06 | DOWN |
| IER2 | ENSG00000160888.7 | 2300.505527 | -1.280309634 | 0.212321961 | -6.030038671 | 1.6392E-09 | 1.00788E-07 | DOWN |
| IGFBP2 | ENSG00000115457.10 | 4820.603999 | 2.437434813 | 0.428346392 | 5.69033581 | 1.2679E-08 | 6.57206E-07 | UP |
| IGSF9B | ENSG00000080854.15 | 1656.197935 | 13.29426552 | 1.281180264 | 10.3765769 | 3.16936E-25 | 1.37836E-22 | UP |
| IL15RA | ENSG00000134470.21 | 100.2038284 | -1.461883475 | 0.490162764 | -2.982444981 | 0.00285956 | 0.027126606 | DOWN |
| IL18R1 | ENSG00000115604.10 | 49.0745769 | 2.886063322 | 1.023313375 | 2.820312323 | 0.004797693 | 0.040931895 | UP |
| IL18RAP | ENSG00000115607.9 | 42.79893506 | 3.569803735 | 0.726702906 | 4.912328967 | 9.00009E-07 | 2.89154E-05 | UP |
| IL23A | ENSG00000110944.9 | 47.43760253 | 3.369389829 | 0.507543135 | 6.638627536 | 3.16617E-11 | 2.56618E-09 | UP |
| IL27RA | ENSG00000104998.4 | 206.3417051 | -1.221085468 | 0.264490039 | -4.616754087 | 3.89789E-06 | 0.000103736 | DOWN |
| IL7R | ENSG00000168685.15 | 10839.89098 | -1.573748748 | 0.50030232 | -3.145595542 | 0.00165749 | 0.017415859 | DOWN |
| INKA2 | ENSG00000197852.12 | 687.0891413 | 1.085067254 | 0.361708612 | 2.999838037 | 0.002701232 | 0.026035496 | UP |
| INO80C | ENSG00000153391.15 | 594.7937792 | 1.98017264 | 0.377282143 | 5.248519379 | 1.53327E-07 | 6.0486E-06 | UP |
| INPP5D | ENSG00000168918.14 | 2227.2598 | -1.205241196 | 0.298083859 | -4.043295741 | 5.27051E-05 | 0.000984067 | DOWN |
| INSC | ENSG00000188487.11 | 169.479915 | 1.28984157 | 0.464842673 | 2.774791658 | 0.005523708 | 0.045832126 | UP |
| INVS | ENSG00000119509.13 | 708.8507465 | 1.057495645 | 0.297595305 | 3.553468841 | 0.000380186 | 0.005267367 | UP |
| IPCEF1 | ENSG00000074706.13 | 907.9061031 | 4.222733539 | 0.492331814 | 8.577007252 | 9.73735E-18 | 1.9452E-15 | UP |
| IQCJ-SCHIP1 | ENSG00000283154.2 | 51.83622188 | -1.194066757 | 0.651604882 | -1.832501243 | 0.066876777 | 0.275208817 | UP |
| IQCJ-SCHIP1 | ENSG00000283154.2 | 51.83622188 | -1.194066757 | 0.651604882 | -1.832501243 | 0.066876777 | 0.275208817 | UP |
| IQGAP2 | ENSG00000145703.16 | 308.5181663 | 1.645170898 | 0.422206122 | 3.896605973 | 9.75501E-05 | 0.001658166 | UP |
| IRF1 | ENSG00000125347.14 | 1763.824355 | -1.99218893 | 0.26928045 | -7.398193701 | 1.38049E-13 | 1.50095E-11 | DOWN |
| IRF2 | ENSG00000168310.11 | 1077.749729 | -1.068328259 | 0.335710601 | -3.182289318 | 0.001461158 | 0.01583824 | DOWN |
| IRS1 | ENSG00000169047.5 | 152.6021264 | 1.334266817 | 0.46130315 | 2.892386094 | 0.003823278 | 0.03417235 | UP |
| IRX3 | ENSG00000177508.12 | 1010.92012 | 1.177591115 | 0.412949075 | 2.851661827 | 0.004349134 | 0.037847444 | UP |
| ISYNA1 | ENSG00000105655.19 | 1456.63632 | -1.143153178 | 0.411480264 | -2.778148258 | 0.005466967 | 0.045460259 | DOWN |
| ITGA2 | ENSG00000164171.11 | 230.0653444 | 4.885498714 | 0.779553498 | 6.267047391 | 3.67958E-10 | 2.59331E-08 | UP |
| ITGA4 | ENSG00000115232.14 | 6783.470197 | -2.063703986 | 0.441206482 | -4.677410847 | 2.9052E-06 | 7.96963E-05 | DOWN |
| ITGA6 | ENSG00000091409.15 | 2864.652124 | 3.385292124 | 0.51103323 | 6.624407039 | 3.48645E-11 | 2.81298E-09 | UP |
| ITGA9 | ENSG00000144668.12 | 78.67505135 | 5.381402249 | 0.720820876 | 7.465658153 | 8.28843E-14 | 9.29503E-12 | UP |
| ITIH5 | ENSG00000123243.15 | 30.58448307 | 3.907677974 | 0.70292456 | 5.559171203 | 2.71059E-08 | 1.30628E-06 | UP |
| ITPR3 | ENSG00000096433.11 | 1052.923735 | -1.809858879 | 0.357043827 | -5.069010415 | 3.99889E-07 | 1.39539E-05 | DOWN |
| IYD | ENSG00000009765.14 | 18.09916926 | 4.441780794 | 0.980965731 | 4.52796734 | 5.95538E-06 | 0.000152572 | UP |
| IZUMO4 | ENSG00000099840.13 | 124.4116473 | -1.46973789 | 0.398588187 | -3.687359381 | 0.000226593 | 0.003424054 | DOWN |
| JAKMIP1 | ENSG00000152969.20 | 68.68799307 | -1.366264654 | 0.48358243 | -2.825298374 | 0.004723661 | 0.040416311 | DOWN |
| JAZF1 | ENSG00000153814.13 | 195.2966076 | 1.741716488 | 0.422181662 | 4.12551431 | 3.69907E-05 | 0.000720067 | UP |
| JCHAIN | ENSG00000132465.11 | 15.24230733 | 2.821962688 | 0.797896179 | 3.536754234 | 0.000405076 | 0.00555182 | UP |
| JPH2 | ENSG00000149596.6 | 77.85676835 | -1.869065664 | 0.387786955 | -4.819826038 | 1.43683E-06 | 4.30592E-05 | DOWN |
| KANK4 | ENSG00000132854.19 | 8.247129124 | 4.123842664 | 1.331947075 | 3.096100994 | 0.001960836 | 0.02004797 | UP |
| KCNAB1 | ENSG00000169282.17 | 56.80959514 | 1.967021057 | 0.433581995 | 4.536676065 | 5.71478E-06 | 0.00014683 | UP |
| KCNC1 | ENSG00000129159.7 | 11.06174533 | 4.230685794 | 1.224005196 | 3.45642797 | 0.000547385 | 0.007052334 | UP |
| KCNJ12 | ENSG00000184185.10 | 29.38946981 | 6.930016736 | 1.316517384 | 5.263900667 | 1.41031E-07 | 5.58826E-06 | UP |
| KCNJ4 | ENSG00000168135.4 | 69.42228486 | -1.381486021 | 0.428870659 | -3.221218313 | 0.001276469 | 0.014186514 | DOWN |
| KCNN2 | ENSG00000080709.15 | 6.839911244 | 6.173532489 | 1.583148334 | 3.899528778 | 9.63801E-05 | 0.001639841 | UP |
| KCNN4 | ENSG00000104783.14 | 1067.548278 | 1.402461682 | 0.395709859 | 3.544166639 | 0.000393856 | 0.005427241 | UP |
| KCNQ1 | ENSG00000053918.17 | 233.2874834 | -1.444242037 | 0.281515354 | -5.130242512 | 2.89369E-07 | 1.05301E-05 | DOWN |
| KCNT1 | ENSG00000107147.13 | 58.90585334 | 5.259019605 | 0.74395439 | 7.06900809 | 1.56045E-12 | 1.52881E-10 | UP |
| KCTD17 | ENSG00000100379.17 | 2337.424551 | 3.549524801 | 0.460869834 | 7.70179461 | 1.34168E-14 | 1.77212E-12 | UP |
| KDM7A | ENSG00000006459.11 | 506.0808292 | 1.284332313 | 0.426059568 | 3.01444307 | 0.002574515 | 0.024976159 | UP |
| KIAA1217 | ENSG00000120549.18 | 44.61791426 | 1.841852375 | 0.52313291 | 3.520811516 | 0.000430228 | 0.005816074 | UP |
| KIAA1614 | ENSG00000135835.12 | 150.8794403 | 1.537441713 | 0.466951442 | 3.292508758 | 0.000992978 | 0.011564854 | UP |
| KIAA1671 | ENSG00000197077.13 | 846.7210972 | 4.446253487 | 0.348973348 | 12.74095432 | 3.50086E-37 | 3.67199E-34 | UP |
| KIAA2012 | ENSG00000182329.14 | 11.10042949 | 3.836280205 | 1.004399474 | 3.819476519 | 0.000133735 | 0.002189745 | UP |
| KIF1A | ENSG00000130294.16 | 35.37512102 | 7.564361143 | 1.36646963 | 5.535696496 | 3.09994E-08 | 1.474E-06 | UP |
| KIF2C | ENSG00000142945.13 | 1910.90107 | -1.074923408 | 0.293434099 | -3.663253226 | 0.000249032 | 0.003703243 | DOWN |
| KIF4B | ENSG00000226650.6 | 19.08633233 | 3.179589174 | 0.916818339 | 3.468068905 | 0.000524213 | 0.006807098 | UP |
| KLF3 | ENSG00000109787.13 | 397.8641316 | 1.453083083 | 0.292611716 | 4.965908751 | 6.83801E-07 | 2.25794E-05 | UP |
| KLHL13 | ENSG00000003096.14 | 567.4927748 | 3.510615503 | 0.523032212 | 6.712044541 | 1.91916E-11 | 1.6066E-09 | UP |
| KLHL24 | ENSG00000114796.16 | 1470.848852 | 1.496239547 | 0.394370324 | 3.793996289 | 0.000148242 | 0.002385651 | UP |
| KLHL35 | ENSG00000149243.15 | 10.38559228 | 3.01033278 | 1.031450872 | 2.918542085 | 0.003516724 | 0.031945515 | UP |
| KLHL4 | ENSG00000102271.14 | 20.67668802 | 3.418356229 | 0.768964621 | 4.445401175 | 8.7728E-06 | 0.000211962 | UP |
| KMO | ENSG00000117009.12 | 78.15886963 | -2.297327308 | 0.606693584 | -3.786635246 | 0.000152701 | 0.002441983 | DOWN |
| KRT222 | ENSG00000213424.9 | 9.856300235 | 5.612667304 | 1.466040302 | 3.828453623 | 0.000128951 | 0.002117241 | UP |
| LACTB2-AS1 | ENSG00000246366.6 | 37.69970809 | 2.209622581 | 0.530127679 | 4.168095097 | 3.07156E-05 | 0.000614002 | UP |
| LAMB1 | ENSG00000091136.14 | 165.967398 | 2.712642849 | 0.331813994 | 8.175191221 | 2.95397E-16 | 4.83232E-14 | UP |
| LAPTM5 | ENSG00000162511.8 | 39900.05031 | -1.643987584 | 0.243137524 | -6.761554344 | 1.36519E-11 | 1.17032E-09 | DOWN |
| LARGE1 | ENSG00000133424.20 | 615.4622275 | 1.648639501 | 0.254524476 | 6.477331876 | 9.33587E-11 | 7.14455E-09 | UP |
| LAT2 | ENSG00000086730.17 | 3771.203485 | -1.78569592 | 0.262702012 | -6.797420049 | 1.06509E-11 | 9.26422E-10 | DOWN |
| LDB2 | ENSG00000169744.13 | 194.1408902 | 1.269097578 | 0.401454761 | 3.161246796 | 0.001570953 | 0.016733374 | UP |
| LDB3 | ENSG00000122367.19 | 74.15267466 | 7.098519462 | 0.868664643 | 8.171760549 | 3.03921E-16 | 4.92656E-14 | UP |
| LEF1 | ENSG00000138795.10 | 10190.92115 | -1.887615898 | 0.247255105 | -7.634284818 | 2.27077E-14 | 2.85142E-12 | DOWN |
| LEPROT | ENSG00000213625.9 | 374.3050258 | 1.130155616 | 0.389063725 | 2.904808499 | 0.003674779 | 0.033126884 | UP |
| LGALS9 | ENSG00000168961.17 | 3829.978003 | -1.54109317 | 0.344339147 | -4.475509628 | 7.62293E-06 | 0.0001888 | DOWN |
| LGI2 | ENSG00000153012.12 | 92.42812947 | 7.033229794 | 0.873789769 | 8.0491098 | 8.33984E-16 | 1.23923E-13 | UP |
| LGR5 | ENSG00000139292.13 | 519.7014587 | 1.688601638 | 0.487715753 | 3.462265935 | 0.000535648 | 0.006927826 | UP |
| LGR6 | ENSG00000133067.17 | 57.47604915 | 5.875067013 | 0.894540965 | 6.567689177 | 5.11021E-11 | 4.03186E-09 | UP |
| LHFPL2 | ENSG00000145685.14 | 508.6337297 | 1.687483141 | 0.309231476 | 5.457022561 | 4.84185E-08 | 2.16922E-06 | UP |
| LHFPL6 | ENSG00000183722.9 | 338.0776376 | 2.354775502 | 0.324552742 | 7.255447877 | 4.00335E-13 | 4.15022E-11 | UP |
| LIMCH1 | ENSG00000064042.18 | 142.666131 | 5.21689242 | 0.46985215 | 11.1032639 | 1.20945E-28 | 6.73927E-26 | UP |
| LINC00114 | ENSG00000223806.8 | 16.05998212 | -3.005427985 | 0.787845258 | -3.814744016 | 0.000136324 | 0.002218518 | DOWN |
| LINC00261 | ENSG00000259974.3 | 22.68355748 | 7.907029516 | 1.374737139 | 5.751666477 | 8.8368E-09 | 4.7318E-07 | UP |
| LINC00426 | ENSG00000238121.6 | 1355.491721 | -2.73165658 | 0.456318588 | -5.98629259 | 2.14678E-09 | 1.28886E-07 | DOWN |
| LINC00457 | ENSG00000225179.2 | 8.110500556 | 6.422263536 | 1.530054936 | 4.197407154 | 2.69988E-05 | 0.000552083 | UP |
| LINC00461 | ENSG00000245526.11 | 67.09762045 | -1.123249427 | 0.361165098 | -3.110071916 | 0.001870418 | 0.019256018 | DOWN |
| LINC00487 | ENSG00000205837.7 | 26.18441524 | 3.745154381 | 0.819749243 | 4.568658543 | 4.90856E-06 | 0.00012796 | UP |
| LINC00501 | ENSG00000203645.2 | 10.64168105 | 2.977580334 | 0.93429255 | 3.186989272 | 0.001437621 | 0.015659268 | UP |
| LINC00511 | ENSG00000227036.8 | 26.4708417 | 5.01307697 | 1.043808255 | 4.802679943 | 1.56556E-06 | 4.63326E-05 | UP |
| LINC00511 | ENSG00000227036.8 | 26.4708417 | 5.01307697 | 1.043808255 | 4.802679943 | 1.56556E-06 | 4.63326E-05 | UP |
| LINC00598 | ENSG00000215483.11 | 96.73858407 | 3.229702491 | 0.405854097 | 7.957792013 | 1.75136E-15 | 2.49829E-13 | UP |
| LINC00598 | ENSG00000215483.11 | 96.73858407 | 3.229702491 | 0.405854097 | 7.957792013 | 1.75136E-15 | 2.49829E-13 | UP |
| LINC00929 | ENSG00000259150.6 | 10.15555873 | 4.755827477 | 1.455147376 | 3.268278907 | 0.001082037 | 0.012328305 | UP |
| LINC00971 | ENSG00000242641.6 | 6.249631198 | 4.687992667 | 1.565954638 | 2.99369634 | 0.002756201 | 0.026450927 | UP |
| LINC01091 | ENSG00000249464.6 | 13.20082443 | 3.117792907 | 0.870813345 | 3.580322836 | 0.00034317 | 0.004829569 | UP |
| LINC01095 | ENSG00000248809.6 | 30.79319325 | 8.346406641 | 1.338953129 | 6.2335316 | 4.56035E-10 | 3.1396E-08 | UP |
| LINC01354 | ENSG00000231768.2 | 9.854882158 | -2.639958824 | 0.95497779 | -2.76441908 | 0.005702425 | 0.047008756 | DOWN |
| LINC01356 | ENSG00000215866.8 | 16.46487603 | 4.01638804 | 0.925567978 | 4.339376614 | 1.42887E-05 | 0.00032129 | UP |
| LINC01363 | ENSG00000231605.6 | 49.89250483 | 7.739869528 | 1.256975616 | 6.157533549 | 7.38866E-10 | 4.87953E-08 | UP |
| LINC01619 | ENSG00000257242.8 | 153.7350501 | 4.039215513 | 0.510161168 | 7.917528352 | 2.42278E-15 | 3.42862E-13 | UP |
| LINC01625 | ENSG00000238099.3 | 18.07930867 | 5.394217204 | 1.055636004 | 5.109921589 | 3.22293E-07 | 1.15398E-05 | UP |
| LINC01630 | ENSG00000227115.8 | 56.42300966 | 8.563744128 | 1.280561614 | 6.687490891 | 2.27029E-11 | 1.88287E-09 | UP |
| LINC01686 | ENSG00000261504.1 | 7.960343118 | 3.882059921 | 1.223773503 | 3.172204587 | 0.001512864 | 0.016230972 | UP |
| LINC01725 | ENSG00000233008.6 | 6.257442359 | 6.047276847 | 1.640510109 | 3.686217362 | 0.000227612 | 0.003433629 | UP |
| LINC01725 | ENSG00000233008.6 | 6.257442359 | 6.047276847 | 1.640510109 | 3.686217362 | 0.000227612 | 0.003433629 | UP |
| LINC01814 | ENSG00000236008.3 | 15.48768707 | 2.61743132 | 0.728642328 | 3.592203224 | 0.000327894 | 0.00464022 | UP |
| LINC01841 | ENSG00000266913.1 | 23.2613584 | 2.313595845 | 0.816556573 | 2.833356466 | 0.004606199 | 0.039601317 | UP |
| LINC02008 | ENSG00000239440.6 | 8.319503265 | 6.465881164 | 1.562015287 | 4.139448069 | 3.48142E-05 | 0.00068518 | UP |
| LINC02018 | ENSG00000272690.6 | 55.59134442 | 2.079608886 | 0.443124174 | 4.693061238 | 2.69147E-06 | 7.45211E-05 | UP |
| LINC02068 | ENSG00000223387.6 | 5.590802767 | 5.891276995 | 1.668432534 | 3.531025004 | 0.000413953 | 0.005651753 | UP |
| LINC02287 | ENSG00000258499.1 | 25.95527246 | 8.099791308 | 1.321826836 | 6.12772497 | 8.91445E-10 | 5.75919E-08 | UP |
| LINC02393 | ENSG00000256597.3 | 21.57155126 | 5.982723317 | 1.242576027 | 4.814774458 | 1.47366E-06 | 4.39269E-05 | UP |
| LINC02432 | ENSG00000248810.2 | 10.13654729 | 4.53413992 | 1.315376541 | 3.447028116 | 0.00056679 | 0.007265582 | UP |
| LINC02623 | ENSG00000234944.1 | 20.52801629 | 4.530552521 | 0.900317569 | 5.032171618 | 4.84955E-07 | 1.65974E-05 | UP |
| LINC02653 | ENSG00000236373.2 | 6.220205474 | 6.039759636 | 1.637135841 | 3.689223266 | 0.00022494 | 0.003404839 | UP |
| LINC02728 | ENSG00000251323.3 | 47.98996598 | 2.719944271 | 0.499338148 | 5.44709889 | 5.1198E-08 | 2.27092E-06 | UP |
| LINGO4 | ENSG00000213171.2 | 9.918652505 | 3.05306134 | 1.059739351 | 2.880954961 | 0.003964723 | 0.035101775 | UP |
| LMO4 | ENSG00000143013.13 | 2976.579582 | 2.386904153 | 0.268284518 | 8.896913494 | 5.74212E-19 | 1.38362E-16 | UP |
| LMO7 | ENSG00000136153.20 | 383.2091822 | -1.008945927 | 0.366261759 | -2.754712718 | 0.005874369 | 0.048092688 | DOWN |
| LRIG1 | ENSG00000144749.13 | 1651.706876 | 2.271367879 | 0.332145091 | 6.838480961 | 8.00373E-12 | 7.10022E-10 | UP |
| LRRC3B | ENSG00000179796.12 | 27.15623034 | 2.887346124 | 0.645659312 | 4.471934458 | 7.75152E-06 | 0.000191172 | UP |
| LRRC4 | ENSG00000128594.8 | 1123.174415 | 1.781603178 | 0.418505124 | 4.257064189 | 2.07129E-05 | 0.000441256 | UP |
| LRRC53 | ENSG00000162621.6 | 10.19274935 | 6.755025527 | 1.584668793 | 4.262736516 | 2.01939E-05 | 0.000431747 | UP |
| LY86 | ENSG00000112799.9 | 59.10825411 | -2.260293479 | 0.598117784 | -3.779010653 | 0.000157453 | 0.002504495 | DOWN |
| LY9 | ENSG00000122224.18 | 69.92778324 | -1.400511437 | 0.391984025 | -3.572878866 | 0.000353078 | 0.004930099 | DOWN |
| LYPD6B | ENSG00000150556.17 | 12.17866943 | 7.010243614 | 1.576575626 | 4.446500059 | 8.72806E-06 | 0.000211167 | UP |
| LYSMD4 | ENSG00000183060.15 | 262.9734222 | 1.070854133 | 0.300585773 | 3.562557613 | 0.000367259 | 0.005106165 | UP |
| LYZ | ENSG00000090382.6 | 212.1204433 | 2.510579525 | 0.564864462 | 4.44456979 | 8.80679E-06 | 0.000212495 | UP |
| MAGI3 | ENSG00000081026.19 | 361.2856212 | 1.012159073 | 0.315961575 | 3.203424573 | 0.001358036 | 0.014919989 | UP |
| MAML2 | ENSG00000184384.14 | 511.0577554 | 2.589495241 | 0.486776752 | 5.319677303 | 1.03951E-07 | 4.24155E-06 | UP |
| MAP1LC3A | ENSG00000101460.13 | 26.59436717 | 7.049584317 | 1.39002646 | 5.071546852 | 3.94595E-07 | 1.38232E-05 | UP |
| MAP1LC3C | ENSG00000197769.6 | 60.92060649 | 9.332813767 | 1.25886642 | 7.41366488 | 1.22856E-13 | 1.34396E-11 | UP |
| MAP2 | ENSG00000078018.19 | 1562.030322 | 11.68667549 | 0.874556477 | 13.36297402 | 9.95076E-41 | 1.26737E-37 | UP |
| MAP3K14 | ENSG00000006062.17 | 330.1099951 | -1.185445625 | 0.297900317 | -3.979336566 | 6.91078E-05 | 0.0012397 | DOWN |
| MAP4K3 | ENSG00000011566.15 | 497.8076823 | 1.374216023 | 0.293670982 | 4.679440962 | 2.87658E-06 | 7.90329E-05 | UP |
| MAPK10 | ENSG00000109339.23 | 66.88459586 | 1.652116948 | 0.538238522 | 3.06948849 | 0.002144257 | 0.021516174 | UP |
| MAS1 | ENSG00000130368.5 | 39.24581469 | 4.978439425 | 0.708118033 | 7.030522022 | 2.05762E-12 | 1.97255E-10 | UP |
| MBOAT2 | ENSG00000143797.12 | 1017.060769 | 1.293036472 | 0.334819264 | 3.8618939 | 0.000112511 | 0.001871447 | UP |
| MCM3 | ENSG00000112118.20 | 5361.907816 | -1.00878844 | 0.322895284 | -3.124196884 | 0.001782911 | 0.018513929 | DOWN |
| MCU | ENSG00000156026.14 | 1019.184653 | 1.392302549 | 0.351117377 | 3.965347887 | 7.3289E-05 | 0.001299022 | UP |
| ME3 | ENSG00000151376.16 | 587.5492982 | 1.63203362 | 0.377255859 | 4.326065673 | 1.51796E-05 | 0.000338335 | UP |
| MECOM | ENSG00000085276.19 | 159.9265229 | -1.031907596 | 0.337732192 | -3.055401947 | 0.002247591 | 0.022339349 | DOWN |
| MEF2C | ENSG00000081189.15 | 8903.474999 | -1.240478484 | 0.275870941 | -4.496589891 | 6.9052E-06 | 0.000173418 | DOWN |
| MEGF11 | ENSG00000157890.17 | 125.2526203 | 2.14068979 | 0.495022579 | 4.324428588 | 1.52928E-05 | 0.000340007 | UP |
| MEGF6 | ENSG00000162591.16 | 168.9250501 | 2.194059436 | 0.446872555 | 4.909810218 | 9.11646E-07 | 2.90797E-05 | UP |
| MELK | ENSG00000165304.8 | 857.5774146 | -1.260580044 | 0.354480464 | -3.556134037 | 0.000376352 | 0.005219519 | DOWN |
| MERTK | ENSG00000153208.17 | 540.7343738 | 2.462981218 | 0.350779928 | 7.021442841 | 2.19589E-12 | 2.09384E-10 | UP |
| MFSD6 | ENSG00000151690.15 | 615.9673955 | 1.168353191 | 0.341413268 | 3.422108334 | 0.000621376 | 0.00783021 | UP |
| MGAT5 | ENSG00000152127.9 | 3781.432612 | 2.437448084 | 0.250610203 | 9.726052884 | 2.33477E-22 | 7.56933E-20 | UP |
| MILR1 | ENSG00000271605.6 | 1366.911747 | -1.221309829 | 0.255098325 | -4.787604268 | 1.68784E-06 | 4.93375E-05 | DOWN |
| MIPEP | ENSG00000027001.10 | 178.1520989 | -1.008020897 | 0.355237853 | -2.837594276 | 0.004545492 | 0.039211741 | DOWN |
| MIR3681HG | ENSG00000224184.6 | 12.78495342 | 7.065607203 | 1.527669202 | 4.625089773 | 3.74436E-06 | 0.0001004 | UP |
| MIR646HG | ENSG00000228340.6 | 101.7706431 | 2.879745638 | 0.718957676 | 4.005445294 | 6.19008E-05 | 0.001135097 | UP |
| MITF | ENSG00000187098.15 | 11.24576583 | 3.500456631 | 1.254940058 | 2.789341697 | 0.005281531 | 0.044257779 | UP |
| MLLT11 | ENSG00000213190.3 | 3359.619413 | 1.333935441 | 0.315513199 | 4.227827696 | 2.35958E-05 | 0.00049209 | UP |
| MMD | ENSG00000108960.9 | 417.8712255 | 1.145823284 | 0.337765122 | 3.392367088 | 0.000692915 | 0.008568221 | UP |
| MME | ENSG00000196549.10 | 2119.301102 | -1.468064706 | 0.290867006 | -5.047202589 | 4.48326E-07 | 1.54625E-05 | DOWN |
| MNS1 | ENSG00000138587.6 | 336.1731862 | 2.342782934 | 0.440239475 | 5.321610315 | 1.02853E-07 | 4.20635E-06 | UP |
| MPP4 | ENSG00000082126.18 | 48.63863855 | 9.007782752 | 1.271597235 | 7.083833231 | 1.40221E-12 | 1.38137E-10 | UP |
| MPP7 | ENSG00000150054.18 | 142.3124764 | 1.516769384 | 0.404880441 | 3.746215497 | 0.000179522 | 0.00282032 | UP |
| MPZ | ENSG00000158887.17 | 472.4021542 | 2.303899761 | 0.514564445 | 4.477378461 | 7.55652E-06 | 0.000187661 | UP |
| MRC1 | ENSG00000260314.3 | 46.90190551 | 2.735858459 | 0.52809216 | 5.18064585 | 2.21119E-07 | 8.33567E-06 | UP |
| MSGN1 | ENSG00000151379.4 | 42.8259006 | 3.77962431 | 0.586423178 | 6.445216447 | 1.15436E-10 | 8.72174E-09 | UP |
| MSH2 | ENSG00000095002.14 | 1651.784705 | -1.088434886 | 0.334766492 | -3.251325657 | 0.001148682 | 0.013012801 | DOWN |
| MTCL1 | ENSG00000168502.17 | 1576.165725 | 3.655692011 | 0.294046269 | 12.43236998 | 1.74386E-35 | 1.63657E-32 | UP |
| MTUS2 | ENSG00000132938.20 | 71.72241718 | -2.231319191 | 0.538091421 | -4.1467288 | 3.37259E-05 | 0.000666704 | DOWN |
| MVB12B | ENSG00000196814.15 | 1072.754945 | 2.261149158 | 0.444202911 | 5.090351954 | 3.574E-07 | 1.26696E-05 | UP |
| MX1 | ENSG00000157601.14 | 304.2389902 | -1.190368335 | 0.378167705 | -3.14772605 | 0.001645458 | 0.017348796 | DOWN |
| MXD3 | ENSG00000213347.10 | 744.3289149 | -1.296064177 | 0.333052793 | -3.891467667 | 9.96397E-05 | 0.00168565 | DOWN |
| MXI1 | ENSG00000119950.21 | 730.5915509 | 2.724133074 | 0.334704585 | 8.138917697 | 3.98827E-16 | 6.34954E-14 | UP |
| MYB | ENSG00000118513.19 | 6042.715019 | -1.502117582 | 0.366121735 | -4.10278177 | 4.08212E-05 | 0.000784357 | DOWN |
| MYCBP2 | ENSG00000005810.18 | 3509.884667 | -1.267195754 | 0.297722723 | -4.256295054 | 2.07842E-05 | 0.000442248 | DOWN |
| MYLK | ENSG00000065534.18 | 1177.089561 | 2.409606631 | 0.688457996 | 3.500005292 | 0.000465249 | 0.006200189 | UP |
| MYO10 | ENSG00000145555.15 | 1590.057808 | 2.061817643 | 0.362966772 | 5.680458378 | 1.34334E-08 | 6.94294E-07 | UP |
| MYO18A | ENSG00000196535.16 | 8010.792595 | -1.318040953 | 0.254892743 | -5.170963045 | 2.32891E-07 | 8.70581E-06 | DOWN |
| MYO1B | ENSG00000128641.19 | 2192.618876 | 2.219303584 | 0.263435831 | 8.424456056 | 3.6243E-17 | 6.80262E-15 | UP |
| MYO5B | ENSG00000167306.20 | 194.7100608 | 6.516572013 | 0.852780982 | 7.641554104 | 2.14615E-14 | 2.73343E-12 | UP |
| MYOF | ENSG00000138119.17 | 173.5281909 | 5.181626534 | 0.442274947 | 11.71584908 | 1.05727E-31 | 6.7329E-29 | UP |
| NAALADL2 | ENSG00000177694.16 | 63.69544855 | 6.275180844 | 1.118390511 | 5.610903153 | 2.01273E-08 | 9.85963E-07 | UP |
| NAV1 | ENSG00000134369.15 | 1148.242297 | 2.565103425 | 0.243483445 | 10.5350219 | 5.95694E-26 | 2.79522E-23 | UP |
| NBEAL2 | ENSG00000160796.17 | 1449.345207 | -1.222909072 | 0.254093831 | -4.812824724 | 1.48812E-06 | 4.42244E-05 | DOWN |
| NDRG1 | ENSG00000104419.14 | 1128.915552 | 3.757278661 | 0.358552706 | 10.47901355 | 1.07863E-25 | 4.93155E-23 | UP |
| NDRG4 | ENSG00000103034.14 | 29.13914895 | 2.091252706 | 0.676952871 | 3.089214621 | 0.002006864 | 0.020463439 | UP |
| NDUFA4 | ENSG00000189043.10 | 2640.782757 | 1.198312273 | 0.318082356 | 3.767301925 | 0.000165021 | 0.002615552 | UP |
| NEK11 | ENSG00000114670.14 | 24.66737627 | 2.31265789 | 0.719152124 | 3.215811804 | 0.001300761 | 0.014388257 | UP |
| NEK2 | ENSG00000117650.13 | 471.5778356 | -1.108613353 | 0.295105804 | -3.756664012 | 0.000172193 | 0.002712351 | DOWN |
| NEXN | ENSG00000162614.18 | 443.74084 | -1.848542343 | 0.30721967 | -6.017005168 | 1.77673E-09 | 1.08496E-07 | DOWN |
| NFASC | ENSG00000163531.15 | 32.45679668 | 3.401962656 | 0.771031156 | 4.41222463 | 1.02314E-05 | 0.000241317 | UP |
| NFIA | ENSG00000162599.17 | 155.9084637 | 1.16195536 | 0.314824688 | 3.69080128 | 0.000223549 | 0.003395313 | UP |
| NFIA-AS2 | ENSG00000237928.6 | 15.57312473 | 3.762974461 | 0.887939417 | 4.237872979 | 2.25647E-05 | 0.000472534 | UP |
| NLGN3 | ENSG00000196338.12 | 153.9017324 | 1.355220251 | 0.481710619 | 2.813349338 | 0.004902836 | 0.041689299 | UP |
| NOXA1 | ENSG00000188747.8 | 19.37943607 | 2.660537176 | 0.754690659 | 3.52533471 | 0.000422948 | 0.005735047 | UP |
| NPAS2 | ENSG00000170485.17 | 11.60407072 | -2.266666122 | 0.816201072 | -2.7770928 | 0.005484752 | 0.04557251 | DOWN |
| NPDC1 | ENSG00000107281.10 | 104.4402021 | 1.673211044 | 0.469743344 | 3.561968608 | 0.000368084 | 0.005111615 | UP |
| NPR1 | ENSG00000169418.10 | 221.6583058 | -1.679612459 | 0.270630885 | -6.206285203 | 5.42517E-10 | 3.70637E-08 | DOWN |
| NR2F1-AS1 | ENSG00000237187.9 | 103.9208404 | 2.630275403 | 0.770426722 | 3.414050069 | 0.000640048 | 0.008014535 | UP |
| NR3C2 | ENSG00000151623.15 | 492.137727 | 2.594520811 | 0.423236576 | 6.130190444 | 8.77739E-10 | 5.69126E-08 | UP |
| NRCAM | ENSG00000091129.20 | 361.9518847 | 7.364800712 | 0.786675369 | 9.36193124 | 7.82923E-21 | 2.21592E-18 | UP |
| NRN1 | ENSG00000124785.9 | 4604.591367 | -1.567615875 | 0.327776443 | -4.782576376 | 1.73063E-06 | 5.05054E-05 | DOWN |
| NRP2 | ENSG00000118257.16 | 890.9131442 | 5.2200485 | 0.563997552 | 9.25544532 | 2.13339E-20 | 5.76371E-18 | UP |
| NRXN3 | ENSG00000021645.18 | 19.92748892 | 2.075047729 | 0.676465482 | 3.067485013 | 0.002158683 | 0.021648748 | UP |
| NTN1 | ENSG00000065320.9 | 162.5196571 | -2.114038839 | 0.424993846 | -4.974281065 | 6.54902E-07 | 2.17055E-05 | DOWN |
| NUCB2 | ENSG00000070081.17 | 1480.184469 | -1.00996757 | 0.308757338 | -3.271072285 | 0.001071405 | 0.012238454 | DOWN |
| NUDT4 | ENSG00000173598.14 | 3108.271932 | 1.145676701 | 0.375657982 | 3.049786656 | 0.00229004 | 0.022675853 | UP |
| NUF2 | ENSG00000143228.13 | 791.7770617 | -1.446976809 | 0.407275578 | -3.552819977 | 0.000381125 | 0.005272182 | DOWN |
| NUP210 | ENSG00000132182.12 | 47.04032985 | 3.471935419 | 0.587765079 | 5.907012073 | 3.48368E-09 | 2.02337E-07 | UP |
| NXN | ENSG00000167693.17 | 185.9964083 | 2.21367712 | 0.433373826 | 5.10800835 | 3.25572E-07 | 1.16277E-05 | UP |
| OAS2 | ENSG00000111335.12 | 466.2940996 | -1.398459411 | 0.293426221 | -4.765966063 | 1.87951E-06 | 5.41414E-05 | DOWN |
| OPTN | ENSG00000123240.17 | 129.2645111 | 1.877187674 | 0.387609761 | 4.842983488 | 1.27904E-06 | 3.89856E-05 | UP |
| OSBPL10 | ENSG00000144645.14 | 154.3436278 | 2.379002963 | 0.378029409 | 6.293169021 | 3.11049E-10 | 2.20969E-08 | UP |
| OTUD3 | ENSG00000169914.6 | 1129.06536 | 1.404051988 | 0.363113883 | 3.866698725 | 0.000110319 | 0.001843571 | UP |
| P2RX5 | ENSG00000083454.22 | 579.2936543 | -1.513329754 | 0.2779182 | -5.445234439 | 5.17372E-08 | 2.28348E-06 | DOWN |
| PACRG-AS3 | ENSG00000225683.6 | 10.19813179 | 6.144456817 | 1.476941557 | 4.160257249 | 3.17889E-05 | 0.000631916 | UP |
| PALLD | ENSG00000129116.19 | 611.0604315 | 1.594453734 | 0.288247176 | 5.53155023 | 3.17413E-08 | 1.50127E-06 | UP |
| PAM | ENSG00000145730.20 | 1118.068741 | 2.759553067 | 0.364906159 | 7.562363625 | 3.9581E-14 | 4.67397E-12 | UP |
| PANX2 | ENSG00000073150.14 | 134.4433899 | 1.00295648 | 0.353639623 | 2.836097581 | 0.004566849 | 0.039357895 | UP |
| PAPSS1 | ENSG00000138801.9 | 1892.752971 | 1.227736244 | 0.423579952 | 2.898475807 | 0.003749812 | 0.033678613 | UP |
| PARP12 | ENSG00000059378.12 | 617.1199051 | -1.023851844 | 0.220057326 | -4.652659656 | 3.27681E-06 | 8.91633E-05 | DOWN |
| PARVB | ENSG00000188677.14 | 71.43476929 | 4.655775013 | 0.854808877 | 5.446568393 | 5.13509E-08 | 2.27205E-06 | UP |
| PATJ | ENSG00000132849.20 | 30.19262931 | 4.87220366 | 0.84864504 | 5.741156111 | 9.40324E-09 | 5.02003E-07 | UP |
| PAWR | ENSG00000177425.11 | 12.85211519 | 4.841081416 | 1.192992073 | 4.057932593 | 4.95091E-05 | 0.000933188 | UP |
| PAX7 | ENSG00000009709.12 | 94.93236984 | 5.94243604 | 0.654318787 | 9.081866755 | 1.06728E-19 | 2.75808E-17 | UP |
| PBX1 | ENSG00000185630.18 | 29.22083302 | 2.81663937 | 0.671803789 | 4.192651809 | 2.75712E-05 | 0.000560573 | UP |
| PCAT1 | ENSG00000253438.4 | 17.31740071 | 3.637576341 | 0.85897689 | 4.234777889 | 2.28778E-05 | 0.000477809 | UP |
| PCAT6 | ENSG00000228288.7 | 63.56413035 | 2.380564183 | 0.575711531 | 4.134994794 | 3.54963E-05 | 0.000694768 | UP |
| PCBP4 | ENSG00000090097.21 | 2261.22673 | 3.062169429 | 0.442255794 | 6.923978087 | 4.39134E-12 | 4.0571E-10 | UP |
| PCDH10 | ENSG00000138650.9 | 2099.972099 | 2.393996336 | 0.3763493 | 6.361102133 | 2.00311E-10 | 1.45193E-08 | UP |
| PCDHGA12 | ENSG00000253159.3 | 33.94176731 | 1.444142147 | 0.519294516 | 2.780969379 | 0.005419685 | 0.045158131 | UP |
| PCED1B | ENSG00000179715.13 | 128.5217137 | 1.75184817 | 0.548035023 | 3.196598932 | 0.001390581 | 0.015211936 | UP |
| PCSK2 | ENSG00000125851.10 | 7.281932141 | 6.260894276 | 1.624022316 | 3.85517749 | 0.000115646 | 0.00191643 | UP |
| PCSK5 | ENSG00000099139.13 | 128.3355242 | 2.456660876 | 0.497883658 | 4.934206691 | 8.04772E-07 | 2.61382E-05 | UP |
| PDE1C | ENSG00000154678.18 | 35.49649794 | 6.831359913 | 1.179222372 | 5.793105757 | 6.90966E-09 | 3.82628E-07 | UP |
| PDGFRA | ENSG00000134853.12 | 405.6454503 | 11.17773351 | 1.254236683 | 8.911981015 | 5.01287E-19 | 1.22445E-16 | UP |
| PDK3 | ENSG00000067992.15 | 557.408183 | 1.66057563 | 0.341078923 | 4.868596434 | 1.12394E-06 | 3.49145E-05 | UP |
| PDLIM1 | ENSG00000107438.9 | 2891.219704 | 1.149974714 | 0.312296875 | 3.682312592 | 0.000231128 | 0.003474907 | UP |
| PDPN | ENSG00000162493.16 | 19.99860341 | -4.154775691 | 0.842226174 | -4.933087835 | 8.09397E-07 | 2.62407E-05 | DOWN |
| PDZRN4 | ENSG00000165966.16 | 154.6246312 | 3.684866083 | 0.4250801 | 8.668639346 | 4.37315E-18 | 9.17383E-16 | UP |
| PELI2 | ENSG00000139946.10 | 1740.104504 | 1.667807331 | 0.289268798 | 5.765597057 | 8.13693E-09 | 4.41002E-07 | UP |
| PFN2 | ENSG00000070087.14 | 957.2335049 | 1.397846664 | 0.492206674 | 2.839958777 | 0.004511936 | 0.039016653 | UP |
| PGA3 | ENSG00000229859.10 | 2280.602814 | 11.80375415 | 0.854664789 | 13.81097513 | 2.18849E-43 | 3.54754E-40 | UP |
| PGM3 | ENSG00000013375.16 | 470.1836413 | 1.951113736 | 0.50142798 | 3.891114602 | 9.97848E-05 | 0.001686505 | UP |
| PHLPP2 | ENSG00000040199.18 | 267.0059178 | 1.220434842 | 0.31025944 | 3.933594552 | 8.36849E-05 | 0.001455791 | UP |
| PHOX2B | ENSG00000109132.6 | 14.03599616 | 5.323270549 | 1.236609169 | 4.304731587 | 1.67188E-05 | 0.000366546 | UP |
| PID1 | ENSG00000153823.18 | 917.1465921 | 1.40844074 | 0.356607379 | 3.949555795 | 7.82964E-05 | 0.001374117 | UP |
| PIEZO1 | ENSG00000103335.22 | 2530.494892 | -1.020504385 | 0.355492098 | -2.870680928 | 0.004095887 | 0.036012705 | DOWN |
| PIK3C2B | ENSG00000133056.13 | 2070.194725 | -1.383984267 | 0.330628989 | -4.185913256 | 2.84022E-05 | 0.000574194 | DOWN |
| PIK3R5 | ENSG00000141506.13 | 378.0361059 | -1.95041298 | 0.265580373 | -7.343965058 | 2.07357E-13 | 2.24084E-11 | DOWN |
| PIP4K2C | ENSG00000166908.18 | 6.714577882 | 5.090577319 | 1.554061275 | 3.275660619 | 0.001054152 | 0.01208358 | UP |
| PITPNC1 | ENSG00000154217.15 | 1193.275247 | -1.283343642 | 0.364127446 | -3.524435349 | 0.000424386 | 0.005749404 | DOWN |
| PJVK | ENSG00000204311.14 | 81.54178727 | 1.307538677 | 0.374040344 | 3.495715629 | 0.000472792 | 0.006288456 | UP |
| PKD1L1 | ENSG00000158683.8 | 16.5753772 | 3.155831008 | 0.849314739 | 3.715737949 | 0.000202611 | 0.00312523 | UP |
| PKDREJ | ENSG00000130943.7 | 7.347275975 | 4.292087605 | 1.535411139 | 2.795399549 | 0.005183561 | 0.043639317 | UP |
| PKP1 | ENSG00000081277.12 | 27.43389652 | 8.176423302 | 1.384864249 | 5.904133423 | 3.54505E-09 | 2.05233E-07 | UP |
| PLCB4 | ENSG00000101333.16 | 90.23805936 | 5.802661953 | 0.685143441 | 8.469265854 | 2.46944E-17 | 4.7347E-15 | UP |
| PLCH2 | ENSG00000149527.18 | 50.92403984 | 3.088600028 | 0.692248702 | 4.461691323 | 8.13153E-06 | 0.000198621 | UP |
| PLD1 | ENSG00000075651.16 | 617.8929599 | -1.050592334 | 0.348201333 | -3.017197906 | 0.002551232 | 0.024804262 | DOWN |
| PLEKHA5 | ENSG00000052126.14 | 21.96846294 | -2.480925623 | 0.799995573 | -3.10117419 | 0.001927549 | 0.019752942 | DOWN |
| PLEKHA6 | ENSG00000143850.16 | 122.7838107 | 4.115949425 | 0.549467943 | 7.490790821 | 6.84599E-14 | 7.82505E-12 | UP |
| PLPP3 | ENSG00000162407.9 | 28.51841997 | 2.037344912 | 0.691009122 | 2.94836182 | 0.003194629 | 0.029628431 | UP |
| PLTP | ENSG00000100979.15 | 401.5955045 | 5.042468817 | 0.578497571 | 8.716490911 | 2.86957E-18 | 6.23991E-16 | UP |
| PLXNA4 | ENSG00000221866.9 | 66.97204217 | 4.498967163 | 0.745339353 | 6.036132594 | 1.57852E-09 | 9.73928E-08 | UP |
| POGLUT3 | ENSG00000178202.13 | 160.3716426 | -1.204879211 | 0.34417664 | -3.500758248 | 0.000463936 | 0.006191955 | DOWN |
| PPARGC1A | ENSG00000109819.9 | 1280.359425 | 8.129995913 | 0.429045354 | 18.94903614 | 4.49746E-80 | 2.67314E-76 | UP |
| PPFIA4 | ENSG00000143847.15 | 656.3575997 | 2.455024193 | 0.584459352 | 4.200504593 | 2.66321E-05 | 0.000546463 | UP |
| PPM1F | ENSG00000100034.14 | 1967.598701 | -1.128900392 | 0.202337433 | -5.579295816 | 2.41494E-08 | 1.16696E-06 | DOWN |
| PRDM1 | ENSG00000057657.17 | 62.47220039 | 1.926275059 | 0.682978366 | 2.820404209 | 0.004796319 | 0.040931895 | UP |
| PRDM7 | ENSG00000126856.14 | 923.2651595 | -1.293880853 | 0.246955099 | -5.239336445 | 1.61155E-07 | 6.31551E-06 | DOWN |
| PREX1 | ENSG00000124126.14 | 2526.256824 | 1.146658502 | 0.298185373 | 3.845455235 | 0.000120329 | 0.001986651 | UP |
| PRICKLE1 | ENSG00000139174.12 | 132.010452 | 3.975398155 | 0.422070199 | 9.418807963 | 4.56238E-21 | 1.40262E-18 | UP |
| PRIM1 | ENSG00000198056.15 | 818.9653843 | -1.022034982 | 0.299645808 | -3.410810214 | 0.000647702 | 0.008087652 | DOWN |
| PRKCB | ENSG00000166501.14 | 2319.162409 | -1.762519288 | 0.392541042 | -4.490025502 | 7.12146E-06 | 0.000178045 | DOWN |
| PRKCE | ENSG00000171132.14 | 1282.144939 | -1.159613543 | 0.325509848 | -3.56245303 | 0.000367406 | 0.005106165 | DOWN |
| PRKG1 | ENSG00000185532.19 | 20.59834934 | 3.27766409 | 0.952264929 | 3.44196661 | 0.000577502 | 0.00736583 | UP |
| PRKG2 | ENSG00000138669.9 | 15.95301865 | 3.143089832 | 0.951706018 | 3.302584803 | 0.000957981 | 0.011228783 | UP |
| PROS1 | ENSG00000184500.15 | 614.6923231 | 4.769498664 | 0.382581529 | 12.46662033 | 1.1354E-35 | 1.12474E-32 | UP |
| PRR7 | ENSG00000131188.12 | 142.9955223 | -1.097896177 | 0.392425156 | -2.797721193 | 0.005146452 | 0.043429426 | DOWN |
| PRTG | ENSG00000166450.13 | 14.60226152 | 3.8063715 | 0.972042308 | 3.915849615 | 9.00864E-05 | 0.001543065 | UP |
| PRUNE2 | ENSG00000106772.18 | 350.7830713 | 2.585958998 | 0.289983619 | 8.917603728 | 4.76486E-19 | 1.18003E-16 | UP |
| PSMA6 | ENSG00000100902.11 | 9856.522653 | -1.003257283 | 0.29350004 | -3.418252633 | 0.000630246 | 0.007914023 | DOWN |
| PTCH1 | ENSG00000185920.15 | 25.54220443 | 4.8534271 | 0.984366331 | 4.930509047 | 8.20156E-07 | 2.65412E-05 | UP |
| PTCRA | ENSG00000171611.9 | 951.5586939 | 9.387928365 | 0.766482271 | 12.24806982 | 1.72042E-34 | 1.53384E-31 | UP |
| PTK2B | ENSG00000120899.18 | 5401.710138 | -1.527357386 | 0.209338644 | -7.29610816 | 2.96211E-13 | 3.14389E-11 | DOWN |
| PTPN14 | ENSG00000152104.12 | 94.91589216 | 3.125170096 | 0.59098276 | 5.288090123 | 1.236E-07 | 4.95261E-06 | UP |
| PTPRD | ENSG00000153707.17 | 601.3885451 | 1.512796091 | 0.428923884 | 3.52695699 | 0.000420365 | 0.00571475 | UP |
| PTPRE | ENSG00000132334.16 | 1436.100897 | -1.123976907 | 0.286143987 | -3.928011625 | 8.56511E-05 | 0.001482761 | DOWN |
| PTPRG | ENSG00000144724.20 | 644.9235269 | 1.331019661 | 0.304941679 | 4.364833513 | 1.2722E-05 | 0.000292326 | UP |
| PTPRM | ENSG00000173482.16 | 1421.631932 | 4.480235797 | 0.440640457 | 10.16755435 | 2.76771E-24 | 1.1477E-21 | UP |
| PTPRN2 | ENSG00000155093.19 | 121.6366684 | 1.654825057 | 0.364291281 | 4.542587607 | 5.55679E-06 | 0.000143184 | UP |
| PYROXD1 | ENSG00000121350.16 | 280.1933718 | 1.027765137 | 0.351408468 | 2.924702247 | 0.00344786 | 0.031446948 | UP |
| PYY | ENSG00000131096.10 | 26.1578239 | 7.023883626 | 1.320552703 | 5.318896862 | 1.04398E-07 | 4.25006E-06 | UP |
| PYY2 | ENSG00000237575.4 | 32.19678703 | 2.684686778 | 0.876621353 | 3.062538655 | 0.002194681 | 0.021935741 | UP |
| RAB17 | ENSG00000124839.13 | 104.7787141 | 1.467988051 | 0.431819195 | 3.399543302 | 0.000674985 | 0.008358094 | UP |
| RAB20 | ENSG00000139832.5 | 203.3426548 | 2.323172762 | 0.316421916 | 7.342009651 | 2.1041E-13 | 2.26014E-11 | UP |
| RAB27B | ENSG00000041353.10 | 64.79874468 | 4.749598436 | 0.68243498 | 6.959781625 | 3.40801E-12 | 3.16501E-10 | UP |
| RAB3IP | ENSG00000127328.21 | 894.9742791 | 1.658674935 | 0.366833897 | 4.521596685 | 6.13749E-06 | 0.000156948 | UP |
| RAC2 | ENSG00000128340.15 | 5375.764784 | -1.168520537 | 0.27495625 | -4.249841705 | 2.13922E-05 | 0.000453175 | DOWN |
| RAD51 | ENSG00000051180.17 | 480.3867298 | -1.511583008 | 0.409636722 | -3.690057377 | 0.000224203 | 0.003402359 | DOWN |
| RALGAPA2 | ENSG00000188559.15 | 1165.015362 | 1.452153778 | 0.314805671 | 4.612857741 | 3.9717E-06 | 0.000105229 | UP |
| RAP1GAP2 | ENSG00000132359.15 | 2028.177591 | -2.064691287 | 0.272333223 | -7.581488817 | 3.41612E-14 | 4.14373E-12 | DOWN |
| RASGEF1B | ENSG00000138670.17 | 57.95544428 | 2.030101241 | 0.485455345 | 4.181849603 | 2.89147E-05 | 0.000582574 | UP |
| RASSF10 | ENSG00000189431.8 | 45.78066282 | 2.346496546 | 0.456715476 | 5.137764472 | 2.78026E-07 | 1.01588E-05 | UP |
| RASSF2 | ENSG00000101265.16 | 1563.920451 | -1.14098087 | 0.284532888 | -4.01001402 | 6.07151E-05 | 0.001117246 | DOWN |
| RASSF4 | ENSG00000107551.21 | 265.4090921 | 1.396025955 | 0.350887561 | 3.978556414 | 6.9335E-05 | 0.001242462 | UP |
| RBFOX2 | ENSG00000100320.23 | 766.0585534 | 1.274667266 | 0.238987301 | 5.333619248 | 9.62743E-08 | 3.98299E-06 | UP |
| RBL1 | ENSG00000080839.12 | 641.5617321 | -1.126938537 | 0.368129707 | -3.061253996 | 0.00220412 | 0.022015539 | DOWN |
| RBMS3 | ENSG00000144642.22 | 347.3640066 | 4.398543153 | 0.309625475 | 14.2060118 | 8.40794E-46 | 1.49922E-42 | UP |
| RCBTB2 | ENSG00000136161.12 | 823.8809822 | -1.15191461 | 0.411501548 | -2.79929593 | 0.005121418 | 0.043238636 | DOWN |
| RDH14 | ENSG00000240857.2 | 861.9609917 | 2.165993298 | 0.364734595 | 5.938546353 | 2.8756E-09 | 1.68114E-07 | UP |
| RERE | ENSG00000142599.19 | 2640.888476 | 1.079103165 | 0.271908756 | 3.968622349 | 7.22893E-05 | 0.001285136 | UP |
| RET | ENSG00000165731.20 | 7.28699513 | 5.05062013 | 1.580258348 | 3.196072424 | 0.001393121 | 0.015221046 | UP |
| RFC2 | ENSG00000049541.11 | 1460.771848 | -1.312411159 | 0.238784635 | -5.496212758 | 3.88034E-08 | 1.78326E-06 | DOWN |
| RFTN1 | ENSG00000131378.14 | 783.1993984 | -2.295384719 | 0.266349372 | -8.61794681 | 6.81653E-18 | 1.39708E-15 | DOWN |
| RGMB | ENSG00000174136.13 | 1981.33274 | 2.563171309 | 0.212329022 | 12.07169554 | 1.49032E-33 | 1.15538E-30 | UP |
| RGS16 | ENSG00000143333.7 | 292.4640598 | 3.618349294 | 0.44893977 | 8.05976556 | 7.64409E-16 | 1.1551E-13 | UP |
| RGS19 | ENSG00000171700.14 | 1341.450105 | -1.699561943 | 0.392048033 | -4.335086008 | 1.45703E-05 | 0.000326797 | DOWN |
| RGS4 | ENSG00000117152.13 | 47.03307659 | 6.55072797 | 1.061524024 | 6.171059551 | 6.78339E-10 | 4.54716E-08 | UP |
| RGS6 | ENSG00000182732.18 | 9.909928752 | 6.704265628 | 1.491594917 | 4.494695948 | 6.96694E-06 | 0.000174722 | UP |
| RGS8 | ENSG00000135824.12 | 265.7703038 | 9.835999551 | 1.102633859 | 8.920458475 | 4.64362E-19 | 1.1662E-16 | UP |
| RGS9 | ENSG00000108370.17 | 152.4108858 | -1.151030619 | 0.390771754 | -2.945531778 | 0.003224 | 0.029847949 | DOWN |
| RHCE | ENSG00000188672.18 | 47.96333134 | 3.689174895 | 0.722272963 | 5.107729467 | 3.26053E-07 | 1.16277E-05 | UP |
| RHEX | ENSG00000263961.8 | 9.892423542 | -3.182048675 | 1.091353664 | -2.915689735 | 0.003549032 | 0.032155893 | DOWN |
| RHOB | ENSG00000143878.10 | 469.5135855 | 1.508623786 | 0.340298906 | 4.433231367 | 9.28312E-06 | 0.000221589 | UP |
| RHOBTB1 | ENSG00000072422.17 | 1658.039869 | 4.81283219 | 0.551982226 | 8.719179647 | 2.80225E-18 | 6.16875E-16 | UP |
| RHOH | ENSG00000168421.13 | 490.8186704 | 1.165891472 | 0.226984796 | 5.13642981 | 2.80007E-07 | 1.02102E-05 | UP |
| RIMS3 | ENSG00000117016.10 | 6188.239347 | 4.371855996 | 0.297265243 | 14.70691949 | 5.8197E-49 | 1.48244E-45 | UP |
| RMI2 | ENSG00000175643.10 | 370.6116245 | -1.048941341 | 0.266771192 | -3.931988805 | 8.4246E-05 | 0.001464123 | DOWN |
| RNASEH2B | ENSG00000136104.20 | 2367.661285 | -1.314449816 | 0.297985573 | -4.411118973 | 1.02838E-05 | 0.000241594 | DOWN |
| RNF122 | ENSG00000133874.2 | 273.8253296 | 1.345853425 | 0.33210547 | 4.052487978 | 5.06758E-05 | 0.000953165 | UP |
| RNF217 | ENSG00000146373.16 | 167.2726428 | 1.402062788 | 0.364145468 | 3.850282131 | 0.000117982 | 0.001951516 | UP |
| ROR1 | ENSG00000185483.12 | 10.75867047 | 4.445322578 | 1.319566174 | 3.368775788 | 0.000755028 | 0.009202261 | UP |
| ROR1-AS1 | ENSG00000223949.7 | 9.91978297 | 3.187255658 | 0.964823748 | 3.303458962 | 0.000954999 | 0.011210399 | UP |
| ROR2 | ENSG00000169071.15 | 213.4448573 | 6.441786613 | 0.632727117 | 10.1809871 | 2.41103E-24 | 1.0236E-21 | UP |
| RPS6KA2 | ENSG00000071242.12 | 1025.824949 | 3.172881039 | 0.264798621 | 11.98224153 | 4.40256E-33 | 3.27092E-30 | UP |
| RRAGD | ENSG00000025039.15 | 599.4216757 | -1.132472217 | 0.388286588 | -2.916588549 | 0.003538823 | 0.032112339 | DOWN |
| RRM2 | ENSG00000171848.15 | 3616.99903 | -1.489945555 | 0.379084777 | -3.930375595 | 8.48133E-05 | 0.001471114 | DOWN |
| RSAD2 | ENSG00000134321.12 | 88.33946972 | -1.344215922 | 0.486580063 | -2.76257912 | 0.005734666 | 0.047187278 | DOWN |
| RSRP1 | ENSG00000117616.18 | 2637.704134 | 1.11991031 | 0.278816828 | 4.016652505 | 5.90307E-05 | 0.001088496 | UP |
| RUNX2 | ENSG00000124813.23 | 391.7420501 | 5.628074948 | 0.476529674 | 11.81054456 | 3.44324E-32 | 2.3614E-29 | UP |
| S100Z | ENSG00000171643.14 | 19.31897364 | 5.27915364 | 1.231560045 | 4.286558061 | 1.81463E-05 | 0.000393155 | UP |
| S1PR1 | ENSG00000170989.9 | 28.24906558 | 2.080834239 | 0.623326606 | 3.338272773 | 0.000843009 | 0.01010195 | UP |
| SASH1 | ENSG00000111961.18 | 41.92178476 | 5.978438066 | 0.991984926 | 6.026742858 | 1.67297E-09 | 1.02511E-07 | UP |
| SATB2 | ENSG00000119042.17 | 492.0434182 | 2.065668861 | 0.413444993 | 4.996236249 | 5.846E-07 | 1.96309E-05 | UP |
| SCAT8 | ENSG00000236345.1 | 48.48367563 | 6.092421976 | 0.970317916 | 6.278789534 | 3.41219E-10 | 2.4144E-08 | UP |
| SCMH1 | ENSG00000010803.16 | 1182.357304 | -1.039731276 | 0.331892836 | -3.132731902 | 0.001731876 | 0.018059107 | DOWN |
| SCML4 | ENSG00000146285.14 | 12.18698379 | 5.81645836 | 1.563312138 | 3.7205995 | 0.00019875 | 0.00308435 | UP |
| SCN1A-AS1 | ENSG00000236107.10 | 86.47163966 | -1.484289531 | 0.471198068 | -3.150033144 | 0.001632519 | 0.017265394 | DOWN |
| SCN1A-AS1 | ENSG00000236107.10 | 86.47163966 | -1.484289531 | 0.471198068 | -3.150033144 | 0.001632519 | 0.017265394 | DOWN |
| SCN2A | ENSG00000136531.16 | 129.5820156 | 4.878661292 | 0.687313262 | 7.098162605 | 1.26426E-12 | 1.25939E-10 | UP |
| SCN4A | ENSG00000007314.12 | 496.6890402 | -1.347264927 | 0.25892548 | -5.203292179 | 1.95789E-07 | 7.47561E-06 | DOWN |
| SCNN1A | ENSG00000111319.13 | 46.23100316 | 3.998676928 | 0.837259702 | 4.775909932 | 1.78896E-06 | 5.18683E-05 | UP |
| SDCBP | ENSG00000137575.12 | 1592.639464 | 1.161306018 | 0.394536673 | 2.943467864 | 0.003245575 | 0.029985415 | UP |
| SDHAP1 | ENSG00000185485.14 | 526.7791916 | 1.228350537 | 0.339691433 | 3.616077466 | 0.000299101 | 0.004290642 | UP |
| SDK1 | ENSG00000146555.19 | 299.7873809 | -1.410784605 | 0.376363714 | -3.748460739 | 0.000177923 | 0.002797662 | DOWN |
| SEC14L1 | ENSG00000129657.16 | 1637.070505 | 1.585745435 | 0.241620479 | 6.56295958 | 5.27502E-11 | 4.14356E-09 | UP |
| SELPLG | ENSG00000110876.9 | 32.39688449 | 1.704675289 | 0.560499092 | 3.041352454 | 0.00235518 | 0.023240292 | UP |
| SEPTIN9 | ENSG00000184640.18 | 14152.38133 | -1.443644408 | 0.270231402 | -5.342252588 | 9.17986E-08 | 3.8334E-06 | DOWN |
| SERGEF | ENSG00000129158.11 | 291.7792185 | 1.793404829 | 0.301358998 | 5.951057834 | 2.66415E-09 | 1.5678E-07 | UP |
| SERPINB2 | ENSG00000197632.9 | 45.74082729 | 8.918394145 | 1.295866441 | 6.882186205 | 5.89409E-12 | 5.3349E-10 | UP |
| SESN3 | ENSG00000149212.12 | 4058.212536 | 3.065906709 | 0.435167601 | 7.045346895 | 1.85E-12 | 1.79279E-10 | UP |
| SFT2D2 | ENSG00000213064.10 | 1922.273385 | 1.446375762 | 0.364619399 | 3.966809682 | 7.28411E-05 | 0.001292368 | UP |
| SGCD | ENSG00000170624.13 | 13.57022608 | 4.762579955 | 1.199947965 | 3.968988733 | 7.21783E-05 | 0.001284442 | UP |
| SGK1 | ENSG00000118515.11 | 342.2026276 | 1.519181455 | 0.46189563 | 3.289014563 | 0.001005388 | 0.011660111 | UP |
| SGPL1 | ENSG00000166224.17 | 1648.215439 | 2.361434674 | 0.251286885 | 9.39736537 | 5.59464E-21 | 1.66263E-18 | UP |
| SH2D3C | ENSG00000095370.20 | 1770.17933 | -1.455204484 | 0.327436291 | -4.444237016 | 8.82043E-06 | 0.000212537 | DOWN |
| SH3BP5 | ENSG00000131370.16 | 236.815908 | 3.735089888 | 0.580718533 | 6.431842064 | 1.26067E-10 | 9.44494E-09 | UP |
| SH3D21 | ENSG00000214193.11 | 236.0143182 | -1.187406949 | 0.401708726 | -2.955890359 | 0.003117679 | 0.029120657 | DOWN |
| SH3KBP1 | ENSG00000147010.18 | 2232.765343 | -1.302853245 | 0.260096876 | -5.00910763 | 5.4683E-07 | 1.85019E-05 | DOWN |
| SH3PXD2A | ENSG00000107957.16 | 1208.891881 | -1.762531657 | 0.401363539 | -4.391359662 | 1.12644E-05 | 0.000261872 | DOWN |
| SH3PXD2B | ENSG00000174705.13 | 566.1267108 | 1.16168537 | 0.383963799 | 3.02550754 | 0.002482162 | 0.024278351 | UP |
| SH3RF3 | ENSG00000172985.11 | 346.0016275 | -1.684006704 | 0.304910064 | -5.522962018 | 3.33332E-08 | 1.55186E-06 | DOWN |
| SHANK1 | ENSG00000161681.15 | 449.8979411 | -1.153143354 | 0.270649091 | -4.260658508 | 2.03825E-05 | 0.000435259 | DOWN |
| SHB | ENSG00000107338.10 | 281.0004403 | 3.577210657 | 0.442380706 | 8.086271864 | 6.15188E-16 | 9.37557E-14 | UP |
| SHROOM3 | ENSG00000138771.16 | 2077.967851 | 1.035515751 | 0.351225107 | 2.948296491 | 0.003195304 | 0.029628431 | UP |
| SIGLEC15 | ENSG00000197046.12 | 327.9700676 | -1.624628647 | 0.444691985 | -3.653379645 | 0.000258811 | 0.00381709 | DOWN |
| SIPA1L1 | ENSG00000197555.9 | 1108.226448 | 1.018643558 | 0.29069822 | 3.504127264 | 0.000458106 | 0.006127903 | UP |
| SKA2 | ENSG00000182628.13 | 1566.79453 | -1.078471207 | 0.335037021 | -3.218961306 | 0.001286558 | 0.014275435 | DOWN |
| SLC12A7 | ENSG00000113504.21 | 2074.555904 | -1.376737348 | 0.286670088 | -4.802514827 | 1.56685E-06 | 4.63326E-05 | DOWN |
| SLC13A4 | ENSG00000164707.15 | 28.07812548 | 2.13472171 | 0.722643468 | 2.954045537 | 0.003136377 | 0.029249342 | UP |
| SLC15A1 | ENSG00000088386.17 | 20.81589638 | 5.908057015 | 1.209322003 | 4.88542919 | 1.03204E-06 | 3.23414E-05 | UP |
| SLC15A2 | ENSG00000163406.11 | 106.4323784 | -2.784756753 | 0.602428577 | -4.622550885 | 3.7905E-06 | 0.000101484 | DOWN |
| SLC16A14 | ENSG00000163053.11 | 396.632374 | 1.917318425 | 0.338457534 | 5.664871457 | 1.47135E-08 | 7.46755E-07 | UP |
| SLC17A7 | ENSG00000104888.10 | 1207.442194 | -1.541276379 | 0.287652013 | -5.35812826 | 8.40885E-08 | 3.55304E-06 | DOWN |
| SLC22A11 | ENSG00000168065.16 | 34.82030477 | -2.547007641 | 0.60260434 | -4.226666605 | 2.37179E-05 | 0.000494058 | DOWN |
| SLC25A40 | ENSG00000075303.13 | 488.560048 | -1.24912357 | 0.452223404 | -2.762182494 | 0.005741638 | 0.04722285 | DOWN |
| SLC25A48 | ENSG00000145832.14 | 31.27957452 | 7.34636119 | 1.305224155 | 5.628428775 | 1.81859E-08 | 8.9826E-07 | UP |
| SLC26A9 | ENSG00000174502.19 | 19.59406381 | -2.007247582 | 0.664617723 | -3.020153559 | 0.002526466 | 0.024611397 | DOWN |
| SLC29A4 | ENSG00000164638.10 | 813.0784736 | 1.445879958 | 0.417513379 | 3.463074554 | 0.00053404 | 0.006915377 | UP |
| SLC38A1 | ENSG00000111371.16 | 3571.149897 | 1.399680317 | 0.336033122 | 4.165304629 | 3.10937E-05 | 0.000619477 | UP |
| SLC38A6 | ENSG00000139974.15 | 61.73859063 | 1.260387126 | 0.451858745 | 2.789338794 | 0.005281578 | 0.044257779 | UP |
| SLC47A1 | ENSG00000142494.13 | 99.65267115 | 1.393729471 | 0.403280946 | 3.455976498 | 0.000548303 | 0.007059056 | UP |
| SLC4A5 | ENSG00000188687.17 | 123.7146135 | 1.82157174 | 0.367632905 | 4.954865888 | 7.23802E-07 | 2.37245E-05 | UP |
| SLC7A2 | ENSG00000003989.17 | 13.9569443 | 7.202428435 | 1.416767497 | 5.0837053 | 3.70142E-07 | 1.30952E-05 | UP |
| SLC9C1 | ENSG00000172139.15 | 14.77733781 | 4.84997069 | 1.088975426 | 4.453700766 | 8.44027E-06 | 0.000205319 | UP |
| SLCO4A1 | ENSG00000101187.16 | 418.9873444 | -1.059241447 | 0.308169972 | -3.43719876 | 0.000587764 | 0.007480672 | DOWN |
| SLIT2 | ENSG00000145147.20 | 560.2014724 | 1.905800366 | 0.357016754 | 5.338125857 | 9.39123E-08 | 3.9125E-06 | UP |
| SMAD1 | ENSG00000170365.10 | 1163.958258 | 1.871015404 | 0.252190135 | 7.419066594 | 1.17949E-13 | 1.31446E-11 | UP |
| SMAD7 | ENSG00000101665.9 | 165.1225886 | 1.29575632 | 0.298379218 | 4.342649356 | 1.40775E-05 | 0.000317741 | UP |
| SMIM11B | ENSG00000273590.4 | 54.16942629 | -3.913248792 | 0.976448658 | -4.00763395 | 6.13301E-05 | 0.001126238 | DOWN |
| SMIM24 | ENSG00000095932.7 | 455.9163412 | -1.359779397 | 0.285138671 | -4.768835433 | 1.85294E-06 | 5.3549E-05 | DOWN |
| SMIM3 | ENSG00000256235.2 | 1336.012462 | -1.354057074 | 0.252963891 | -5.352768218 | 8.66188E-08 | 3.64269E-06 | DOWN |
| SMOC2 | ENSG00000112562.18 | 39.30514062 | 8.694873765 | 1.36443341 | 6.372516019 | 1.85952E-10 | 1.35335E-08 | UP |
| SNPH | ENSG00000101298.15 | 87.9922656 | 2.332305149 | 0.459970383 | 5.070555049 | 3.96657E-07 | 1.38682E-05 | UP |
| SNX16 | ENSG00000104497.14 | 176.9083013 | 1.604325031 | 0.426472922 | 3.761845007 | 0.000168665 | 0.002661467 | UP |
| SNX9 | ENSG00000130340.16 | 135.746606 | 2.250466204 | 0.319138552 | 7.051690217 | 1.76757E-12 | 1.72227E-10 | UP |
| SOX2-OT | ENSG00000242808.9 | 58.17680496 | 4.51866112 | 0.824406161 | 5.481110323 | 4.22665E-08 | 1.9275E-06 | UP |
| SOX4 | ENSG00000124766.7 | 7449.057809 | 1.207399141 | 0.253708581 | 4.759000013 | 1.94554E-06 | 5.54544E-05 | UP |
| SOX6 | ENSG00000110693.18 | 30.61677189 | 2.525933604 | 0.825376612 | 3.060340657 | 0.002210854 | 0.022047949 | UP |
| SP110 | ENSG00000135899.17 | 550.1521813 | -1.166046843 | 0.348930808 | -3.341770967 | 0.000832457 | 0.010031536 | DOWN |
| SPACA3 | ENSG00000141316.13 | 23.8415136 | 7.974531144 | 1.407718705 | 5.664861249 | 1.47144E-08 | 7.46755E-07 | UP |
| SPC24 | ENSG00000161888.11 | 511.5446851 | -1.185851797 | 0.400617818 | -2.96005755 | 0.003075816 | 0.028789959 | DOWN |
| SPC25 | ENSG00000152253.9 | 397.041214 | -1.120707326 | 0.390748476 | -2.868104152 | 0.004129396 | 0.036235854 | DOWN |
| SPECC1 | ENSG00000128487.16 | 272.4251008 | -1.284424847 | 0.267573706 | -4.800265569 | 1.58455E-06 | 4.67011E-05 | DOWN |
| SPIB | ENSG00000269404.7 | 628.0156229 | -1.599976286 | 0.422234518 | -3.789307166 | 0.000151068 | 0.002423876 | DOWN |
| SPIN2A | ENSG00000147059.8 | 35.84133084 | 1.329334462 | 0.466283839 | 2.850912579 | 0.004359395 | 0.037881276 | UP |
| SPINK4 | ENSG00000122711.9 | 45.01500895 | 8.113058599 | 1.271195395 | 6.382227808 | 1.7453E-10 | 1.27543E-08 | UP |
| SPTBN4 | ENSG00000160460.16 | 74.66763545 | 1.342294556 | 0.421238914 | 3.186539777 | 0.001439857 | 0.015674046 | UP |
| SRGAP1 | ENSG00000196935.9 | 72.26596459 | 2.426805029 | 0.699701423 | 3.468343711 | 0.000523677 | 0.006807098 | UP |
| SRP14-AS1 | ENSG00000248508.8 | 171.6714485 | 1.175100549 | 0.37333988 | 3.147535562 | 0.001646531 | 0.017348796 | UP |
| SRRD | ENSG00000100104.13 | 970.8660936 | -1.044277508 | 0.290621248 | -3.593259323 | 0.000326567 | 0.004625116 | DOWN |
| SSBP3-AS1 | ENSG00000198711.5 | 22.44390225 | 1.562965711 | 0.560334969 | 2.789341728 | 0.00528153 | 0.044257779 | UP |
| SSTR2 | ENSG00000180616.9 | 82.45716442 | 1.823948834 | 0.525449447 | 3.471216583 | 0.000518106 | 0.006758118 | UP |
| ST6GAL2 | ENSG00000144057.15 | 12.93584352 | 6.238248191 | 1.575707156 | 3.959014953 | 7.52595E-05 | 0.001328666 | UP |
| ST8SIA4 | ENSG00000113532.13 | 599.5036245 | 1.330721271 | 0.327439497 | 4.064021849 | 4.82343E-05 | 0.000912053 | UP |
| ST8SIA5 | ENSG00000101638.13 | 353.0926513 | -1.506078506 | 0.28997024 | -5.19390716 | 2.05926E-07 | 7.81248E-06 | DOWN |
| STAP1 | ENSG00000035720.8 | 936.3220385 | 6.744997875 | 0.325110475 | 20.74678732 | 1.31062E-95 | 2.33696E-91 | UP |
| STARD9 | ENSG00000159433.12 | 570.0996989 | -1.079126831 | 0.269207982 | -4.008524645 | 6.10993E-05 | 0.001123156 | DOWN |
| STAT5A | ENSG00000126561.16 | 368.0160457 | -1.091801225 | 0.232797628 | -4.689915596 | 2.73318E-06 | 7.54416E-05 | DOWN |
| STIL | ENSG00000123473.15 | 694.8998448 | -1.02073482 | 0.353428026 | -2.88809813 | 0.003875789 | 0.034502846 | DOWN |
| STK39 | ENSG00000198648.11 | 1566.704028 | -1.74965461 | 0.406276912 | -4.30655683 | 1.65815E-05 | 0.00036412 | DOWN |
| STON1 | ENSG00000243244.6 | 178.4161555 | 1.824840432 | 0.347849168 | 5.246068117 | 1.55379E-07 | 6.10258E-06 | UP |
| STOX1 | ENSG00000165730.16 | 46.70247595 | -1.789244472 | 0.509852302 | -3.509338814 | 0.000449222 | 0.006022618 | DOWN |
| STUM | ENSG00000203685.10 | 28.63617335 | 2.015357567 | 0.613576025 | 3.284609377 | 0.001021239 | 0.011801493 | UP |
| SYNDIG1 | ENSG00000101463.6 | 13.86232908 | 5.237684881 | 1.433393757 | 3.654044714 | 0.000258141 | 0.003810362 | UP |
| SYNE3 | ENSG00000176438.12 | 1770.585735 | -1.445215134 | 0.303342476 | -4.764301906 | 1.89509E-06 | 5.42396E-05 | DOWN |
| SYNGR1 | ENSG00000100321.15 | 1309.069768 | -1.682962229 | 0.26617465 | -6.322774276 | 2.56908E-10 | 1.83973E-08 | DOWN |
| SYTL2 | ENSG00000137501.17 | 772.6862066 | 2.069772672 | 0.47995453 | 4.312434915 | 1.61466E-05 | 0.00035582 | UP |
| TAFA1 | ENSG00000183662.11 | 41.214885 | -4.786301871 | 1.291488266 | -3.706035896 | 0.000210528 | 0.003234521 | DOWN |
| TARID | ENSG00000227954.7 | 6.237505483 | 6.014661629 | 1.706908849 | 3.52371577 | 0.00042554 | 0.005757065 | UP |
| TBC1D8 | ENSG00000204634.12 | 468.8040344 | 1.41587828 | 0.322898107 | 4.384907337 | 1.16035E-05 | 0.000269404 | UP |
| TBC1D9 | ENSG00000109436.8 | 336.3877984 | 1.145276999 | 0.379026785 | 3.021625497 | 0.002514214 | 0.024524589 | UP |
| TCF12 | ENSG00000140262.17 | 4089.919315 | 1.126293969 | 0.400925878 | 2.809232405 | 0.004965978 | 0.042145816 | UP |
| TCF7L1 | ENSG00000152284.5 | 10.6665 | 2.17784052 | 0.790520636 | 2.754944552 | 0.005870208 | 0.0480807 | UP |
| TDRD3 | ENSG00000083544.14 | 892.1746389 | 2.608224742 | 0.259205641 | 10.06237646 | 8.10186E-24 | 3.00967E-21 | UP |
| TEC | ENSG00000135605.13 | 235.029964 | -1.285546911 | 0.439433396 | -2.925464749 | 0.003439421 | 0.031411222 | DOWN |
| TENT5C | ENSG00000183508.5 | 40.24008749 | 2.583087665 | 0.530900229 | 4.865486061 | 1.14176E-06 | 3.5345E-05 | UP |
| TESMIN | ENSG00000132749.11 | 151.8431766 | -1.272968512 | 0.397280987 | -3.204201947 | 0.001354374 | 0.014888933 | DOWN |
| TEX41 | ENSG00000226674.11 | 68.80219961 | 3.823063578 | 0.547357729 | 6.984579506 | 2.8571E-12 | 2.67289E-10 | UP |
| TFEB | ENSG00000112561.18 | 547.7950295 | -1.2982596 | 0.276299167 | -4.698745972 | 2.61764E-06 | 7.27027E-05 | DOWN |
| TFPI | ENSG00000003436.16 | 206.9433758 | 1.053306938 | 0.281608431 | 3.740324584 | 0.000183783 | 0.002877112 | UP |
| TGFB2 | ENSG00000092969.12 | 13.18948629 | 3.624953567 | 1.046281946 | 3.464604909 | 0.000531011 | 0.006881146 | UP |
| TGFBR2 | ENSG00000163513.18 | 1726.261017 | 1.551100907 | 0.257825629 | 6.01608504 | 1.78686E-09 | 1.08742E-07 | UP |
| TGFBR3 | ENSG00000069702.11 | 179.1063428 | 2.926861076 | 0.389483333 | 7.514727403 | 5.70296E-14 | 6.60322E-12 | UP |
| THSD4 | ENSG00000187720.14 | 10.9488594 | 2.931792289 | 1.052226601 | 2.786274635 | 0.005331768 | 0.044604333 | UP |
| TIPARP | ENSG00000163659.13 | 492.3020737 | 1.135300711 | 0.409879378 | 2.76984101 | 0.005608366 | 0.046361973 | UP |
| TJP2 | ENSG00000119139.20 | 193.0045148 | -1.06088519 | 0.353170887 | -3.003886306 | 0.002665549 | 0.025733301 | DOWN |
| TLE4 | ENSG00000106829.19 | 1751.961955 | 1.809913049 | 0.309651506 | 5.844999985 | 5.06568E-09 | 2.85842E-07 | UP |
| TMCC3 | ENSG00000057704.13 | 104.1649971 | 9.218976662 | 1.232656109 | 7.478952643 | 7.49173E-14 | 8.45936E-12 | UP |
| TMEM100 | ENSG00000166292.12 | 17.12551073 | 4.05667495 | 0.981648806 | 4.132511469 | 3.58821E-05 | 0.00070155 | UP |
| TMEM119 | ENSG00000183160.9 | 170.7832043 | 6.546243293 | 0.573078394 | 11.42294555 | 3.21161E-30 | 1.9747E-27 | UP |
| TMEM132A | ENSG00000006118.14 | 1462.557711 | 6.526076309 | 0.576561361 | 11.31896229 | 1.05716E-29 | 6.08071E-27 | UP |
| TMEM132B | ENSG00000139364.10 | 12.72276189 | 5.684805981 | 1.510633268 | 3.763193955 | 0.000167757 | 0.002649491 | UP |
| TMEM178B | ENSG00000261115.6 | 40.8598129 | 2.381574239 | 0.573130431 | 4.155379144 | 3.24749E-05 | 0.000643399 | UP |
| TMEM181 | ENSG00000146433.8 | 774.8551331 | 1.292400148 | 0.373606069 | 3.459258975 | 0.000541664 | 0.006988718 | UP |
| TMEM237 | ENSG00000155755.19 | 1566.812446 | 1.13199514 | 0.367977034 | 3.076265733 | 0.002096109 | 0.021152081 | UP |
| TMEM273 | ENSG00000204161.14 | 389.3553938 | 1.71430456 | 0.289359899 | 5.92447179 | 3.13303E-09 | 1.82565E-07 | UP |
| TMEM87B | ENSG00000153214.11 | 897.4609296 | 1.805165459 | 0.566779192 | 3.184953656 | 0.001447772 | 0.015731396 | UP |
| TMPRSS3 | ENSG00000160183.16 | 80.59513412 | 5.621277039 | 0.633269285 | 8.876598275 | 6.89362E-19 | 1.63894E-16 | UP |
| TNFAIP8 | ENSG00000145779.8 | 205.3489192 | -1.395568919 | 0.442507947 | -3.153771424 | 0.001611753 | 0.017086304 | DOWN |
| TNS1 | ENSG00000079308.19 | 3576.938964 | -1.090653687 | 0.322435589 | -3.382547475 | 0.000718169 | 0.008813259 | DOWN |
| TOM1L2 | ENSG00000175662.17 | 700.7102342 | 1.327656012 | 0.304551619 | 4.359379267 | 1.30432E-05 | 0.000297789 | UP |
| TOR1B | ENSG00000136816.16 | 677.1916843 | 1.565524689 | 0.282495185 | 5.541774762 | 2.99421E-08 | 1.42754E-06 | UP |
| TP53INP2 | ENSG00000078804.13 | 6900.350796 | 4.543837355 | 0.354450249 | 12.81939388 | 1.27695E-37 | 1.50472E-34 | UP |
| TPST1 | ENSG00000169902.15 | 238.7766378 | 1.315722472 | 0.400909282 | 3.281845875 | 0.0010313 | 0.011871596 | UP |
| TRAF3IP3 | ENSG00000009790.15 | 333.0042421 | -1.413589077 | 0.391606832 | -3.609715051 | 0.000306534 | 0.004376141 | DOWN |
| TRAK2 | ENSG00000115993.13 | 1196.225214 | 1.068496062 | 0.371107336 | 2.879210294 | 0.003986724 | 0.035261545 | UP |
| TRANK1 | ENSG00000168016.14 | 587.1865113 | -1.122811768 | 0.341759884 | -3.285381991 | 0.001018442 | 0.011776808 | DOWN |
| TRIB1 | ENSG00000173334.4 | 819.9334355 | 1.530311118 | 0.312774668 | 4.892695203 | 9.94644E-07 | 3.13348E-05 | UP |
| TRIM22 | ENSG00000132274.16 | 3454.395232 | -1.187637419 | 0.40666685 | -2.920418564 | 0.003495615 | 0.031801183 | DOWN |
| TRIP13 | ENSG00000071539.14 | 754.4339878 | -1.249024904 | 0.249758581 | -5.000928886 | 5.70548E-07 | 1.92314E-05 | DOWN |
| TSACC | ENSG00000163467.11 | 298.7235849 | 4.287805649 | 0.440330402 | 9.737700675 | 2.08212E-22 | 6.87524E-20 | UP |
| TSC22D1 | ENSG00000102804.15 | 1124.528167 | 1.420514471 | 0.306447989 | 4.635417831 | 3.56217E-06 | 9.6238E-05 | UP |
| TSGA10 | ENSG00000135951.15 | 91.37372181 | -1.51010796 | 0.441229562 | -3.422499513 | 0.000620482 | 0.007824481 | DOWN |
| TSKU | ENSG00000182704.8 | 334.4909618 | 1.440411184 | 0.396248001 | 3.635125431 | 0.000277845 | 0.004044295 | UP |
| TSPAN18 | ENSG00000157570.12 | 61.47295772 | 2.006528534 | 0.485577593 | 4.13225108 | 3.59228E-05 | 0.000701576 | UP |
| TSPEAR | ENSG00000175894.18 | 18.66390251 | 3.068508154 | 0.948864603 | 3.233873564 | 0.001221235 | 0.013695499 | UP |
| TTC7A | ENSG00000068724.17 | 3654.471245 | -1.165182163 | 0.333618891 | -3.492554516 | 0.000478424 | 0.006326965 | DOWN |
| TTC7B | ENSG00000165914.15 | 835.3542819 | 1.48584966 | 0.335300773 | 4.431393485 | 9.36261E-06 | 0.00022289 | UP |
| TTYH2 | ENSG00000141540.11 | 510.1341154 | 1.120589854 | 0.341077618 | 3.285439431 | 0.001018234 | 0.011776808 | UP |
| TUBA1A | ENSG00000167552.14 | 8335.24154 | 1.142872236 | 0.328979482 | 3.473992449 | 0.000512775 | 0.006703299 | UP |
| UBASH3B | ENSG00000154127.10 | 6675.758437 | -2.232816003 | 0.460715039 | -4.846414411 | 1.25713E-06 | 3.85152E-05 | DOWN |
| UBE2QL1 | ENSG00000215218.4 | 247.303141 | 6.47157571 | 0.504997393 | 12.81506756 | 1.35021E-37 | 1.50472E-34 | UP |
| UNC5A | ENSG00000113763.12 | 7.741455231 | 5.340149988 | 1.517310283 | 3.519484477 | 0.000432386 | 0.005840819 | UP |
| UNC5B | ENSG00000107731.12 | 504.0760159 | 10.46731266 | 1.063805565 | 9.839497936 | 7.60896E-23 | 2.55991E-20 | UP |
| UNC5D | ENSG00000156687.11 | 127.3480492 | 6.430499202 | 0.665167985 | 9.667481525 | 4.14451E-22 | 1.31966E-19 | UP |
| USP20 | ENSG00000136878.13 | 1126.102788 | 1.62464604 | 0.440718837 | 3.686354892 | 0.000227489 | 0.003433629 | UP |
| UXS1 | ENSG00000115652.14 | 2261.749825 | 2.053146405 | 0.247315516 | 8.301729061 | 1.02607E-16 | 1.7763E-14 | UP |
| VAT1L | ENSG00000171724.3 | 455.8890833 | -1.756515915 | 0.310125198 | -5.663892933 | 1.47977E-08 | 7.47471E-07 | DOWN |
| VAV3 | ENSG00000134215.16 | 2240.746446 | -1.268520809 | 0.251361777 | -5.046593899 | 4.49756E-07 | 1.54818E-05 | DOWN |
| VLDLR | ENSG00000147852.16 | 46.93776033 | 3.322754038 | 0.772195589 | 4.302995364 | 1.68504E-05 | 0.000368211 | UP |
| VMO1 | ENSG00000182853.12 | 22.51892399 | 3.71863669 | 0.759766855 | 4.894444483 | 9.85838E-07 | 3.11675E-05 | UP |
| VWC2 | ENSG00000188730.5 | 153.2198914 | 8.757013841 | 1.117492087 | 7.836309489 | 4.63981E-15 | 6.41337E-13 | UP |
| WDFY4 | ENSG00000128815.19 | 3672.349427 | 1.97189507 | 0.502355053 | 3.925301558 | 8.66212E-05 | 0.001498101 | UP |
| WDR72 | ENSG00000166415.15 | 18.74803253 | 3.544627457 | 1.050725829 | 3.373503684 | 0.000742181 | 0.009064302 | UP |
| WEE1 | ENSG00000166483.11 | 838.4996402 | -1.16979663 | 0.364962524 | -3.205251373 | 0.001349446 | 0.014843903 | DOWN |
| WIPF1 | ENSG00000115935.17 | 8278.968653 | 1.459123118 | 0.244379206 | 5.97073353 | 2.36189E-09 | 1.40383E-07 | UP |
| WNT9A | ENSG00000143816.8 | 27.21839526 | 2.456652269 | 0.608372874 | 4.038070028 | 5.38928E-05 | 0.00100519 | UP |
| WT1 | ENSG00000184937.14 | 120.3242701 | -2.966817248 | 0.585937914 | -5.063364527 | 4.11921E-07 | 1.43177E-05 | DOWN |
| XBP1 | ENSG00000100219.16 | 7335.315469 | 1.183805098 | 0.295934115 | 4.000231934 | 6.32804E-05 | 0.0011561 | UP |
| YPEL2 | ENSG00000175155.10 | 276.5856216 | 1.612606503 | 0.449479328 | 3.587721176 | 0.000333581 | 0.004713216 | UP |
| ZBTB18 | ENSG00000179456.10 | 707.014289 | 1.647562849 | 0.333377687 | 4.942030955 | 7.73129E-07 | 2.52485E-05 | UP |
| ZBTB20 | ENSG00000181722.16 | 215.8770708 | 4.892083173 | 0.715465032 | 6.837627212 | 8.05156E-12 | 7.10729E-10 | UP |
| ZBTB42 | ENSG00000179627.10 | 67.39566406 | -1.266430187 | 0.456875191 | -2.771939056 | 0.005572347 | 0.046106968 | DOWN |
| ZDHHC22 | ENSG00000177108.5 | 174.5511599 | 9.05008923 | 1.135714017 | 7.968633911 | 1.60438E-15 | 2.30707E-13 | UP |
| ZEB1 | ENSG00000148516.21 | 417.1554803 | 1.28841587 | 0.366263654 | 3.51772789 | 0.000435258 | 0.005870721 | UP |
| ZFP36L2 | ENSG00000152518.8 | 3227.597412 | -2.16401276 | 0.348665947 | -6.206550362 | 5.41603E-10 | 3.70637E-08 | DOWN |
| ZFYVE28 | ENSG00000159733.13 | 39.05883161 | 4.640575133 | 0.651504445 | 7.122860278 | 1.0571E-12 | 1.05894E-10 | UP |
| ZNF385B | ENSG00000144331.20 | 13.96881905 | 7.208026025 | 1.43216095 | 5.032972045 | 4.82934E-07 | 1.656E-05 | UP |
| ZNF423 | ENSG00000102935.11 | 557.3868479 | -1.092169521 | 0.267413617 | -4.084195615 | 4.42298E-05 | 0.000844391 | DOWN |
| ZNF532 | ENSG00000074657.13 | 92.04493314 | 3.702758139 | 0.631210275 | 5.866124626 | 4.46099E-09 | 2.56579E-07 | UP |
| ZNF827 | ENSG00000151612.18 | 946.2043407 | 1.076633993 | 0.334689953 | 3.216810019 | 0.001296244 | 0.014356104 | UP |
| ZNF831 | ENSG00000124203.6 | 28.23429646 | 1.588318644 | 0.575791669 | 2.758495357 | 0.005806813 | 0.047627085 | UP |
| ZSWIM4 | ENSG00000132003.9 | 1002.517648 | 1.576862179 | 0.454376385 | 3.470387614 | 0.000519708 | 0.006774057 | UP |

**Supplementary Table 5.** **Differential gene expression in NALM-6*^TCF12-/-^* cells.**

| gene_id | BaseMean_DESeq2 | BaseMean_Control | BaseMean_KOTCF12 | FoldChange | log2FoldChange | p-value | q-value | Regulation |
| --- | --- | --- | --- | --- | --- | --- | --- | --- |
| A2M | 860.5764686 | 282.0690511 | 1439.083886 | 5.101096818 | 2.350807483 | 7.84E-112 | 2.62E-110 | Up |
| AARS1 | 15914.49237 | 25028.20417 | 6800.780577 | 0.271725727 | -1.879776931 | 1.58E-283 | 2.46E-281 | Down |
| AASS | 2817.554494 | 3898.847952 | 1736.261036 | 0.445312491 | -1.167110017 | 1.24E-51 | 1.34E-50 | Down |
| ABCA1 | 1430.498857 | 2273.336735 | 587.6609785 | 0.258494548 | -1.951794245 | 3.18E-102 | 9.12E-101 | Down |
| ABCA12 | 8.149304803 | 1.434810153 | 14.86379945 | 10.19067444 | 3.34917763 | 0.00323171 | 0.005287455 | Up |
| ABCA2 | 240.2177211 | 397.5931068 | 82.84233536 | 0.208402591 | -2.262554879 | 1.88E-36 | 1.35E-35 | Down |
| ABCA4 | 20.88659121 | 3.354713668 | 38.41846875 | 11.52568057 | 3.526780037 | 2.48E-06 | 5.24E-06 | Up |
| ABCB1 | 16.23838749 | 5.937781365 | 26.53899361 | 4.479602159 | 2.16337061 | 0.005728519 | 0.009126024 | Up |
| ABCB4 | 2726.425534 | 1623.675457 | 3829.175612 | 2.358299375 | 1.237746873 | 3.05E-88 | 7.06E-87 | Up |
| ABCC2 | 185.9363427 | 106.993205 | 264.8794803 | 2.475953627 | 1.307984294 | 6.81E-12 | 1.99E-11 | Up |
| ABCD1 | 339.7645741 | 491.924467 | 187.6046813 | 0.381243775 | -1.391214314 | 4.45E-20 | 1.85E-19 | Down |
| ABCD2 | 30.2277416 | 49.13972022 | 11.31576298 | 0.230377995 | -2.117925171 | 0.000325708 | 0.000589573 | Down |
| ABCE1 | 4638.486822 | 3050.807927 | 6226.165717 | 2.041073208 | 1.029327929 | 8.53E-52 | 9.28E-51 | Up |
| ABCG1 | 1299.661706 | 1823.007193 | 776.3162179 | 0.42586869 | -1.231519427 | 5.58E-42 | 4.72E-41 | Down |
| ABHD14B | 2823.991158 | 4106.920398 | 1541.061919 | 0.375243399 | -1.414101403 | 2.28E-105 | 6.96E-104 | Down |
| ABHD15 | 2039.061942 | 3347.875146 | 730.2487379 | 0.218115632 | -2.196834925 | 1.02E-135 | 4.60E-134 | Down |
| ABHD16A | 2108.0446 | 1386.034622 | 2830.054578 | 2.042475609 | 1.03031885 | 4.16E-40 | 3.33E-39 | Up |
| ABHD17C | 380.2869629 | 537.7483744 | 222.8255514 | 0.414376384 | -1.270986311 | 1.40E-17 | 5.26E-17 | Down |
| ABHD4 | 1992.649172 | 768.6439942 | 3216.654349 | 4.185750162 | 2.065486203 | 2.90E-135 | 1.30E-133 | Up |
| ABI2 | 3490.131928 | 4820.196659 | 2160.067197 | 0.44811129 | -1.158071021 | 2.25E-78 | 4.36E-77 | Down |
| ABI3BP | 33.33241737 | 51.17653475 | 15.4883 | 0.302296312 | -1.725964717 | 0.000258179 | 0.000472392 | Down |
| ABTB2 | 54.66060638 | 0 | 109.3212128 | 536.8029026 | 9.068248662 | 1.18E-13 | 3.74E-13 | Up |
| ACACB | 600.1611933 | 908.6674565 | 291.6549302 | 0.320898414 | -1.639811436 | 9.61E-43 | 8.29E-42 | Down |
| ACAD9 | 1309.234061 | 1748.294965 | 870.1731576 | 0.497667534 | -1.006745821 | 2.14E-34 | 1.45E-33 | Down |
| ACAP1 | 2540.01328 | 3856.169649 | 1223.856912 | 0.317367888 | -1.655771935 | 4.14E-97 | 1.09E-95 | Down |
| ACBD7 | 1331.177954 | 1993.166909 | 669.1889994 | 0.335711991 | -1.574704027 | 1.03E-73 | 1.80E-72 | Down |
| ACCS | 2.386578082 | 4.773156163 | 0 | 0.034444589 | -4.859578824 | 0.030439244 | 0.043976956 | Down |
| ACIN1 | 14884.91808 | 9839.025044 | 19930.81112 | 2.025589128 | 1.018341566 | 1.14E-105 | 3.51E-104 | Up |
| ACLY | 16981.25288 | 22695.21762 | 11267.28813 | 0.496461134 | -1.010247317 | 1.25E-82 | 2.66E-81 | Down |
| ACOT11 | 3.042917152 | 5.78708674 | 0.298747564 | 0.055222598 | -4.178597419 | 0.023468823 | 0.03442276 | Down |
| ACOXL | 5.468770466 | 1.117045182 | 9.82049575 | 8.841741444 | 3.144330547 | 0.015347227 | 0.023074072 | Up |
| ACP2 | 829.0100326 | 458.2765128 | 1199.743552 | 2.618171851 | 1.388559796 | 1.01E-38 | 7.81E-38 | Up |
| ACSBG1 | 8.462335402 | 1.464233454 | 15.46043735 | 10.51064667 | 3.393779529 | 0.001665085 | 0.002810806 | Up |
| ACSL1 | 743.2880309 | 343.017557 | 1143.558505 | 3.330348338 | 1.735673084 | 7.59E-54 | 8.72E-53 | Up |
| ACSM3 | 226.3454089 | 345.9230161 | 106.7678016 | 0.308544344 | -1.696450246 | 3.95E-18 | 1.52E-17 | Down |
| ACSS1 | 3358.055959 | 4818.273179 | 1897.838739 | 0.393888118 | -1.344142199 | 5.77E-78 | 1.11E-76 | Down |
| ACTA1 | 31.23819576 | 16.46681191 | 46.00957962 | 2.786582591 | 1.478496914 | 0.005337751 | 0.008538801 | Up |
| ACTA2 | 424.6937861 | 22.32911291 | 827.0584592 | 36.81908616 | 5.202381915 | 3.28E-109 | 1.06E-107 | Up |
| ACTG1 | 83577.85517 | 114995.85 | 52159.86035 | 0.453580964 | -1.140568001 | 3.22E-113 | 1.10E-111 | Down |
| ACTR3C | 1683.676138 | 2683.529812 | 683.8224642 | 0.254805614 | -1.972531031 | 3.38E-119 | 1.24E-117 | Down |
| ACTR8 | 4174.611822 | 2709.953774 | 5639.26987 | 2.08129216 | 1.057479497 | 6.48E-67 | 9.81E-66 | Up |
| ACVR1 | 767.3104202 | 392.5538841 | 1142.066956 | 2.909481512 | 1.540762079 | 2.73E-46 | 2.58E-45 | Up |
| ACVR1C | 9.021128431 | 0.731222196 | 17.31103467 | 23.5049335 | 4.554891694 | 0.000479271 | 0.000852085 | Up |
| ACVR2B | 1073.530114 | 1726.140902 | 420.9193252 | 0.243807376 | -2.036186321 | 1.73E-73 | 3.02E-72 | Down |
| ADA | 8110.878099 | 14928.58976 | 1293.166441 | 0.086614691 | -3.529244441 | 0 | 0 | Down |
| ADAM11 | 213.8503291 | 327.4293499 | 100.2713083 | 0.306025848 | -1.708274582 | 1.39E-17 | 5.24E-17 | Down |
| ADAM19 | 75.3610158 | 42.49961581 | 108.2224158 | 2.541118137 | 1.345463448 | 3.87E-05 | 7.56E-05 | Up |
| ADAM22 | 769.3171656 | 1291.619601 | 247.0147304 | 0.19126688 | -2.386341016 | 2.51E-55 | 2.98E-54 | Down |
| ADAM8 | 70.02956354 | 45.86200988 | 94.19711721 | 2.05561316 | 1.039568793 | 0.001024419 | 0.001768468 | Up |
| ADAMTS10 | 358.7121447 | 573.1918987 | 144.2323906 | 0.251621705 | -1.990671722 | 1.34E-44 | 1.21E-43 | Down |
| ADAMTS17 | 448.3638243 | 216.7654473 | 679.9622013 | 3.134484 | 1.648227966 | 1.76E-34 | 1.19E-33 | Up |
| ADAMTS2 | 31.43710498 | 61.9852197 | 0.888990269 | 0.014406067 | -6.117179717 | 9.45E-11 | 2.60E-10 | Down |
| ADAMTSL1 | 5.657927752 | 0 | 11.3158555 | 55.56630461 | 5.796138393 | 0.000185222 | 0.000343294 | Up |
| ADAMTSL2 | 41.46867163 | 21.50987885 | 61.42746442 | 2.860668115 | 1.516352131 | 0.000725411 | 0.001267436 | Up |
| ADAP2 | 60.2305642 | 23.19466207 | 97.26646634 | 4.18936335 | 2.066731017 | 4.71E-09 | 1.18E-08 | Up |
| ADARB1 | 2529.623154 | 3511.363505 | 1547.882804 | 0.440806943 | -1.181781147 | 2.64E-64 | 3.74E-63 | Down |
| ADARB2 | 177.5169238 | 314.8141116 | 40.21973596 | 0.127835881 | -2.967635264 | 3.01E-40 | 2.42E-39 | Down |
| ADAT2 | 767.8162414 | 1141.361717 | 394.2707662 | 0.345455419 | -1.533428554 | 1.91E-44 | 1.73E-43 | Down |
| ADAT3 | 142.6105215 | 78.80893931 | 206.4121037 | 2.625219793 | 1.392438216 | 2.07E-08 | 4.99E-08 | Up |
| ADCY10 | 16.04635513 | 6.125063599 | 25.96764667 | 4.200778703 | 2.070656787 | 0.005344318 | 0.008547335 | Up |
| ADCY7 | 200.4085426 | 68.1279573 | 332.6891279 | 4.897271378 | 2.291978144 | 5.96E-20 | 2.46E-19 | Up |
| ADD2 | 6236.754935 | 8823.601136 | 3649.908733 | 0.413668903 | -1.273451587 | 3.02E-104 | 9.06E-103 | Down |
| ADD3 | 3614.365881 | 5153.021114 | 2075.710647 | 0.402794841 | -1.311882889 | 1.63E-57 | 2.02E-56 | Down |
| ADGRA2 | 56.82970628 | 83.60768261 | 30.05172996 | 0.359781376 | -1.474807587 | 4.42E-05 | 8.59E-05 | Down |
| ADGRA3 | 1122.988231 | 1670.781683 | 575.1947801 | 0.344315839 | -1.538195548 | 8.43E-56 | 1.01E-54 | Down |
| ADGRE1 | 12.97950575 | 6.301603402 | 19.6574081 | 3.12537324 | 1.644028491 | 0.031701373 | 0.045648266 | Up |
| ADGRE2 | 311.3462536 | 534.4130743 | 88.27943285 | 0.165147488 | -2.598173071 | 2.35E-39 | 1.84E-38 | Down |
| ADGRF3 | 118.2088383 | 177.020854 | 59.39682255 | 0.335587656 | -1.575238446 | 3.02E-09 | 7.66E-09 | Down |
| ADGRF4 | 2.549085884 | 0 | 5.098171768 | 25.0206883 | 4.645049572 | 0.029735055 | 0.043040274 | Up |
| ADGRG1 | 54.0960795 | 97.17466871 | 11.01749029 | 0.113346383 | -3.141189741 | 1.86E-14 | 6.10E-14 | Down |
| ADGRG3 | 3.926108997 | 0.385822986 | 7.466395009 | 18.81779809 | 4.23402592 | 0.011240191 | 0.017205108 | Up |
| ADGRG7 | 4.179275841 | 0.356399685 | 8.002151997 | 20.1836267 | 4.335113523 | 0.011058792 | 0.016948017 | Up |
| ADGRL1 | 3339.818847 | 4523.690071 | 2155.947622 | 0.476618227 | -1.069093971 | 1.41E-52 | 1.58E-51 | Down |
| ADGRL2 | 11.05441462 | 22.10882925 | 0 | 0.00743875 | -7.070724129 | 3.18E-07 | 7.16E-07 | Down |
| ADH1C | 20.56476687 | 0 | 41.12953375 | 201.9483563 | 7.657842593 | 5.63E-09 | 1.41E-08 | Up |
| ADHFE1 | 9.198315878 | 2.621702411 | 15.77492935 | 6.066367747 | 2.600832958 | 0.005567163 | 0.008878146 | Up |
| ADM | 31.25622766 | 0.771645971 | 61.74080936 | 83.39015285 | 6.381805127 | 7.74E-09 | 1.92E-08 | Up |
| ADORA2A | 1005.089027 | 101.0472191 | 1909.130835 | 18.81157521 | 4.233548754 | 2.00E-205 | 1.82E-203 | Up |
| ADORA3 | 12.46167575 | 0.751434084 | 24.17191742 | 32.6575719 | 5.029345625 | 3.67E-05 | 7.18E-05 | Up |
| ADRB1 | 41.84148608 | 60.17353186 | 23.50944029 | 0.390531866 | -1.356487824 | 0.001056993 | 0.001820406 | Down |
| ADRB3 | 19.35368764 | 34.55391907 | 4.153456203 | 0.120417904 | -3.053878188 | 1.80E-05 | 3.59E-05 | Down |
| AEBP1 | 43535.45499 | 63127.24619 | 23943.66379 | 0.379295068 | -1.398607481 | 2.71E-188 | 2.10E-186 | Down |
| AEN | 4257.363454 | 2099.996098 | 6414.73081 | 3.05432416 | 1.610853186 | 5.59E-112 | 1.88E-110 | Up |
| AFF3 | 2623.573799 | 3652.737983 | 1594.409615 | 0.436490798 | -1.195976855 | 2.09E-77 | 3.99E-76 | Down |
| AFTPH | 3323.639212 | 1841.410001 | 4805.868422 | 2.609240193 | 1.383629757 | 9.10E-97 | 2.38E-95 | Up |
| AGAP3 | 185.7750806 | 277.9452788 | 93.60488246 | 0.336736943 | -1.570306088 | 1.54E-11 | 4.42E-11 | Down |
| AGBL5 | 2322.783392 | 3113.486851 | 1532.079934 | 0.49206915 | -1.023067025 | 4.42E-46 | 4.18E-45 | Down |
| AGER | 368.8842815 | 512.6936623 | 225.0749008 | 0.438836167 | -1.188245665 | 1.96E-16 | 6.98E-16 | Down |
| AGO2 | 4565.230694 | 2966.403862 | 6164.057526 | 2.07797062 | 1.055175257 | 3.29E-68 | 5.14E-67 | Up |
| AGRN | 522.0341622 | 848.1933751 | 195.8749492 | 0.230945286 | -2.114376994 | 1.60E-65 | 2.34E-64 | Down |
| AHCY | 6480.726803 | 9504.222786 | 3457.230821 | 0.363748518 | -1.458986727 | 1.01E-154 | 5.64E-153 | Down |
| AHCYL1 | 8151.866013 | 11026.16746 | 5277.564569 | 0.478654691 | -1.062942846 | 4.35E-104 | 1.30E-102 | Down |
| AHNAK2 | 22.05232076 | 36.05525623 | 8.049385288 | 0.223490067 | -2.161717381 | 0.000224185 | 0.000412204 | Down |
| AHRR | 289.9049671 | 57.37222762 | 522.4377066 | 9.12438502 | 3.189727325 | 4.61E-66 | 6.85E-65 | Up |
| AIG1 | 325.327045 | 480.4468002 | 170.2072897 | 0.354158438 | -1.497533177 | 7.08E-22 | 3.13E-21 | Down |
| AJM1 | 125.3617957 | 73.57754183 | 177.1460496 | 2.405930503 | 1.26659497 | 4.94E-06 | 1.02E-05 | Up |
| AJUBA | 784.7090796 | 1141.240928 | 428.1772313 | 0.375098895 | -1.414657082 | 1.97E-42 | 1.69E-41 | Down |
| AK3 | 1503.754712 | 2107.047379 | 900.4620453 | 0.427334815 | -1.226561236 | 1.72E-28 | 9.80E-28 | Down |
| AK5 | 43.28832342 | 17.97504726 | 68.60159958 | 3.807230751 | 1.928742012 | 2.61E-06 | 5.50E-06 | Up |
| AKAP14 | 5.051751127 | 8.910516277 | 1.192985976 | 0.133977781 | -2.899934336 | 0.019156855 | 0.028462375 | Down |
| AKAP17A-2 | 81.98371851 | 25.66923991 | 138.2981971 | 5.390760292 | 2.43048876 | 0.011124903 | 0.017043686 | Up |
| AKAP3 | 98.63075631 | 23.56743747 | 173.6940751 | 7.357103968 | 2.879137979 | 0.000268753 | 0.000491027 | Up |
| AKAP5 | 88.75914542 | 153.6874885 | 23.83080236 | 0.155175829 | -2.688024244 | 5.43E-17 | 1.99E-16 | Down |
| AKAP9 | 22930.81323 | 34238.34347 | 11623.28298 | 0.339481712 | -1.558594235 | 5.81E-23 | 2.69E-22 | Down |
| AKIRIN1 | 5664.304138 | 3178.729219 | 8149.879057 | 2.564316711 | 1.358574456 | 6.36E-109 | 2.05E-107 | Up |
| AKIRIN2 | 3684.594506 | 1731.229419 | 5637.959593 | 3.255667815 | 1.702953505 | 4.79E-131 | 2.02E-129 | Up |
| AKR1B1 | 6834.297558 | 9195.120928 | 4473.474189 | 0.486501464 | -1.03948395 | 7.79E-81 | 1.61E-79 | Down |
| AKR7A2 | 1534.644669 | 2074.116086 | 995.1732519 | 0.479882403 | -1.059247183 | 4.11E-38 | 3.11E-37 | Down |
| AKT1 | 8756.813157 | 11750.67827 | 5762.94804 | 0.490434586 | -1.027867371 | 9.63E-65 | 1.38E-63 | Down |
| AKT1S1 | 1546.38247 | 717.8214964 | 2374.943443 | 3.308009713 | 1.725963471 | 8.31E-102 | 2.35E-100 | Up |
| ALAD | 2201.067376 | 3053.750873 | 1348.38388 | 0.44152544 | -1.179431529 | 1.30E-56 | 1.59E-55 | Down |
| ALB | 25.04641556 | 1.818844078 | 48.27398704 | 26.24766352 | 4.7141171 | 5.59E-09 | 1.40E-08 | Up |
| ALDH2 | 164.8009127 | 81.39943742 | 248.2023879 | 3.052633059 | 1.610054181 | 5.14E-14 | 1.65E-13 | Up |
| ALDH8A1 | 28.79302745 | 13.80263055 | 43.78342434 | 3.155816383 | 1.658013266 | 0.001979005 | 0.003322534 | Up |
| ALG1 | 1040.876142 | 630.9593946 | 1450.79289 | 2.300305454 | 1.201825447 | 1.49E-29 | 8.72E-29 | Up |
| ALG10 | 1370.361251 | 1899.23383 | 841.4886719 | 0.443086569 | -1.174339498 | 2.38E-25 | 1.21E-24 | Down |
| ALG1L2 | 31.82571002 | 53.81718413 | 9.834235899 | 0.182728357 | -2.452227558 | 5.90E-07 | 1.30E-06 | Down |
| ALG2 | 1422.271544 | 722.1001312 | 2122.442957 | 2.939840268 | 1.555737771 | 1.81E-71 | 3.04E-70 | Up |
| ALKBH1 | 1356.430115 | 681.3766386 | 2031.483592 | 2.981035223 | 1.575813422 | 9.01E-66 | 1.32E-64 | Up |
| ALKBH7 | 1279.930967 | 1775.007093 | 784.8548419 | 0.442156283 | -1.177371706 | 5.89E-30 | 3.51E-29 | Down |
| ALLC | 23.68098542 | 4.44796884 | 42.91400201 | 9.670855331 | 3.273643493 | 5.17E-06 | 1.07E-05 | Up |
| ALOX5 | 3419.498667 | 5235.180466 | 1603.816868 | 0.306340772 | -1.706790703 | 1.22E-184 | 8.93E-183 | Down |
| ALOX5AP | 186.2125045 | 25.03524905 | 347.38976 | 13.83629171 | 3.790385431 | 4.37E-11 | 1.22E-10 | Up |
| ALPK1 | 2596.485633 | 3996.880194 | 1196.091072 | 0.299281258 | -1.740426162 | 1.70E-111 | 5.65E-110 | Down |
| ALPK2 | 10.32564189 | 0.356399685 | 20.2948841 | 51.15472462 | 5.676795588 | 5.43E-05 | 0.000105181 | Up |
| ALPK3 | 122.1563284 | 54.64920772 | 189.6634491 | 3.463908865 | 1.792400973 | 8.95E-12 | 2.59E-11 | Up |
| ALPP | 27.74033074 | 38.77723588 | 16.7034256 | 0.430189582 | -1.216955506 | 0.026605512 | 0.038757095 | Down |
| AMBP | 3.710218823 | 7.420437645 | 0 | 0.022182052 | -5.494463347 | 0.001667119 | 0.002813212 | Down |
| AMIGO1 | 35.73552326 | 9.536834806 | 61.9342117 | 6.475787146 | 2.695055566 | 2.64E-08 | 6.32E-08 | Up |
| AMN | 336.031414 | 477.6004296 | 194.4623984 | 0.407199289 | -1.296193054 | 2.19E-17 | 8.15E-17 | Down |
| AMN1 | 169.3247579 | 232.4688177 | 106.180698 | 0.456696664 | -1.130691843 | 2.97E-08 | 7.11E-08 | Down |
| AMPD3 | 404.0109888 | 158.4770284 | 649.5449491 | 4.097123856 | 2.034611506 | 3.31E-47 | 3.23E-46 | Up |
| AMT | 205.0647186 | 354.3745503 | 55.7548869 | 0.157282619 | -2.668568842 | 9.69E-39 | 7.48E-38 | Down |
| ANAPC10 | 1147.936717 | 746.8920393 | 1548.981394 | 2.073858424 | 1.052317409 | 7.25E-35 | 4.95E-34 | Up |
| ANGPT4 | 3.129834442 | 0 | 6.259668884 | 30.73483586 | 4.941802875 | 0.005879591 | 0.009357023 | Up |
| ANGPTL2 | 52.19985331 | 27.19769522 | 77.20201141 | 2.835022398 | 1.503360133 | 6.59E-05 | 0.000126998 | Up |
| ANGPTL6 | 51.27128973 | 77.55552812 | 24.98705133 | 0.322204364 | -1.633952061 | 1.98E-05 | 3.96E-05 | Down |
| ANK1 | 90.39278578 | 37.550444 | 143.2351276 | 3.811556715 | 1.930380343 | 3.57E-09 | 9.03E-09 | Up |
| ANKDD1A | 19.43639983 | 28.78141899 | 10.09138067 | 0.350434456 | -1.512783461 | 0.02710622 | 0.039457486 | Down |
| ANKK1 | 6.871286512 | 0 | 13.74257302 | 67.46136758 | 6.07598966 | 5.76E-05 | 0.000111258 | Up |
| ANKRD10 | 15150.60556 | 22196.63362 | 8104.577507 | 0.365130775 | -1.453514825 | 1.65E-78 | 3.23E-77 | Down |
| ANKRD13D | 1635.982226 | 2317.90114 | 954.0633126 | 0.411528426 | -1.280936008 | 1.30E-45 | 1.21E-44 | Down |
| ANKRD24 | 101.5943006 | 38.10333733 | 165.0852638 | 4.320035247 | 2.111043083 | 3.69E-14 | 1.19E-13 | Up |
| ANKRD30B | 957.4897843 | 1663.280664 | 251.698905 | 0.151364306 | -2.723903063 | 2.63E-149 | 1.36E-147 | Down |
| ANKRD34A | 56.78319623 | 30.65373443 | 82.91265804 | 2.705809027 | 1.436060019 | 2.51E-05 | 4.97E-05 | Up |
| ANKRD36 | 666.1887361 | 1029.505571 | 302.8719017 | 0.29423839 | -1.764942604 | 1.06E-43 | 9.36E-43 | Down |
| ANKRD36B | 622.7904911 | 910.3223165 | 335.2586658 | 0.368196376 | -1.441452666 | 6.30E-30 | 3.74E-29 | Down |
| ANKRD36C | 659.7126514 | 999.4446452 | 319.9806576 | 0.320191386 | -1.642993597 | 3.98E-29 | 2.31E-28 | Down |
| ANKRD37 | 176.5058327 | 23.10639217 | 329.9052733 | 14.22199083 | 3.830051526 | 3.10E-50 | 3.24E-49 | Up |
| ANKRD53 | 16.13228985 | 7.764047794 | 24.50053191 | 3.157309833 | 1.658695842 | 0.013129082 | 0.019907465 | Up |
| ANKRD6 | 14.6620401 | 7.308377733 | 22.01570247 | 3.002876177 | 1.586344987 | 0.027517144 | 0.040009466 | Up |
| ANKRD65 | 9.452373407 | 0.365611098 | 18.53913572 | 46.72174586 | 5.546022279 | 0.000126978 | 0.000239049 | Up |
| ANKRD9 | 181.9384054 | 299.2018645 | 64.67494634 | 0.216206595 | -2.209517562 | 2.24E-26 | 1.19E-25 | Down |
| ANO8 | 699.0959603 | 1102.403855 | 295.788066 | 0.268252558 | -1.898336165 | 7.71E-66 | 1.14E-64 | Down |
| ANO9 | 32.85515857 | 1.078410468 | 64.63190667 | 58.56554024 | 5.871980132 | 3.07E-10 | 8.24E-10 | Up |
| ANP32B | 10971.48713 | 15105.83282 | 6837.141441 | 0.452622553 | -1.143619621 | 2.54E-139 | 1.19E-137 | Down |
| ANP32E | 6301.323678 | 8632.538923 | 3970.108433 | 0.459903286 | -1.120597588 | 7.54E-40 | 5.97E-39 | Down |
| ANPEP | 75.27977741 | 28.05966626 | 122.4998886 | 4.367272202 | 2.126732454 | 1.64E-10 | 4.48E-10 | Up |
| ANTXR2 | 19.65229588 | 1.42559874 | 37.87899302 | 25.86622045 | 4.692997359 | 3.18E-07 | 7.16E-07 | Up |
| ANXA2R | 283.2643539 | 391.8256998 | 174.703008 | 0.445934029 | -1.1650978 | 6.27E-14 | 2.01E-13 | Down |
| AOC2 | 407.2720593 | 209.4077004 | 605.1364181 | 2.887660248 | 1.52990101 | 8.60E-28 | 4.77E-27 | Up |
| AOPEP | 236.7231486 | 125.3638108 | 348.0824864 | 2.77900829 | 1.474570138 | 1.07E-16 | 3.87E-16 | Up |
| AP1M2 | 12.83945799 | 1.513868642 | 24.16504735 | 16.25514362 | 4.022824398 | 4.31E-05 | 8.40E-05 | Up |
| AP3D1 | 7101.587877 | 4704.459951 | 9498.715803 | 2.018970049 | 1.013619509 | 3.51E-69 | 5.61E-68 | Up |
| AP4S1 | 581.0201008 | 325.4155685 | 836.624633 | 2.573543606 | 1.363756227 | 5.31E-28 | 2.96E-27 | Up |
| AP5Z1 | 2013.597423 | 1212.48366 | 2814.711186 | 2.320880117 | 1.214672004 | 3.05E-48 | 3.05E-47 | Up |
| APBB1 | 6.123084104 | 1.839055965 | 10.40711224 | 5.651856788 | 2.49872491 | 0.028057492 | 0.040752405 | Up |
| APBB1IP | 7278.861599 | 10921.58197 | 3636.141231 | 0.332938536 | -1.58667223 | 8.50E-193 | 6.82E-191 | Down |
| APIP | 788.5041273 | 1060.988517 | 516.0197382 | 0.486402958 | -1.039776093 | 3.86E-26 | 2.03E-25 | Down |
| APLF | 293.5725053 | 142.7470639 | 444.3979467 | 3.116087244 | 1.639735626 | 2.40E-24 | 1.17E-23 | Up |
| APLP1 | 33.57587089 | 46.56049676 | 20.59124503 | 0.442228337 | -1.177136623 | 0.010938205 | 0.016768771 | Down |
| APOBEC3B | 1303.251639 | 1961.736363 | 644.7669141 | 0.328660133 | -1.60533163 | 8.85E-66 | 1.30E-64 | Down |
| APOBEC3H | 148.0972302 | 83.50377775 | 212.6906826 | 2.545272146 | 1.34781992 | 3.63E-07 | 8.13E-07 | Up |
| APOBR | 184.480404 | 99.95296515 | 269.0078428 | 2.688334784 | 1.426712811 | 2.28E-11 | 6.48E-11 | Up |
| APOC1 | 3.142143835 | 0 | 6.28428767 | 30.84447429 | 4.946940152 | 0.007371615 | 0.011564315 | Up |
| APOL1 | 324.8771256 | 482.2820534 | 167.4721978 | 0.347303981 | -1.525729146 | 5.10E-17 | 1.87E-16 | Down |
| APOL6 | 3091.39495 | 4793.782425 | 1389.007475 | 0.289736001 | -1.787189137 | 6.65E-176 | 4.61E-174 | Down |
| APOM | 106.5304196 | 47.77833527 | 165.282504 | 3.464162407 | 1.792506568 | 9.20E-11 | 2.53E-10 | Up |
| APOO | 194.4626434 | 276.927754 | 111.9975328 | 0.404646679 | -1.305265338 | 2.69E-11 | 7.62E-11 | Down |
| APP | 11367.50871 | 15938.55522 | 6796.462211 | 0.426415942 | -1.229666719 | 7.49E-118 | 2.69E-116 | Down |
| APPBP2 | 2011.548946 | 1334.965561 | 2688.132331 | 2.01377338 | 1.009901339 | 4.18E-31 | 2.57E-30 | Up |
| AQP3 | 20.41416317 | 4.124570578 | 36.70375576 | 8.984316355 | 3.167408729 | 3.45E-06 | 7.23E-06 | Up |
| AQP5 | 14.26434263 | 27.33407735 | 1.194607907 | 0.04364773 | -4.517949558 | 2.47E-06 | 5.22E-06 | Down |
| AQP7 | 7.794322594 | 14.09328544 | 1.495359753 | 0.105989302 | -3.238009438 | 0.003092471 | 0.005068013 | Down |
| ARFIP1 | 1077.164541 | 650.7087921 | 1503.62029 | 2.310030859 | 1.207912124 | 1.39E-37 | 1.04E-36 | Up |
| ARHGAP18 | 502.956377 | 722.8090368 | 283.1037172 | 0.391660532 | -1.35232434 | 3.19E-15 | 1.08E-14 | Down |
| ARHGAP24 | 67.90752445 | 17.72739547 | 118.0876534 | 6.663884389 | 2.736363372 | 2.16E-11 | 6.15E-11 | Up |
| ARHGAP29 | 9.493354025 | 2.550066274 | 16.43664178 | 6.40544102 | 2.679297905 | 0.008512389 | 0.013235663 | Up |
| ARHGAP33 | 1028.64378 | 1497.168482 | 560.1190791 | 0.374107208 | -1.418476332 | 8.15E-51 | 8.62E-50 | Down |
| ARHGAP6 | 920.2428869 | 465.0857669 | 1375.400007 | 2.955820975 | 1.563558892 | 4.03E-54 | 4.68E-53 | Up |
| ARHGEF12 | 39.45654236 | 8.9435177 | 69.96956702 | 7.866805521 | 2.975777918 | 2.96E-09 | 7.51E-09 | Up |
| ARHGEF17 | 240.4848209 | 370.3756663 | 110.5939754 | 0.298593782 | -1.743743972 | 6.83E-23 | 3.15E-22 | Down |
| ARHGEF28 | 22.70809344 | 4.144782465 | 41.27140442 | 10.06556801 | 3.331356682 | 1.39E-05 | 2.81E-05 | Up |
| ARHGEF37 | 19.73163924 | 0.722010783 | 38.74126769 | 52.63765728 | 5.718023376 | 6.72E-07 | 1.48E-06 | Up |
| ARHGEF39 | 2002.831679 | 2989.392418 | 1016.27094 | 0.339979288 | -1.556481236 | 1.24E-111 | 4.13E-110 | Down |
| ARHGEF40 | 1519.519052 | 941.8823152 | 2097.155789 | 2.226611967 | 1.154850161 | 7.59E-52 | 8.28E-51 | Up |
| ARHGEF9 | 1515.395916 | 2214.179382 | 816.61245 | 0.368789248 | -1.439131502 | 3.55E-67 | 5.39E-66 | Down |
| ARID3A | 1816.382312 | 2781.527509 | 851.2371155 | 0.306033135 | -1.708240227 | 2.12E-119 | 7.84E-118 | Down |
| ARID3B | 756.5647775 | 283.1906732 | 1229.938882 | 4.340785307 | 2.117956069 | 5.75E-70 | 9.35E-69 | Up |
| ARID3C | 10.79484516 | 17.13023413 | 4.459456193 | 0.260846221 | -1.938728561 | 0.031185981 | 0.044966813 | Down |
| ARID4B | 3249.794556 | 1785.644846 | 4713.944266 | 2.639875371 | 1.400469822 | 4.52E-50 | 4.70E-49 | Up |
| ARID5A | 12107.03997 | 4370.94764 | 19843.1323 | 4.539883033 | 2.182655128 | 0 | 0 | Up |
| ARL17A | 364.0038696 | 564.3146914 | 163.6930477 | 0.290111785 | -1.785319194 | 3.20E-30 | 1.92E-29 | Down |
| ARL17B | 410.4301587 | 612.1198787 | 208.7404386 | 0.341133483 | -1.551591729 | 3.24E-26 | 1.71E-25 | Down |
| ARL4D | 1368.020452 | 799.5489666 | 1936.491937 | 2.422085682 | 1.276249901 | 6.40E-49 | 6.47E-48 | Up |
| ARL8B | 3408.162605 | 1995.93161 | 4820.393599 | 2.414890346 | 1.271957681 | 2.31E-100 | 6.34E-99 | Up |
| ARL9 | 23.40258465 | 1.493656754 | 45.31151254 | 30.6431172 | 4.937491159 | 1.37E-08 | 3.35E-08 | Up |
| ARMC2 | 61.78462131 | 100.3057786 | 23.26346398 | 0.23185877 | -2.108681796 | 8.69E-10 | 2.27E-09 | Down |
| ARMC5 | 483.6345723 | 302.3869446 | 664.8822 | 2.197891035 | 1.136119863 | 4.93E-18 | 1.89E-17 | Up |
| ARMC7 | 560.0300337 | 342.4606029 | 777.5994646 | 2.270890769 | 1.183258313 | 1.54E-22 | 6.99E-22 | Up |
| ARMCX1 | 9.460413694 | 0.751434084 | 18.1693933 | 24.53715315 | 4.616895969 | 0.000263733 | 0.000482108 | Up |
| ARMCX3 | 1901.440521 | 1209.107437 | 2593.773605 | 2.145286084 | 1.101170051 | 2.41E-50 | 2.52E-49 | Up |
| ARMH1 | 82.5557447 | 122.4711413 | 42.64034811 | 0.347958041 | -1.523014749 | 2.35E-07 | 5.35E-07 | Down |
| ARMH4 | 913.9198274 | 1520.912468 | 306.9271868 | 0.201853672 | -2.308618263 | 8.84E-98 | 2.33E-96 | Down |
| ARNTL2 | 4.495963293 | 0.356399685 | 8.6355269 | 21.77150562 | 4.444369276 | 0.006327888 | 0.010022167 | Up |
| ARPP21 | 4649.563556 | 7015.691175 | 2283.435938 | 0.325470405 | -1.619401728 | 2.88E-167 | 1.83E-165 | Down |
| ARRB1 | 1775.42672 | 2550.829497 | 1000.023942 | 0.392015241 | -1.35101835 | 4.32E-53 | 4.87E-52 | Down |
| ARRDC3 | 3589.661047 | 1154.708419 | 6024.613676 | 5.216780392 | 2.383159702 | 4.80E-258 | 6.22E-256 | Up |
| ARRDC4 | 9548.96311 | 258.2905413 | 18839.63568 | 73.02953574 | 6.190408154 | 0 | 0 | Up |
| ARRDC5 | 68.18729112 | 108.6705578 | 27.70402441 | 0.255079896 | -1.970978894 | 5.74E-10 | 1.52E-09 | Down |
| ARSA | 436.8304985 | 688.0673114 | 185.5936856 | 0.269745563 | -1.890328865 | 1.95E-38 | 1.49E-37 | Down |
| ARSH | 3.280019189 | 0 | 6.560038379 | 32.20849603 | 5.009369391 | 0.004681355 | 0.007528705 | Up |
| ARTN | 25.0807234 | 8.698919882 | 41.46252692 | 4.736191444 | 2.243727398 | 0.000518081 | 0.000918376 | Up |
| ASB13 | 1877.591615 | 2667.318033 | 1087.865198 | 0.40783918 | -1.293927716 | 4.26E-66 | 6.34E-65 | Down |
| ASB2 | 5.219920341 | 0 | 10.43984068 | 51.25638009 | 5.679659689 | 0.000530861 | 0.000939711 | Up |
| ASB3 | 40.26640016 | 26.56447848 | 53.96832183 | 2.038998073 | 1.027860412 | 0.023796621 | 0.03487037 | Up |
| ASB4 | 38.10985081 | 0 | 76.21970161 | 374.2785289 | 8.547968478 | 7.26E-12 | 2.12E-11 | Up |
| ASCC3 | 3245.32723 | 2141.782874 | 4348.871586 | 2.030536284 | 1.021860807 | 5.19E-45 | 4.77E-44 | Up |
| ASF1B | 5643.270934 | 7592.292331 | 3694.249538 | 0.48659863 | -1.039195835 | 1.45E-51 | 1.57E-50 | Down |
| ASIC1 | 598.9257593 | 1070.301456 | 127.5500623 | 0.119170708 | -3.068898432 | 2.43E-108 | 7.79E-107 | Down |
| ASNS | 3325.810241 | 5941.834579 | 709.785903 | 0.119455204 | -3.065458395 | 0 | 0 | Down |
| ASS1 | 1840.784967 | 3642.554827 | 39.01510665 | 0.010718665 | -6.543731014 | 0 | 0 | Down |
| ASXL1 | 8807.060409 | 12035.49357 | 5578.627252 | 0.463498568 | -1.109363213 | 3.13E-99 | 8.45E-98 | Down |
| ASXL3 | 2.530288767 | 0 | 5.060577534 | 24.84973922 | 4.635158807 | 0.030352511 | 0.043860785 | Up |
| ATAD2B | 3023.016943 | 1998.51957 | 4047.514316 | 2.025247497 | 1.018098225 | 8.63E-36 | 6.08E-35 | Up |
| ATAD5 | 2349.586063 | 3300.279065 | 1398.89306 | 0.423870036 | -1.238306111 | 4.35E-35 | 3.01E-34 | Down |
| ATAT1 | 586.5634504 | 794.1453896 | 378.9815113 | 0.477262893 | -1.067143922 | 1.80E-21 | 7.85E-21 | Down |
| ATF3 | 4282.88775 | 845.1026729 | 7720.672827 | 9.140121079 | 3.192213277 | 0 | 0 | Up |
| ATF5 | 41801.54492 | 66822.37188 | 16780.71796 | 0.251125206 | -1.993521254 | 0 | 0 | Down |
| ATG101 | 2598.324282 | 1635.071052 | 3561.577513 | 2.177881926 | 1.12292574 | 2.10E-56 | 2.54E-55 | Up |
| ATG16L2 | 1590.989856 | 2525.541928 | 656.4377834 | 0.259885512 | -1.944051884 | 2.57E-134 | 1.14E-132 | Down |
| ATOH8 | 13.74150838 | 24.48619189 | 2.99682488 | 0.122170188 | -3.033035815 | 0.000420328 | 0.000752791 | Down |
| ATP11C | 1660.281733 | 2291.601947 | 1028.96152 | 0.448995976 | -1.155225578 | 2.86E-27 | 1.56E-26 | Down |
| ATP13A1 | 4199.870655 | 2669.348714 | 5730.392596 | 2.14655466 | 1.10202291 | 1.63E-67 | 2.50E-66 | Up |
| ATP13A2 | 2161.166892 | 1146.370025 | 3175.963758 | 2.76994384 | 1.469856726 | 1.10E-64 | 1.57E-63 | Up |
| ATP2A1 | 479.4533033 | 297.522149 | 661.3844576 | 2.220789786 | 1.151072838 | 4.70E-15 | 1.58E-14 | Up |
| ATP2A3 | 11072.79122 | 15303.04366 | 6842.538772 | 0.447137854 | -1.161208406 | 3.69E-77 | 6.99E-76 | Down |
| ATP2B3 | 36.62686102 | 69.08251712 | 4.171204915 | 0.060408424 | -4.049106437 | 3.31E-13 | 1.02E-12 | Down |
| ATP2C2 | 97.58660818 | 136.4239837 | 58.74923262 | 0.430933789 | -1.214461872 | 6.79E-06 | 1.40E-05 | Down |
| ATP4B | 66.86788506 | 121.8332981 | 11.902472 | 0.097720019 | -3.355202052 | 1.70E-18 | 6.69E-18 | Down |
| ATP6AP1L | 101.5696881 | 146.7593166 | 56.38005962 | 0.384062303 | -1.380587728 | 5.51E-07 | 1.22E-06 | Down |
| ATP6V0A4 | 2.538589597 | 0 | 5.077179194 | 24.92394386 | 4.639460468 | 0.015671324 | 0.023540928 | Up |
| ATP6V0B | 4223.175309 | 2580.379816 | 5865.970802 | 2.273379482 | 1.184838525 | 8.95E-66 | 1.31E-64 | Up |
| ATP6V0E1 | 3180.392564 | 1918.358458 | 4442.426669 | 2.316190344 | 1.211753819 | 1.45E-59 | 1.89E-58 | Up |
| ATP6V1FNB | 125.3191921 | 26.98662297 | 223.6517612 | 8.298469734 | 3.052845323 | 7.44E-28 | 4.13E-27 | Up |
| ATP6V1G3 | 2.256091312 | 4.512182625 | 0 | 0.036520333 | -4.775156266 | 0.030344432 | 0.043854542 | Down |
| ATP6V1H | 1684.766634 | 905.0151978 | 2464.518071 | 2.722476334 | 1.444919508 | 3.23E-77 | 6.13E-76 | Up |
| ATP7B | 376.9437826 | 535.2648927 | 218.6226725 | 0.408580676 | -1.291307122 | 2.20E-17 | 8.18E-17 | Down |
| ATP8A1 | 2883.71798 | 4408.917416 | 1358.518544 | 0.308138745 | -1.698347997 | 1.12E-61 | 1.52E-60 | Down |
| ATP8B3 | 449.6519794 | 786.068686 | 113.2352729 | 0.144060297 | -2.795255314 | 3.71E-92 | 9.20E-91 | Down |
| ATP8B4 | 46.745223 | 1.157468957 | 92.33297704 | 83.13109989 | 6.377316395 | 2.08E-12 | 6.21E-12 | Up |
| ATPAF1 | 489.9923263 | 208.7092055 | 771.2754472 | 3.695279483 | 1.885683483 | 2.12E-40 | 1.71E-39 | Up |
| AVIL | 171.8213936 | 42.4980848 | 301.1447024 | 7.092476336 | 2.826289432 | 2.01E-32 | 1.29E-31 | Up |
| B3GALT4 | 198.9703265 | 310.0279159 | 87.91273701 | 0.283609804 | -1.81802069 | 7.21E-23 | 3.32E-22 | Down |
| B3GNT4 | 28.67091037 | 10.17646702 | 47.16535371 | 4.671375817 | 2.223847516 | 0.0001568 | 0.000292807 | Up |
| B4GALT2 | 1470.470023 | 1965.373569 | 975.5664775 | 0.4964834 | -1.010182613 | 2.41E-34 | 1.63E-33 | Down |
| B4GALT6 | 226.253953 | 332.7262422 | 119.7816637 | 0.359869142 | -1.474455697 | 3.94E-14 | 1.27E-13 | Down |
| B4GAT1 | 600.1296143 | 826.4588028 | 373.8004258 | 0.452207987 | -1.144941622 | 3.61E-23 | 1.69E-22 | Down |
| B9D1 | 29.23345766 | 39.68552203 | 18.78139328 | 0.473721905 | -1.07788771 | 0.021948235 | 0.032332621 | Down |
| BAALC | 33.7238625 | 9.031787602 | 58.41593739 | 6.526318561 | 2.70626941 | 1.11E-06 | 2.40E-06 | Up |
| BAAT | 16.84795961 | 3.30507848 | 30.39084074 | 9.171713028 | 3.197191215 | 2.17E-05 | 4.31E-05 | Up |
| BAHCC1 | 10105.86634 | 18013.59529 | 2198.137396 | 0.122027013 | -3.034727546 | 0 | 0 | Down |
| BAIAP3 | 28.63769139 | 46.51265063 | 10.76273215 | 0.231166235 | -2.112997404 | 7.02E-05 | 0.000135068 | Down |
| BANK1 | 52.6601382 | 4.244052842 | 101.0762236 | 23.92824657 | 4.580642777 | 0.011482308 | 0.017550534 | Up |
| BANP | 1253.766862 | 591.8656909 | 1915.668033 | 3.236413702 | 1.694396035 | 9.16E-80 | 1.83E-78 | Up |
| BASP1 | 9.706396731 | 16.13983552 | 3.27295794 | 0.202752591 | -2.302207747 | 0.007494952 | 0.011745846 | Down |
| BATF | 77.25293273 | 15.7117997 | 138.7940658 | 8.794585125 | 3.136615522 | 1.70E-14 | 5.56E-14 | Up |
| BATF2 | 18.21304051 | 3.284866593 | 33.14121443 | 10.02386967 | 3.325367658 | 1.02E-05 | 2.08E-05 | Up |
| BAX | 2190.955566 | 1168.144656 | 3213.766475 | 2.750282469 | 1.459579799 | 6.88E-90 | 1.63E-88 | Up |
| BAZ2B | 1203.438206 | 682.2419136 | 1724.634498 | 2.528118164 | 1.338063897 | 9.80E-45 | 8.93E-44 | Up |
| BBLN | 1220.025944 | 1644.720115 | 795.3317734 | 0.483554949 | -1.048248254 | 2.64E-29 | 1.54E-28 | Down |
| BBS12 | 189.2127689 | 115.8863134 | 262.5392244 | 2.261144341 | 1.17705309 | 1.08E-06 | 2.35E-06 | Up |
| BCAM | 332.3502195 | 558.0120317 | 106.6884073 | 0.19123816 | -2.386557662 | 4.47E-53 | 5.03E-52 | Down |
| BCAR1 | 15.42727295 | 5.219614811 | 25.63493108 | 4.932178929 | 2.302225139 | 0.003015721 | 0.004954526 | Up |
| BCAS2 | 1997.129634 | 1255.601832 | 2738.657437 | 2.180616214 | 1.124735879 | 1.35E-41 | 1.12E-40 | Up |
| BCAT1 | 7775.491293 | 11206.10968 | 4344.872904 | 0.38772814 | -1.366882648 | 1.02E-80 | 2.08E-79 | Down |
| BCDIN3D | 612.7913838 | 375.4402676 | 850.1424999 | 2.265565456 | 1.179871173 | 3.34E-26 | 1.76E-25 | Up |
| BCL10 | 1239.708125 | 805.1789351 | 1674.237314 | 2.079162555 | 1.056002557 | 3.00E-30 | 1.81E-29 | Up |
| BCL11B | 128.0081048 | 190.9828745 | 65.03333523 | 0.340421611 | -1.55460547 | 2.92E-09 | 7.43E-09 | Down |
| BCL2 | 1913.090448 | 2764.814487 | 1061.366409 | 0.383914395 | -1.381143438 | 1.98E-74 | 3.54E-73 | Down |
| BCL2L1 | 4668.817962 | 6388.1433 | 2949.492624 | 0.461711196 | -1.114937379 | 5.30E-73 | 9.20E-72 | Down |
| BCL3 | 1761.83912 | 305.4977771 | 3218.180462 | 10.53078377 | 3.39654091 | 3.99E-217 | 4.10E-215 | Up |
| BCL6 | 1817.240797 | 779.0447976 | 2855.436796 | 3.665266609 | 1.873918143 | 2.35E-120 | 8.76E-119 | Up |
| BCL7A | 2691.336365 | 3605.558798 | 1777.113932 | 0.492846515 | -1.020789671 | 1.34E-60 | 1.77E-59 | Down |
| BCL7C | 333.6490179 | 445.0843653 | 222.2136704 | 0.499219164 | -1.002254777 | 2.11E-11 | 6.00E-11 | Down |
| BCLAF1 | 7055.956315 | 4570.615539 | 9541.29709 | 2.087519018 | 1.061789341 | 5.76E-37 | 4.24E-36 | Up |
| BCR | 9394.526448 | 13907.21783 | 4881.835071 | 0.351028384 | -1.510340403 | 1.39E-155 | 7.85E-154 | Down |
| BDH1 | 2335.562695 | 3148.458202 | 1522.667187 | 0.483631649 | -1.048019438 | 8.11E-52 | 8.85E-51 | Down |
| BEND3 | 963.0300378 | 1326.523238 | 599.5368377 | 0.452034777 | -1.145494326 | 3.76E-26 | 1.98E-25 | Down |
| BEST1 | 167.3005515 | 104.1501707 | 230.4509323 | 2.209137938 | 1.143483503 | 3.12E-07 | 7.04E-07 | Up |
| BEST2 | 7.90477271 | 2.671337599 | 13.13820782 | 4.987864907 | 2.318422391 | 0.030739913 | 0.044378989 | Up |
| BEST3 | 3028.789791 | 5757.299238 | 300.2803431 | 0.052142592 | -4.26139389 | 0 | 0 | Down |
| BEST4 | 12.4789438 | 4.681042032 | 20.27684556 | 4.290330259 | 2.101088707 | 0.011757349 | 0.017935356 | Up |
| BET1 | 510.5057385 | 338.7397298 | 682.2717473 | 2.015455865 | 1.011106191 | 4.53E-15 | 1.52E-14 | Up |
| BFSP2 | 59.71758881 | 19.58103003 | 99.85414758 | 5.102903499 | 2.351318359 | 5.15E-10 | 1.36E-09 | Up |
| BHLHE40 | 1002.025022 | 103.5382141 | 1900.511829 | 18.37903353 | 4.199988999 | 5.08E-63 | 7.01E-62 | Up |
| BIN2 | 19.26574982 | 3.637688155 | 34.89381148 | 9.516815639 | 3.250478923 | 1.78E-05 | 3.56E-05 | Up |
| BIRC3 | 298.5109926 | 95.8114947 | 501.2104905 | 5.221052699 | 2.384340721 | 1.78E-39 | 1.39E-38 | Up |
| BLNK | 3851.035377 | 5304.336967 | 2397.733787 | 0.452052352 | -1.145438234 | 5.49E-90 | 1.31E-88 | Down |
| BLVRB | 1009.236085 | 1362.260995 | 656.2111757 | 0.481760272 | -1.053612667 | 6.08E-29 | 3.50E-28 | Down |
| BLZF1 | 673.6072648 | 393.4183435 | 953.796186 | 2.42168829 | 1.276013179 | 7.34E-23 | 3.37E-22 | Up |
| BMP4 | 346.836387 | 66.94207191 | 626.7307021 | 9.346353967 | 3.224403676 | 2.20E-78 | 4.29E-77 | Up |
| BMP7 | 52.91778447 | 11.00106826 | 94.83450067 | 8.590171987 | 3.102687016 | 1.06E-13 | 3.37E-13 | Up |
| BMPER | 23.93648226 | 1.493656754 | 46.37930777 | 31.36514949 | 4.971090533 | 2.21E-08 | 5.31E-08 | Up |
| BMS1 | 2662.364595 | 1750.01756 | 3574.71163 | 2.042525575 | 1.030354143 | 7.89E-47 | 7.61E-46 | Up |
| BMT2 | 410.2002501 | 270.0704279 | 550.3300723 | 2.037007815 | 1.026451515 | 8.30E-14 | 2.64E-13 | Up |
| BNIP1 | 1352.713015 | 683.7327744 | 2021.693255 | 2.957709054 | 1.564480143 | 7.71E-63 | 1.06E-61 | Up |
| BNIP3 | 971.6988788 | 577.2305368 | 1366.167221 | 2.36564234 | 1.24223197 | 3.38E-32 | 2.16E-31 | Up |
| BNIP5 | 8.019089743 | 0 | 16.03817949 | 78.77334108 | 6.299635562 | 2.12E-05 | 4.21E-05 | Up |
| BRAF | 3185.524746 | 2003.362549 | 4367.686943 | 2.180411342 | 1.12460033 | 6.42E-58 | 8.03E-57 | Up |
| BRAP | 2616.264207 | 1694.391317 | 3538.137097 | 2.088292326 | 1.06232368 | 2.30E-63 | 3.20E-62 | Up |
| BRF2 | 1412.842872 | 499.0977931 | 2326.58795 | 4.659235459 | 2.22009324 | 1.59E-140 | 7.54E-139 | Up |
| BRI3BP | 6115.039575 | 9065.40351 | 3164.67564 | 0.349086099 | -1.518345186 | 4.53E-162 | 2.71E-160 | Down |
| BRINP2 | 5.959299388 | 0 | 11.91859878 | 58.52249489 | 5.87091937 | 0.000127155 | 0.000239349 | Up |
| BRIP1 | 2167.455864 | 3023.145558 | 1311.766169 | 0.433922759 | -1.20448984 | 4.03E-35 | 2.79E-34 | Down |
| BRME1 | 529.0999969 | 772.8816476 | 285.3183461 | 0.36909155 | -1.437949386 | 2.42E-31 | 1.50E-30 | Down |
| BRPF3 | 2762.597588 | 1630.393539 | 3894.801638 | 2.389169043 | 1.256508934 | 1.08E-72 | 1.86E-71 | Up |
| BSPRY | 2048.069456 | 2777.881859 | 1318.257054 | 0.474563783 | -1.075326092 | 2.01E-44 | 1.82E-43 | Down |
| BST1 | 17.17064775 | 6.283180576 | 28.05811492 | 4.468956322 | 2.159937944 | 0.001540232 | 0.002608615 | Up |
| BTBD19 | 41.41301008 | 21.16832387 | 61.65769629 | 2.920431406 | 1.5461815 | 0.000168883 | 0.000314186 | Up |
| BTBD6 | 282.2906442 | 480.6043689 | 83.97691959 | 0.174797028 | -2.516247439 | 1.80E-48 | 1.81E-47 | Down |
| BTD | 425.3910729 | 652.6499225 | 198.1322232 | 0.303430634 | -1.720561348 | 6.05E-26 | 3.16E-25 | Down |
| BTG1 | 14965.67832 | 5396.914785 | 24534.44186 | 4.54609882 | 2.184629045 | 0 | 0 | Up |
| BTK | 11056.56789 | 17812.5986 | 4300.537171 | 0.241430431 | -2.050320566 | 0 | 0 | Down |
| BTLA | 9.629492303 | 2.19545565 | 17.06352896 | 7.738923784 | 2.952132951 | 0.006032175 | 0.009582261 | Up |
| BTN3A1 | 1364.285645 | 1938.279785 | 790.291504 | 0.40769749 | -1.294429019 | 1.11E-59 | 1.44E-58 | Down |
| BTN3A2 | 1178.473442 | 1579.440076 | 777.5068071 | 0.492291263 | -1.022415958 | 5.96E-31 | 3.65E-30 | Down |
| BTN3A3 | 1035.763247 | 1471.289467 | 600.2370281 | 0.40791041 | -1.293675769 | 1.10E-39 | 8.64E-39 | Down |
| BTNL9 | 246.0678505 | 359.5603735 | 132.5753275 | 0.368633085 | -1.439742537 | 3.12E-16 | 1.10E-15 | Down |
| BUB1B | 6171.33098 | 8320.888477 | 4021.773482 | 0.48334216 | -1.048883255 | 2.15E-57 | 2.65E-56 | Down |
| C10orf143 | 162.4248755 | 94.32525223 | 230.5244988 | 2.442933285 | 1.288614465 | 2.12E-09 | 5.41E-09 | Up |
| C10orf71 | 1.993332744 | 3.986665488 | 0 | 0.041172711 | -4.602167727 | 0.030273481 | 0.043769382 | Down |
| C10orf88 | 814.1710078 | 509.3154247 | 1119.026591 | 2.195701171 | 1.134681721 | 9.04E-29 | 5.17E-28 | Up |
| C11orf96 | 383.1875522 | 250.1560314 | 516.2190731 | 2.065964034 | 1.046815139 | 8.88E-12 | 2.58E-11 | Up |
| C12orf4 | 1522.867118 | 992.5000492 | 2053.234186 | 2.068854925 | 1.048832482 | 2.32E-42 | 1.99E-41 | Up |
| C12orf42 | 44.87445108 | 1.828055491 | 87.92084666 | 47.7222276 | 5.576589482 | 7.48E-14 | 2.39E-13 | Up |
| C12orf50 | 2.686150273 | 0 | 5.372300546 | 26.37442831 | 4.721067917 | 0.012173645 | 0.018543887 | Up |
| C12orf60 | 121.1172781 | 41.83697578 | 200.3975804 | 4.797410999 | 2.262256042 | 6.43E-18 | 2.45E-17 | Up |
| C13orf46 | 14.937197 | 5.496956007 | 24.37743798 | 4.423764354 | 2.145274538 | 0.004933717 | 0.007914374 | Up |
| C14orf132 | 4700.51467 | 7209.141143 | 2191.888197 | 0.304042632 | -1.717654465 | 3.44E-140 | 1.62E-138 | Down |
| C15orf62 | 20.10126104 | 4.512182625 | 35.69033945 | 7.98303949 | 2.996938148 | 8.72E-05 | 0.000166267 | Up |
| C16orf86 | 226.8727711 | 359.1392692 | 94.60627308 | 0.263378755 | -1.924789116 | 2.44E-21 | 1.06E-20 | Down |
| C16orf95 | 112.530339 | 179.6967765 | 45.36390144 | 0.252340976 | -1.986553602 | 9.47E-15 | 3.14E-14 | Down |
| C17orf113 | 21.21780216 | 35.86592689 | 6.569677436 | 0.183095035 | -2.449335428 | 0.00010227 | 0.000193982 | Down |
| C17orf64 | 4.328458448 | 0.356399685 | 8.300517211 | 20.93551071 | 4.387880207 | 0.009743605 | 0.015020398 | Up |
| C17orf67 | 324.4809376 | 439.6191539 | 209.3427213 | 0.476183372 | -1.070410852 | 9.52E-13 | 2.88E-12 | Down |
| C17orf97 | 34.14577873 | 11.44215972 | 56.84939773 | 4.970500148 | 2.313391028 | 1.80E-06 | 3.86E-06 | Up |
| C18orf54 | 792.2131883 | 1228.336595 | 356.0897818 | 0.28988716 | -1.786436664 | 1.59E-56 | 1.94E-55 | Down |
| C19orf12 | 711.067107 | 966.431198 | 455.7030161 | 0.471569331 | -1.084458201 | 5.00E-27 | 2.70E-26 | Down |
| C19orf18 | 34.68482105 | 18.12984416 | 51.23979794 | 2.843219819 | 1.507525643 | 0.006792515 | 0.010705486 | Up |
| C19orf38 | 171.6904219 | 71.69706329 | 271.6837805 | 3.792417476 | 1.923117788 | 1.55E-19 | 6.35E-19 | Up |
| C19orf44 | 422.691916 | 271.5630859 | 573.820746 | 2.113115867 | 1.079371876 | 2.32E-13 | 7.23E-13 | Up |
| C1GALT1 | 898.9327299 | 1314.87464 | 482.9908193 | 0.367333302 | -1.444838399 | 3.42E-32 | 2.18E-31 | Down |
| C1QL1 | 20.02308338 | 34.09135081 | 5.954815946 | 0.174836733 | -2.515919767 | 0.000240647 | 0.000441244 | Down |
| C1QL4 | 8.650200936 | 14.32456957 | 2.975832306 | 0.207744436 | -2.267118256 | 0.013472417 | 0.020381273 | Down |
| C1QTNF12 | 19.70613701 | 28.10930144 | 11.30297258 | 0.402534056 | -1.31281725 | 0.025787451 | 0.037652393 | Down |
| C1QTNF6 | 128.6808319 | 187.8811878 | 69.48047592 | 0.369559555 | -1.436121218 | 9.15E-10 | 2.39E-09 | Down |
| C1R | 132.6678089 | 227.8269346 | 37.5086832 | 0.164758922 | -2.601571505 | 5.60E-21 | 2.40E-20 | Down |
| C1RL | 218.4703924 | 341.6149914 | 95.32579336 | 0.279152573 | -1.840874241 | 5.68E-24 | 2.74E-23 | Down |
| C1orf131 | 1392.51966 | 812.5448946 | 1972.494426 | 2.427561821 | 1.279508036 | 5.14E-43 | 4.47E-42 | Up |
| C1orf159 | 867.2285289 | 1206.578905 | 527.8781527 | 0.437524783 | -1.192563355 | 1.54E-25 | 7.90E-25 | Down |
| C1orf162 | 160.3177284 | 104.2128696 | 216.4225872 | 2.072509301 | 1.051378576 | 1.88E-05 | 3.75E-05 | Up |
| C1orf189 | 11.80062886 | 0.356399685 | 23.24485804 | 58.59888936 | 5.872801416 | 2.10E-05 | 4.18E-05 | Up |
| C1orf21 | 2642.270336 | 536.2637205 | 4748.276951 | 8.856262896 | 3.146698049 | 0 | 0 | Up |
| C1orf216 | 156.6199346 | 233.361735 | 79.87813416 | 0.342336258 | -1.546513993 | 2.44E-10 | 6.58E-10 | Down |
| C2 | 145.5539111 | 80.58478836 | 210.5230339 | 2.610555455 | 1.384356805 | 2.51E-08 | 6.02E-08 | Up |
| C20orf203 | 15.04225055 | 29.49063218 | 0.593868917 | 0.020201677 | -5.629381105 | 2.32E-06 | 4.91E-06 | Down |
| C20orf204 | 37.58133808 | 19.12688292 | 56.03579323 | 2.927172596 | 1.549507814 | 0.000997881 | 0.001723737 | Up |
| C20orf96 | 989.6156619 | 1433.871254 | 545.3600697 | 0.380309049 | -1.394755829 | 8.33E-51 | 8.81E-50 | Down |
| C21orf58 | 2800.265487 | 4955.188745 | 645.3422288 | 0.13025801 | -2.940556003 | 0 | 0 | Down |
| C22orf15 | 78.22290904 | 134.3804957 | 22.06532239 | 0.164190267 | -2.606559485 | 1.61E-16 | 5.75E-16 | Down |
| C22orf23 | 35.08477415 | 23.02938078 | 47.14016752 | 2.05089485 | 1.036253527 | 0.023480787 | 0.034436668 | Up |
| C2CD5 | 1603.772445 | 2179.067333 | 1028.477556 | 0.471977206 | -1.083210909 | 3.19E-31 | 1.97E-30 | Down |
| C2orf68 | 2173.841565 | 3369.99927 | 977.6838607 | 0.290097769 | -1.785388892 | 3.01E-147 | 1.52E-145 | Down |
| C2orf69 | 549.6154334 | 747.7473701 | 351.4834966 | 0.470113827 | -1.088917982 | 6.60E-21 | 2.82E-20 | Down |
| C2orf88 | 24.50021111 | 4.865004187 | 44.13541804 | 9.148019479 | 3.193459438 | 4.36E-07 | 9.74E-07 | Up |
| C3 | 64.02862933 | 23.12660405 | 104.9306546 | 4.526887815 | 2.178519554 | 1.58E-10 | 4.30E-10 | Up |
| C3AR1 | 87.1372304 | 39.18558396 | 135.0888768 | 3.45017395 | 1.786669101 | 1.77E-09 | 4.53E-09 | Up |
| C3orf20 | 232.1139489 | 126.0139194 | 338.2139784 | 2.68571131 | 1.425304236 | 1.62E-13 | 5.08E-13 | Up |
| C3orf52 | 1125.645193 | 518.6613991 | 1732.628988 | 3.338372119 | 1.739144777 | 3.58E-81 | 7.42E-80 | Up |
| C3orf86 | 11.65542477 | 4.818947122 | 18.49190242 | 3.844950113 | 1.94296488 | 0.017612436 | 0.026280347 | Up |
| C4orf19 | 8.030196022 | 1.157468957 | 14.90292309 | 13.2592862 | 3.728931206 | 0.001932143 | 0.003247789 | Up |
| C4orf46 | 2017.513304 | 2706.129992 | 1328.896617 | 0.491034503 | -1.026103694 | 2.94E-36 | 2.10E-35 | Down |
| C4orf47 | 18.66007332 | 0 | 37.32014665 | 183.2138581 | 7.517384821 | 1.01E-08 | 2.48E-08 | Up |
| C4orf54 | 39.2395415 | 71.31992756 | 7.159155439 | 0.100285657 | -3.317812815 | 2.45E-12 | 7.29E-12 | Down |
| C5 | 230.6007934 | 315.7960557 | 145.4055311 | 0.460400805 | -1.119037738 | 8.45E-11 | 2.33E-10 | Down |
| C5AR1 | 10.00206172 | 0.365611098 | 19.63851234 | 49.51538869 | 5.629805059 | 5.95E-05 | 0.000114904 | Up |
| C5orf63 | 118.7444452 | 195.8056658 | 41.68322451 | 0.212958563 | -2.231355352 | 3.58E-18 | 1.38E-17 | Down |
| C6orf52 | 25.08368462 | 13.80673283 | 36.36063641 | 2.644059969 | 1.402754898 | 0.008733275 | 0.013551769 | Up |
| C7orf57 | 66.80040374 | 113.1313243 | 20.46948321 | 0.181161272 | -2.464653518 | 1.66E-08 | 4.03E-08 | Down |
| C8orf76 | 44.38542901 | 28.82415598 | 59.94670204 | 2.080499238 | 1.05692976 | 0.012821698 | 0.019471181 | Up |
| C8orf88 | 4.177072848 | 0 | 8.354145697 | 41.01481256 | 5.35807313 | 0.001314313 | 0.002240762 | Up |
| C9orf47 | 101.4596994 | 151.5854119 | 51.33398694 | 0.338637608 | -1.562185893 | 2.60E-07 | 5.89E-07 | Down |
| C9orf72 | 428.0539597 | 578.6742761 | 277.4336433 | 0.479390853 | -1.060725713 | 7.39E-17 | 2.69E-16 | Down |
| CA2 | 19.32875371 | 29.45225551 | 9.20525191 | 0.313276774 | -1.674490281 | 0.014298702 | 0.021570125 | Down |
| CA5B | 148.9722703 | 198.6862785 | 99.25826215 | 0.499658542 | -1.000985575 | 1.36E-05 | 2.73E-05 | Down |
| CABLES1 | 2812.467391 | 514.6394524 | 5110.29533 | 9.933207281 | 3.312259617 | 0 | 0 | Up |
| CABLES2 | 978.0991885 | 1452.555224 | 503.6431528 | 0.346775857 | -1.527924635 | 1.85E-46 | 1.77E-45 | Down |
| CABP1 | 19.77824572 | 30.57288688 | 8.983604565 | 0.293418628 | -1.768967631 | 0.009385131 | 0.014500017 | Down |
| CABYR | 13.74108765 | 6.279602453 | 21.20257284 | 3.377274105 | 1.755859276 | 0.015785537 | 0.023699663 | Up |
| CACNA1A | 179.5773515 | 105.942953 | 253.2117501 | 2.392618039 | 1.258590101 | 1.21E-08 | 2.96E-08 | Up |
| CACNA1B | 8.100557774 | 0.385822986 | 15.81529256 | 39.86379254 | 5.317007066 | 0.000220752 | 0.000406053 | Up |
| CACNA1E | 8.067255807 | 0.385822986 | 15.74868863 | 39.7127724 | 5.311531176 | 0.000293287 | 0.000533532 | Up |
| CACNA2D3 | 402.7415048 | 543.7580741 | 261.7249355 | 0.481160207 | -1.055410762 | 1.11E-14 | 3.66E-14 | Down |
| CACNB4 | 203.3414357 | 286.3253773 | 120.357494 | 0.420270817 | -1.250608814 | 1.75E-09 | 4.49E-09 | Down |
| CACNG8 | 10.22584417 | 20.45168833 | 0 | 0.00803349 | -6.959757484 | 1.09E-06 | 2.37E-06 | Down |
| CAGE1 | 3.286460649 | 0 | 6.572921298 | 32.26582399 | 5.011934964 | 0.005551947 | 0.008854898 | Up |
| CALCB | 25.10070914 | 7.280743494 | 42.92067479 | 5.856075384 | 2.549934125 | 9.07E-06 | 1.85E-05 | Up |
| CALHM6 | 74.03264493 | 104.5677302 | 43.4975597 | 0.415891951 | -1.265719332 | 1.72E-05 | 3.45E-05 | Down |
| CALM3 | 17664.62227 | 25384.22315 | 9945.021392 | 0.391783036 | -1.351873162 | 6.00E-125 | 2.39E-123 | Down |
| CALML4 | 520.0888117 | 708.5932393 | 331.5843841 | 0.467863747 | -1.095839652 | 1.42E-16 | 5.10E-16 | Down |
| CALR3 | 3.586447794 | 0 | 7.172895588 | 35.20965733 | 5.137899281 | 0.003364281 | 0.005492696 | Up |
| CAMK1G | 6.716044796 | 0 | 13.43208959 | 65.94276667 | 6.043142512 | 6.81E-05 | 0.000131045 | Up |
| CAMK2B | 44.19830575 | 71.72954056 | 16.66707095 | 0.232392266 | -2.105366035 | 6.98E-06 | 1.44E-05 | Down |
| CAMK2D | 690.8127233 | 100.2947701 | 1281.330677 | 12.80130301 | 3.678218761 | 2.30E-162 | 1.38E-160 | Up |
| CAMK2G | 2591.766968 | 3714.638769 | 1468.895167 | 0.395415318 | -1.338559334 | 2.24E-98 | 5.95E-97 | Down |
| CAMK2N2 | 138.5856999 | 190.1086219 | 87.06277785 | 0.458262619 | -1.125753487 | 1.58E-06 | 3.39E-06 | Down |
| CAMKK1 | 153.0156705 | 64.71387288 | 241.317468 | 3.731981529 | 1.899941846 | 1.76E-15 | 6.02E-15 | Up |
| CAMKK2 | 2663.482932 | 4147.095065 | 1179.8708 | 0.284493224 | -1.813533802 | 7.24E-155 | 4.07E-153 | Down |
| CAMKV | 54.3230005 | 102.9753352 | 5.67066576 | 0.055001461 | -4.184386259 | 5.64E-18 | 2.16E-17 | Down |
| CAP2 | 330.1858812 | 217.215484 | 443.1562784 | 2.039109225 | 1.027939055 | 1.54E-11 | 4.42E-11 | Up |
| CAPG | 289.5419214 | 397.2415249 | 181.8423179 | 0.457428713 | -1.128381167 | 4.94E-10 | 1.31E-09 | Down |
| CAPN13 | 54.32481632 | 34.49149435 | 74.1581383 | 2.146751327 | 1.102155083 | 0.002310738 | 0.00385012 | Up |
| CAPN14 | 15.5479564 | 0.71279937 | 30.38311343 | 41.38459626 | 5.371021978 | 6.13E-06 | 1.27E-05 | Up |
| CAPN3 | 838.0826746 | 547.3760826 | 1128.789267 | 2.061226715 | 1.043503196 | 4.86E-20 | 2.01E-19 | Up |
| CAPRIN2 | 553.7131891 | 760.6501599 | 346.7762184 | 0.455842737 | -1.133391906 | 4.74E-15 | 1.59E-14 | Down |
| CAPS | 130.3340318 | 213.8932476 | 46.77481602 | 0.218621606 | -2.193492108 | 1.97E-16 | 7.01E-16 | Down |
| CAPSL | 16.46890366 | 24.6110494 | 8.326757928 | 0.338346724 | -1.563425676 | 0.020179821 | 0.029886216 | Down |
| CARD11 | 8.977200201 | 15.57005038 | 2.384350022 | 0.152796212 | -2.710319314 | 0.007732727 | 0.012095248 | Down |
| CARD14 | 58.86481959 | 101.6362093 | 16.0934299 | 0.158396113 | -2.658391159 | 5.62E-13 | 1.72E-12 | Down |
| CARD6 | 78.35048778 | 10.11019807 | 146.5907775 | 14.63994716 | 3.871838442 | 3.76E-21 | 1.62E-20 | Up |
| CARD9 | 39.24315825 | 53.83560696 | 24.65070953 | 0.458216704 | -1.125898043 | 0.016944387 | 0.025338032 | Down |
| CARF | 406.3314572 | 563.6796361 | 248.9832782 | 0.441715169 | -1.178811717 | 7.56E-17 | 2.74E-16 | Down |
| CARHSP1 | 5001.124705 | 7876.975852 | 2125.273558 | 0.269801243 | -1.890031102 | 1.93E-235 | 2.24E-233 | Down |
| CARMIL3 | 196.2918733 | 310.1113509 | 82.47239566 | 0.266049031 | -1.910235945 | 6.81E-18 | 2.60E-17 | Down |
| CASK | 1107.725543 | 1510.383629 | 705.0674563 | 0.466814267 | -1.099079441 | 3.66E-29 | 2.12E-28 | Down |
| CASP7 | 3987.883487 | 2005.689727 | 5970.077246 | 2.976181037 | 1.573462286 | 1.31E-154 | 7.28E-153 | Up |
| CASS4 | 220.1219403 | 18.50798675 | 421.7358939 | 22.8476717 | 4.513975249 | 5.32E-66 | 7.89E-65 | Up |
| CASTOR2 | 1049.483514 | 1413.858352 | 685.1086752 | 0.484522129 | -1.045365538 | 3.78E-32 | 2.40E-31 | Down |
| CATSPER2 | 210.426311 | 319.4885296 | 101.3640925 | 0.31733983 | -1.655899487 | 1.56E-14 | 5.10E-14 | Down |
| CATSPER3 | 64.9604691 | 41.72233655 | 88.19860166 | 2.10953734 | 1.076926625 | 0.002238997 | 0.003736869 | Up |
| CATSPERE | 40.79914399 | 15.59973173 | 65.99855626 | 4.241191649 | 2.084469676 | 8.11E-06 | 1.66E-05 | Up |
| CATSPERZ | 30.45492493 | 17.07880988 | 43.83103998 | 2.571924507 | 1.362848296 | 0.004731544 | 0.00760325 | Up |
| CBARP | 48.47214654 | 30.78448327 | 66.15980981 | 2.152419054 | 1.105958983 | 0.006081063 | 0.009656602 | Up |
| CBFA2T3 | 564.405145 | 855.230196 | 273.5800939 | 0.319881416 | -1.644390916 | 9.65E-34 | 6.37E-33 | Down |
| CBLN3 | 169.9244476 | 104.8473765 | 235.0015187 | 2.243903993 | 1.166010951 | 1.87E-07 | 4.29E-07 | Up |
| CBR4 | 671.1350257 | 962.6753005 | 379.5947509 | 0.394321616 | -1.342555298 | 2.08E-36 | 1.49E-35 | Down |
| CBS | 257.9297631 | 490.508363 | 25.35116325 | 0.051686003 | -4.274082547 | 9.88E-76 | 1.81E-74 | Down |
| CBSL | 107.8251804 | 207.8807347 | 7.769626015 | 0.037347033 | -4.742862569 | 5.94E-10 | 1.57E-09 | Down |
| CBX2 | 3093.263196 | 4678.408741 | 1508.117651 | 0.322358147 | -1.63326365 | 2.64E-117 | 9.41E-116 | Down |
| CBX5 | 19978.16072 | 29583.40662 | 10372.91482 | 0.350639931 | -1.511937794 | 1.43E-227 | 1.59E-225 | Down |
| CBX7 | 321.7921885 | 551.3729502 | 92.21142677 | 0.167124564 | -2.581004301 | 3.46E-49 | 3.54E-48 | Down |
| CCAR1 | 7495.672593 | 4635.249179 | 10356.09601 | 2.234187412 | 1.15975021 | 2.11E-44 | 1.90E-43 | Up |
| CCBE1 | 11.57861165 | 18.37392589 | 4.783297422 | 0.25994496 | -1.943721913 | 0.022453954 | 0.0330145 | Down |
| CCDC102A | 200.800879 | 119.079582 | 282.522176 | 2.369847365 | 1.244794142 | 7.37E-11 | 2.04E-10 | Up |
| CCDC103 | 51.01922716 | 10.77899554 | 91.25945878 | 8.501988529 | 3.087800312 | 1.30E-12 | 3.92E-12 | Up |
| CCDC106 | 1214.469334 | 1620.739294 | 808.1993739 | 0.498653765 | -1.003889652 | 4.62E-33 | 3.00E-32 | Down |
| CCDC112 | 211.1898189 | 330.9511178 | 91.42851992 | 0.276296535 | -1.855710627 | 3.38E-19 | 1.36E-18 | Down |
| CCDC113 | 114.9814254 | 74.87728426 | 155.0855665 | 2.074266556 | 1.052601301 | 7.48E-05 | 0.000143597 | Up |
| CCDC121 | 225.5342021 | 358.9263503 | 92.14205385 | 0.256677497 | -1.961971273 | 1.42E-27 | 7.84E-27 | Down |
| CCDC13 | 18.76637672 | 5.101921609 | 32.43083183 | 6.320160377 | 2.659961168 | 0.000164858 | 0.000307193 | Up |
| CCDC134 | 1746.969875 | 2407.570889 | 1086.368862 | 0.451218002 | -1.148103468 | 4.49E-54 | 5.21E-53 | Down |
| CCDC137 | 3895.95115 | 2459.150962 | 5332.751337 | 2.168660884 | 1.116804475 | 2.42E-60 | 3.17E-59 | Up |
| CCDC14 | 6198.463565 | 8534.979139 | 3861.94799 | 0.452488982 | -1.144045431 | 9.34E-38 | 7.01E-37 | Down |
| CCDC153 | 26.49837888 | 37.7858304 | 15.21092736 | 0.402801928 | -1.311857508 | 0.007125002 | 0.011202777 | Down |
| CCDC154 | 116.8578393 | 47.90855996 | 185.8071186 | 3.877442657 | 1.955105446 | 7.69E-14 | 2.45E-13 | Up |
| CCDC157 | 302.5427386 | 150.1214445 | 454.9640327 | 3.033035769 | 1.600762512 | 1.43E-22 | 6.52E-22 | Up |
| CCDC159 | 66.30713699 | 98.86049217 | 33.75378182 | 0.341401388 | -1.550459172 | 1.69E-05 | 3.39E-05 | Down |
| CCDC163 | 432.9617031 | 605.2663727 | 260.6570335 | 0.430787302 | -1.214952371 | 5.20E-20 | 2.15E-19 | Down |
| CCDC173 | 24.39422415 | 14.4755303 | 34.31291801 | 2.37434764 | 1.247531182 | 0.028228554 | 0.040996573 | Up |
| CCDC188 | 31.43030392 | 61.66924379 | 1.191364046 | 0.019319481 | -5.693799847 | 1.80E-11 | 5.14E-11 | Down |
| CCDC189 | 179.3523157 | 268.3726144 | 90.33201705 | 0.336848027 | -1.569830245 | 1.06E-10 | 2.91E-10 | Down |
| CCDC194 | 12.56713032 | 19.46359487 | 5.67066576 | 0.291838021 | -1.776760241 | 0.024129025 | 0.035346257 | Down |
| CCDC3 | 51.45668016 | 18.58167805 | 84.33168227 | 4.552859773 | 2.186773025 | 5.01E-08 | 1.18E-07 | Up |
| CCDC30 | 65.27765792 | 21.36481752 | 109.1904983 | 5.106895015 | 2.352446401 | 4.34E-11 | 1.22E-10 | Up |
| CCDC61 | 344.2817347 | 560.3584722 | 128.2049972 | 0.228750266 | -2.128154674 | 2.03E-40 | 1.64E-39 | Down |
| CCDC65 | 32.67270136 | 7.253109255 | 58.09229346 | 7.939472904 | 2.989043231 | 8.97E-08 | 2.09E-07 | Up |
| CCDC69 | 4909.41057 | 6661.981734 | 3156.839406 | 0.473855648 | -1.077480461 | 5.17E-69 | 8.22E-68 | Down |
| CCDC78 | 133.2965398 | 216.3230909 | 50.26998871 | 0.232512531 | -2.104619625 | 2.12E-16 | 7.52E-16 | Down |
| CCDC85A | 4.186485424 | 8.072601353 | 0.300369495 | 0.039658112 | -4.656240198 | 0.004690855 | 0.007540486 | Down |
| CCDC85B | 1814.258412 | 2464.537349 | 1163.979476 | 0.472320386 | -1.082162288 | 1.83E-39 | 1.43E-38 | Down |
| CCDC86 | 3457.336827 | 2186.344934 | 4728.328719 | 2.16314373 | 1.113129528 | 8.73E-55 | 1.03E-53 | Up |
| CCDC9B | 122.5674658 | 180.6229533 | 64.51197833 | 0.357142499 | -1.485428275 | 7.30E-08 | 1.71E-07 | Down |
| CCER2 | 64.7690958 | 96.69572473 | 32.84246686 | 0.339510621 | -1.558471388 | 8.45E-06 | 1.73E-05 | Down |
| CCL24 | 2.82059059 | 0 | 5.641181181 | 27.70864135 | 4.792264067 | 0.010948423 | 0.016782582 | Up |
| CCL3 | 55.05972573 | 0.365611098 | 109.7538404 | 276.6608536 | 8.111974713 | 3.21E-11 | 9.03E-11 | Up |
| CCL4 | 143.1941995 | 1.899691627 | 284.4887074 | 154.019923 | 7.26697317 | 9.27E-27 | 4.98E-26 | Up |
| CCL4L2 | 36.96443421 | 0 | 73.92886841 | 362.9884701 | 8.503779913 | 1.11E-11 | 3.20E-11 | Up |
| CCM2L | 545.3048227 | 936.5644864 | 154.045159 | 0.164510416 | -2.603749161 | 1.89E-93 | 4.80E-92 | Down |
| CCND3 | 47149.2797 | 87694.19734 | 6604.362072 | 0.075311336 | -3.730989156 | 0 | 0 | Down |
| CCNE1 | 402.8236155 | 643.5073652 | 162.1398659 | 0.25195572 | -1.988757888 | 2.68E-48 | 2.69E-47 | Down |
| CCNE2 | 945.9554686 | 1378.799701 | 513.1112358 | 0.372184495 | -1.42591014 | 5.51E-33 | 3.56E-32 | Down |
| CCNL1 | 6682.358805 | 4171.93491 | 9192.782701 | 2.203539442 | 1.13982272 | 3.45E-83 | 7.48E-82 | Up |
| CCNP | 35.25060863 | 15.65268699 | 54.84853027 | 3.487138161 | 1.802043525 | 0.000177583 | 0.000329445 | Up |
| CCPG1 | 902.9010764 | 1211.987034 | 593.8151191 | 0.489879748 | -1.029500445 | 2.46E-18 | 9.59E-18 | Down |
| CCR4 | 8.504376709 | 0 | 17.00875342 | 83.5042622 | 6.383777932 | 1.14E-05 | 2.32E-05 | Up |
| CCR7 | 236.2355818 | 15.75427058 | 456.7168931 | 29.36182082 | 4.875869532 | 6.03E-65 | 8.71E-64 | Up |
| CCT6B | 199.6926131 | 101.953442 | 297.4317841 | 2.918815544 | 1.545383042 | 1.29E-14 | 4.25E-14 | Up |
| CD163 | 7.417300542 | 1.137257069 | 13.69734401 | 12.26090508 | 3.615993575 | 0.002888379 | 0.004755454 | Up |
| CD1A | 26.36753258 | 0 | 52.73506517 | 258.9415041 | 8.016482414 | 2.43E-10 | 6.55E-10 | Up |
| CD1C | 159.7639252 | 2.166032349 | 317.3618181 | 143.5502429 | 7.165411961 | 1.86E-30 | 1.13E-29 | Up |
| CD200 | 73.97328817 | 46.04776916 | 101.8988072 | 2.212003671 | 1.14535378 | 0.000186381 | 0.000345212 | Up |
| CD200R1 | 234.3508256 | 30.56367546 | 438.1379758 | 14.31838645 | 3.839797018 | 2.47E-65 | 3.58E-64 | Up |
| CD209 | 24.37396235 | 0.722010783 | 48.02591393 | 65.23136088 | 6.027493822 | 1.14E-07 | 2.64E-07 | Up |
| CD22 | 3978.464608 | 2207.984878 | 5748.944339 | 2.604089706 | 1.380779148 | 1.28E-99 | 3.49E-98 | Up |
| CD24 | 9155.779771 | 15466.69912 | 2844.860418 | 0.183935336 | -2.442729428 | 0 | 0 | Down |
| CD28 | 122.3845887 | 1.543291942 | 243.2258854 | 164.5255131 | 7.362167511 | 1.58E-22 | 7.15E-22 | Up |
| CD300A | 2.685530483 | 0 | 5.371060966 | 26.36903209 | 4.720772711 | 0.012584799 | 0.019140762 | Up |
| CD300LB | 2.678660409 | 0 | 5.357320817 | 26.30787722 | 4.717422937 | 0.016421693 | 0.024596189 | Up |
| CD38 | 5823.74118 | 8701.488941 | 2945.99342 | 0.338556452 | -1.562531683 | 2.42E-151 | 1.29E-149 | Down |
| CD4 | 7.540520946 | 14.18155534 | 0.899486555 | 0.063277684 | -3.982159381 | 0.001108486 | 0.001904833 | Down |
| CD40 | 183.262902 | 56.33450703 | 310.1912969 | 5.517172952 | 2.463929207 | 1.10E-27 | 6.08E-27 | Up |
| CD44 | 243.9775222 | 130.5624235 | 357.3926209 | 2.737204065 | 1.452702995 | 1.68E-16 | 5.98E-16 | Up |
| CD47 | 8373.747437 | 11470.18969 | 5277.305183 | 0.460096088 | -1.119992906 | 1.24E-90 | 2.98E-89 | Down |
| CD48 | 1163.435993 | 1694.841113 | 632.030873 | 0.372970746 | -1.422865619 | 1.25E-55 | 1.49E-54 | Down |
| CD52 | 158.0187631 | 262.3557412 | 53.68178501 | 0.204579104 | -2.289269299 | 3.95E-25 | 1.99E-24 | Down |
| CD69 | 2388.296713 | 175.1659131 | 4601.427513 | 26.23536697 | 4.713441065 | 0 | 0 | Up |
| CD70 | 310.7784622 | 75.83084528 | 545.7260792 | 7.206694816 | 2.849337752 | 4.78E-58 | 5.99E-57 | Up |
| CD74 | 85171.58023 | 136685.5368 | 33657.62362 | 0.246241441 | -2.021854514 | 0 | 0 | Down |
| CD79B | 12859.49702 | 21426.60468 | 4292.389369 | 0.200328418 | -2.319561 | 0 | 0 | Down |
| CD80 | 66.09908804 | 1.868479266 | 130.3296968 | 70.57080767 | 6.140999616 | 2.02E-17 | 7.53E-17 | Up |
| CD81 | 10803.44335 | 14735.05648 | 6871.830222 | 0.466364768 | -1.100469293 | 5.51E-69 | 8.74E-68 | Down |
| CD82 | 1498.558334 | 700.6350364 | 2296.481631 | 3.278888768 | 1.713206961 | 5.86E-71 | 9.77E-70 | Up |
| CD83 | 2079.470811 | 953.4398803 | 3205.501741 | 3.363683919 | 1.750042144 | 4.53E-101 | 1.27E-99 | Up |
| CD86 | 1478.256298 | 12.72985337 | 2943.782743 | 228.1535452 | 7.833861261 | 1.81E-206 | 1.65E-204 | Up |
| CD9 | 20489.66151 | 34707.08304 | 6272.239974 | 0.180718198 | -2.468186302 | 0 | 0 | Down |
| CD96 | 808.2431146 | 361.7635348 | 1254.722694 | 3.472505702 | 1.795977063 | 1.92E-47 | 1.89E-46 | Up |
| CD99L2 | 951.5708429 | 1286.940123 | 616.2015627 | 0.478907076 | -1.062182343 | 6.92E-22 | 3.07E-21 | Down |
| CDC20B | 60.66145216 | 6.707638275 | 114.615266 | 17.21176548 | 4.105323183 | 2.68E-18 | 1.04E-17 | Up |
| CDC25B | 5197.405075 | 7937.870904 | 2456.939246 | 0.30952485 | -1.691872856 | 6.23E-187 | 4.73E-185 | Down |
| CDC25C | 896.4564826 | 1203.478926 | 589.4340395 | 0.489781497 | -1.029789821 | 2.33E-26 | 1.24E-25 | Down |
| CDC42EP1 | 59.12168225 | 10.62445669 | 107.6189078 | 10.0868547 | 3.334404477 | 5.82E-16 | 2.03E-15 | Up |
| CDC45 | 3624.673671 | 5054.864127 | 2194.483215 | 0.434138321 | -1.203773322 | 8.71E-93 | 2.18E-91 | Down |
| CDCA7 | 6540.081735 | 9900.685067 | 3179.478403 | 0.321134736 | -1.638749371 | 4.44E-217 | 4.53E-215 | Down |
| CDCA7L | 8317.094003 | 11710.09264 | 4924.095364 | 0.420512516 | -1.249779354 | 1.39E-110 | 4.60E-109 | Down |
| CDH18 | 10.99873944 | 0 | 21.99747888 | 108.035369 | 6.755359894 | 1.38E-06 | 2.97E-06 | Up |
| CDH26 | 23.68733869 | 36.01483245 | 11.35984493 | 0.315445942 | -1.664535302 | 0.003068566 | 0.005031215 | Down |
| CDH4 | 32.63055016 | 1.117045182 | 64.14405514 | 57.89610432 | 5.855394371 | 3.77E-10 | 1.01E-09 | Up |
| CDH5 | 4.379638927 | 0.722010783 | 8.03726707 | 10.97588074 | 3.456264805 | 0.020800112 | 0.030745775 | Up |
| CDK14 | 3038.518181 | 1661.586353 | 4415.45001 | 2.657140495 | 1.409874512 | 4.35E-82 | 9.14E-81 | Up |
| CDK15 | 4.928616377 | 0 | 9.857232754 | 48.38895086 | 5.596605755 | 0.000531043 | 0.000939914 | Up |
| CDK18 | 1921.966254 | 2792.039359 | 1051.893148 | 0.376797232 | -1.408139728 | 8.84E-77 | 1.66E-75 | Down |
| CDK2 | 5382.209339 | 7563.488537 | 3200.93014 | 0.423225122 | -1.24050283 | 3.79E-112 | 1.28E-110 | Down |
| CDK20 | 689.2628413 | 979.427907 | 399.0977755 | 0.407352553 | -1.295650144 | 1.89E-28 | 1.07E-27 | Down |
| CDK7 | 1289.479084 | 814.6047088 | 1764.35346 | 2.165259948 | 1.114540237 | 1.98E-39 | 1.55E-38 | Up |
| CDKL3 | 185.1543948 | 118.3069291 | 252.0018604 | 2.131308554 | 1.091739471 | 6.63E-08 | 1.55E-07 | Up |
| CDKL4 | 3.858954641 | 0 | 7.717909283 | 37.90940443 | 5.244483886 | 0.002625395 | 0.004344132 | Up |
| CDKN1A | 14449.95708 | 4953.541284 | 23946.37287 | 4.834898412 | 2.273485577 | 1.17E-307 | 2.09E-305 | Up |
| CDKN1C | 239.4968736 | 448.5726571 | 30.42109002 | 0.06781842 | -3.882179013 | 3.27E-69 | 5.23E-68 | Down |
| CDKN2AIP | 2294.93323 | 1253.732903 | 3336.133556 | 2.661419112 | 1.412195719 | 1.29E-73 | 2.26E-72 | Up |
| CDKN2C | 4392.363804 | 6011.792018 | 2772.935591 | 0.461303204 | -1.116212783 | 1.59E-76 | 2.98E-75 | Down |
| CDON | 474.52623 | 683.1838924 | 265.8685676 | 0.38907067 | -1.361895869 | 7.60E-27 | 4.09E-26 | Down |
| CDPF1 | 371.1270777 | 524.0795386 | 218.1746168 | 0.41629522 | -1.2643211 | 7.16E-19 | 2.85E-18 | Down |
| CDRT1 | 12.84750953 | 5.149767736 | 20.54525132 | 3.985291619 | 1.994685296 | 0.016928695 | 0.025317296 | Up |
| CDT1 | 6202.999433 | 9066.531808 | 3339.467058 | 0.368334845 | -1.440910211 | 1.04E-102 | 3.01E-101 | Down |
| CEACAM21 | 1047.604617 | 669.4168679 | 1425.792366 | 2.131081984 | 1.091586096 | 2.04E-28 | 1.16E-27 | Up |
| CEBPZ | 2332.139185 | 1431.748757 | 3232.529614 | 2.257864683 | 1.174959026 | 6.35E-37 | 4.67E-36 | Up |
| CEBPZOS | 2412.064614 | 3246.978887 | 1577.150341 | 0.485724576 | -1.04178961 | 3.79E-38 | 2.87E-37 | Down |
| CECR2 | 1006.491769 | 1569.616189 | 443.3673491 | 0.282489639 | -1.823730141 | 4.15E-84 | 9.13E-83 | Down |
| CELF6 | 221.798087 | 110.2837049 | 333.3124691 | 3.022131896 | 1.595566626 | 1.72E-15 | 5.90E-15 | Up |
| CELSR1 | 31.54283105 | 13.538345 | 49.5473171 | 3.647924083 | 1.867075706 | 0.000258836 | 0.000473532 | Up |
| CELSR2 | 4135.517703 | 6200.393036 | 2070.64237 | 0.333963256 | -1.582238716 | 7.94E-89 | 1.85E-87 | Down |
| CENPH | 2294.872426 | 3470.096871 | 1119.64798 | 0.322679914 | -1.631824319 | 1.75E-96 | 4.56E-95 | Down |
| CENPK | 1310.820406 | 1812.805936 | 808.8348749 | 0.446135412 | -1.16444643 | 5.48E-26 | 2.86E-25 | Down |
| CENPP | 499.1686332 | 667.5013075 | 330.8359589 | 0.495617057 | -1.012702256 | 3.11E-18 | 1.21E-17 | Down |
| CENPS-CORT | 92.37388252 | 60.54145617 | 124.2063089 | 2.051553562 | 1.036716821 | 0.000467581 | 0.00083269 | Up |
| CENPV | 820.1022434 | 1147.188762 | 493.0157253 | 0.429911226 | -1.217889313 | 3.15E-27 | 1.72E-26 | Down |
| CEP295NL | 11.412687 | 19.24842035 | 3.576953648 | 0.185935804 | -2.427123494 | 0.001923125 | 0.003233023 | Down |
| CEP57L1 | 715.4890427 | 979.9385553 | 451.0395301 | 0.460247649 | -1.119517743 | 1.76E-20 | 7.42E-20 | Down |
| CEP70 | 917.3440296 | 1353.154177 | 481.5338825 | 0.355860913 | -1.490614615 | 4.33E-31 | 2.65E-30 | Down |
| CEP78 | 1783.081676 | 2449.953568 | 1116.209784 | 0.455573407 | -1.134244559 | 2.95E-49 | 3.02E-48 | Down |
| CERS3 | 8.923523822 | 1.502868167 | 16.34417948 | 11.00218674 | 3.45971839 | 0.001571326 | 0.002659978 | Up |
| CES3 | 208.1000886 | 288.5692033 | 127.6309738 | 0.442157991 | -1.177366131 | 4.97E-11 | 1.39E-10 | Down |
| CES4A | 5.155281802 | 9.716694688 | 0.593868917 | 0.061093545 | -4.032836223 | 0.009069085 | 0.014039874 | Down |
| CETN3 | 687.5033772 | 924.0922036 | 450.9145509 | 0.487816049 | -1.035590871 | 2.52E-19 | 1.02E-18 | Down |
| CETP | 46.94995998 | 22.26847724 | 71.63144273 | 3.204655159 | 1.680169122 | 5.75E-05 | 0.0001112 | Up |
| CFAP161 | 3.886771025 | 0 | 7.77354205 | 38.15885724 | 5.253946062 | 0.003915763 | 0.006354183 | Up |
| CFAP206 | 24.2813817 | 7.688567428 | 40.87419597 | 5.297623409 | 2.405345291 | 4.76E-05 | 9.24E-05 | Up |
| CFAP20DC | 22.24318528 | 5.511534605 | 38.97483596 | 7.060005352 | 2.819669277 | 3.42E-05 | 6.71E-05 | Up |
| CFAP251 | 225.7896984 | 67.10019695 | 384.4791999 | 5.717003375 | 2.515259142 | 5.57E-28 | 3.10E-27 | Up |
| CFAP36 | 213.5484517 | 141.2035059 | 285.8933975 | 2.025960312 | 1.018605913 | 5.44E-07 | 1.20E-06 | Up |
| CFAP43 | 50.23355198 | 17.84814264 | 82.61896132 | 4.607094061 | 2.203857053 | 6.88E-07 | 1.51E-06 | Up |
| CFAP46 | 8.370858202 | 2.13839811 | 14.60331829 | 6.720022255 | 2.748466011 | 0.012460492 | 0.018964174 | Up |
| CFAP53 | 40.3275504 | 7.67219977 | 72.98290103 | 9.46281909 | 3.242270044 | 9.36E-10 | 2.45E-09 | Up |
| CFAP57 | 5.524640216 | 1.493656754 | 9.555623679 | 6.439303504 | 2.68690465 | 0.024715521 | 0.036159582 | Up |
| CFAP58 | 117.6942781 | 169.8406538 | 65.54790236 | 0.386041386 | -1.373172573 | 1.29E-08 | 3.16E-08 | Down |
| CFAP65 | 2.246092317 | 0 | 4.492184633 | 22.04699165 | 4.462509906 | 0.030344341 | 0.043854542 | Up |
| CFAP70 | 237.1066729 | 130.093972 | 344.1193737 | 2.642255854 | 1.401770172 | 8.46E-14 | 2.69E-13 | Up |
| CFAP73 | 153.6899698 | 258.8101591 | 48.56978057 | 0.187742009 | -2.413176593 | 1.88E-23 | 8.88E-23 | Down |
| CFAP74 | 2.438002331 | 4.876004662 | 0 | 0.033800028 | -4.886831769 | 0.013417726 | 0.020309611 | Down |
| CFAP97D1 | 5.749088799 | 10.31769219 | 1.180485408 | 0.114966721 | -3.120711787 | 0.015184657 | 0.022846988 | Down |
| CFHR1 | 3.299818447 | 0 | 6.599636894 | 32.38834751 | 5.017402957 | 0.009665348 | 0.014914678 | Up |
| CGNL1 | 45.17727571 | 10.23300041 | 80.12155101 | 7.798514785 | 2.963199391 | 1.29E-08 | 3.14E-08 | Up |
| CGRRF1 | 699.3779585 | 405.8775212 | 992.8783959 | 2.445140737 | 1.289917506 | 4.79E-33 | 3.10E-32 | Up |
| CHAC1 | 667.3305125 | 1204.958479 | 129.7025463 | 0.107621546 | -3.215961161 | 3.37E-145 | 1.67E-143 | Down |
| CHAF1B | 2181.441378 | 3096.966997 | 1265.915758 | 0.408735513 | -1.290760497 | 1.22E-71 | 2.05E-70 | Down |
| CHCHD10 | 523.5751211 | 763.4615325 | 283.6887097 | 0.371746731 | -1.427608037 | 2.43E-19 | 9.84E-19 | Down |
| CHD2 | 12401.77933 | 7682.825802 | 17120.73286 | 2.228356963 | 1.155980358 | 1.34E-84 | 2.99E-83 | Up |
| CHD5 | 149.4562036 | 72.48918725 | 226.42322 | 3.12615022 | 1.644387105 | 2.30E-11 | 6.52E-11 | Up |
| CHEK2 | 860.7438583 | 1352.326015 | 369.161702 | 0.272957479 | -1.87325187 | 2.33E-79 | 4.62E-78 | Down |
| CHFR | 358.0824457 | 215.0896669 | 501.0752245 | 2.330644949 | 1.220729241 | 7.24E-14 | 2.31E-13 | Up |
| CHIC2 | 894.2783035 | 568.5653602 | 1219.991247 | 2.147029391 | 1.102341941 | 8.36E-24 | 4.02E-23 | Up |
| CHL1 | 17.74689288 | 0 | 35.49378577 | 174.2630108 | 7.445122565 | 1.23E-08 | 3.02E-08 | Up |
| CHMP4A | 2457.127207 | 1315.635818 | 3598.618595 | 2.735568002 | 1.451840419 | 3.87E-76 | 7.18E-75 | Up |
| CHORDC1 | 1445.519928 | 784.3074316 | 2106.732425 | 2.685866896 | 1.425387811 | 3.43E-50 | 3.58E-49 | Up |
| CHRDL1 | 55.7752132 | 36.18574703 | 75.36467937 | 2.083459062 | 1.058980753 | 0.003078045 | 0.005045824 | Up |
| CHRM4 | 30.76450908 | 19.23741988 | 42.29159827 | 2.200846127 | 1.138058282 | 0.013063051 | 0.019816009 | Up |
| CHRNA10 | 45.8421214 | 19.57181862 | 72.11242418 | 3.68620067 | 1.882134611 | 2.28E-06 | 4.84E-06 | Up |
| CHRNA5 | 235.3386597 | 329.28965 | 141.3876694 | 0.429418198 | -1.219544763 | 5.65E-12 | 1.66E-11 | Down |
| CHRNA6 | 27.04742088 | 0.722010783 | 53.37283098 | 72.47380477 | 6.179387731 | 3.35E-08 | 7.99E-08 | Up |
| CHRNA9 | 15.12557372 | 3.749748067 | 26.50139938 | 7.130888933 | 2.834081934 | 0.000234564 | 0.000430392 | Up |
| CHRNB1 | 477.502465 | 681.9832199 | 273.0217102 | 0.400225225 | -1.321115997 | 1.07E-26 | 5.74E-26 | Down |
| CHRNB2 | 26.32776667 | 15.05963599 | 37.59589736 | 2.492640165 | 1.317674632 | 0.014976587 | 0.022543704 | Up |
| CHST12 | 1011.460627 | 1466.162732 | 556.7585227 | 0.379771807 | -1.396795287 | 5.23E-47 | 5.06E-46 | Down |
| CHST14 | 1045.468669 | 1445.497675 | 645.4396617 | 0.446624728 | -1.162864963 | 1.73E-35 | 1.21E-34 | Down |
| CHST15 | 6173.790481 | 10504.81204 | 1842.768919 | 0.175422505 | -2.511094255 | 0 | 0 | Down |
| CHST2 | 9079.374412 | 12573.59875 | 5585.150073 | 0.444200846 | -1.170715953 | 2.07E-66 | 3.11E-65 | Down |
| CHST5 | 18.02654155 | 28.27458273 | 7.778500371 | 0.27464813 | -1.864343627 | 0.008066325 | 0.012587242 | Down |
| CHST6 | 47.54755293 | 27.32638889 | 67.76871696 | 2.480403093 | 1.310574594 | 0.00157701 | 0.002668949 | Up |
| CHST7 | 1038.39843 | 422.4827543 | 1654.314106 | 3.916041775 | 1.969396155 | 2.08E-80 | 4.24E-79 | Up |
| CHST9 | 21.0002739 | 0.365611098 | 41.63493669 | 104.9773221 | 6.713933891 | 3.11E-07 | 7.02E-07 | Up |
| CHTF18 | 3172.952584 | 4813.773528 | 1532.131641 | 0.318269474 | -1.651679302 | 3.62E-154 | 1.99E-152 | Down |
| CIAO3 | 4498.586935 | 6154.537649 | 2842.636221 | 0.461918503 | -1.114289756 | 8.89E-65 | 1.28E-63 | Down |
| CIART | 40.45536748 | 55.87883697 | 25.03189799 | 0.44821211 | -1.157746467 | 0.007383498 | 0.011580338 | Down |
| CIDEB | 374.7150371 | 609.0375976 | 140.3924766 | 0.230534126 | -2.116947766 | 1.18E-46 | 1.13E-45 | Down |
| CIDEC | 7.153287505 | 0 | 14.30657501 | 70.24579512 | 6.134339964 | 3.51E-05 | 6.87E-05 | Up |
| CIITA | 7499.000912 | 11038.94928 | 3959.052545 | 0.358634713 | -1.47941296 | 6.72E-172 | 4.50E-170 | Down |
| CILP | 13.69116173 | 6.218966791 | 21.16335667 | 3.391916213 | 1.762100533 | 0.01834625 | 0.027301882 | Up |
| CILP2 | 39.69687589 | 10.03292864 | 69.36082314 | 6.936904406 | 2.794292004 | 1.49E-07 | 3.42E-07 | Up |
| CIR1 | 1379.972729 | 851.7412543 | 1908.204204 | 2.241010672 | 1.164149519 | 1.60E-29 | 9.40E-29 | Up |
| CIT | 6750.807478 | 10053.0065 | 3448.60846 | 0.343041994 | -1.543542898 | 3.52E-130 | 1.46E-128 | Down |
| CKM | 57.93931279 | 113.807528 | 2.071097608 | 0.018320986 | -5.770359006 | 5.06E-19 | 2.03E-18 | Down |
| CLBA1 | 158.6732807 | 55.7885119 | 261.5580495 | 4.692120776 | 2.230240149 | 1.10E-23 | 5.26E-23 | Up |
| CLCC1 | 1269.407863 | 1715.046971 | 823.7687562 | 0.480374537 | -1.057768415 | 7.37E-37 | 5.38E-36 | Down |
| CLCF1 | 35.82931105 | 3.736958531 | 67.92166356 | 18.34298731 | 4.197156708 | 1.67E-11 | 4.77E-11 | Up |
| CLCN3 | 1772.710681 | 1040.649903 | 2504.771458 | 2.407002754 | 1.267237792 | 3.58E-63 | 4.95E-62 | Up |
| CLCN4 | 4.703976779 | 0.751434084 | 8.656519474 | 11.65810312 | 3.543261163 | 0.016145892 | 0.024214474 | Up |
| CLCN5 | 1026.974321 | 1459.948101 | 594.0005411 | 0.406925402 | -1.297163754 | 3.12E-46 | 2.96E-45 | Down |
| CLCNKA | 39.0349403 | 1.791209838 | 76.27867077 | 41.53505601 | 5.376257595 | 1.13E-12 | 3.40E-12 | Up |
| CLDN15 | 1000.645863 | 629.8331288 | 1371.458598 | 2.176145339 | 1.121774913 | 1.15E-29 | 6.78E-29 | Up |
| CLEC11A | 3982.090749 | 5961.325491 | 2002.856007 | 0.3359556 | -1.573657515 | 3.10E-125 | 1.24E-123 | Down |
| CLEC12A | 116.0150404 | 156.4831675 | 75.54691325 | 0.482873915 | -1.050281565 | 0.00013606 | 0.000255419 | Down |
| CLEC14A | 4210.118448 | 6461.74827 | 1958.488627 | 0.30307719 | -1.722242818 | 1.93E-188 | 1.50E-186 | Down |
| CLEC18A | 61.00539228 | 103.8372902 | 18.1734944 | 0.175168816 | -2.513182133 | 4.02E-11 | 1.13E-10 | Down |
| CLEC2B | 1732.399331 | 498.0021844 | 2966.796478 | 5.959597277 | 2.575214843 | 7.45E-118 | 2.68E-116 | Up |
| CLEC2D | 7432.863666 | 3951.713741 | 10914.01359 | 2.761724756 | 1.465569542 | 7.89E-108 | 2.50E-106 | Up |
| CLEC3B | 31.66586833 | 10.268057 | 53.06367966 | 5.152635218 | 2.36531046 | 1.23E-06 | 2.66E-06 | Up |
| CLEC4F | 4.99177978 | 9.983559561 | 0 | 0.016478374 | -5.923282262 | 0.000609926 | 0.001073231 | Down |
| CLEC4M | 13.27242109 | 3.27565518 | 23.269187 | 7.052348265 | 2.818103721 | 0.000546137 | 0.000965643 | Up |
| CLECL1 | 55.77084859 | 3.36213602 | 108.1795612 | 32.51252603 | 5.022923744 | 4.49E-18 | 1.73E-17 | Up |
| CLIP2 | 2393.723689 | 3586.680376 | 1200.767002 | 0.334807055 | -1.578598167 | 1.70E-91 | 4.16E-90 | Down |
| CLK1 | 6905.999265 | 3981.755735 | 9830.242796 | 2.468612542 | 1.303700418 | 2.23E-64 | 3.16E-63 | Up |
| CLK4 | 1388.376414 | 705.0013502 | 2071.751477 | 2.937214834 | 1.554448791 | 5.80E-52 | 6.34E-51 | Up |
| CLMN | 979.1518863 | 1407.244134 | 551.0596384 | 0.391624497 | -1.352457084 | 1.96E-46 | 1.87E-45 | Down |
| CLNS1A | 2946.457608 | 3948.8587 | 1944.056517 | 0.492276186 | -1.022460146 | 1.85E-46 | 1.77E-45 | Down |
| CLSPN | 1670.420487 | 2308.817072 | 1032.023903 | 0.446929299 | -1.161881469 | 4.45E-47 | 4.32E-46 | Down |
| CLSTN1 | 8376.028341 | 11529.71349 | 5222.343192 | 0.452965638 | -1.142526483 | 5.44E-87 | 1.23E-85 | Down |
| CLTRN | 10.94698539 | 4.343065173 | 17.5509056 | 4.010076785 | 2.003629862 | 0.017282461 | 0.02580463 | Up |
| CLU | 4.664115035 | 8.138870306 | 1.189359764 | 0.146381729 | -2.772192601 | 0.028533112 | 0.041408537 | Down |
| CLYBL | 824.2930591 | 1432.736592 | 215.8495258 | 0.150637444 | -2.730847673 | 5.15E-133 | 2.23E-131 | Down |
| CMTM2 | 4.339375814 | 0 | 8.678751628 | 42.59716174 | 5.412685401 | 0.001443946 | 0.002452427 | Up |
| CMTM4 | 295.9609202 | 463.5145666 | 128.4072739 | 0.277025032 | -1.85191175 | 5.57E-30 | 3.32E-29 | Down |
| CMTM7 | 1761.061456 | 2627.58851 | 894.5344028 | 0.340482808 | -1.554346139 | 6.68E-90 | 1.59E-88 | Down |
| CMYA5 | 68.51937002 | 31.50954803 | 105.529192 | 3.341082954 | 1.740315803 | 4.71E-07 | 1.05E-06 | Up |
| CNIH3 | 50.82926153 | 31.01397834 | 70.64454472 | 2.278407091 | 1.188025541 | 0.001574877 | 0.002665664 | Up |
| CNKSR1 | 56.91548399 | 31.24167629 | 82.58929169 | 2.650539986 | 1.406286305 | 0.000149803 | 0.000280155 | Up |
| CNOT10 | 2621.669168 | 1626.573453 | 3616.764883 | 2.224091284 | 1.153216002 | 2.50E-55 | 2.96E-54 | Up |
| CNPY1 | 10.74229943 | 19.99448725 | 1.49011161 | 0.074564439 | -3.745368432 | 0.000230734 | 0.000423629 | Down |
| CNTF | 48.67165402 | 29.13244343 | 68.2108646 | 2.341437723 | 1.227394666 | 0.000812045 | 0.001412398 | Up |
| CNTFR | 3.128021336 | 0 | 6.256042671 | 30.7188406 | 4.941051861 | 0.005940266 | 0.009449246 | Up |
| CNTNAP4 | 73.97366275 | 0.771645971 | 147.1756795 | 199.1746632 | 7.637890325 | 5.51E-13 | 1.69E-12 | Up |
| COA5 | 736.6357046 | 988.9639446 | 484.3074646 | 0.489773318 | -1.029813914 | 4.42E-21 | 1.90E-20 | Down |
| COBL | 704.021734 | 1008.156089 | 399.8873796 | 0.396552747 | -1.334415317 | 2.00E-24 | 9.79E-24 | Down |
| COL11A2 | 354.170633 | 216.6946014 | 491.6466646 | 2.268315199 | 1.181621127 | 1.83E-14 | 5.99E-14 | Up |
| COL15A1 | 6.086270682 | 1.128045656 | 11.04449571 | 9.904841456 | 3.308133883 | 0.009752158 | 0.015031913 | Up |
| COL1A1 | 104.5156105 | 151.3078126 | 57.72340827 | 0.381687425 | -1.389536437 | 1.26E-06 | 2.71E-06 | Down |
| COL20A1 | 18.83733658 | 2.513220621 | 35.16145253 | 13.74400493 | 3.780730554 | 2.13E-06 | 4.51E-06 | Up |
| COL25A1 | 4.405657829 | 0.751434084 | 8.059881575 | 10.85100138 | 3.439756282 | 0.022121307 | 0.032563371 | Up |
| COL27A1 | 818.1964337 | 496.7761909 | 1139.616677 | 2.295236592 | 1.198642874 | 1.25E-30 | 7.58E-30 | Up |
| COL8A2 | 10.32287109 | 20.35062083 | 0.295121352 | 0.015748767 | -5.988617266 | 1.95E-05 | 3.89E-05 | Down |
| COLEC11 | 15.28299324 | 5.834932866 | 24.73105361 | 4.216901303 | 2.076183256 | 0.002962867 | 0.004871154 | Up |
| COLEC12 | 41.92422838 | 26.48847397 | 57.35998279 | 2.163743809 | 1.113529692 | 0.021405875 | 0.031583969 | Up |
| COLGALT2 | 87.20308751 | 3.986665488 | 170.4195095 | 42.11672379 | 5.39632131 | 2.63E-25 | 1.34E-24 | Up |
| COLQ | 169.5788083 | 106.7765076 | 232.381109 | 2.174957883 | 1.120987464 | 2.94E-07 | 6.65E-07 | Up |
| COMP | 4.969323216 | 0.356399685 | 9.582246746 | 24.14625869 | 4.593727765 | 0.006339485 | 0.010039387 | Up |
| COPS5 | 3829.259936 | 1726.368001 | 5932.151871 | 3.435178656 | 1.780385132 | 5.06E-125 | 2.02E-123 | Up |
| COPZ2 | 161.7620448 | 102.629928 | 220.8941616 | 2.152862134 | 1.106255935 | 3.81E-08 | 9.06E-08 | Up |
| COQ10A | 1015.164571 | 1371.30836 | 659.0207818 | 0.480646157 | -1.056952895 | 2.61E-28 | 1.47E-27 | Down |
| COQ10B | 1302.155359 | 864.4285954 | 1739.882122 | 2.012406367 | 1.008921659 | 4.79E-35 | 3.30E-34 | Up |
| COQ4 | 1229.291292 | 701.7225927 | 1756.859991 | 2.504391767 | 1.324460264 | 3.78E-47 | 3.68E-46 | Up |
| COQ5 | 1707.043148 | 1059.935702 | 2354.150593 | 2.22225643 | 1.152025301 | 2.65E-37 | 1.97E-36 | Up |
| COQ9 | 3035.72961 | 1832.44563 | 4239.013591 | 2.313350618 | 1.209983941 | 2.73E-64 | 3.85E-63 | Up |
| CORIN | 36.77251508 | 17.20724552 | 56.33778465 | 3.264824692 | 1.707005527 | 0.000200387 | 0.000370165 | Up |
| CORO1A | 11642.29902 | 15767.32952 | 7517.268522 | 0.476753167 | -1.068685572 | 9.90E-67 | 1.49E-65 | Down |
| CORO6 | 645.4102861 | 1156.113081 | 134.7074911 | 0.116516131 | -3.101398392 | 1.75E-140 | 8.23E-139 | Down |
| COTL1 | 38.57699288 | 58.64023353 | 18.51375223 | 0.31583071 | -1.662776635 | 0.000154305 | 0.000288304 | Down |
| CPAMD8 | 142.6047249 | 258.4000461 | 26.80940365 | 0.103736866 | -3.268999402 | 6.13E-37 | 4.51E-36 | Down |
| CPB2 | 10.45228173 | 3.630265803 | 17.27429766 | 4.724016934 | 2.240014136 | 0.009741861 | 0.015019379 | Up |
| CPEB4 | 1010.598353 | 336.2405958 | 1684.95611 | 5.007640281 | 2.324130931 | 7.94E-79 | 1.56E-77 | Up |
| CPED1 | 13.10620262 | 2.956101147 | 23.25630408 | 7.873808703 | 2.977061662 | 0.000356425 | 0.00064174 | Up |
| CPLX3 | 13.43954539 | 2.13839811 | 24.74069267 | 11.33976716 | 3.503319112 | 0.000199273 | 0.000368205 | Up |
| CPM | 964.1312228 | 1493.533115 | 434.7293308 | 0.291101088 | -1.780407862 | 4.23E-78 | 8.19E-77 | Down |
| CPN2 | 2.843778621 | 0 | 5.687557242 | 27.91714344 | 4.803079423 | 0.02004415 | 0.029688458 | Up |
| CPNE2 | 1586.076316 | 2378.88775 | 793.2648816 | 0.333485805 | -1.584302741 | 1.17E-65 | 1.72E-64 | Down |
| CPNE5 | 15.12090749 | 3.295867067 | 26.9459479 | 8.147643176 | 3.026382799 | 0.000992018 | 0.001714035 | Up |
| CPNE7 | 353.4789946 | 534.329705 | 172.6282842 | 0.323028485 | -1.630266705 | 1.64E-30 | 9.97E-30 | Down |
| CPO | 20.23974655 | 6.2133335 | 34.2661596 | 5.490463483 | 2.456927941 | 0.000103748 | 0.000196623 | Up |
| CPT1A | 4391.736666 | 6131.551172 | 2651.92216 | 0.432471057 | -1.209324512 | 2.19E-91 | 5.32E-90 | Down |
| CPXM2 | 44.24106081 | 24.08015701 | 64.40196461 | 2.678173759 | 1.421249565 | 0.000764119 | 0.001331545 | Up |
| CRACD | 2139.906202 | 3029.912043 | 1249.900362 | 0.412539078 | -1.277397308 | 3.15E-57 | 3.89E-56 | Down |
| CRACDL | 7.430851321 | 0 | 14.86170264 | 72.98991391 | 6.189625214 | 3.72E-05 | 7.27E-05 | Up |
| CRACR2B | 1129.794225 | 2116.642562 | 142.9458878 | 0.067570654 | -3.887459361 | 1.99E-259 | 2.61E-257 | Down |
| CRADD | 1040.899526 | 457.8464299 | 1623.952622 | 3.546142131 | 1.826250361 | 5.87E-81 | 1.21E-79 | Up |
| CRB1 | 20.9482013 | 2.579489574 | 39.31691302 | 15.23052304 | 3.928893582 | 9.70E-07 | 2.11E-06 | Up |
| CRB2 | 92.68467739 | 128.5547823 | 56.81457248 | 0.442050849 | -1.177715763 | 0.000195265 | 0.000361136 | Down |
| CRB3 | 27.69251855 | 4.817158061 | 50.56787905 | 10.52906464 | 3.396305373 | 2.99E-08 | 7.15E-08 | Up |
| CRBN | 2244.761906 | 1424.572852 | 3064.95096 | 2.151573915 | 1.105392403 | 4.42E-47 | 4.29E-46 | Up |
| CREB3 | 1792.926083 | 1064.511307 | 2521.340859 | 2.36843934 | 1.243936722 | 1.41E-57 | 1.75E-56 | Up |
| CREB3L1 | 206.3343893 | 379.2812981 | 33.38748056 | 0.088000594 | -3.50634292 | 8.07E-47 | 7.77E-46 | Down |
| CREBRF | 955.8844783 | 583.9970056 | 1327.771951 | 2.273351967 | 1.184821064 | 4.67E-28 | 2.61E-27 | Up |
| CREM | 1545.411234 | 201.0981736 | 2889.724294 | 14.37235699 | 3.84522477 | 0 | 0 | Up |
| CRIM1 | 5103.783608 | 3079.990164 | 7127.577052 | 2.314030435 | 1.21040784 | 5.56E-91 | 1.34E-89 | Up |
| CRISPLD1 | 205.8908315 | 288.9327592 | 122.8489037 | 0.425103952 | -1.234112425 | 2.07E-08 | 4.99E-08 | Down |
| CRLF1 | 3.611838817 | 0.356399685 | 6.867277949 | 17.30830315 | 4.113392389 | 0.0157184 | 0.023606531 | Up |
| CRMP1 | 13.44985167 | 1.543291942 | 25.35641139 | 16.94772658 | 4.083019854 | 4.18E-05 | 8.13E-05 | Up |
| CROCC2 | 15.03891376 | 28.57598004 | 1.501847477 | 0.052278354 | -4.257642476 | 1.78E-05 | 3.57E-05 | Down |
| CROT | 1050.991572 | 1620.803773 | 481.1793701 | 0.29686608 | -1.752115834 | 1.31E-87 | 2.99E-86 | Down |
| CRTAP | 4895.86796 | 7443.292311 | 2348.443609 | 0.315517195 | -1.664209463 | 1.88E-119 | 6.94E-118 | Down |
| CRTC2 | 3640.998986 | 2123.248068 | 5158.749904 | 2.429485792 | 1.280650995 | 5.59E-101 | 1.56E-99 | Up |
| CRYBA4 | 16.23946162 | 24.40329723 | 8.075626005 | 0.330043595 | -1.599271493 | 0.026190415 | 0.038184491 | Down |
| CRYBB2 | 19.98010228 | 0 | 39.96020456 | 196.1956857 | 7.616149507 | 4.27E-09 | 1.08E-08 | Up |
| CRYBB3 | 2.226912849 | 0 | 4.453825699 | 21.87605974 | 4.451281002 | 0.030671028 | 0.044288761 | Up |
| CRYBG1 | 2663.534746 | 3825.494273 | 1501.575219 | 0.392528561 | -1.349130465 | 2.69E-103 | 7.92E-102 | Down |
| CRYM | 6.840654881 | 0 | 13.68130976 | 67.18852328 | 6.070142917 | 7.26E-05 | 0.000139434 | Up |
| CSAD | 271.3432445 | 411.9992728 | 130.6872162 | 0.317225759 | -1.656418172 | 4.96E-14 | 1.59E-13 | Down |
| CSF1 | 172.9076599 | 28.92316831 | 316.8921514 | 10.91523077 | 3.448270726 | 1.93E-32 | 1.23E-31 | Up |
| CSF1R | 35.04507917 | 2.96531256 | 67.12484577 | 22.714466 | 4.505539484 | 1.43E-11 | 4.11E-11 | Up |
| CSMD1 | 1182.094508 | 686.2103981 | 1677.978619 | 2.446473735 | 1.290703794 | 1.52E-47 | 1.50E-46 | Up |
| CSNK1D | 9031.59235 | 5525.553188 | 12537.63151 | 2.269155753 | 1.182155638 | 2.57E-79 | 5.08E-78 | Up |
| CSNKA2IP | 2.987093294 | 0 | 5.974186589 | 29.32760946 | 4.874187574 | 0.010131673 | 0.015587449 | Up |
| CSPG5 | 49.46995166 | 2.948678795 | 95.99122453 | 32.5335507 | 5.023856381 | 2.37E-16 | 8.38E-16 | Up |
| CSRNP1 | 3131.1751 | 1427.597261 | 4834.75294 | 3.387025696 | 1.760018932 | 2.40E-105 | 7.31E-104 | Up |
| CTH | 317.7179246 | 476.9311414 | 158.5047078 | 0.33250092 | -1.588569761 | 3.14E-25 | 1.59E-24 | Down |
| CTIF | 1520.603614 | 2208.353426 | 832.8538021 | 0.377073533 | -1.407082205 | 6.69E-67 | 1.01E-65 | Down |
| CTNNBIP1 | 467.8998725 | 702.7409158 | 233.0588292 | 0.331656886 | -1.592236614 | 7.24E-38 | 5.45E-37 | Down |
| CTPS1 | 7491.815857 | 10929.07765 | 4054.554067 | 0.370992342 | -1.430538687 | 1.18E-121 | 4.48E-120 | Down |
| CTSC | 1836.8972 | 2665.641146 | 1008.153253 | 0.378170533 | -1.40289114 | 4.22E-74 | 7.45E-73 | Down |
| CTSE | 5.062436905 | 0 | 10.12487381 | 49.71791211 | 5.635693807 | 0.000392192 | 0.000704584 | Up |
| CTSF | 8.310645945 | 13.93132423 | 2.689967661 | 0.192698792 | -2.375580568 | 0.01394218 | 0.021059781 | Down |
| CTSG | 83.62888821 | 147.0351267 | 20.22264967 | 0.137680809 | -2.860600613 | 1.72E-15 | 5.90E-15 | Down |
| CTSH | 7.590530215 | 0 | 15.18106043 | 74.54929657 | 6.220122835 | 2.24E-05 | 4.45E-05 | Up |
| CTSO | 995.8187319 | 1484.840819 | 506.796645 | 0.341301515 | -1.550881278 | 8.41E-67 | 1.27E-65 | Down |
| CTSV | 107.7806267 | 172.3328642 | 43.22838924 | 0.250839727 | -1.995162243 | 8.31E-12 | 2.42E-11 | Down |
| CTSW | 4.332123389 | 0 | 8.664246778 | 42.53183972 | 5.410471355 | 0.001220396 | 0.002086799 | Up |
| CTSZ | 48.78551539 | 4.490181676 | 93.0808491 | 20.94870589 | 4.388789219 | 1.18E-15 | 4.06E-15 | Up |
| CTXN2 | 14.93656868 | 0.356399685 | 29.51673767 | 74.40629321 | 6.217352743 | 2.73E-06 | 5.75E-06 | Up |
| CUBN | 128.3347237 | 176.277591 | 80.39185629 | 0.455996814 | -1.132904351 | 1.80E-06 | 3.86E-06 | Down |
| CUL1 | 4821.863771 | 3203.804292 | 6439.923249 | 2.010117893 | 1.007280118 | 1.39E-72 | 2.40E-71 | Up |
| CWC22 | 1792.878951 | 1128.817131 | 2456.940771 | 2.176188028 | 1.121803214 | 6.15E-26 | 3.20E-25 | Up |
| CWF19L2 | 879.976873 | 446.3559655 | 1313.597781 | 2.942527939 | 1.557056114 | 2.09E-31 | 1.30E-30 | Up |
| CX3CR1 | 22.21129239 | 9.169434647 | 35.25315013 | 3.834621376 | 1.939084135 | 0.001033313 | 0.001782943 | Up |
| CXADR | 14.2200916 | 2.177032824 | 26.26315037 | 11.9327141 | 3.576850316 | 3.41E-05 | 6.69E-05 | Up |
| CXCL10 | 15.48294936 | 6.803330528 | 24.16256818 | 3.579618512 | 1.839805844 | 0.018216763 | 0.027126647 | Up |
| CXCL11 | 3.323518628 | 0.365611098 | 6.281426158 | 15.82938674 | 3.984533458 | 0.028666035 | 0.04157969 | Up |
| CXCL16 | 9.160648448 | 15.63657738 | 2.684719517 | 0.171873499 | -2.540580981 | 0.005399387 | 0.008632423 | Down |
| CXCL8 | 390.1016604 | 1.828055491 | 778.3752654 | 422.0970068 | 8.721430789 | 4.44E-40 | 3.56E-39 | Up |
| CXCR5 | 2.388213674 | 0 | 4.776427348 | 23.44869797 | 4.551435912 | 0.021959295 | 0.03234548 | Up |
| CXorf65 | 70.04932972 | 36.43313271 | 103.6655267 | 2.843916607 | 1.507879161 | 8.19E-05 | 0.000156526 | Up |
| CYB561 | 46.91822905 | 30.99913364 | 62.83732447 | 2.027642545 | 1.019803341 | 0.00563576 | 0.008986508 | Up |
| CYB5D1 | 529.6715932 | 289.5521795 | 769.7910068 | 2.658425657 | 1.410572122 | 6.00E-33 | 3.87E-32 | Up |
| CYBB | 4.89054186 | 8.892093451 | 0.888990269 | 0.100379386 | -3.316465062 | 0.013246335 | 0.02007209 | Down |
| CYBC1 | 4874.477506 | 7241.540494 | 2507.414518 | 0.346242901 | -1.530143602 | 7.58E-119 | 2.78E-117 | Down |
| CYGB | 10.70615831 | 4.106147752 | 17.30616887 | 4.235564947 | 2.082554412 | 0.015958381 | 0.023943613 | Up |
| CYLD | 2788.991031 | 1216.548835 | 4361.433226 | 3.584506577 | 1.841774539 | 8.66E-164 | 5.29E-162 | Up |
| CYP19A1 | 5.571844674 | 0.385822986 | 10.75786636 | 27.1123786 | 4.760879783 | 0.002410086 | 0.00400891 | Up |
| CYP1A1 | 205.7151352 | 5.834932866 | 405.5953375 | 68.79656483 | 6.104264624 | 4.85E-52 | 5.32E-51 | Up |
| CYP21A2 | 24.79364911 | 9.939291557 | 39.64800667 | 3.985211096 | 1.994656146 | 0.000218977 | 0.000403002 | Up |
| CYP26B1 | 27.65090851 | 4.33385376 | 50.96796327 | 11.60403305 | 3.536554404 | 1.68E-08 | 4.08E-08 | Up |
| CYP4F2 | 67.52892127 | 131.7953809 | 3.262461653 | 0.024848003 | -5.330726293 | 1.19E-23 | 5.69E-23 | Down |
| CYRIA | 225.1617091 | 40.08440066 | 410.2390176 | 10.2705669 | 3.360443911 | 5.57E-51 | 5.91E-50 | Up |
| CYS1 | 6.386471552 | 1.42559874 | 11.34734436 | 7.815379439 | 2.966315918 | 0.017172885 | 0.025652067 | Up |
| CYSLTR1 | 33.18312906 | 46.09866925 | 20.26758886 | 0.439096682 | -1.187389461 | 0.013060838 | 0.019814819 | Down |
| CYTH4 | 204.8368021 | 64.28889103 | 345.3847132 | 5.362177663 | 2.42281902 | 3.05E-31 | 1.88E-30 | Up |
| CYTIP | 1227.501828 | 30.42781748 | 2424.575838 | 80.10611319 | 6.323840439 | 4.77E-275 | 7.04E-273 | Up |
| D2HGDH | 761.2268558 | 1017.301459 | 505.1522527 | 0.496473084 | -1.010212591 | 1.24E-20 | 5.23E-20 | Down |
| DACT1 | 471.6277263 | 669.081687 | 274.1737656 | 0.409892045 | -1.286684104 | 1.22E-23 | 5.83E-23 | Down |
| DAD1 | 3149.677515 | 1945.397304 | 4353.957725 | 2.238555295 | 1.162567955 | 1.98E-60 | 2.60E-59 | Up |
| DAPK2 | 41.60566245 | 81.12209623 | 2.08922867 | 0.025739185 | -5.279889805 | 3.38E-15 | 1.14E-14 | Down |
| DAZ1 | 169.1371658 | 23.58048505 | 314.6938466 | 13.33622161 | 3.737278078 | 3.85E-41 | 3.16E-40 | Up |
| DBNDD2 | 70.06174792 | 11.91983073 | 128.2036651 | 10.81876209 | 3.435463526 | 3.68E-18 | 1.42E-17 | Up |
| DCAF13 | 2001.05347 | 1269.542909 | 2732.564031 | 2.152764583 | 1.106190562 | 1.36E-50 | 1.43E-49 | Up |
| DCAF16 | 4040.934203 | 5972.23711 | 2109.631296 | 0.353230579 | -1.501317852 | 2.08E-161 | 1.22E-159 | Down |
| DCAF4L1 | 48.84338447 | 18.51719816 | 79.16957079 | 4.28315252 | 2.098673051 | 3.93E-07 | 8.81E-07 | Up |
| DCDC2C | 5.962734425 | 0 | 11.92546885 | 58.55275321 | 5.871665104 | 0.000126146 | 0.00023758 | Up |
| DCP1B | 1038.212452 | 494.3038929 | 1582.121011 | 3.199182098 | 1.677703113 | 2.36E-74 | 4.20E-73 | Up |
| DCUN1D4 | 544.1073769 | 730.6851526 | 357.5296013 | 0.489281389 | -1.031263688 | 4.52E-14 | 1.45E-13 | Down |
| DDHD1 | 2636.929484 | 3638.343258 | 1635.515709 | 0.449506084 | -1.153587452 | 4.23E-47 | 4.11E-46 | Down |
| DDIT4L | 915.166671 | 1478.800213 | 351.5331288 | 0.237669691 | -2.072970158 | 4.17E-93 | 1.05E-91 | Down |
| DDN | 2226.848754 | 3267.087283 | 1186.610225 | 0.363244513 | -1.460987088 | 1.93E-76 | 3.60E-75 | Down |
| DDO | 90.61524915 | 158.3355372 | 22.89496115 | 0.144765378 | -2.788211486 | 8.99E-19 | 3.56E-18 | Down |
| DDX10 | 2032.396002 | 1257.505675 | 2807.286329 | 2.232236575 | 1.158489934 | 7.17E-64 | 1.00E-62 | Up |
| DDX11 | 3265.626733 | 4565.708589 | 1965.544877 | 0.430499295 | -1.21591722 | 2.81E-95 | 7.24E-94 | Down |
| DDX20 | 2761.853655 | 1584.006028 | 3939.701281 | 2.487204498 | 1.314525131 | 7.43E-101 | 2.06E-99 | Up |
| DDX27 | 6035.62355 | 3213.680387 | 8857.566713 | 2.756780038 | 1.46298416 | 2.15E-120 | 8.04E-119 | Up |
| DDX31 | 1815.790777 | 1126.800421 | 2504.781134 | 2.221851512 | 1.151762404 | 2.76E-42 | 2.35E-41 | Up |
| DDX47 | 3297.488665 | 2179.558477 | 4415.418854 | 2.025531042 | 1.018300195 | 1.46E-52 | 1.63E-51 | Up |
| DDX49 | 3995.731796 | 2072.135214 | 5919.328377 | 2.856571488 | 1.514284635 | 5.14E-121 | 1.94E-119 | Up |
| DDX50 | 2698.778907 | 1739.858268 | 3657.699546 | 2.102660072 | 1.072215635 | 8.98E-54 | 1.03E-52 | Up |
| DDX56 | 4748.483467 | 2954.401174 | 6542.56576 | 2.214680375 | 1.147098503 | 5.34E-65 | 7.72E-64 | Up |
| DELE1 | 1216.165413 | 1644.017115 | 788.3137117 | 0.479411157 | -1.060664612 | 4.14E-34 | 2.78E-33 | Down |
| DENND11 | 1276.395618 | 1938.902026 | 613.8892111 | 0.316593928 | -1.659294509 | 1.05E-74 | 1.90E-73 | Down |
| DENND2D | 390.2207682 | 253.7535204 | 526.688016 | 2.074695652 | 1.052899715 | 1.16E-13 | 3.66E-13 | Up |
| DENND6B | 225.7109254 | 99.28415961 | 352.1376912 | 3.547396491 | 1.826760589 | 1.58E-21 | 6.92E-21 | Up |
| DEPDC1B | 1402.036963 | 2001.686178 | 802.3877485 | 0.40080507 | -1.319027335 | 3.56E-59 | 4.57E-58 | Down |
| DEPDC4 | 87.76699364 | 117.6877749 | 57.84621238 | 0.49148203 | -1.024789428 | 0.000282918 | 0.000515616 | Down |
| DEPP1 | 493.725895 | 712.4143252 | 275.0374649 | 0.386102145 | -1.372945526 | 1.45E-26 | 7.72E-26 | Down |
| DEPTOR | 83.09114224 | 145.0100535 | 21.17223103 | 0.145980587 | -2.77615157 | 6.79E-19 | 2.71E-18 | Down |
| DERL3 | 581.0100708 | 1085.186158 | 76.83398346 | 0.070846188 | -3.819165968 | 6.77E-142 | 3.26E-140 | Down |
| DGAT1 | 991.4363104 | 1397.260549 | 585.6120715 | 0.419035745 | -1.25485478 | 5.43E-34 | 3.62E-33 | Down |
| DGKG | 138.6377644 | 59.85039165 | 217.4251371 | 3.634642857 | 1.861813611 | 1.52E-13 | 4.76E-13 | Up |
| DGKI | 15.21651912 | 0.356399685 | 30.07663856 | 75.82716887 | 6.244642953 | 2.73E-06 | 5.74E-06 | Up |
| DHFR | 7368.076868 | 11036.22745 | 3699.926291 | 0.335238531 | -1.576740117 | 1.87E-211 | 1.82E-209 | Down |
| DHPS | 3248.74185 | 4942.680737 | 1554.802962 | 0.314539803 | -1.668685501 | 1.96E-143 | 9.59E-142 | Down |
| DHRS1 | 334.8552953 | 538.8308952 | 130.8796953 | 0.242842799 | -2.041905388 | 7.15E-43 | 6.19E-42 | Down |
| DHRS11 | 388.9964526 | 632.1803274 | 145.8125779 | 0.230573449 | -2.116701701 | 2.34E-50 | 2.45E-49 | Down |
| DHRS2 | 838.6908721 | 21.95250133 | 1655.429243 | 74.87271297 | 6.226368126 | 5.88E-188 | 4.53E-186 | Up |
| DHRS3 | 21.64556495 | 2.175243763 | 41.11588613 | 18.66655156 | 4.222383525 | 1.32E-07 | 3.03E-07 | Up |
| DHRS7B | 715.1841553 | 298.6852846 | 1131.683026 | 3.791880942 | 1.922913667 | 2.25E-68 | 3.53E-67 | Up |
| DHX29 | 1661.714813 | 1093.473377 | 2229.956249 | 2.039215028 | 1.02801391 | 6.40E-41 | 5.22E-40 | Up |
| DHX37 | 3939.134452 | 2486.199219 | 5392.069685 | 2.168918061 | 1.116975551 | 1.08E-60 | 1.44E-59 | Up |
| DIABLO | 3252.395621 | 2089.223567 | 4415.567675 | 2.113518267 | 1.079646581 | 2.63E-63 | 3.66E-62 | Up |
| DIO2 | 62.32987188 | 23.21692912 | 101.4428146 | 4.367303205 | 2.126742695 | 3.22E-07 | 7.25E-07 | Up |
| DIP2A | 1333.782111 | 2012.033085 | 655.5311369 | 0.32576846 | -1.618081164 | 2.44E-84 | 5.41E-83 | Down |
| DIPK1B | 1801.980986 | 3100.096037 | 503.8659349 | 0.162563867 | -2.620921466 | 5.85E-234 | 6.65E-232 | Down |
| DIXDC1 | 289.9216332 | 485.7599865 | 94.08327998 | 0.193705406 | -2.36806388 | 2.14E-38 | 1.64E-37 | Down |
| DKK3 | 3.739638964 | 0 | 7.479277928 | 36.71104707 | 5.198142358 | 0.003354858 | 0.005478601 | Up |
| DLG2 | 90.97348808 | 159.2745196 | 22.67245657 | 0.14227637 | -2.813232022 | 2.87E-21 | 1.24E-20 | Down |
| DLG3 | 1836.612721 | 2589.370999 | 1083.854443 | 0.418585359 | -1.256406243 | 8.96E-68 | 1.38E-66 | Down |
| DLGAP1 | 21.32831672 | 3.630265803 | 39.02636764 | 10.64181528 | 3.411672362 | 6.21E-07 | 1.37E-06 | Up |
| DLL1 | 42.85482519 | 6.185699261 | 79.52395112 | 12.75213498 | 3.672666901 | 1.31E-11 | 3.76E-11 | Up |
| DLL3 | 203.7531842 | 314.9197802 | 92.58658812 | 0.293999289 | -1.766115427 | 2.78E-18 | 1.08E-17 | Down |
| DLX2 | 9.340278962 | 1.078410468 | 17.60214746 | 16.01853188 | 4.001670023 | 0.000408377 | 0.000732334 | Up |
| DMBT1 | 26.26453486 | 0 | 52.52906973 | 257.8856897 | 8.010587909 | 3.08E-10 | 8.25E-10 | Up |
| DMC1 | 72.93202936 | 112.760346 | 33.10371272 | 0.293471965 | -1.768705404 | 2.82E-08 | 6.74E-08 | Down |
| DMGDH | 169.3595976 | 253.4442341 | 85.27496096 | 0.33643485 | -1.571600938 | 2.71E-13 | 8.43E-13 | Down |
| DMRTA2 | 4.757630231 | 0 | 9.515260462 | 46.7310153 | 5.546308477 | 0.000652796 | 0.001145296 | Up |
| DNA2 | 1918.369742 | 2642.349585 | 1194.389899 | 0.452051021 | -1.145442482 | 3.07E-54 | 3.57E-53 | Down |
| DNAAF11 | 10.73372245 | 0 | 21.46744491 | 105.4022502 | 6.719761857 | 1.61E-06 | 3.46E-06 | Up |
| DNAAF3 | 131.5847946 | 184.0549525 | 79.11463669 | 0.429696364 | -1.218610527 | 7.50E-06 | 1.54E-05 | Down |
| DNAAF8 | 15.13597738 | 4.056512564 | 26.2154422 | 6.462392334 | 2.69206834 | 0.0003311 | 0.00059863 | Up |
| DNAH10 | 189.1679775 | 74.12917025 | 304.2067848 | 4.103622283 | 2.036897944 | 3.03E-24 | 1.48E-23 | Up |
| DNAH12 | 64.06629774 | 23.14297171 | 104.9896238 | 4.528970906 | 2.179183271 | 5.56E-09 | 1.39E-08 | Up |
| DNAH17 | 221.764593 | 26.80856021 | 416.7206257 | 15.49957011 | 3.954156297 | 1.26E-55 | 1.51E-54 | Up |
| DNAH3 | 68.14187211 | 4.995486925 | 131.2882573 | 26.44995649 | 4.725193444 | 0.00047284 | 0.000841838 | Up |
| DNAI4 | 188.9271882 | 40.69997676 | 337.1543996 | 8.33092841 | 3.058477281 | 2.44E-33 | 1.60E-32 | Up |
| DNAJB2 | 3631.542046 | 745.4979362 | 6517.586156 | 8.751107396 | 3.129465592 | 0 | 0 | Up |
| DNAJB5 | 663.907912 | 906.0443382 | 421.7714859 | 0.465514119 | -1.10310317 | 8.81E-18 | 3.35E-17 | Down |
| DNAJB6 | 2724.304487 | 1379.897202 | 4068.711772 | 2.948671486 | 1.5600651 | 5.29E-100 | 1.45E-98 | Up |
| DNAJC15 | 22.02954366 | 3.295867067 | 40.76322026 | 12.31592523 | 3.622453109 | 7.65E-07 | 1.68E-06 | Up |
| DNAJC2 | 1569.517755 | 981.4734433 | 2157.562066 | 2.198782846 | 1.13670513 | 6.10E-25 | 3.05E-24 | Up |
| DNAJC30 | 465.2092982 | 223.0184557 | 707.4001406 | 3.174626631 | 1.666586926 | 3.14E-34 | 2.11E-33 | Up |
| DNAJC5B | 2.711251496 | 5.122133496 | 0.300369495 | 0.062474789 | -4.000582066 | 0.032143583 | 0.046232232 | Down |
| DNAJC7 | 4079.058534 | 2397.484562 | 5760.632505 | 2.402530759 | 1.264554901 | 8.20E-102 | 2.33E-100 | Up |
| DNASE1L1 | 603.7647974 | 845.3229646 | 362.2066302 | 0.428513704 | -1.222586753 | 9.19E-29 | 5.26E-28 | Down |
| DNASE2 | 1354.789237 | 1904.953375 | 804.6250987 | 0.422333917 | -1.243543982 | 9.99E-46 | 9.33E-45 | Down |
| DNM1 | 1346.142257 | 2005.363616 | 686.9208973 | 0.342597735 | -1.545412482 | 2.48E-44 | 2.23E-43 | Down |
| DNM3 | 57.75879553 | 7.832105809 | 107.6854852 | 13.84479262 | 3.791271539 | 1.99E-15 | 6.78E-15 | Up |
| DNMT3B | 1980.380808 | 3156.920073 | 803.8415441 | 0.254583393 | -1.973789783 | 2.56E-134 | 1.14E-132 | Down |
| DNMT3L | 6.092737565 | 0 | 12.18547513 | 59.84748123 | 5.903218625 | 0.000134114 | 0.000251902 | Up |
| DNPH1 | 683.6525974 | 1173.661649 | 193.6435458 | 0.165038534 | -2.599125181 | 1.06E-105 | 3.25E-104 | Down |
| DNTT | 18745.11717 | 33864.95543 | 3625.278899 | 0.107051488 | -3.223623244 | 0 | 0 | Down |
| DNTTIP1 | 2535.136295 | 1684.264309 | 3386.008281 | 2.010274472 | 1.007392492 | 1.11E-35 | 7.77E-35 | Up |
| DNTTIP2 | 3084.627436 | 1677.141633 | 4492.113239 | 2.678223905 | 1.421276578 | 1.69E-52 | 1.88E-51 | Up |
| DOC2A | 69.58216032 | 121.5439496 | 17.62037105 | 0.144871503 | -2.78715426 | 6.87E-15 | 2.29E-14 | Down |
| DOCK10 | 6474.399471 | 3322.359447 | 9626.439496 | 2.897602135 | 1.534859514 | 4.31E-90 | 1.03E-88 | Up |
| DOCK8 | 169.8485718 | 53.03248252 | 286.664661 | 5.401481888 | 2.433355263 | 7.78E-29 | 4.47E-28 | Up |
| DOHH | 1043.615041 | 612.2263215 | 1475.00376 | 2.409066262 | 1.268474076 | 3.76E-35 | 2.61E-34 | Up |
| DOK3 | 11319.39421 | 18409.468 | 4229.32041 | 0.229747635 | -2.121878086 | 0 | 0 | Down |
| DOK5 | 9.382442076 | 0 | 18.76488415 | 92.1425526 | 6.52579566 | 3.86E-06 | 8.06E-06 | Up |
| DOK7 | 65.02184569 | 128.8523273 | 1.191364046 | 0.009253422 | -6.755797354 | 5.17E-18 | 1.98E-17 | Down |
| DPCD | 17.54112937 | 8.853458737 | 26.2288 | 2.962669573 | 1.566897731 | 0.010442083 | 0.016045434 | Up |
| DPEP1 | 91.06257489 | 165.3635197 | 16.76163006 | 0.101209503 | -3.304583331 | 4.07E-18 | 1.57E-17 | Down |
| DPF2 | 7699.730917 | 4997.960578 | 10401.50126 | 2.081415741 | 1.057565157 | 7.96E-80 | 1.60E-78 | Up |
| DPH2 | 1964.842212 | 1229.805197 | 2699.879227 | 2.195097445 | 1.134284986 | 8.13E-44 | 7.22E-43 | Up |
| DPYD | 151.6688602 | 65.81733827 | 237.5203822 | 3.604620285 | 1.849847292 | 1.66E-15 | 5.68E-15 | Up |
| DPYSL2 | 2365.906232 | 3579.173857 | 1152.638607 | 0.322022652 | -1.634765921 | 4.99E-117 | 1.77E-115 | Down |
| DRC1 | 6.408573517 | 1.493656754 | 11.32349028 | 7.635767185 | 2.932773116 | 0.010592492 | 0.016265743 | Up |
| DRC3 | 23.02450947 | 6.685637326 | 39.36338161 | 5.908269778 | 2.562735703 | 1.42E-05 | 2.85E-05 | Up |
| DRC7 | 23.14778071 | 6.597367424 | 39.698194 | 5.996701307 | 2.584169115 | 1.72E-05 | 3.45E-05 | Up |
| DRICH1 | 2796.287473 | 4620.185616 | 972.3893305 | 0.210460979 | -2.248375326 | 3.99E-235 | 4.57E-233 | Down |
| DSCC1 | 502.7669604 | 689.4909781 | 316.0429427 | 0.458461271 | -1.12512823 | 3.74E-16 | 1.31E-15 | Down |
| DTNBP1 | 1867.999381 | 1207.560517 | 2528.438245 | 2.093530912 | 1.06593822 | 1.45E-44 | 1.31E-43 | Up |
| DTX1 | 15044.15293 | 25864.28733 | 4224.018522 | 0.16332007 | -2.614226003 | 0 | 0 | Down |
| DTX4 | 701.4044198 | 343.5707418 | 1059.238098 | 3.085396771 | 1.625456027 | 3.72E-46 | 3.52E-45 | Up |
| DUSP10 | 987.1421325 | 220.6124186 | 1753.671846 | 7.936955101 | 2.988585644 | 7.86E-170 | 5.17E-168 | Up |
| DUSP12 | 1444.687324 | 853.9533679 | 2035.421281 | 2.383734005 | 1.253223258 | 4.74E-44 | 4.23E-43 | Up |
| DUSP14 | 1226.305142 | 631.0059344 | 1821.604349 | 2.88702934 | 1.529585769 | 1.59E-47 | 1.57E-46 | Up |
| DUSP16 | 6325.018632 | 2408.172611 | 10241.86465 | 4.252784918 | 2.088407893 | 2.52E-279 | 3.80E-277 | Up |
| DUSP19 | 66.47070222 | 117.1619917 | 15.77941279 | 0.134732705 | -2.891828001 | 3.51E-16 | 1.24E-15 | Down |
| DUSP2 | 494.6143082 | 144.0306553 | 845.1979611 | 5.864458587 | 2.551997923 | 3.15E-76 | 5.86E-75 | Up |
| DUSP22 | 1694.873069 | 2344.924262 | 1044.821877 | 0.445606597 | -1.166157504 | 1.70E-49 | 1.75E-48 | Down |
| DUSP26 | 17.49371658 | 6.961189461 | 28.02624371 | 4.014169523 | 2.005101545 | 0.002562517 | 0.004246168 | Up |
| DUSP4 | 152.1318118 | 80.45688494 | 223.8067386 | 2.78214648 | 1.47619838 | 3.79E-10 | 1.01E-09 | Up |
| DUSP5 | 545.047937 | 22.95211136 | 1067.143763 | 46.63376456 | 5.543302992 | 1.49E-138 | 6.96E-137 | Up |
| DUT | 4622.955602 | 6856.640931 | 2389.270273 | 0.348465195 | -1.520913531 | 8.52E-168 | 5.49E-166 | Down |
| DYDC2 | 6.637037394 | 11.19935097 | 2.07472382 | 0.185164548 | -2.433120195 | 0.034737634 | 0.049762028 | Down |
| DYNC1LI1 | 2247.522997 | 1493.183248 | 3001.862745 | 2.009875617 | 1.007106222 | 4.77E-35 | 3.29E-34 | Up |
| DYNLT2 | 29.73025923 | 46.00581436 | 13.4547041 | 0.292409399 | -1.77393841 | 0.001365452 | 0.00232509 | Down |
| DYRK3 | 3174.981358 | 4599.687856 | 1750.274859 | 0.380545352 | -1.393859694 | 6.97E-110 | 2.28E-108 | Down |
| DZIP1L | 3.28932216 | 0 | 6.57864432 | 32.29182584 | 5.013097111 | 0.006638692 | 0.010480912 | Up |
| E2F1 | 10361.41063 | 18034.08981 | 2688.731459 | 0.149088239 | -2.745761639 | 0 | 0 | Down |
| E2F2 | 17076.13734 | 29164.78167 | 4987.493003 | 0.171011118 | -2.547837972 | 0 | 0 | Down |
| E2F7 | 1356.137786 | 2007.567442 | 704.7081299 | 0.351000072 | -1.510456769 | 2.26E-72 | 3.87E-71 | Down |
| EAPP | 1973.037688 | 1168.574232 | 2777.501144 | 2.377229398 | 1.249281127 | 3.75E-56 | 4.53E-55 | Up |
| EBF3 | 132.8464309 | 233.2394154 | 32.45344634 | 0.139163766 | -2.845144472 | 3.13E-28 | 1.76E-27 | Down |
| EBI3 | 19.90256309 | 5.737709596 | 34.06741658 | 6.016163559 | 2.588843789 | 0.001021462 | 0.001763811 | Up |
| EBPL | 701.8840116 | 979.065093 | 424.7029301 | 0.433854845 | -1.204715654 | 6.90E-24 | 3.33E-23 | Down |
| ECE1 | 937.9970362 | 367.7552783 | 1508.238794 | 4.104176154 | 2.037092654 | 6.33E-92 | 1.56E-90 | Up |
| ECHDC2 | 2.795296547 | 5.590593093 | 0 | 0.029456251 | -5.085282343 | 0.008644863 | 0.013426592 | Down |
| ECI1 | 1628.846211 | 2319.737402 | 937.95502 | 0.404360494 | -1.306286042 | 4.49E-35 | 3.10E-34 | Down |
| EDA | 798.1544523 | 1277.094894 | 319.2140108 | 0.249988075 | -2.000068817 | 4.27E-88 | 9.85E-87 | Down |
| EDEM1 | 15828.22135 | 24407.99886 | 7248.44385 | 0.296970101 | -1.751610407 | 2.21E-182 | 1.58E-180 | Down |
| EDEM2 | 46.64433981 | 64.67676918 | 28.61191044 | 0.442111167 | -1.177518919 | 0.002730443 | 0.004509344 | Down |
| EEF1A1 | 283747.3937 | 408053.4024 | 159441.3849 | 0.390736324 | -1.355732715 | 2.54E-150 | 1.33E-148 | Down |
| EEF1AKMT2 | 204.603082 | 314.7130683 | 94.49309579 | 0.300251981 | -1.73575433 | 6.83E-19 | 2.72E-18 | Down |
| EEF1AKNMT | 3361.067931 | 2075.314411 | 4646.82145 | 2.238750465 | 1.162693732 | 1.91E-70 | 3.13E-69 | Up |
| EEF1B2 | 20758.73629 | 29079.76141 | 12437.71117 | 0.427716003 | -1.225274909 | 1.59E-138 | 7.39E-137 | Down |
| EEF1G | 85339.71905 | 114208.2329 | 56471.20523 | 0.494459433 | -1.016075932 | 1.78E-75 | 3.25E-74 | Down |
| EEF2K | 3139.442855 | 4656.311736 | 1622.573973 | 0.348460406 | -1.520933356 | 3.31E-122 | 1.27E-120 | Down |
| EEPD1 | 132.7742086 | 196.96876 | 68.57965725 | 0.348232387 | -1.521877709 | 2.87E-10 | 7.71E-10 | Down |
| EFCAB5 | 196.5368688 | 106.559544 | 286.5141936 | 2.685512202 | 1.425197276 | 8.45E-12 | 2.46E-11 | Up |
| EFCAB6 | 22.21226327 | 0 | 44.42452655 | 218.1187805 | 7.768970184 | 1.43E-09 | 3.70E-09 | Up |
| EFCAB8 | 207.5607963 | 290.676389 | 124.4452035 | 0.428158206 | -1.223784117 | 1.60E-08 | 3.88E-08 | Down |
| EFEMP1 | 93.27190894 | 172.5731098 | 13.97070809 | 0.081035842 | -3.625296046 | 2.56E-25 | 1.30E-24 | Down |
| EFHB | 11.8210266 | 3.949819836 | 19.69223335 | 4.934549196 | 2.302918291 | 0.012988472 | 0.019709342 | Up |
| EFNA2 | 8.984919689 | 0.385822986 | 17.58401639 | 44.32790866 | 5.470143394 | 0.000109674 | 0.000207514 | Up |
| EFNA3 | 170.8812257 | 238.2689521 | 103.4934993 | 0.434388048 | -1.202943688 | 1.84E-09 | 4.71E-09 | Down |
| EFNA5 | 34.42588154 | 6.261179627 | 62.59058346 | 9.986781186 | 3.320019762 | 2.26E-10 | 6.12E-10 | Up |
| EFNB2 | 767.0737022 | 320.2414947 | 1213.90591 | 3.792483562 | 1.923142927 | 7.46E-70 | 1.21E-68 | Up |
| EFR3B | 36.85670355 | 49.54959933 | 24.16380777 | 0.487771412 | -1.03572289 | 0.013503175 | 0.020418897 | Down |
| EGFL6 | 3.888821571 | 0 | 7.777643142 | 38.17542636 | 5.254572365 | 0.002456234 | 0.004080779 | Up |
| EGFL7 | 21.36900384 | 6.972189936 | 35.76581774 | 5.116838481 | 2.355252694 | 0.000110784 | 0.000209472 | Up |
| EGFL8 | 298.1391589 | 420.1205105 | 176.1578073 | 0.419233517 | -1.254174033 | 5.55E-16 | 1.94E-15 | Down |
| EGR1 | 3440.143556 | 136.5572624 | 6743.729849 | 49.40440608 | 5.626567808 | 0 | 0 | Up |
| EGR2 | 489.3264215 | 81.83026917 | 896.8225738 | 10.92737757 | 3.449875309 | 7.83E-106 | 2.42E-104 | Up |
| EGR3 | 57.33952074 | 3.27565518 | 111.4033863 | 33.6441607 | 5.072284226 | 6.25E-18 | 2.39E-17 | Up |
| EGR4 | 34.12410282 | 2.13839811 | 66.10980753 | 30.52907576 | 4.932112009 | 0.000101698 | 0.000192975 | Up |
| EID2B | 160.8112205 | 238.7814377 | 82.84100325 | 0.346845554 | -1.527634702 | 5.82E-10 | 1.54E-09 | Down |
| EIF2AK3 | 7326.014483 | 2516.268098 | 12135.76087 | 4.822596242 | 2.26981003 | 0 | 0 | Up |
| EIF2B2 | 2678.669266 | 1515.221847 | 3842.116686 | 2.535850846 | 1.342469891 | 9.65E-90 | 2.28E-88 | Up |
| EIF2B4 | 2673.021695 | 1631.316146 | 3714.727244 | 2.277502262 | 1.187452487 | 9.88E-76 | 1.81E-74 | Up |
| EIF4EBP1 | 3634.763618 | 6140.607891 | 1128.919346 | 0.183848862 | -2.443407849 | 8.68E-285 | 1.37E-282 | Down |
| EIF4EBP2 | 10848.93987 | 14933.02776 | 6764.851984 | 0.453028505 | -1.142326267 | 5.11E-99 | 1.37E-97 | Down |
| EIF6 | 2567.233605 | 1466.056598 | 3668.410611 | 2.503314585 | 1.323839602 | 3.46E-52 | 3.81E-51 | Up |
| ELAPOR1 | 92.02982235 | 20.2201381 | 163.8395066 | 8.087916719 | 3.015768142 | 9.62E-22 | 4.24E-21 | Up |
| ELAVL3 | 7.053097456 | 0.722010783 | 13.38418413 | 18.25711837 | 4.190387169 | 0.002708765 | 0.00447514 | Up |
| ELK3 | 800.5847259 | 519.4158791 | 1081.753573 | 2.081591872 | 1.057687234 | 3.49E-23 | 1.63E-22 | Up |
| ELK4 | 3927.768264 | 5399.742634 | 2455.793893 | 0.454809042 | -1.136667158 | 3.50E-50 | 3.65E-49 | Down |
| ELL3 | 326.4438161 | 439.8248348 | 213.0627974 | 0.484667193 | -1.044933665 | 1.47E-11 | 4.22E-11 | Down |
| ELOVL2 | 913.8038916 | 1507.281525 | 320.3262581 | 0.212544067 | -2.234166108 | 3.03E-99 | 8.22E-98 | Down |
| ELOVL7 | 7.61125235 | 1.850056439 | 13.37244826 | 7.243317068 | 2.856650529 | 0.00761813 | 0.011929452 | Up |
| ELP3 | 2557.318954 | 1323.676592 | 3790.961316 | 2.862999269 | 1.517527302 | 2.94E-99 | 7.99E-98 | Up |
| EMB | 1357.571122 | 1856.67615 | 858.4660942 | 0.462366674 | -1.112890678 | 9.69E-43 | 8.35E-42 | Down |
| EMC3 | 1506.335992 | 976.006368 | 2036.665615 | 2.086010738 | 1.060746584 | 1.73E-37 | 1.29E-36 | Up |
| EMC6 | 1175.674177 | 755.0650007 | 1596.283353 | 2.1151783 | 1.080779281 | 3.13E-25 | 1.59E-24 | Up |
| EMC8 | 2028.031637 | 1349.720729 | 2706.342544 | 2.005537791 | 1.003989151 | 8.65E-41 | 7.04E-40 | Up |
| EME1 | 1348.234901 | 1867.321559 | 829.1482431 | 0.443987542 | -1.171408899 | 2.09E-43 | 1.84E-42 | Down |
| EMG1 | 3376.005451 | 1969.238205 | 4782.772696 | 2.428837351 | 1.280265882 | 8.10E-91 | 1.95E-89 | Up |
| EML3 | 2099.812706 | 3056.440908 | 1143.184503 | 0.3740313 | -1.418769092 | 7.26E-87 | 1.64E-85 | Down |
| EML6 | 1124.665161 | 1723.549638 | 525.7806851 | 0.305038008 | -1.712939082 | 6.99E-74 | 1.23E-72 | Down |
| EMP1 | 48.35915597 | 5.926780891 | 90.79153105 | 15.36402941 | 3.941484725 | 4.36E-14 | 1.40E-13 | Up |
| EMP2 | 1122.326252 | 1930.354801 | 314.2977028 | 0.162853672 | -2.618351841 | 1.33E-141 | 6.33E-140 | Down |
| EMP3 | 1090.103341 | 631.211697 | 1548.994986 | 2.454847335 | 1.295633307 | 2.40E-45 | 2.22E-44 | Up |
| ENC1 | 1914.229579 | 1149.38598 | 2679.073179 | 2.33011859 | 1.220403382 | 7.13E-55 | 8.39E-54 | Up |
| ENDOU | 13.10023209 | 4.670041558 | 21.53042263 | 4.565678665 | 2.190829325 | 0.013858742 | 0.020936028 | Up |
| ENG | 2303.074331 | 3637.218244 | 968.9304179 | 0.266414349 | -1.908256308 | 3.30E-102 | 9.45E-101 | Down |
| ENGASE | 558.1782232 | 817.8226071 | 298.5338392 | 0.365045823 | -1.453850523 | 1.30E-27 | 7.15E-27 | Down |
| ENO2 | 116.9917156 | 69.79479234 | 164.1886388 | 2.349571629 | 1.232397751 | 1.33E-06 | 2.86E-06 | Up |
| ENOX1 | 54.20161947 | 33.61878088 | 74.78445807 | 2.225641923 | 1.154221501 | 0.002760825 | 0.004555723 | Up |
| ENTPD1 | 372.2517463 | 528.5955896 | 215.907903 | 0.408323409 | -1.292215814 | 2.94E-14 | 9.55E-14 | Down |
| ENTPD5 | 810.7611326 | 1340.091449 | 281.4308165 | 0.210014668 | -2.251437999 | 1.08E-100 | 2.98E-99 | Down |
| EPAS1 | 110.5064729 | 68.29271443 | 152.7202313 | 2.23648665 | 1.161234146 | 2.25E-05 | 4.47E-05 | Up |
| EPB41L1 | 539.7822187 | 285.7218258 | 793.8426115 | 2.780809252 | 1.475504787 | 5.10E-27 | 2.75E-26 | Up |
| EPB41L4A | 24.71159017 | 5.952626069 | 43.47055428 | 7.336614229 | 2.875114428 | 2.38E-06 | 5.03E-06 | Up |
| EPC1 | 4530.548263 | 2045.929191 | 7015.167336 | 3.428639068 | 1.77763604 | 9.46E-210 | 8.99E-208 | Up |
| EPC2 | 1881.229815 | 1222.4986 | 2539.961031 | 2.077603886 | 1.054920618 | 5.92E-29 | 3.42E-28 | Up |
| EPHA4 | 20.93630715 | 10.86137411 | 31.01124019 | 2.838167916 | 1.504959947 | 0.017344675 | 0.025894735 | Up |
| EPHB1 | 4.987010552 | 0.722010783 | 9.252010321 | 12.62400169 | 3.658097398 | 0.011686892 | 0.017837686 | Up |
| EPHB2 | 13.48370622 | 0.722010783 | 26.24540166 | 35.68686682 | 5.157321339 | 1.79E-05 | 3.57E-05 | Up |
| EPM2A | 560.5865338 | 754.6182345 | 366.5548331 | 0.48583775 | -1.041453502 | 9.55E-20 | 3.92E-19 | Down |
| EPM2AIP1 | 1766.633445 | 2467.552246 | 1065.714644 | 0.431893146 | -1.211253672 | 5.76E-26 | 3.01E-25 | Down |
| EPS15L1 | 2890.01418 | 1668.550921 | 4111.477439 | 2.463941012 | 1.300967717 | 4.41E-80 | 8.95E-79 | Up |
| EPS8L2 | 31.61446341 | 6.03526268 | 57.19366413 | 9.594104806 | 3.2621482 | 4.24E-08 | 1.00E-07 | Up |
| ERAL1 | 3610.469661 | 2401.307827 | 4819.631495 | 2.007211797 | 1.005192855 | 7.81E-48 | 7.74E-47 | Up |
| ERAP1 | 4399.663605 | 6527.078184 | 2272.249027 | 0.348154337 | -1.522201099 | 7.74E-119 | 2.83E-117 | Down |
| ERBB3 | 36.3531578 | 62.87455887 | 9.831756739 | 0.156327704 | -2.677354621 | 1.05E-08 | 2.57E-08 | Down |
| ERCC6L | 1103.941113 | 1515.970726 | 691.9114998 | 0.456408266 | -1.131603173 | 7.87E-32 | 4.97E-31 | Down |
| ERF | 2431.727861 | 1571.501843 | 3291.95388 | 2.095332828 | 1.067179424 | 1.35E-44 | 1.23E-43 | Up |
| ERG | 3773.610228 | 6334.1728 | 1213.047656 | 0.191522193 | -2.384416516 | 1.31E-304 | 2.24E-302 | Down |
| ERGIC1 | 29312.3154 | 42461.68801 | 16162.9428 | 0.380647777 | -1.39347144 | 1.52E-164 | 9.36E-163 | Down |
| ERMP1 | 2199.1319 | 3673.050018 | 725.2137827 | 0.197461063 | -2.340359893 | 7.56E-217 | 7.66E-215 | Down |
| ERO1A | 2264.648111 | 3102.821729 | 1426.474492 | 0.459761127 | -1.121043604 | 2.11E-33 | 1.38E-32 | Down |
| ERP27 | 63.0622452 | 102.6514048 | 23.47308564 | 0.228831036 | -2.12764536 | 2.02E-08 | 4.87E-08 | Down |
| ERP44 | 2745.183058 | 1830.463221 | 3659.902895 | 2.000106509 | 1.000076828 | 3.78E-43 | 3.30E-42 | Up |
| ERVH48-1 | 218.0797236 | 416.8169135 | 19.34253376 | 0.046434405 | -4.42866205 | 4.19E-58 | 5.26E-57 | Down |
| ESAM | 1573.502683 | 934.0977653 | 2212.9076 | 2.36961143 | 1.244650505 | 4.73E-42 | 4.01E-41 | Up |
| ESM1 | 148.4274526 | 3.384136969 | 293.4707683 | 88.26345752 | 6.463744358 | 6.68E-35 | 4.57E-34 | Up |
| ESPNL | 23.19736702 | 8.280353518 | 38.11438051 | 4.641843545 | 2.214697897 | 0.000329691 | 0.00059647 | Up |
| ESR2 | 32.43201129 | 50.00909749 | 14.8549251 | 0.297747671 | -1.747837871 | 0.004492079 | 0.007240254 | Down |
| ESRRB | 39.36588385 | 5.458321294 | 73.27344641 | 13.30537959 | 3.733937765 | 8.81E-12 | 2.56E-11 | Up |
| ETAA1 | 774.8246238 | 1040.322702 | 509.3265457 | 0.489686095 | -1.030070864 | 1.24E-13 | 3.91E-13 | Down |
| ETS1 | 2231.832063 | 3405.020625 | 1058.643502 | 0.310917293 | -1.685397233 | 6.88E-95 | 1.76E-93 | Down |
| ETV3 | 2153.833331 | 1329.575997 | 2978.090666 | 2.239280406 | 1.163035196 | 7.23E-60 | 9.44E-59 | Up |
| ETV5 | 4214.205967 | 1499.02547 | 6929.386464 | 4.623746695 | 2.209062364 | 1.12E-305 | 1.95E-303 | Up |
| ETV7 | 12.29829148 | 2.294726026 | 22.30185693 | 9.920139948 | 3.310360474 | 0.000493674 | 0.000876792 | Up |
| EVA1A | 6.55397927 | 0 | 13.10795854 | 64.36373877 | 6.008176227 | 6.43E-05 | 0.000124024 | Up |
| EVI2A | 627.5182299 | 295.602295 | 959.4341649 | 3.245116529 | 1.698270285 | 3.41E-34 | 2.29E-33 | Up |
| EVL | 3235.453124 | 4326.798184 | 2144.108064 | 0.495579628 | -1.01281121 | 4.50E-55 | 5.32E-54 | Down |
| EXD3 | 141.9730534 | 232.2963883 | 51.64971851 | 0.2222201 | -2.169938777 | 1.59E-16 | 5.67E-16 | Down |
| EXOC6B | 1417.36162 | 714.7869185 | 2119.936321 | 2.966523119 | 1.568773026 | 2.00E-76 | 3.73E-75 | Up |
| EXOSC10 | 5857.062254 | 3640.534248 | 8073.59026 | 2.217554033 | 1.148969258 | 4.35E-88 | 1.00E-86 | Up |
| EYA4 | 70.72158986 | 30.7215344 | 110.7216453 | 3.592470761 | 1.844976415 | 1.21E-07 | 2.81E-07 | Up |
| F13B | 6.101183306 | 0 | 12.20236661 | 59.9219674 | 5.905013087 | 0.000163263 | 0.000304384 | Up |
| F2R | 78.38252817 | 145.1303179 | 11.63473841 | 0.080184006 | -3.64054169 | 1.27E-21 | 5.59E-21 | Down |
| F2RL3 | 1487.254542 | 2488.958593 | 485.5504916 | 0.195047046 | -2.358105948 | 5.76E-147 | 2.88E-145 | Down |
| F3 | 52.18617212 | 29.6469601 | 74.72538415 | 2.524701446 | 1.336112795 | 0.000448023 | 0.000799296 | Up |
| FABP12 | 3.43483229 | 0 | 6.869664581 | 33.72326509 | 5.07567232 | 0.004132174 | 0.006688143 | Up |
| FABP3 | 2.385160987 | 0 | 4.770321975 | 23.42166281 | 4.549771598 | 0.021134103 | 0.031202889 | Up |
| FADS1 | 4661.932502 | 6296.045092 | 3027.819912 | 0.480917547 | -1.056138528 | 2.35E-81 | 4.88E-80 | Down |
| FADS3 | 9067.992012 | 15039.75391 | 3096.23011 | 0.205869168 | -2.280200313 | 0 | 0 | Down |
| FAH | 61.35311959 | 89.58000448 | 33.1262347 | 0.36946499 | -1.436490434 | 0.000196064 | 0.000362518 | Down |
| FAHD1 | 608.6866696 | 359.0883196 | 858.2850196 | 2.389702015 | 1.256830732 | 3.38E-30 | 2.03E-29 | Up |
| FAHD2A | 1148.211552 | 1652.747973 | 643.6751315 | 0.389496023 | -1.360319496 | 1.89E-54 | 2.21E-53 | Down |
| FAM102A | 352.1172067 | 477.5139488 | 226.7204647 | 0.474628979 | -1.075127906 | 3.92E-12 | 1.16E-11 | Down |
| FAM102B | 589.8419909 | 889.6557197 | 290.0282621 | 0.325899372 | -1.617501524 | 6.74E-37 | 4.95E-36 | Down |
| FAM107B | 22413.23519 | 13789.24276 | 31037.22762 | 2.250874445 | 1.170485585 | 3.69E-146 | 1.84E-144 | Up |
| FAM114A1 | 31.08209007 | 50.19023841 | 11.97394172 | 0.238086645 | -2.070441396 | 0.000224819 | 0.000413316 | Down |
| FAM117B | 805.046242 | 1249.194866 | 360.8976177 | 0.288855688 | -1.791579189 | 4.31E-68 | 6.72E-67 | Down |
| FAM135B | 10.92613416 | 1.908903041 | 19.94336528 | 10.66987456 | 3.41547131 | 0.000525954 | 0.000931499 | Up |
| FAM13B | 2795.496897 | 1272.953189 | 4318.040604 | 3.391953682 | 1.76211647 | 1.54E-119 | 5.70E-118 | Up |
| FAM161A | 394.5968112 | 571.7813947 | 217.4122277 | 0.380355755 | -1.394578662 | 8.33E-23 | 3.82E-22 | Down |
| FAM161B | 264.1343659 | 159.2307838 | 369.0379481 | 2.317080678 | 1.212308278 | 3.90E-11 | 1.10E-10 | Up |
| FAM163B | 30.5483236 | 10.15778615 | 50.93886104 | 4.979644058 | 2.316042623 | 2.08E-05 | 4.15E-05 | Up |
| FAM167A | 134.7512912 | 82.87620237 | 186.6263801 | 2.250703922 | 1.170376284 | 2.20E-07 | 5.01E-07 | Up |
| FAM171A2 | 684.2930524 | 914.3911197 | 454.194985 | 0.496628667 | -1.009760555 | 4.75E-20 | 1.97E-19 | Down |
| FAM184B | 4.79878131 | 0.385822986 | 9.211739633 | 23.22689912 | 4.537724657 | 0.010776547 | 0.016535562 | Up |
| FAM186B | 33.52022458 | 4.499393089 | 62.54105607 | 14.05358564 | 3.812866363 | 1.41E-10 | 3.85E-10 | Up |
| FAM189B | 1250.576802 | 1681.395341 | 819.7582639 | 0.487625626 | -1.03615415 | 1.12E-25 | 5.79E-25 | Down |
| FAM200A | 449.5908763 | 144.1000277 | 755.0817249 | 5.247263642 | 2.391565278 | 5.73E-46 | 5.38E-45 | Up |
| FAM209A | 60.59864057 | 18.27312449 | 102.9241567 | 5.656244979 | 2.499844606 | 2.94E-09 | 7.46E-09 | Up |
| FAM209B | 75.11735195 | 18.41792778 | 131.8167761 | 7.150875951 | 2.838119977 | 2.73E-17 | 1.01E-16 | Up |
| FAM20A | 6.893967636 | 1.868479266 | 11.91945601 | 6.421187179 | 2.682840054 | 0.017063839 | 0.025505659 | Up |
| FAM20B | 2549.007419 | 3721.663481 | 1376.351356 | 0.369838367 | -1.435033196 | 1.21E-70 | 2.01E-69 | Down |
| FAM20C | 157.2244848 | 247.7262123 | 66.72275727 | 0.269312619 | -1.892646265 | 1.79E-18 | 7.03E-18 | Down |
| FAM210B | 141.4087331 | 197.852224 | 84.96524223 | 0.429226611 | -1.220188573 | 5.06E-07 | 1.12E-06 | Down |
| FAM214A | 2485.454396 | 1598.560924 | 3372.347868 | 2.109394091 | 1.076828654 | 1.29E-31 | 8.07E-31 | Up |
| FAM217B | 1226.447485 | 1675.405344 | 777.4896257 | 0.464017451 | -1.107749032 | 3.99E-37 | 2.95E-36 | Down |
| FAM228A | 4.257740939 | 8.515481878 | 0 | 0.019324464 | -5.693427816 | 0.000626776 | 0.001101344 | Down |
| FAM53A | 84.76591045 | 43.5158596 | 126.0159613 | 2.895836075 | 1.533979938 | 3.26E-07 | 7.35E-07 | Up |
| FAM53B | 788.0569093 | 1152.644777 | 423.4690421 | 0.367326577 | -1.444864814 | 5.97E-40 | 4.75E-39 | Down |
| FAM53C | 7431.119925 | 3851.439107 | 11010.80074 | 2.859053307 | 1.515537519 | 1.65E-153 | 8.95E-152 | Up |
| FAM71F1 | 21.37510961 | 2.193666589 | 40.55655264 | 18.37385951 | 4.199582797 | 6.44E-07 | 1.42E-06 | Up |
| FAM71F2 | 108.2727614 | 26.22597747 | 190.3195453 | 7.261583899 | 2.860284264 | 1.68E-21 | 7.33E-21 | Up |
| FAM72A | 512.0199246 | 683.4906903 | 340.5491589 | 0.498355281 | -1.00475348 | 3.11E-08 | 7.44E-08 | Down |
| FAM72B | 993.595328 | 1341.819508 | 645.371148 | 0.480847237 | -1.056349465 | 8.36E-28 | 4.63E-27 | Down |
| FAM72C | 185.0957422 | 254.7853992 | 115.4060852 | 0.452975059 | -1.142496477 | 2.92E-09 | 7.42E-09 | Down |
| FAM72D | 305.8653699 | 414.7593802 | 196.9713596 | 0.475066052 | -1.07379998 | 3.99E-10 | 1.06E-09 | Down |
| FAM78A | 53.58580457 | 91.38246477 | 15.78914437 | 0.172871358 | -2.532229237 | 3.44E-09 | 8.71E-09 | Down |
| FAM83C | 9.324298063 | 1.096833294 | 17.55176283 | 15.90554923 | 3.991458285 | 0.000731012 | 0.001276579 | Up |
| FAM83E | 36.86344249 | 4.916428437 | 68.81045654 | 14.22432665 | 3.830288455 | 5.98E-11 | 1.66E-10 | Up |
| FANCA | 2806.644383 | 4080.335666 | 1532.9531 | 0.375699131 | -1.412350317 | 8.05E-83 | 1.73E-81 | Down |
| FANCD2 | 3779.680587 | 5974.870371 | 1584.490804 | 0.265176242 | -1.914976567 | 5.45E-189 | 4.27E-187 | Down |
| FANCE | 1575.377945 | 2162.972025 | 987.7838646 | 0.456730003 | -1.130586531 | 1.59E-43 | 1.40E-42 | Down |
| FANCG | 3055.034063 | 4112.22184 | 1997.846285 | 0.485784604 | -1.041611326 | 4.51E-61 | 6.01E-60 | Down |
| FANCL | 218.5409462 | 292.2230091 | 144.8588832 | 0.495497496 | -1.013050328 | 1.92E-06 | 4.10E-06 | Down |
| FANK1 | 26.20208 | 38.09233685 | 14.31182315 | 0.375211562 | -1.414223808 | 0.005322251 | 0.008516952 | Down |
| FAP | 4.82579472 | 0.365611098 | 9.285978342 | 23.39822524 | 4.5483272 | 0.006259713 | 0.009920878 | Up |
| FAR2 | 435.0975709 | 701.8994228 | 168.2957189 | 0.23982354 | -2.059954822 | 2.36E-52 | 2.61E-51 | Down |
| FARSA | 8082.396151 | 5025.055731 | 11139.73657 | 2.216991721 | 1.148603383 | 7.49E-79 | 1.47E-77 | Up |
| FAS | 281.4675462 | 131.8488442 | 431.0862481 | 3.274078175 | 1.71108877 | 4.94E-22 | 2.20E-21 | Up |
| FASTKD2 | 1188.842211 | 724.3498231 | 1653.334598 | 2.28182651 | 1.190189106 | 2.15E-38 | 1.64E-37 | Up |
| FAT2 | 3.750907488 | 0.356399685 | 7.14541529 | 18.015123 | 4.171136597 | 0.013393809 | 0.020275622 | Up |
| FBLL1 | 25.12888121 | 5.232404347 | 45.02535807 | 8.667186166 | 3.115563693 | 9.61E-07 | 2.09E-06 | Up |
| FBLN5 | 4.239919827 | 0.751434084 | 7.728405569 | 10.40903853 | 3.37976491 | 0.0254827 | 0.037238783 | Up |
| FBN2 | 27.75249259 | 12.04263308 | 43.4623521 | 3.596154847 | 1.846455143 | 0.00104253 | 0.001796612 | Up |
| FBP2 | 2.088035354 | 0 | 4.176070708 | 20.50288462 | 4.357754997 | 0.032012881 | 0.046063348 | Up |
| FBRS | 6251.403421 | 3918.299042 | 8584.5078 | 2.191071773 | 1.131636743 | 6.49E-77 | 1.22E-75 | Up |
| FBRSL1 | 4766.473811 | 7313.880659 | 2219.066962 | 0.303416792 | -1.720627165 | 9.26E-149 | 4.74E-147 | Down |
| FBXL16 | 32.74847623 | 57.7580506 | 7.738901856 | 0.134158557 | -2.897989016 | 5.26E-09 | 1.32E-08 | Down |
| FBXL3 | 2039.616282 | 1333.138479 | 2746.094085 | 2.059480641 | 1.042280565 | 4.14E-28 | 2.32E-27 | Up |
| FBXL8 | 107.3099345 | 181.5762724 | 33.04359651 | 0.181987638 | -2.458087639 | 8.54E-17 | 3.10E-16 | Down |
| FBXO24 | 40.74842438 | 57.68742133 | 23.80942743 | 0.412303491 | -1.27822142 | 0.005400747 | 0.008633601 | Down |
| FBXO28 | 2796.331023 | 1624.257774 | 3968.404272 | 2.443184027 | 1.288762535 | 8.05E-74 | 1.41E-72 | Up |
| FBXO32 | 41.29998806 | 7.736413555 | 74.86356257 | 9.668843891 | 3.273343396 | 1.99E-11 | 5.66E-11 | Up |
| FBXO36 | 193.2899942 | 273.7787534 | 112.8012349 | 0.411913404 | -1.279587021 | 1.14E-09 | 2.96E-09 | Down |
| FBXO39 | 23.3898056 | 8.072601353 | 38.70700985 | 4.783599113 | 2.25809649 | 6.84E-05 | 0.000131715 | Up |
| FBXO43 | 305.6032156 | 443.8491109 | 167.3573203 | 0.376892136 | -1.407776402 | 1.83E-18 | 7.17E-18 | Down |
| FBXO6 | 24.73967217 | 6.205911148 | 43.27343319 | 6.940500847 | 2.795039776 | 2.81E-05 | 5.54E-05 | Up |
| FBXO8 | 740.3140792 | 481.3502272 | 999.2779312 | 2.076727664 | 1.054312038 | 3.97E-21 | 1.71E-20 | Up |
| FBXW4 | 2566.905732 | 1348.731844 | 3785.079619 | 2.805669402 | 1.488345023 | 3.38E-87 | 7.67E-86 | Up |
| FCER2 | 72.22618538 | 23.71070975 | 120.741661 | 5.069628123 | 2.341879924 | 2.51E-10 | 6.78E-10 | Up |
| FCGBP | 51.04942006 | 7.745624968 | 94.35321514 | 12.18271441 | 3.606763708 | 1.31E-14 | 4.33E-14 | Up |
| FCGR1A | 4.056525126 | 0.356399685 | 7.756650568 | 19.55168771 | 4.289221242 | 0.009257704 | 0.014311113 | Up |
| FCGR2A | 854.1636617 | 361.255883 | 1347.07144 | 3.726699719 | 1.897898578 | 7.83E-73 | 1.36E-71 | Up |
| FCGR2B | 8.89897926 | 2.285514613 | 15.51244391 | 6.89453761 | 2.785453799 | 0.007804452 | 0.012200558 | Up |
| FCHSD2 | 1726.524199 | 2533.57122 | 919.4771769 | 0.362887675 | -1.462405035 | 7.95E-80 | 1.60E-78 | Down |
| FCMR | 200.6877374 | 19.62324287 | 381.7522319 | 19.50490135 | 4.285764797 | 1.87E-57 | 2.31E-56 | Up |
| FDX2 | 769.209766 | 272.1911774 | 1266.228355 | 4.656030215 | 2.219100421 | 5.27E-80 | 1.07E-78 | Up |
| FEZ1 | 55.54215649 | 16.37880005 | 94.70551292 | 5.80236697 | 2.536641542 | 7.68E-09 | 1.90E-08 | Up |
| FGD1 | 36.01216793 | 10.21663275 | 61.80770311 | 6.018220035 | 2.589336855 | 4.64E-07 | 1.03E-06 | Up |
| FGD2 | 134.0188934 | 43.72924506 | 224.3085418 | 5.139308647 | 2.361574297 | 1.11E-19 | 4.57E-19 | Up |
| FGD5 | 33.96865393 | 2.888043133 | 65.04926472 | 22.15474117 | 4.469543567 | 1.70E-11 | 4.84E-11 | Up |
| FGD6 | 879.3094475 | 1301.469491 | 457.1494037 | 0.35120266 | -1.509624323 | 6.12E-40 | 4.87E-39 | Down |
| FGF18 | 13.10641857 | 6.005839379 | 20.20699777 | 3.385922694 | 1.759549034 | 0.030495216 | 0.044053233 | Up |
| FGF8 | 11.72414518 | 2.561066748 | 20.88722361 | 8.114917797 | 3.020576481 | 0.000513017 | 0.000910098 | Up |
| FGFR4 | 93.69157699 | 24.38692151 | 162.9962325 | 6.689763281 | 2.741955162 | 8.18E-18 | 3.11E-17 | Up |
| FGR | 10.36922587 | 0.742222671 | 19.99622906 | 27.07088875 | 4.758670348 | 0.000143913 | 0.000269869 | Up |
| FHAD1 | 30.37914775 | 41.71928258 | 19.03901293 | 0.456679827 | -1.130745031 | 0.015193876 | 0.022858379 | Down |
| FHDC1 | 89.0682794 | 20.42942128 | 157.7071375 | 7.675844703 | 2.940325523 | 2.53E-18 | 9.83E-18 | Up |
| FHL1 | 756.7635121 | 1114.755267 | 398.7717572 | 0.357758001 | -1.482944062 | 3.68E-39 | 2.86E-38 | Down |
| FIBCD1 | 4.2026324 | 0.365611098 | 8.039653702 | 20.26938097 | 4.341230124 | 0.008111829 | 0.012649711 | Up |
| FIGNL1 | 2347.773748 | 3417.247884 | 1278.299611 | 0.374089659 | -1.418544007 | 5.15E-54 | 5.94E-53 | Down |
| FKBP11 | 933.1871888 | 1331.90736 | 534.4670177 | 0.401204059 | -1.317591892 | 2.53E-34 | 1.71E-33 | Down |
| FKRP | 971.931993 | 511.355118 | 1432.508868 | 2.802096558 | 1.486506671 | 3.44E-48 | 3.44E-47 | Up |
| FKTN | 1090.844227 | 1476.951655 | 704.7367988 | 0.477190348 | -1.067363231 | 6.42E-17 | 2.34E-16 | Down |
| FLACC1 | 18.55055202 | 8.755977422 | 28.34512662 | 3.223008276 | 1.688407893 | 0.006674792 | 0.01053311 | Up |
| FLNB | 11542.34504 | 16807.83053 | 6276.859546 | 0.373454115 | -1.420997101 | 9.83E-166 | 6.12E-164 | Down |
| FLRT1 | 600.3767863 | 1156.727601 | 44.02597173 | 0.038108423 | -4.713746279 | 1.71E-167 | 1.10E-165 | Down |
| FLT1 | 1189.672471 | 1666.339024 | 713.0059189 | 0.427827066 | -1.22490034 | 5.35E-43 | 4.65E-42 | Down |
| FLVCR2 | 4.362333941 | 0.356399685 | 8.368268196 | 21.08952005 | 4.398454358 | 0.007576232 | 0.011866521 | Up |
| FMN2 | 5.368674333 | 0 | 10.73734867 | 52.71703347 | 5.720197283 | 0.000286594 | 0.000522042 | Up |
| FN1 | 5.759791983 | 10.04035099 | 1.479232973 | 0.148008479 | -2.756248273 | 0.022387938 | 0.032934892 | Down |
| FNDC5 | 40.87029338 | 73.97105328 | 7.769533487 | 0.104869351 | -3.253335002 | 4.66E-11 | 1.30E-10 | Down |
| FNDC7 | 7.741651049 | 2.928466908 | 12.55483519 | 4.271150718 | 2.094624808 | 0.033963844 | 0.048688764 | Up |
| FOS | 1021.957184 | 75.23368394 | 1968.680684 | 26.25693085 | 4.714626385 | 2.21E-266 | 3.07E-264 | Up |
| FOSB | 1155.375935 | 165.7828349 | 2144.969035 | 12.90141166 | 3.689457027 | 6.08E-209 | 5.66E-207 | Up |
| FOSL1 | 55.29524981 | 15.39197956 | 95.19852006 | 6.164179312 | 2.623908829 | 4.89E-11 | 1.36E-10 | Up |
| FOSL2 | 4590.031868 | 538.6799103 | 8641.383826 | 16.04807384 | 4.004328244 | 0 | 0 | Up |
| FOXD2 | 43.19285626 | 69.06971952 | 17.31599299 | 0.250780092 | -1.99550527 | 1.78E-05 | 3.56E-05 | Down |
| FOXD4 | 69.56033222 | 23.73528195 | 115.3853825 | 4.89243666 | 2.290553173 | 1.27E-07 | 2.94E-07 | Up |
| FOXD4L1 | 85.77142121 | 21.96734604 | 149.5754964 | 6.782959426 | 2.761914863 | 2.23E-17 | 8.28E-17 | Up |
| FOXI1 | 2.525278063 | 0 | 5.050556126 | 24.80591705 | 4.632612388 | 0.017999223 | 0.026819983 | Up |
| FOXL1 | 1134.376022 | 1524.062307 | 744.6897359 | 0.48866329 | -1.033087366 | 2.60E-32 | 1.66E-31 | Down |
| FOXM1 | 5600.163154 | 7613.001947 | 3587.324362 | 0.471191125 | -1.085615728 | 8.19E-78 | 1.57E-76 | Down |
| FOXO3 | 1743.819157 | 1109.303844 | 2378.334469 | 2.144241546 | 1.100467433 | 1.09E-46 | 1.05E-45 | Up |
| FOXO6 | 2422.562786 | 3857.274168 | 987.8514041 | 0.256124195 | -1.965084553 | 6.59E-152 | 3.53E-150 | Down |
| FOXP2 | 5.185071895 | 10.06977429 | 0.300369495 | 0.031860859 | -4.972071043 | 0.001632799 | 0.002759327 | Down |
| FOXS1 | 5.644323876 | 10.38753927 | 0.901108486 | 0.086432922 | -3.532275254 | 0.008634245 | 0.013411603 | Down |
| FPGS | 6568.304014 | 9590.60672 | 3546.001309 | 0.369751458 | -1.435372258 | 2.06E-161 | 1.21E-159 | Down |
| FPR1 | 71.88351542 | 0.365611098 | 143.4014197 | 361.4911513 | 8.497816523 | 2.32E-12 | 6.92E-12 | Up |
| FPR2 | 13.13540011 | 0.356399685 | 25.91440053 | 65.33191181 | 6.029715953 | 8.02E-06 | 1.64E-05 | Up |
| FRAS1 | 9.097416773 | 1.484445341 | 16.7103882 | 11.30034203 | 3.498294534 | 0.001084404 | 0.001864836 | Up |
| FRAT1 | 364.3201735 | 490.4881995 | 238.1521474 | 0.485522625 | -1.042389568 | 8.92E-11 | 2.46E-10 | Down |
| FRMD3 | 28.02734782 | 8.022966165 | 48.03172948 | 5.951606362 | 2.57327911 | 2.32E-06 | 4.90E-06 | Up |
| FRMD4B | 3414.121094 | 1294.535912 | 5533.706275 | 4.274042326 | 2.095601195 | 5.42E-133 | 2.34E-131 | Up |
| FRMPD1 | 29.7853468 | 46.72398092 | 12.84671268 | 0.274947508 | -1.862771886 | 0.000255449 | 0.000467643 | Down |
| FRMPD2 | 8.191269206 | 0 | 16.38253841 | 80.44407503 | 6.329914261 | 1.17E-05 | 2.37E-05 | Up |
| FRY | 315.6556669 | 485.238355 | 146.0729787 | 0.301034009 | -1.73200161 | 3.33E-22 | 1.49E-21 | Down |
| FSD2 | 35.32968197 | 11.57264246 | 59.08672147 | 5.133200691 | 2.359858666 | 1.13E-06 | 2.45E-06 | Up |
| FSTL4 | 289.4308947 | 34.4989167 | 544.3628727 | 15.72401286 | 3.974897544 | 9.76E-80 | 1.94E-78 | Up |
| FTL | 17246.39685 | 22998.23759 | 11494.55611 | 0.499814999 | -1.0005339 | 4.68E-70 | 7.64E-69 | Down |
| FUT10 | 668.6347598 | 936.4377408 | 400.8317788 | 0.427957716 | -1.224459837 | 1.49E-26 | 7.92E-26 | Down |
| FUT5 | 16.11156751 | 25.38448443 | 6.8386506 | 0.26951442 | -1.891565632 | 0.009141626 | 0.014141127 | Down |
| FXYD1 | 9.400541088 | 15.2451211 | 3.555961074 | 0.23394867 | -2.095736065 | 0.029092791 | 0.042163422 | Down |
| FZD2 | 916.2361083 | 1386.588747 | 445.8834701 | 0.321573038 | -1.636781646 | 3.96E-69 | 6.31E-68 | Down |
| FZD3 | 394.2169662 | 534.5259486 | 253.9079837 | 0.474873155 | -1.074385893 | 2.16E-15 | 7.36E-15 | Down |
| FZD5 | 398.6660357 | 563.9587503 | 233.3733212 | 0.413938866 | -1.27251038 | 3.93E-20 | 1.63E-19 | Down |
| GAA | 1393.215675 | 1962.21078 | 824.2205693 | 0.420101465 | -1.251190278 | 8.48E-52 | 9.24E-51 | Down |
| GAB1 | 7377.796734 | 11903.53505 | 2852.058421 | 0.239589287 | -2.061364693 | 2.37E-304 | 4.01E-302 | Down |
| GAB3 | 1097.476119 | 1545.242422 | 649.7098166 | 0.420528816 | -1.249723432 | 3.22E-41 | 2.65E-40 | Down |
| GABARAPL1 | 419.2586405 | 85.97968612 | 752.5375949 | 8.753785439 | 3.129907023 | 2.05E-74 | 3.65E-73 | Up |
| GABBR1 | 812.6948015 | 1113.134987 | 512.2546161 | 0.4600465 | -1.120148403 | 4.36E-23 | 2.03E-22 | Down |
| GAD1 | 14.64491071 | 2.204667063 | 27.08515436 | 12.25468419 | 3.6152614 | 2.51E-05 | 4.97E-05 | Up |
| GADD45B | 1486.595756 | 379.4545673 | 2593.736944 | 6.839919081 | 2.773979258 | 5.95E-209 | 5.58E-207 | Up |
| GADD45G | 22.77209384 | 0.71279937 | 44.83138831 | 61.00987941 | 5.930970974 | 3.48E-07 | 7.82E-07 | Up |
| GADD45GIP1 | 2276.712459 | 1443.294515 | 3110.130402 | 2.155616883 | 1.108100791 | 2.19E-46 | 2.08E-45 | Up |
| GADL1 | 6.917986942 | 12.34786656 | 1.488107328 | 0.120837274 | -3.048862556 | 0.007576085 | 0.011866521 | Down |
| GAL3ST1 | 82.08987447 | 3.808594668 | 160.3711543 | 43.23666538 | 5.434183355 | 2.20E-23 | 1.04E-22 | Up |
| GAL3ST4 | 275.695538 | 462.3323088 | 89.05876728 | 0.192656965 | -2.375893754 | 8.37E-29 | 4.80E-28 | Down |
| GALC | 50.51941331 | 0 | 101.0388266 | 496.1050388 | 8.9545018 | 2.63E-13 | 8.16E-13 | Up |
| GALNT12 | 100.6541886 | 167.0551931 | 34.25318415 | 0.205164768 | -2.285145087 | 2.02E-17 | 7.51E-17 | Down |
| GALNT14 | 1991.986238 | 2756.156515 | 1227.815961 | 0.445512934 | -1.16646078 | 1.81E-55 | 2.15E-54 | Down |
| GALNT6 | 240.7059929 | 138.9637984 | 342.4481875 | 2.461747262 | 1.299682654 | 5.71E-13 | 1.75E-12 | Up |
| GALNT8 | 12.12393384 | 0.722010783 | 23.52585689 | 32.01537658 | 5.000693075 | 5.73E-05 | 0.000110765 | Up |
| GALNT9 | 102.7500192 | 0 | 205.5000383 | 1008.944719 | 9.978631414 | 1.08E-16 | 3.89E-16 | Up |
| GALR2 | 21.74873534 | 3.838017968 | 39.65945272 | 10.55148985 | 3.399374814 | 8.87E-06 | 1.81E-05 | Up |
| GAREM1 | 172.9421184 | 262.4854417 | 83.3987951 | 0.317705992 | -1.654235794 | 1.18E-14 | 3.89E-14 | Down |
| GARNL3 | 18.99700698 | 27.87238402 | 10.12162995 | 0.363746949 | -1.458992947 | 0.01470832 | 0.022161536 | Down |
| GAS1 | 214.7554754 | 357.3469949 | 72.16395585 | 0.201960349 | -2.307856017 | 3.88E-33 | 2.52E-32 | Down |
| GATA2 | 4.990666947 | 1.069199055 | 8.912134839 | 8.184282653 | 3.032855971 | 0.03138631 | 0.045232157 | Up |
| GATA3 | 1198.047477 | 731.8137305 | 1664.281223 | 2.273501985 | 1.184916264 | 3.82E-45 | 3.52E-44 | Up |
| GATM | 988.8829007 | 1682.671991 | 295.0938101 | 0.175346246 | -2.51172155 | 1.98E-144 | 9.70E-143 | Down |
| GBP2 | 2072.8772 | 417.3174172 | 3728.436983 | 8.935431318 | 3.159537371 | 7.03E-308 | 1.27E-305 | Up |
| GBP5 | 14.29185301 | 1.811421726 | 26.77228429 | 14.6058406 | 3.868473485 | 2.93E-05 | 5.77E-05 | Up |
| GBP6 | 13.72358473 | 0 | 27.44716946 | 134.7543946 | 7.074188512 | 1.81E-07 | 4.14E-07 | Up |
| GCAT | 1314.310223 | 1866.881083 | 761.7393641 | 0.408050481 | -1.293180451 | 1.03E-33 | 6.78E-33 | Down |
| GCNT1 | 1747.440676 | 2440.977315 | 1053.904037 | 0.431698581 | -1.211903744 | 8.49E-60 | 1.11E-58 | Down |
| GCSH | 365.668667 | 495.968182 | 235.3691521 | 0.474460451 | -1.075640258 | 2.24E-12 | 6.67E-12 | Down |
| GDAP1L1 | 9.035369755 | 18.07073951 | 0 | 0.009101884 | -6.779619053 | 1.94E-06 | 4.14E-06 | Down |
| GDF11 | 822.0249451 | 1097.863031 | 546.1868592 | 0.497461754 | -1.007342483 | 8.01E-20 | 3.30E-19 | Down |
| GDF15 | 12.1406573 | 1.107833769 | 23.17348084 | 20.94122975 | 4.38827426 | 7.09E-05 | 0.000136227 | Up |
| GDNF | 27.60016509 | 8.967573817 | 46.23275637 | 5.181741088 | 2.373436931 | 6.62E-05 | 0.000127552 | Up |
| GDPD1 | 394.652436 | 240.6414222 | 548.6634498 | 2.279369885 | 1.188635057 | 5.70E-15 | 1.91E-14 | Up |
| GFI1 | 1585.8875 | 2304.902675 | 866.8723248 | 0.376154364 | -1.410603267 | 1.45E-70 | 2.40E-69 | Down |
| GFRA3 | 17.30714722 | 5.636650159 | 28.97764429 | 5.178335393 | 2.372488409 | 0.001593488 | 0.00269585 | Up |
| GFY | 4.032564859 | 0 | 8.065129718 | 39.59158959 | 5.307122087 | 0.001854484 | 0.003121412 | Up |
| GGCT | 1097.749369 | 1508.017001 | 687.4817377 | 0.45588048 | -1.133272458 | 6.34E-35 | 4.35E-34 | Down |
| GGNBP2 | 4384.915508 | 2641.130915 | 6128.700102 | 2.320315072 | 1.21432072 | 8.83E-108 | 2.79E-106 | Up |
| GGT1 | 165.2857464 | 69.5466164 | 261.0248764 | 3.744008536 | 1.904583724 | 7.64E-15 | 2.54E-14 | Up |
| GHRL | 215.7697874 | 0.365611098 | 431.1739637 | 1086.900155 | 10.0860037 | 2.36E-17 | 8.77E-17 | Up |
| GIMAP6 | 21.07818598 | 9.09216522 | 33.06420674 | 3.616309768 | 1.854518262 | 0.001935255 | 0.003252626 | Up |
| GINS2 | 2232.291544 | 3478.408089 | 986.1749982 | 0.283484355 | -1.818658979 | 6.60E-89 | 1.54E-87 | Down |
| GINS4 | 2592.492254 | 3538.920004 | 1646.064504 | 0.465133122 | -1.104284417 | 1.80E-65 | 2.62E-64 | Down |
| GIPR | 18.03866893 | 31.29489766 | 4.782440193 | 0.152574444 | -2.712414764 | 4.95E-05 | 9.60E-05 | Down |
| GJA3 | 286.8625196 | 168.1791365 | 405.5459027 | 2.41113023 | 1.269709576 | 1.42E-15 | 4.90E-15 | Up |
| GJA5 | 18.54632723 | 5.469321768 | 31.6233327 | 5.747226915 | 2.522866011 | 0.000134106 | 0.000251902 | Up |
| GJC2 | 11.25165381 | 4.374277535 | 18.12903009 | 4.126716106 | 2.044994193 | 0.018275222 | 0.027207857 | Up |
| GK5 | 508.4286198 | 702.9819436 | 313.875296 | 0.446474127 | -1.163351519 | 6.32E-23 | 2.92E-22 | Down |
| GLB1L | 126.7636412 | 173.9263236 | 79.60095881 | 0.457733311 | -1.127420808 | 4.46E-07 | 9.95E-07 | Down |
| GLCCI1 | 872.2119428 | 1290.262235 | 454.1616504 | 0.351967438 | -1.506486129 | 1.09E-50 | 1.14E-49 | Down |
| GLDN | 26.34716994 | 37.79914409 | 14.89519578 | 0.394903648 | -1.3404274 | 0.017115339 | 0.025574369 | Down |
| GLIPR1 | 32.40034637 | 14.39826087 | 50.40243188 | 3.501302414 | 1.807891675 | 0.000203953 | 0.000376352 | Up |
| GLRX | 2658.161054 | 3732.85383 | 1583.468278 | 0.424223872 | -1.237102288 | 1.27E-83 | 2.77E-82 | Down |
| GLT1D1 | 11.15703384 | 0 | 22.31406768 | 109.5804015 | 6.775845985 | 1.42E-06 | 3.06E-06 | Up |
| GM2A | 1979.989406 | 2730.618532 | 1229.36028 | 0.450255104 | -1.151185464 | 1.29E-48 | 1.30E-47 | Down |
| GMPR | 4.817103547 | 0.385822986 | 9.248384109 | 23.31183462 | 4.542990642 | 0.004229056 | 0.006832196 | Up |
| GNA14 | 3.578384403 | 0 | 7.156768807 | 35.13821377 | 5.13496895 | 0.003661417 | 0.005957626 | Up |
| GNAZ | 78.2716479 | 129.1654992 | 27.37779655 | 0.212279943 | -2.235960029 | 2.74E-10 | 7.39E-10 | Down |
| GNB5 | 1259.173039 | 1688.829174 | 829.5169045 | 0.491292342 | -1.025346343 | 1.41E-32 | 9.03E-32 | Down |
| GNG4 | 4.49978068 | 0.356399685 | 8.643161676 | 21.78867451 | 4.445506529 | 0.007811196 | 0.012209725 | Up |
| GNG7 | 6010.461281 | 10951.07737 | 1069.845191 | 0.097700442 | -3.355491095 | 0 | 0 | Down |
| GNGT2 | 311.6249893 | 425.8600092 | 197.3899694 | 0.463428532 | -1.109581224 | 2.34E-13 | 7.28E-13 | Down |
| GNMT | 2.42239615 | 4.8447923 | 0 | 0.033989173 | -4.878780921 | 0.01532452 | 0.023042753 | Down |
| GNPTAB | 1904.133767 | 2599.80977 | 1208.457765 | 0.464808982 | -1.105290148 | 6.66E-53 | 7.46E-52 | Down |
| GNRH1 | 28.34042395 | 42.34917923 | 14.33166868 | 0.338145402 | -1.564284359 | 0.001808933 | 0.003045849 | Down |
| GNRH2 | 38.93610112 | 15.86070526 | 62.01149698 | 3.909345374 | 1.966927046 | 5.86E-05 | 0.000113175 | Up |
| GOLGA6L10 | 108.801553 | 160.1387543 | 57.46435175 | 0.358738378 | -1.478996003 | 1.90E-07 | 4.33E-07 | Down |
| GOLGA6L9 | 341.2631599 | 515.3248341 | 167.2014857 | 0.324409382 | -1.62411255 | 6.33E-23 | 2.93E-22 | Down |
| GOLGA7B | 887.6831787 | 1678.394827 | 96.97153004 | 0.057747649 | -4.114093982 | 2.47E-191 | 1.97E-189 | Down |
| GOLGA8B | 1565.534465 | 2141.539587 | 989.5293433 | 0.462092432 | -1.113746633 | 1.18E-24 | 5.79E-24 | Down |
| GOLGA8H | 20.25821873 | 35.15339362 | 5.363043839 | 0.152634854 | -2.711843657 | 0.000100968 | 0.000191643 | Down |
| GOLGA8S | 21.3698915 | 35.26365641 | 7.476126594 | 0.212140261 | -2.236909645 | 0.002491926 | 0.004136609 | Down |
| GOLGB1 | 2137.016917 | 1386.535807 | 2887.498027 | 2.082488239 | 1.058308348 | 2.96E-27 | 1.61E-26 | Up |
| GOLIM4 | 2327.418751 | 1355.467301 | 3299.370201 | 2.433861763 | 1.283247229 | 9.36E-46 | 8.75E-45 | Up |
| GORAB | 807.9804048 | 435.8343654 | 1180.126444 | 2.706947274 | 1.436666787 | 2.05E-34 | 1.39E-33 | Up |
| GOSR1 | 1809.192827 | 1072.807304 | 2545.578351 | 2.372867376 | 1.246631468 | 8.71E-67 | 1.31E-65 | Up |
| GOT1L1 | 2.026467221 | 4.052934441 | 0 | 0.040576591 | -4.623208529 | 0.03119079 | 0.044969073 | Down |
| GP1BB | 40.33917627 | 54.74104768 | 25.93730486 | 0.474006426 | -1.077021476 | 0.013643693 | 0.020617896 | Down |
| GP5 | 13.46373029 | 20.96978312 | 5.957677457 | 0.284036741 | -1.815850534 | 0.008825615 | 0.013685869 | Down |
| GPATCH1 | 1520.186853 | 977.1782484 | 2063.195457 | 2.111680255 | 1.078391402 | 1.93E-36 | 1.39E-35 | Up |
| GPATCH2 | 932.8469108 | 610.4890496 | 1255.204772 | 2.056108394 | 1.039916323 | 1.76E-23 | 8.35E-23 | Up |
| GPATCH3 | 1343.169199 | 834.024826 | 1852.313571 | 2.220677688 | 1.151000014 | 5.04E-43 | 4.39E-42 | Up |
| GPATCH4 | 2826.275859 | 1068.989458 | 4583.56226 | 4.289703605 | 2.100877969 | 2.61E-193 | 2.11E-191 | Up |
| GPBAR1 | 11.38206338 | 4.006877376 | 18.75724938 | 4.654561873 | 2.218645375 | 0.006509444 | 0.010288571 | Up |
| GPC2 | 404.6555942 | 625.2214592 | 184.0897291 | 0.294444066 | -1.763934496 | 7.07E-36 | 4.99E-35 | Down |
| GPC3 | 9.205808005 | 1.107833769 | 17.30378224 | 15.63010592 | 3.96625565 | 0.00040923 | 0.000733672 | Up |
| GPD1 | 65.62435787 | 16.47781238 | 114.7709033 | 6.937395656 | 2.794394167 | 1.76E-14 | 5.76E-14 | Up |
| GPD1L | 1138.585903 | 1664.802364 | 612.3694421 | 0.367826855 | -1.442901281 | 4.78E-58 | 5.99E-57 | Down |
| GPER1 | 1553.314215 | 2382.656145 | 723.9722843 | 0.303808379 | -1.718766435 | 1.12E-82 | 2.40E-81 | Down |
| GPIHBP1 | 28.48525743 | 45.34980643 | 11.62070844 | 0.256549708 | -1.962689709 | 0.000208087 | 0.000383418 | Down |
| GPLD1 | 230.0512201 | 347.763595 | 112.3388452 | 0.322994183 | -1.63041991 | 5.86E-20 | 2.42E-19 | Down |
| GPM6B | 493.7095118 | 793.0605314 | 194.3584921 | 0.245089772 | -2.028617813 | 2.27E-42 | 1.94E-41 | Down |
| GPNMB | 49.27294224 | 10.04214006 | 88.50374442 | 8.85581829 | 3.14662562 | 1.73E-12 | 5.18E-12 | Up |
| GPR132 | 471.7523247 | 69.54945376 | 873.9551957 | 12.58711761 | 3.653876046 | 7.54E-108 | 2.39E-106 | Up |
| GPR137 | 1317.767904 | 654.8261984 | 1980.709609 | 3.024448143 | 1.596671925 | 1.65E-64 | 2.36E-63 | Up |
| GPR137C | 127.9952682 | 78.54412961 | 177.4464069 | 2.262039344 | 1.177624023 | 1.35E-06 | 2.91E-06 | Up |
| GPR146 | 347.9161628 | 493.1183311 | 202.7139944 | 0.410994843 | -1.282807805 | 6.66E-14 | 2.13E-13 | Down |
| GPR155 | 1875.379461 | 3002.391424 | 748.3674983 | 0.249277908 | -2.004173063 | 6.13E-132 | 2.61E-130 | Down |
| GPR156 | 123.1569768 | 7.133885036 | 239.1800686 | 34.00312638 | 5.087595494 | 2.61E-35 | 1.82E-34 | Up |
| GPR157 | 55.28838136 | 90.31737605 | 20.25938668 | 0.224452845 | -2.155515712 | 2.15E-08 | 5.17E-08 | Down |
| GPR160 | 355.0879394 | 562.8852151 | 147.2906638 | 0.261591764 | -1.934610974 | 1.19E-38 | 9.16E-38 | Down |
| GPR161 | 167.1406634 | 235.052352 | 99.22897486 | 0.42211368 | -1.244296509 | 1.40E-08 | 3.42E-08 | Down |
| GPR171 | 12.95781013 | 1.828055491 | 24.08756477 | 13.10481998 | 3.712025631 | 0.000176188 | 0.000327118 | Up |
| GPR174 | 364.5298248 | 541.1143546 | 187.945295 | 0.347361759 | -1.525489159 | 6.70E-25 | 3.34E-24 | Down |
| GPR183 | 12.82939258 | 0 | 25.65878517 | 125.9724744 | 6.976964722 | 2.62E-07 | 5.94E-07 | Up |
| GPR3 | 132.4195414 | 63.52645647 | 201.3126264 | 3.164124593 | 1.66180641 | 1.10E-10 | 3.03E-10 | Up |
| GPR35 | 135.9361975 | 30.05121397 | 241.821181 | 8.022619785 | 3.004073425 | 2.73E-23 | 1.29E-22 | Up |
| GPR62 | 2.820781766 | 0 | 5.641563532 | 27.70952952 | 4.79231031 | 0.013233034 | 0.020054125 | Up |
| GPR63 | 941.4501052 | 1501.138853 | 381.7613573 | 0.254320961 | -1.97527772 | 9.15E-84 | 2.00E-82 | Down |
| GPR65 | 204.7304045 | 3.373136494 | 406.0876725 | 122.2054784 | 6.933165151 | 5.77E-42 | 4.87E-41 | Up |
| GPR68 | 19.57450088 | 0.356399685 | 38.79260207 | 97.78053004 | 6.611475321 | 3.42E-07 | 7.70E-07 | Up |
| GPR83 | 25.13109427 | 14.83014092 | 35.43204763 | 2.392648834 | 1.25860867 | 0.032066195 | 0.046135276 | Up |
| GPRC5A | 22.65168704 | 9.257704549 | 36.04566954 | 3.900627425 | 1.963706203 | 0.000396705 | 0.000712322 | Up |
| GPRC5B | 128.1178591 | 225.2732407 | 30.9624775 | 0.137481462 | -2.862690999 | 5.23E-28 | 2.92E-27 | Down |
| GPRC5C | 45.41782523 | 17.61353843 | 73.22211203 | 4.180222867 | 2.063579861 | 1.14E-05 | 2.32E-05 | Up |
| GPRIN2 | 6.624160496 | 11.77271423 | 1.47560676 | 0.125875406 | -2.989931668 | 0.013748207 | 0.02077357 | Down |
| GPSM1 | 1674.859552 | 2688.117483 | 661.6016215 | 0.246115718 | -2.022591295 | 1.10E-137 | 5.05E-136 | Down |
| GPT2 | 3786.839734 | 6455.590988 | 1118.08848 | 0.17319875 | -2.529499581 | 0 | 0 | Down |
| GPX3 | 150.3733282 | 221.162774 | 79.58388227 | 0.359895911 | -1.474348383 | 1.04E-11 | 3.01E-11 | Down |
| GRAPL | 102.9728098 | 161.0652053 | 44.88041433 | 0.278753307 | -1.842939176 | 7.64E-09 | 1.89E-08 | Down |
| GRB10 | 3450.686653 | 5243.442106 | 1657.931201 | 0.31617942 | -1.66118463 | 7.05E-167 | 4.46E-165 | Down |
| GRB7 | 8.713162432 | 0.751434084 | 16.67489078 | 22.50639192 | 4.492262887 | 0.000591998 | 0.001042877 | Up |
| GREM2 | 55.66685016 | 6.639580261 | 104.6941201 | 15.76781437 | 3.978910792 | 5.30E-17 | 1.94E-16 | Up |
| GRID2IP | 1271.103251 | 2112.774747 | 429.4317561 | 0.203235806 | -2.298773496 | 1.22E-142 | 5.92E-141 | Down |
| GRIN2C | 39.39079288 | 15.537307 | 63.24427877 | 4.075036801 | 2.026813088 | 2.90E-06 | 6.10E-06 | Up |
| GRK2 | 25343.39571 | 40906.19768 | 9780.593737 | 0.239097683 | -2.064327945 | 0 | 0 | Down |
| GRK3 | 1411.486771 | 1899.568594 | 923.4049486 | 0.486181114 | -1.040434242 | 3.37E-35 | 2.34E-34 | Down |
| GRK5 | 271.0624732 | 114.1838815 | 427.9410649 | 3.750587967 | 1.90711678 | 3.66E-29 | 2.12E-28 | Up |
| GSAP | 7.141980253 | 0 | 14.28396051 | 70.14532172 | 6.132274983 | 4.40E-05 | 8.56E-05 | Up |
| GSDMD | 1480.176238 | 1978.480889 | 981.871587 | 0.496263731 | -1.010821075 | 6.97E-37 | 5.10E-36 | Down |
| GSDME | 621.4227498 | 833.5768443 | 409.2686552 | 0.490927655 | -1.026417656 | 7.53E-21 | 3.21E-20 | Down |
| GSKIP | 491.02797 | 323.7722401 | 658.2836999 | 2.032356944 | 1.023153805 | 1.92E-14 | 6.28E-14 | Up |
| GSTA4 | 4.109215997 | 7.918062498 | 0.300369495 | 0.040354326 | -4.631132857 | 0.008682119 | 0.013479927 | Down |
| GSTK1 | 188.2797089 | 123.869896 | 252.6895217 | 2.039648719 | 1.028320704 | 1.07E-07 | 2.48E-07 | Up |
| GSTM1 | 710.9867927 | 987.2615945 | 434.711991 | 0.440386357 | -1.18315832 | 2.73E-26 | 1.45E-25 | Down |
| GSTM2 | 194.7283182 | 322.0836127 | 67.37302366 | 0.209134378 | -2.257497858 | 3.65E-30 | 2.19E-29 | Down |
| GTF2H1 | 3076.31822 | 1796.483663 | 4356.152778 | 2.424961994 | 1.277962136 | 8.28E-93 | 2.07E-91 | Up |
| GTF2IRD1 | 988.93892 | 1350.064548 | 627.8132921 | 0.465046853 | -1.104552022 | 4.45E-35 | 3.07E-34 | Down |
| GTF2IRD2 | 319.5810864 | 144.0882451 | 495.0739278 | 3.434585566 | 1.780136027 | 3.12E-30 | 1.87E-29 | Up |
| GTF2IRD2B | 480.3938359 | 300.9923002 | 659.7953715 | 2.190609739 | 1.131332488 | 1.59E-16 | 5.69E-16 | Up |
| GTSF1L | 49.12987557 | 85.1417712 | 13.11797995 | 0.15404206 | -2.698603774 | 1.66E-09 | 4.27E-09 | Down |
| GVQW3 | 674.3106167 | 1031.414972 | 317.2062611 | 0.307456562 | -1.701545497 | 2.16E-44 | 1.95E-43 | Down |
| GYPC | 758.0150635 | 1019.794203 | 496.2359242 | 0.48670057 | -1.038893632 | 7.01E-18 | 2.67E-17 | Down |
| GZMA | 1685.425723 | 64.40837329 | 3306.443073 | 51.24257842 | 5.679271166 | 0 | 0 | Up |
| GZMB | 16.12553855 | 0 | 32.25107711 | 158.3252588 | 7.306747628 | 3.99E-08 | 9.46E-08 | Up |
| GZMK | 733.1509539 | 125.7363201 | 1340.565588 | 10.65888945 | 3.413985226 | 9.99E-163 | 6.02E-161 | Up |
| H1-0 | 1196.263031 | 261.3502479 | 2131.175814 | 8.142257841 | 3.025428908 | 3.66E-177 | 2.56E-175 | Up |
| H1-2 | 385.974059 | 218.3647899 | 553.5833282 | 2.536050296 | 1.342583358 | 9.44E-18 | 3.58E-17 | Up |
| H1-3 | 57.00050425 | 87.16784329 | 26.8331652 | 0.307652829 | -1.700624835 | 1.11E-06 | 2.41E-06 | Down |
| H1-5 | 64.31807705 | 114.6290914 | 14.00706274 | 0.122188482 | -3.032819801 | 3.07E-15 | 1.04E-14 | Down |
| H2AC12 | 26.52729402 | 47.70490201 | 5.349686041 | 0.112424969 | -3.152965603 | 4.67E-07 | 1.04E-06 | Down |
| H2AC4 | 6.881595679 | 12.57020538 | 1.192985976 | 0.094902866 | -3.397404534 | 0.002576277 | 0.004266929 | Down |
| H2AC6 | 332.7128775 | 110.1831616 | 555.2425935 | 5.050155815 | 2.336327901 | 9.23E-41 | 7.50E-40 | Up |
| H2AX | 3967.276827 | 5543.409461 | 2391.144193 | 0.431358173 | -1.213041803 | 1.62E-55 | 1.92E-54 | Down |
| H2BC10 | 7.112068844 | 13.03277364 | 1.191364046 | 0.091254625 | -3.453958504 | 0.004784264 | 0.007681434 | Down |
| H2BC12 | 612.6739902 | 403.3134005 | 822.03458 | 2.037859727 | 1.027054749 | 3.97E-21 | 1.71E-20 | Up |
| H2BC13 | 27.45398312 | 39.37081103 | 15.53715522 | 0.394289355 | -1.342673334 | 0.017081777 | 0.02552697 | Down |
| H2BC15 | 88.48262339 | 23.55106175 | 153.414185 | 6.505379379 | 2.701633194 | 6.63E-17 | 2.42E-16 | Up |
| H2BC17 | 41.2334299 | 22.19888821 | 60.26797158 | 2.717520256 | 1.442290789 | 0.000476402 | 0.000847527 | Up |
| H2BC4 | 187.9704454 | 106.6273359 | 269.313555 | 2.52332489 | 1.335325972 | 1.19E-09 | 3.08E-09 | Up |
| H2BC5 | 279.4032656 | 127.9501986 | 430.8563326 | 3.366702089 | 1.751336066 | 2.87E-26 | 1.52E-25 | Up |
| H3-3A | 6792.952464 | 9066.67197 | 4519.232958 | 0.498452422 | -1.004472291 | 1.31E-61 | 1.76E-60 | Down |
| H3-3B | 28677.59608 | 15011.30392 | 42343.88824 | 2.820827107 | 1.496118243 | 7.26E-180 | 5.16E-178 | Up |
| H3-5 | 170.612372 | 86.34066274 | 254.8840813 | 2.948849616 | 1.56015225 | 1.00E-11 | 2.91E-11 | Up |
| H3C11 | 15.66825823 | 25.35174105 | 5.984775404 | 0.236074044 | -2.082688664 | 0.007992273 | 0.01248011 | Down |
| H3C3 | 19.04966148 | 30.33418039 | 7.765142573 | 0.255910355 | -1.96628957 | 0.001116966 | 0.001918692 | Down |
| H3C6 | 125.9550573 | 9.477988205 | 242.4321265 | 25.34412369 | 4.663579376 | 2.83E-38 | 2.15E-37 | Up |
| H3C8 | 43.28073235 | 65.69532618 | 20.86613851 | 0.317846206 | -1.653599228 | 0.000347201 | 0.000626434 | Down |
| H3Y1 | 4.621139368 | 0 | 9.242278736 | 45.37872295 | 5.503944104 | 0.001043138 | 0.001797437 | Up |
| H4-16 | 2.992578877 | 0 | 5.985157755 | 29.37614433 | 4.876573147 | 0.009131576 | 0.014128732 | Up |
| H4C15 | 6.338559107 | 0.385822986 | 12.29129523 | 30.9711506 | 4.952853076 | 0.002285969 | 0.003811598 | Up |
| H4C8 | 45.48399173 | 4.016088789 | 86.95189467 | 21.48320228 | 4.425137152 | 3.25E-15 | 1.10E-14 | Up |
| H4C9 | 41.4436656 | 68.32313654 | 14.56419466 | 0.21326632 | -2.229271945 | 1.15E-06 | 2.49E-06 | Down |
| H6PD | 2481.473086 | 3347.734977 | 1615.211195 | 0.48244392 | -1.051566844 | 5.38E-55 | 6.35E-54 | Down |
| HABP2 | 12.38351296 | 0 | 24.76702592 | 121.593242 | 6.925919237 | 4.26E-07 | 9.52E-07 | Up |
| HAGHL | 365.5294453 | 601.0330704 | 130.0258201 | 0.21628046 | -2.209024765 | 1.49E-51 | 1.61E-50 | Down |
| HAPLN2 | 22.74086706 | 43.98637437 | 1.495359753 | 0.033916577 | -4.881865622 | 3.21E-09 | 8.14E-09 | Down |
| HAPLN3 | 181.2485339 | 290.6753741 | 71.82169374 | 0.247199397 | -2.016252868 | 6.84E-24 | 3.30E-23 | Down |
| HAPLN4 | 4.153693643 | 0 | 8.307387285 | 40.80525648 | 5.350683105 | 0.002249209 | 0.00375301 | Up |
| HAS3 | 124.8657045 | 52.96289349 | 196.7685155 | 3.721107192 | 1.89573195 | 4.02E-14 | 1.30E-13 | Up |
| HASPIN | 2343.251009 | 3310.198597 | 1376.303422 | 0.41580591 | -1.26601783 | 4.32E-83 | 9.36E-82 | Down |
| HAUS4 | 3095.838258 | 4963.545562 | 1228.130954 | 0.247437868 | -2.014861786 | 5.51E-195 | 4.52E-193 | Down |
| HBE1 | 3.129695283 | 6.259390566 | 0 | 0.026271394 | -5.250363456 | 0.003963311 | 0.006428331 | Down |
| HBP1 | 2684.676011 | 1680.495516 | 3688.856506 | 2.195160437 | 1.134326385 | 1.80E-67 | 2.75E-66 | Up |
| HCCS | 1817.570549 | 1183.336839 | 2451.804259 | 2.072440438 | 1.051330639 | 9.88E-41 | 8.02E-40 | Up |
| HCFC1R1 | 515.4195917 | 774.281885 | 256.5572983 | 0.331464946 | -1.593071787 | 1.86E-36 | 1.34E-35 | Down |
| HCK | 64.71899675 | 92.24008356 | 37.19790995 | 0.402998591 | -1.3111533 | 0.000322434 | 0.000583875 | Down |
| HCN2 | 17.44258399 | 9.25949361 | 25.62567438 | 2.772065483 | 1.470961338 | 0.016226805 | 0.024333191 | Up |
| HCN3 | 383.9763476 | 524.6692776 | 243.2834177 | 0.463623959 | -1.108972972 | 4.98E-16 | 1.74E-15 | Down |
| HCRTR1 | 29.05839147 | 6.836331952 | 51.28045098 | 7.602464595 | 2.926467193 | 3.23E-06 | 6.77E-06 | Up |
| HCST | 63.10813408 | 31.63671069 | 94.57955748 | 2.997696359 | 1.583854258 | 2.20E-05 | 4.37E-05 | Up |
| HDAC11 | 75.7563648 | 42.97038862 | 108.542341 | 2.529605456 | 1.338912385 | 8.41E-06 | 1.72E-05 | Up |
| HDAC7 | 3575.293432 | 4848.353882 | 2302.232983 | 0.474843592 | -1.07447571 | 2.06E-60 | 2.70E-59 | Down |
| HDC | 560.6434537 | 10.88516412 | 1110.401743 | 101.2950833 | 6.662420339 | 4.77E-39 | 3.70E-38 | Up |
| HDGF | 44774.62225 | 64470.85998 | 25078.38452 | 0.388990595 | -1.36219282 | 1.65E-163 | 9.97E-162 | Down |
| HEATR4 | 17.54462989 | 1.128045656 | 33.96121413 | 30.5847262 | 4.934739456 | 1.05E-06 | 2.28E-06 | Up |
| HEBP1 | 341.710517 | 495.233165 | 188.1878689 | 0.380042396 | -1.395767725 | 8.12E-19 | 3.23E-18 | Down |
| HECW2 | 13.31537771 | 3.668900517 | 22.9618549 | 6.240411015 | 2.641641053 | 0.000895403 | 0.001552322 | Up |
| HEG1 | 1193.810049 | 1630.218388 | 757.4017113 | 0.464658254 | -1.105758059 | 8.21E-37 | 5.97E-36 | Down |
| HELLS | 2909.648185 | 4507.517659 | 1311.778711 | 0.291017144 | -1.780823948 | 1.04E-99 | 2.84E-98 | Down |
| HELQ | 1490.305697 | 767.8687044 | 2212.742689 | 2.881880331 | 1.527010429 | 1.25E-57 | 1.56E-56 | Up |
| HELZ2 | 7.183689232 | 0.356399685 | 14.01097878 | 35.31977035 | 5.142404057 | 0.000723732 | 0.001264726 | Up |
| HEPACAM2 | 2.522845167 | 0 | 5.045690334 | 24.7843322 | 4.631356482 | 0.01805114 | 0.026894454 | Up |
| HERC6 | 150.4953316 | 91.41036512 | 209.5802981 | 2.294883774 | 1.198421089 | 1.40E-07 | 3.21E-07 | Up |
| HES1 | 3.71864639 | 0 | 7.43729278 | 36.52381862 | 5.190765704 | 0.003020846 | 0.00496177 | Up |
| HES6 | 328.5491068 | 498.0483071 | 159.0499065 | 0.319504125 | -1.646093538 | 8.42E-24 | 4.04E-23 | Down |
| HES7 | 206.8436085 | 304.0955017 | 109.5917153 | 0.36050735 | -1.471899421 | 2.99E-15 | 1.01E-14 | Down |
| HESX1 | 50.59863387 | 85.36050772 | 15.83676001 | 0.185568835 | -2.429973654 | 2.92E-07 | 6.59E-07 | Down |
| HEXD | 627.8033565 | 863.2578703 | 392.3488427 | 0.454458859 | -1.137778398 | 6.05E-19 | 2.42E-18 | Down |
| HEXIM2 | 785.1259108 | 235.9829052 | 1334.268916 | 5.656638548 | 2.499944987 | 4.56E-103 | 1.33E-101 | Up |
| HGF | 30.69399294 | 1.473444867 | 59.91454101 | 40.61348001 | 5.343886746 | 3.30E-10 | 8.84E-10 | Up |
| HGFAC | 13.54051719 | 1.087621881 | 25.99341251 | 23.57713968 | 4.559316799 | 2.24E-05 | 4.45E-05 | Up |
| HHIPL1 | 79.64723203 | 139.019333 | 20.27513111 | 0.1457671 | -2.778262962 | 2.45E-16 | 8.68E-16 | Down |
| HHLA2 | 20.35865753 | 29.71833819 | 10.99897688 | 0.370081164 | -1.434086384 | 0.017769758 | 0.026497991 | Down |
| HIC2 | 887.5424876 | 1204.900164 | 570.184811 | 0.473212559 | -1.079439731 | 6.01E-30 | 3.57E-29 | Down |
| HIGD2A | 1696.326455 | 2269.446111 | 1123.206799 | 0.494944582 | -1.014661097 | 1.14E-32 | 7.35E-32 | Down |
| HIP1 | 2118.8621 | 3235.831094 | 1001.893105 | 0.309601625 | -1.69151505 | 5.70E-93 | 1.43E-91 | Down |
| HIP1R | 3282.684324 | 4506.023811 | 2059.344837 | 0.457032401 | -1.129631648 | 7.58E-75 | 1.37E-73 | Down |
| HIVEP2 | 11803.22009 | 7011.13157 | 16595.30861 | 2.367056398 | 1.24309408 | 1.41E-95 | 3.64E-94 | Up |
| HLA-DOB | 55.77033773 | 89.77085677 | 21.76981869 | 0.242367119 | -2.044734106 | 3.51E-08 | 8.36E-08 | Down |
| HLA-DPA1 | 9557.367744 | 15987.72513 | 3127.010355 | 0.195588526 | -2.354106358 | 0 | 0 | Down |
| HLA-DPB1 | 5475.9314 | 9298.194871 | 1653.667928 | 0.177851548 | -2.491254565 | 0 | 0 | Down |
| HLA-DQA1 | 95.88629356 | 140.5447101 | 51.22787702 | 0.36485363 | -1.454610287 | 3.86E-07 | 8.65E-07 | Down |
| HLA-DQA2 | 167.5855649 | 266.9283014 | 68.24282834 | 0.255660121 | -1.967700953 | 3.47E-19 | 1.40E-18 | Down |
| HLA-DQB1 | 184.3683141 | 262.1377293 | 106.598899 | 0.406575112 | -1.298406191 | 4.30E-09 | 1.08E-08 | Down |
| HLA-DQB2 | 72.94124488 | 102.0803547 | 43.80213505 | 0.429071883 | -1.220708729 | 0.000718545 | 0.001256389 | Down |
| HLA-DRA | 30592.02029 | 48657.99188 | 12526.04871 | 0.257431888 | -1.957737325 | 0 | 0 | Down |
| HLA-DRB1 | 1750.626679 | 2585.104549 | 916.1488099 | 0.354387548 | -1.496600183 | 2.93E-51 | 3.15E-50 | Down |
| HLA-DRB5 | 993.6826685 | 1450.921458 | 536.4438794 | 0.36971278 | -1.435523181 | 0.000414493 | 0.000742725 | Down |
| HLA-F | 2360.486721 | 3265.728636 | 1455.244806 | 0.445617853 | -1.166121061 | 1.03E-64 | 1.48E-63 | Down |
| HLF | 3.884146953 | 0 | 7.768293907 | 38.13472128 | 5.25303325 | 0.003343133 | 0.005461382 | Up |
| HLTF | 2635.961374 | 3666.67377 | 1605.248978 | 0.437805421 | -1.191638277 | 6.37E-39 | 4.94E-38 | Down |
| HMBOX1 | 3368.676229 | 4769.189925 | 1968.162533 | 0.412686807 | -1.276880778 | 1.36E-60 | 1.80E-59 | Down |
| HMCN1 | 2.08984846 | 0 | 4.17969692 | 20.51890993 | 4.358882184 | 0.032643688 | 0.046893186 | Up |
| HMG20B | 3615.154836 | 4981.316064 | 2248.993608 | 0.451528765 | -1.147110196 | 3.16E-58 | 3.97E-57 | Down |
| HMGA1 | 16772.45535 | 23896.94257 | 9647.968124 | 0.403735355 | -1.308518166 | 2.42E-115 | 8.43E-114 | Down |
| HMGB1 | 26366.55469 | 36511.18898 | 16221.9204 | 0.444299552 | -1.170395407 | 2.50E-142 | 1.21E-140 | Down |
| HMGB2 | 25338.89884 | 34932.9842 | 15744.81348 | 0.450711877 | -1.149722628 | 1.06E-133 | 4.66E-132 | Down |
| HMGB3 | 2968.5091 | 4021.6544 | 1915.3638 | 0.476296509 | -1.070068119 | 6.06E-44 | 5.40E-43 | Down |
| HMGCL | 1402.498472 | 880.5103826 | 1924.486562 | 2.185164011 | 1.127741568 | 2.99E-44 | 2.68E-43 | Up |
| HMGN3 | 2253.659284 | 3154.995086 | 1352.323482 | 0.428670195 | -1.222059985 | 6.67E-64 | 9.36E-63 | Down |
| HMGN5 | 737.3908212 | 1232.164235 | 242.6174069 | 0.196932288 | -2.34422843 | 2.37E-90 | 5.68E-89 | Down |
| HOMER3 | 211.3302027 | 297.174478 | 125.4859273 | 0.422230286 | -1.243898028 | 8.81E-12 | 2.56E-11 | Down |
| HOOK1 | 3.580006334 | 0 | 7.160012669 | 35.15293016 | 5.135573044 | 0.005074964 | 0.008130611 | Up |
| HORMAD1 | 9.298838158 | 0.385822986 | 18.21185333 | 45.90289408 | 5.52051321 | 0.000123122 | 0.000232105 | Up |
| HOXA7 | 3.868211348 | 0 | 7.736422696 | 37.99122585 | 5.247594359 | 0.003639932 | 0.005925998 | Up |
| HPCA | 11.6581762 | 20.32477566 | 2.991576736 | 0.147005474 | -2.766058217 | 0.002827247 | 0.004658677 | Down |
| HPD | 6.435053479 | 0.356399685 | 12.51370727 | 31.54708062 | 4.979434599 | 0.000880823 | 0.001528192 | Up |
| HPDL | 89.0108454 | 134.5040883 | 43.51760251 | 0.323612101 | -1.62766254 | 1.26E-07 | 2.90E-07 | Down |
| HPN | 2.534343594 | 0 | 5.068687189 | 24.88645848 | 4.637289034 | 0.015355062 | 0.02308335 | Up |
| HPS4 | 28921.01124 | 45988.78185 | 11853.24063 | 0.257743659 | -1.95599116 | 0 | 0 | Down |
| HPSE2 | 44.28188425 | 62.91318552 | 25.65058298 | 0.407838915 | -1.293928656 | 0.002818808 | 0.004645323 | Down |
| HRH1 | 268.9393623 | 14.62416976 | 523.2545549 | 35.87668515 | 5.164974693 | 2.99E-11 | 8.43E-11 | Up |
| HSBP1L1 | 160.1363556 | 45.84743128 | 274.4252798 | 5.992947675 | 2.583265778 | 1.59E-29 | 9.31E-29 | Up |
| HSD11B1L | 83.77867545 | 122.8607924 | 44.69655852 | 0.363998949 | -1.457993808 | 2.75E-06 | 5.80E-06 | Down |
| HSD17B7 | 765.7376279 | 443.0150321 | 1088.460224 | 2.456514611 | 1.29661282 | 8.40E-26 | 4.35E-25 | Up |
| HSD17B8 | 571.686355 | 832.1694357 | 311.2032743 | 0.374053837 | -1.418682167 | 2.24E-33 | 1.47E-32 | Down |
| HSF4 | 352.1867125 | 536.8467537 | 167.5266713 | 0.31209385 | -1.679948165 | 1.04E-30 | 6.36E-30 | Down |
| HSPA1B | 808.5650404 | 469.6941337 | 1147.435947 | 2.444596922 | 1.289596605 | 1.52E-33 | 9.96E-33 | Up |
| HSPA6 | 97.28221064 | 14.131396 | 180.4330253 | 12.64555488 | 3.660558438 | 3.47E-21 | 1.50E-20 | Up |
| HSPG2 | 129.7315731 | 180.6195918 | 78.84355448 | 0.436233349 | -1.196828029 | 2.88E-05 | 5.69E-05 | Down |
| HSPH1 | 2359.457797 | 1163.096238 | 3555.819355 | 3.056039943 | 1.6116634 | 6.42E-83 | 1.38E-81 | Up |
| HTR3A | 24.86768939 | 0 | 49.73537878 | 244.2222082 | 7.932050586 | 4.71E-10 | 1.25E-09 | Up |
| HTRA4 | 4.836894715 | 0.71279937 | 8.960990061 | 12.29418396 | 3.619904072 | 0.017378201 | 0.025941995 | Up |
| HVCN1 | 3474.444705 | 1337.189183 | 5611.700228 | 4.196896966 | 2.069323046 | 6.58E-212 | 6.43E-210 | Up |
| HYAL3 | 126.8042606 | 31.15314834 | 222.4553728 | 7.119180895 | 2.83171126 | 1.92E-22 | 8.68E-22 | Up |
| HYKK | 258.655977 | 382.2244092 | 135.0875447 | 0.353292325 | -1.501065686 | 4.41E-17 | 1.62E-16 | Down |
| IBA57 | 904.0251733 | 575.0345489 | 1233.015798 | 2.143334049 | 1.099856719 | 6.19E-23 | 2.87E-22 | Up |
| ICAM1 | 4390.427909 | 1950.953807 | 6829.90201 | 3.501739636 | 1.80807182 | 3.83E-122 | 1.46E-120 | Up |
| ICAM2 | 2074.062034 | 1234.687176 | 2913.436892 | 2.360332242 | 1.238989949 | 7.27E-42 | 6.11E-41 | Up |
| ICAM4 | 166.9548571 | 234.9487628 | 98.96095146 | 0.421310375 | -1.247044651 | 4.12E-10 | 1.10E-09 | Down |
| ICAM5 | 169.1390963 | 237.7048002 | 100.5733923 | 0.423007322 | -1.241245459 | 6.21E-09 | 1.55E-08 | Down |
| ICOSLG | 72.44289085 | 37.63308061 | 107.2527011 | 2.849600828 | 1.510759841 | 7.09E-05 | 0.00013631 | Up |
| ID1 | 38.18585794 | 71.61389446 | 4.757821406 | 0.06654222 | -3.9095862 | 6.20E-13 | 1.89E-12 | Down |
| ID2 | 827.0884019 | 549.7982051 | 1104.378599 | 2.009521998 | 1.006852371 | 6.13E-23 | 2.84E-22 | Up |
| ID3 | 1773.869806 | 2873.386904 | 674.3527083 | 0.234683928 | -2.091209049 | 4.08E-174 | 2.76E-172 | Down |
| IDH1 | 1206.551928 | 1651.945896 | 761.1579591 | 0.460660393 | -1.118224531 | 7.20E-39 | 5.58E-38 | Down |
| IDH2 | 17634.88767 | 24621.96288 | 10647.81247 | 0.43245619 | -1.209374106 | 1.75E-118 | 6.35E-117 | Down |
| IER2 | 7938.574146 | 3359.554498 | 12517.59379 | 3.726076902 | 1.89765745 | 9.59E-218 | 9.93E-216 | Up |
| IER3 | 324.4524466 | 433.7693763 | 215.135517 | 0.496075459 | -1.011368505 | 3.98E-12 | 1.18E-11 | Down |
| IER5 | 1894.999853 | 1000.295776 | 2789.70393 | 2.788863573 | 1.479677361 | 1.09E-67 | 1.68E-66 | Up |
| IER5L | 43.77970953 | 58.91678447 | 28.6426346 | 0.485915332 | -1.04122314 | 0.012422113 | 0.018907838 | Down |
| IFFO2 | 38.49679558 | 55.54160087 | 21.4519903 | 0.385740353 | -1.374298019 | 0.002381946 | 0.003963529 | Down |
| IFI27L1 | 623.1330369 | 931.5625759 | 314.7034978 | 0.337827161 | -1.565642769 | 8.62E-45 | 7.87E-44 | Down |
| IFI44L | 29.19680759 | 52.11218903 | 6.281426158 | 0.120371821 | -3.054430402 | 2.56E-07 | 5.79E-07 | Down |
| IFI6 | 354.9483352 | 550.5386549 | 159.3580156 | 0.289524639 | -1.788241966 | 2.91E-30 | 1.75E-29 | Down |
| IFIH1 | 881.6479643 | 509.8245661 | 1253.471362 | 2.457880926 | 1.297415025 | 2.76E-30 | 1.66E-29 | Up |
| IFIT1 | 329.2329587 | 512.7098294 | 145.7560879 | 0.284220214 | -1.814918931 | 2.06E-32 | 1.32E-31 | Down |
| IFITM10 | 29.8426071 | 13.67982821 | 46.005386 | 3.364979873 | 1.750597876 | 0.000590264 | 0.001039953 | Up |
| IFITM2 | 7246.336551 | 9834.144022 | 4658.529081 | 0.473729694 | -1.077863989 | 9.46E-73 | 1.63E-71 | Down |
| IFNAR2 | 1427.866923 | 2099.382077 | 756.3517696 | 0.360197873 | -1.473138435 | 8.12E-66 | 1.20E-64 | Down |
| IFNLR1 | 332.3789645 | 84.93732299 | 579.8206059 | 6.829932061 | 2.771871228 | 9.62E-52 | 1.05E-50 | Up |
| IFRD1 | 2664.731709 | 1525.644635 | 3803.818783 | 2.493465581 | 1.318152288 | 4.32E-73 | 7.53E-72 | Up |
| IFT140 | 1614.129271 | 2313.828784 | 914.4297578 | 0.395156821 | -1.339502783 | 2.57E-66 | 3.84E-65 | Down |
| IFT57 | 502.4550313 | 320.3003413 | 684.6097212 | 2.138363176 | 1.096506899 | 2.62E-19 | 1.06E-18 | Up |
| IFT74 | 78.82585003 | 42.09410509 | 115.557595 | 2.746417192 | 1.457550794 | 1.94E-06 | 4.13E-06 | Up |
| IGF1 | 34.63161213 | 9.140011346 | 60.1232129 | 6.549015569 | 2.711278061 | 8.04E-08 | 1.88E-07 | Up |
| IGFALS | 17.95059918 | 9.97229298 | 25.92890538 | 2.601454449 | 1.379318446 | 0.022497894 | 0.033072095 | Up |
| IGFBP6 | 7.309014013 | 13.12667683 | 1.49135119 | 0.113392981 | -3.140596757 | 0.004708057 | 0.007566384 | Down |
| IGIP | 198.1492383 | 293.059643 | 103.2388337 | 0.352208289 | -1.505499231 | 1.40E-13 | 4.41E-13 | Down |
| IGLL1 | 27878.02985 | 40633.18265 | 15122.87706 | 0.372185879 | -1.425904774 | 6.63E-134 | 2.93E-132 | Down |
| IGLON5 | 7.107149124 | 13.61355926 | 0.600738991 | 0.043869129 | -4.51065013 | 0.001008116 | 0.0017412 | Down |
| IGSF3 | 1353.99047 | 2108.958563 | 599.0223773 | 0.284032096 | -1.815874127 | 5.63E-104 | 1.68E-102 | Down |
| IGSF6 | 45.40096441 | 23.14323782 | 67.65869101 | 2.919242161 | 1.545593892 | 0.00017876 | 0.000331583 | Up |
| IKZF2 | 13107.15837 | 20232.76096 | 5981.555773 | 0.295641497 | -1.758079311 | 1.81E-153 | 9.75E-152 | Down |
| IKZF3 | 161.3441903 | 57.48019332 | 265.2081873 | 4.599815617 | 2.201576032 | 4.29E-21 | 1.85E-20 | Up |
| IKZF4 | 369.6082578 | 537.0913515 | 202.1251641 | 0.376388644 | -1.409704994 | 6.24E-21 | 2.67E-20 | Down |
| IL10 | 3.162455243 | 0.365611098 | 5.959299388 | 15.02332729 | 3.909132464 | 0.025560143 | 0.037344086 | Up |
| IL11 | 49.14730436 | 12.14753674 | 86.14707198 | 7.083827752 | 2.824529132 | 3.00E-11 | 8.44E-11 | Up |
| IL11RA | 291.186965 | 431.8185104 | 150.5554196 | 0.348758192 | -1.519700991 | 3.34E-22 | 1.50E-21 | Down |
| IL17C | 6.140934268 | 0.356399685 | 11.92546885 | 30.06201766 | 4.909869936 | 0.001121874 | 0.001926406 | Up |
| IL17F | 2.242657279 | 0 | 4.485314559 | 22.01596179 | 4.460477966 | 0.026953197 | 0.039238854 | Up |
| IL18R1 | 16.6552827 | 0 | 33.3105654 | 163.588597 | 7.353928378 | 3.20E-08 | 7.64E-08 | Up |
| IL18RAP | 327.6151156 | 1.453232979 | 653.7769982 | 443.2146548 | 8.791861774 | 9.00E-33 | 5.79E-32 | Up |
| IL19 | 4.21620402 | 0.356399685 | 8.076008355 | 20.35125875 | 4.347046125 | 0.009051512 | 0.014015798 | Up |
| IL1B | 626.90639 | 1129.360297 | 124.4524825 | 0.11014267 | -3.182554605 | 1.37E-116 | 4.84E-115 | Down |
| IL1R1 | 128.6893738 | 6.636002138 | 250.7427454 | 37.76571274 | 5.23900511 | 7.01E-38 | 5.28E-37 | Up |
| IL1R2 | 3.873888106 | 0 | 7.747776212 | 38.04259701 | 5.249543831 | 0.001935606 | 0.003252822 | Up |
| IL21R | 1079.841673 | 405.9156651 | 1753.767682 | 4.323492566 | 2.112197209 | 3.53E-92 | 8.75E-91 | Up |
| IL23A | 236.2601692 | 76.64265698 | 395.8776813 | 5.162470202 | 2.368061549 | 1.02E-36 | 7.40E-36 | Up |
| IL24 | 77.34614459 | 0.356399685 | 154.3358895 | 389.0798537 | 8.60392247 | 1.11E-12 | 3.35E-12 | Up |
| IL27RA | 97.40029469 | 146.8404222 | 47.96016722 | 0.32681083 | -1.613472302 | 5.15E-09 | 1.29E-08 | Down |
| IL2RG | 9808.121165 | 3312.858983 | 16303.38335 | 4.921792528 | 2.299183844 | 0 | 0 | Up |
| IL31RA | 6.130200542 | 0.356399685 | 11.9040014 | 30.01295415 | 4.907513424 | 0.001862721 | 0.003134515 | Up |
| IL32 | 212.8520704 | 12.71168859 | 412.9924521 | 32.84158958 | 5.037452052 | 1.98E-61 | 2.65E-60 | Up |
| IL36B | 2.991148122 | 0 | 5.982296243 | 29.36337753 | 4.875946019 | 0.008987528 | 0.01391983 | Up |
| IL37 | 9.500707133 | 15.74095689 | 3.260457371 | 0.207356106 | -2.269817561 | 0.01461837 | 0.022030792 | Down |
| IL4I1 | 1038.293569 | 96.02898243 | 1980.558155 | 20.64224777 | 4.367528172 | 8.40E-269 | 1.18E-266 | Up |
| IL5RA | 3.016126155 | 0.385822986 | 5.646429324 | 14.23882962 | 3.831758662 | 0.032388592 | 0.046560489 | Up |
| IL7R | 18515.37153 | 27446.08123 | 9584.661828 | 0.349221816 | -1.517784406 | 4.96E-129 | 2.04E-127 | Down |
| ILDR1 | 2.221473531 | 0 | 4.442947061 | 21.82711446 | 4.448049514 | 0.033140251 | 0.047576942 | Up |
| ILDR2 | 259.3326139 | 388.6610723 | 130.0041554 | 0.334336985 | -1.580625139 | 5.70E-21 | 2.44E-20 | Down |
| ILKAP | 2241.44213 | 1450.13628 | 3032.74798 | 2.09214521 | 1.064982988 | 2.36E-42 | 2.01E-41 | Up |
| IMPA2 | 464.0745583 | 791.6477959 | 136.5013208 | 0.172450111 | -2.535749037 | 1.45E-65 | 2.11E-64 | Down |
| IMPG2 | 33.85080215 | 20.35983225 | 47.34177205 | 2.328404218 | 1.219341536 | 0.00715753 | 0.011249494 | Up |
| INAVA | 5.919683932 | 11.24387702 | 0.595490847 | 0.053104087 | -4.235033292 | 0.003327949 | 0.005437859 | Down |
| ING3 | 1690.617194 | 1079.958813 | 2301.275574 | 2.130986294 | 1.091521314 | 7.48E-44 | 6.65E-43 | Up |
| INHBA | 7.905569759 | 1.811421726 | 13.99971779 | 7.65658958 | 2.936701926 | 0.007996207 | 0.012484847 | Up |
| INHBC | 27.18473753 | 36.48508917 | 17.88438589 | 0.490082631 | -1.028903077 | 0.0321611 | 0.04625263 | Down |
| INHBE | 394.6659322 | 648.7185174 | 140.613347 | 0.216746724 | -2.205917909 | 4.14E-40 | 3.32E-39 | Down |
| INPP5J | 5.515536414 | 1.473444867 | 9.55762796 | 6.47953054 | 2.69588929 | 0.025240945 | 0.036897159 | Up |
| INSC | 97.16566488 | 141.9308919 | 52.40043783 | 0.369283958 | -1.437197504 | 1.40E-07 | 3.22E-07 | Down |
| INSIG1 | 2936.861927 | 4618.455935 | 1255.267918 | 0.271791497 | -1.879427772 | 8.25E-164 | 5.06E-162 | Down |
| INSIG2 | 789.8528401 | 374.5033484 | 1205.202332 | 3.219313372 | 1.686753017 | 1.94E-50 | 2.04E-49 | Up |
| INSL3 | 51.13835995 | 21.24175713 | 81.03496277 | 3.804517456 | 1.927713482 | 1.89E-06 | 4.03E-06 | Up |
| INSR | 3618.776028 | 5440.423687 | 1797.128369 | 0.33031783 | -1.598073247 | 9.78E-123 | 3.76E-121 | Down |
| INTS1 | 8526.289195 | 12177.06357 | 4875.514823 | 0.400373587 | -1.320581292 | 5.70E-101 | 1.58E-99 | Down |
| INTS12 | 1634.670399 | 966.545555 | 2302.795244 | 2.383346858 | 1.252988928 | 3.93E-53 | 4.43E-52 | Up |
| IP6K1 | 5456.945121 | 3018.547984 | 7895.342259 | 2.615754768 | 1.387227292 | 5.27E-103 | 1.53E-101 | Up |
| IPCEF1 | 1032.707299 | 449.0584913 | 1616.356107 | 3.60142133 | 1.84856639 | 2.43E-69 | 3.89E-68 | Up |
| IPP | 223.8401624 | 301.3655756 | 146.3147492 | 0.485648262 | -1.042016297 | 1.06E-09 | 2.77E-09 | Down |
| IQCD | 22.02195748 | 7.314011024 | 36.72990394 | 5.005984025 | 2.323653687 | 0.001698053 | 0.002863322 | Up |
| IQCE | 647.4894601 | 406.8807093 | 888.0982108 | 2.182107448 | 1.125722142 | 9.07E-25 | 4.49E-24 | Up |
| IQCN | 196.4527884 | 28.70467372 | 364.2009032 | 12.6720438 | 3.663577322 | 2.16E-39 | 1.68E-38 | Up |
| IQSEC1 | 7223.474675 | 10745.35392 | 3701.595429 | 0.344475427 | -1.537527023 | 7.36E-209 | 6.80E-207 | Down |
| IQSEC3 | 3.116772694 | 6.233545388 | 0 | 0.026368608 | -5.245034749 | 0.004123522 | 0.006674918 | Down |
| IRAG1 | 43.52568151 | 70.36969468 | 16.68166833 | 0.236759827 | -2.078503789 | 4.32E-06 | 9.00E-06 | Down |
| IRAK2 | 34.9937565 | 4.344854235 | 65.64265876 | 14.92521817 | 3.899680115 | 1.71E-10 | 4.65E-10 | Up |
| IRF8 | 730.3635653 | 343.4177339 | 1117.309397 | 3.254661499 | 1.702507504 | 1.95E-54 | 2.28E-53 | Up |
| IRGM | 57.97078612 | 13.12667683 | 102.8148954 | 7.782915077 | 2.960310615 | 1.60E-13 | 5.02E-13 | Up |
| IRX1 | 974.1269995 | 1301.843566 | 646.4104329 | 0.496639649 | -1.00972865 | 7.40E-24 | 3.57E-23 | Down |
| IRX5 | 154.9349401 | 213.600321 | 96.26955916 | 0.450715138 | -1.149712189 | 7.75E-08 | 1.81E-07 | Down |
| ISL2 | 48.06019726 | 78.2038476 | 17.91654692 | 0.228860223 | -2.127461359 | 2.99E-06 | 6.28E-06 | Down |
| ISM1 | 2033.090558 | 2737.340805 | 1328.840311 | 0.48541551 | -1.042707889 | 3.79E-49 | 3.87E-48 | Down |
| ITGA10 | 105.1931812 | 57.77110624 | 152.6152562 | 2.645362865 | 1.403465631 | 5.08E-08 | 1.20E-07 | Up |
| ITGA2B | 84.18657586 | 113.5312017 | 54.84195002 | 0.482734423 | -1.050698388 | 0.000296448 | 0.000539083 | Down |
| ITGA9 | 37.81476215 | 14.29156814 | 61.33795616 | 4.279992805 | 2.097608371 | 3.49E-06 | 7.31E-06 | Up |
| ITGAL | 58.92853366 | 25.39011772 | 92.46694961 | 3.637785547 | 1.863060496 | 1.29E-07 | 2.97E-07 | Up |
| ITGAM | 167.1687376 | 69.57450869 | 264.7629666 | 3.802950924 | 1.927119321 | 9.72E-18 | 3.68E-17 | Up |
| ITGAV | 205.5721426 | 282.9745079 | 128.1697774 | 0.452759199 | -1.143184141 | 2.39E-09 | 6.09E-09 | Down |
| ITGB1 | 3504.510214 | 5000.153129 | 2008.867298 | 0.401791612 | -1.315480648 | 8.21E-84 | 1.80E-82 | Down |
| ITGB1BP2 | 54.13146599 | 82.93352602 | 25.32940597 | 0.305400161 | -1.711227274 | 6.93E-06 | 1.43E-05 | Down |
| ITGB3 | 125.7016459 | 10.84705356 | 240.5562382 | 22.40218933 | 4.485567827 | 2.79E-36 | 1.99E-35 | Up |
| ITGB3BP | 1026.335517 | 1422.519703 | 630.151332 | 0.443120291 | -1.174229704 | 2.08E-33 | 1.36E-32 | Down |
| ITGB7 | 52.92787624 | 30.17811052 | 75.67764196 | 2.516081889 | 1.331178877 | 0.000353748 | 0.000637334 | Up |
| ITIH3 | 152.9432281 | 260.6218872 | 45.26456907 | 0.173647738 | -2.525764476 | 1.69E-20 | 7.10E-20 | Down |
| ITK | 16.61470001 | 2.553644396 | 30.67575562 | 11.93143918 | 3.576696168 | 1.41E-05 | 2.85E-05 | Up |
| ITM2A | 372.6769272 | 534.0480449 | 211.3058095 | 0.395756261 | -1.337315919 | 1.12E-21 | 4.96E-21 | Down |
| ITPA | 1979.213046 | 1089.658658 | 2868.767435 | 2.633088107 | 1.396755797 | 1.62E-56 | 1.97E-55 | Up |
| ITPKC | 423.0225474 | 255.7558359 | 590.289259 | 2.310654948 | 1.208301837 | 2.74E-15 | 9.29E-15 | Up |
| IZUMO1 | 57.17218331 | 96.40711695 | 17.93724967 | 0.185880258 | -2.427554541 | 8.21E-10 | 2.15E-09 | Down |
| JAM2 | 25.0098823 | 7.075038434 | 42.94472617 | 6.096050612 | 2.607874881 | 2.97E-05 | 5.85E-05 | Up |
| JAZF1 | 15.8630561 | 3.747959005 | 27.97815319 | 7.531448341 | 2.91292733 | 0.000114369 | 0.000216191 | Up |
| JCAD | 48.06121535 | 4.662619206 | 91.45981149 | 19.53011494 | 4.287628534 | 0.002232213 | 0.003725996 | Up |
| JCHAIN | 476.693044 | 81.31653471 | 872.0695533 | 10.73660192 | 3.424465554 | 4.45E-107 | 1.40E-105 | Up |
| JDP2 | 1294.727564 | 2182.435796 | 407.0193323 | 0.186505994 | -2.422706094 | 4.12E-120 | 1.53E-118 | Down |
| JMJD6 | 2370.030206 | 1145.794849 | 3594.265564 | 3.136676247 | 1.649236629 | 2.47E-134 | 1.10E-132 | Up |
| JOSD1 | 6087.227478 | 4009.069799 | 8165.385156 | 2.036842533 | 1.02633445 | 3.44E-74 | 6.09E-73 | Up |
| JOSD2 | 120.6656386 | 71.80297382 | 169.5283034 | 2.35620682 | 1.23646618 | 1.06E-06 | 2.30E-06 | Up |
| JRK | 1878.925856 | 2927.360423 | 830.4912897 | 0.283665969 | -1.817735011 | 3.62E-129 | 1.50E-127 | Down |
| JSRP1 | 10.59740877 | 4.479181202 | 16.71563635 | 3.748614358 | 1.906357415 | 0.025854004 | 0.03772971 | Up |
| JUN | 12081.76104 | 888.807886 | 23274.71419 | 26.18839037 | 4.710855484 | 0 | 0 | Up |
| JUNB | 8163.819603 | 3492.374447 | 12835.26476 | 3.675686017 | 1.878013535 | 3.91E-157 | 2.23E-155 | Up |
| JUND | 8038.99596 | 4349.031879 | 11728.96004 | 2.697050657 | 1.431382619 | 8.95E-108 | 2.82E-106 | Up |
| KALRN | 64.80776124 | 93.50935438 | 36.1061681 | 0.385550556 | -1.375008047 | 0.000272583 | 0.000497653 | Down |
| KANK2 | 5806.938705 | 9481.724566 | 2132.152845 | 0.224868324 | -2.152847643 | 2.74E-250 | 3.42E-248 | Down |
| KANK3 | 383.9480116 | 562.7941573 | 205.1018659 | 0.364368661 | -1.456529215 | 3.65E-25 | 1.85E-24 | Down |
| KANSL1L | 774.3132618 | 1058.365008 | 490.2615159 | 0.463134563 | -1.110496666 | 3.39E-24 | 1.65E-23 | Down |
| KANSL2 | 3141.686936 | 2003.188105 | 4280.185767 | 2.136478598 | 1.095234865 | 3.04E-58 | 3.83E-57 | Up |
| KASH5 | 4.352426805 | 0.385822986 | 8.319030624 | 20.97829389 | 4.390825447 | 0.008267189 | 0.012877504 | Up |
| KAT5 | 3052.15918 | 1901.788365 | 4202.529996 | 2.210492627 | 1.144367922 | 1.50E-47 | 1.48E-46 | Up |
| KAZALD1 | 584.7356799 | 809.8932366 | 359.5781231 | 0.443934006 | -1.17158287 | 4.83E-25 | 2.42E-24 | Down |
| KAZN | 941.5781811 | 1386.853082 | 496.3032806 | 0.357893286 | -1.482398617 | 1.99E-56 | 2.41E-55 | Down |
| KBTBD11 | 1479.221785 | 2383.203613 | 575.2399573 | 0.241317961 | -2.050992799 | 7.21E-121 | 2.71E-119 | Down |
| KBTBD6 | 554.6479023 | 783.6225246 | 325.67328 | 0.415645788 | -1.266573504 | 2.29E-27 | 1.25E-26 | Down |
| KBTBD8 | 803.1098146 | 366.7236576 | 1239.495972 | 3.381088042 | 1.757487584 | 3.78E-51 | 4.04E-50 | Up |
| KCNC4 | 189.491302 | 278.1553108 | 100.8272932 | 0.362173065 | -1.46524884 | 1.78E-11 | 5.08E-11 | Down |
| KCNE5 | 33.69397213 | 19.72814653 | 47.65979773 | 2.422354218 | 1.276409844 | 0.009392665 | 0.014508425 | Up |
| KCNH3 | 7.34861627 | 0.356399685 | 14.34083285 | 36.14381441 | 5.175676863 | 0.000593763 | 0.001045852 | Up |
| KCNIP2 | 44.94149122 | 72.6583047 | 17.22467774 | 0.237415383 | -2.074514682 | 2.15E-05 | 4.28E-05 | Down |
| KCNJ10 | 24.66027163 | 39.16179395 | 10.1587493 | 0.25924743 | -1.947598407 | 0.000442303 | 0.000789702 | Down |
| KCNJ11 | 66.48832764 | 114.7531586 | 18.22349667 | 0.158549163 | -2.656997832 | 2.89E-12 | 8.59E-12 | Down |
| KCNJ12 | 69.39917206 | 112.534429 | 26.26391507 | 0.233279241 | -2.099870163 | 2.75E-10 | 7.40E-10 | Down |
| KCNJ14 | 291.2958417 | 124.1635968 | 458.4280866 | 3.69471289 | 1.885462259 | 6.32E-30 | 3.75E-29 | Up |
| KCNJ16 | 11.2209877 | 2.175243763 | 20.26673163 | 9.220170305 | 3.204793399 | 0.000550277 | 0.000972715 | Up |
| KCNJ2 | 1836.438408 | 297.0048859 | 3375.87193 | 11.37297651 | 3.507537977 | 6.06E-260 | 8.01E-258 | Up |
| KCNK3 | 16.14455519 | 1.848267378 | 30.44084301 | 16.48998435 | 4.043518125 | 5.63E-06 | 1.16E-05 | Up |
| KCNK7 | 13.24498342 | 5.913991355 | 20.57597548 | 3.481871993 | 1.799863165 | 0.011788128 | 0.017978353 | Up |
| KCNMB1 | 159.5505793 | 67.28030681 | 251.8208518 | 3.746384708 | 1.905499054 | 2.94E-19 | 1.19E-18 | Up |
| KCNMB3 | 58.10955503 | 86.98107715 | 29.23803292 | 0.336381005 | -1.571831853 | 7.27E-05 | 0.000139541 | Down |
| KCNMB4 | 174.4564666 | 262.8661556 | 86.04677762 | 0.327420627 | -1.610782881 | 4.70E-14 | 1.51E-13 | Down |
| KCNN1 | 16.54286743 | 4.442335549 | 28.6433993 | 6.459413815 | 2.691403248 | 0.000181962 | 0.000337432 | Up |
| KCNN2 | 3.929314587 | 0.365611098 | 7.493018076 | 18.88093025 | 4.238857942 | 0.022096926 | 0.032530934 | Up |
| KCNN3 | 462.7232954 | 864.6391435 | 60.80744731 | 0.070314341 | -3.830037223 | 4.96E-134 | 2.20E-132 | Down |
| KCNS3 | 1361.638658 | 1976.515903 | 746.7614132 | 0.377814542 | -1.404249864 | 1.46E-67 | 2.26E-66 | Down |
| KCTD5 | 2716.382233 | 1562.429074 | 3870.335392 | 2.477183422 | 1.308700696 | 1.84E-77 | 3.51E-76 | Up |
| KCTD7 | 760.701926 | 1188.388102 | 333.0157503 | 0.28018935 | -1.835525977 | 4.89E-68 | 7.61E-67 | Down |
| KDM1B | 3677.023512 | 5236.207154 | 2117.83987 | 0.404458225 | -1.305937394 | 1.23E-74 | 2.20E-73 | Down |
| KDM3A | 5256.160909 | 2773.756289 | 7738.56553 | 2.790035848 | 1.480283659 | 6.65E-162 | 3.96E-160 | Up |
| KDM4B | 1068.071267 | 699.1697788 | 1436.972756 | 2.054836913 | 1.039023896 | 1.27E-25 | 6.52E-25 | Up |
| KDM6A | 2930.261896 | 1843.207418 | 4017.316373 | 2.179293838 | 1.123860731 | 7.76E-56 | 9.34E-55 | Up |
| KDM6B | 7038.971973 | 4578.727251 | 9499.216694 | 2.074709279 | 1.052909191 | 1.93E-64 | 2.75E-63 | Up |
| KDR | 74.6265987 | 5.620016394 | 143.633181 | 25.86990482 | 4.693202841 | 3.15E-23 | 1.48E-22 | Up |
| KEL | 16.17304328 | 27.27138652 | 5.074700033 | 0.18595825 | -2.426949339 | 0.001203911 | 0.002060389 | Down |
| KIAA0040 | 3868.128685 | 5290.357938 | 2445.899431 | 0.462321923 | -1.11303032 | 1.66E-70 | 2.74E-69 | Down |
| KIAA0513 | 213.2663994 | 138.5319103 | 288.0008885 | 2.078248429 | 1.055368121 | 6.49E-08 | 1.52E-07 | Up |
| KIAA0753 | 985.689577 | 562.2141044 | 1409.16505 | 2.505134902 | 1.324888295 | 2.42E-45 | 2.24E-44 | Up |
| KIAA0754 | 702.5335342 | 965.2243104 | 439.8427579 | 0.455720323 | -1.133779384 | 1.95E-21 | 8.50E-21 | Down |
| KIAA0825 | 55.50359032 | 27.68663281 | 83.32054783 | 3.010881529 | 1.590185942 | 1.63E-05 | 3.28E-05 | Up |
| KIAA0930 | 3137.510051 | 4403.279276 | 1871.740826 | 0.425084592 | -1.234178128 | 4.11E-74 | 7.27E-73 | Down |
| KIAA1217 | 63.17179179 | 0.365611098 | 125.9779725 | 317.6017788 | 8.311075182 | 9.06E-12 | 2.63E-11 | Up |
| KIAA1671 | 75.6081428 | 41.1738116 | 110.042474 | 2.677484606 | 1.42087828 | 5.38E-06 | 1.11E-05 | Up |
| KIAA1841 | 579.3524849 | 808.7928908 | 349.9120789 | 0.432650261 | -1.208726821 | 1.03E-20 | 4.37E-20 | Down |
| KIAA1958 | 3050.547366 | 4795.119239 | 1305.975493 | 0.272377694 | -1.876319532 | 2.11E-132 | 9.01E-131 | Down |
| KIDINS220 | 4471.528951 | 2904.718132 | 6038.339771 | 2.078825994 | 1.055769004 | 1.54E-46 | 1.48E-45 | Up |
| KIF12 | 7.86587274 | 15.73174548 | 0 | 0.010445684 | -6.580949155 | 8.60E-06 | 1.76E-05 | Down |
| KIF14 | 1829.798764 | 2689.105325 | 970.4922034 | 0.360889106 | -1.4703725 | 7.10E-41 | 5.79E-40 | Down |
| KIF17 | 86.70474891 | 125.370701 | 48.03879684 | 0.383210927 | -1.383789398 | 1.20E-05 | 2.43E-05 | Down |
| KIF18B | 3814.351265 | 5249.838514 | 2378.864016 | 0.453140961 | -1.141968187 | 1.36E-74 | 2.43E-73 | Down |
| KIF21B | 2858.579129 | 3913.768928 | 1803.38933 | 0.460795277 | -1.117802166 | 7.57E-57 | 9.27E-56 | Down |
| KIF26A | 257.9329977 | 387.4644445 | 128.4015509 | 0.3312929 | -1.59382081 | 3.42E-17 | 1.26E-16 | Down |
| KIF3C | 1438.972523 | 1923.889197 | 954.0558486 | 0.495880743 | -1.011934894 | 2.49E-36 | 1.78E-35 | Down |
| KIFC3 | 46.8581075 | 17.6685408 | 76.04767419 | 4.331884419 | 2.11499475 | 7.43E-06 | 1.53E-05 | Up |
| KIZ | 469.7307833 | 636.738301 | 302.7232657 | 0.475388961 | -1.072819691 | 1.30E-17 | 4.88E-17 | Down |
| KLF10 | 9738.270177 | 829.1345422 | 18647.40581 | 22.50505692 | 4.492177309 | 0 | 0 | Up |
| KLF13 | 6809.727163 | 10910.23877 | 2709.215562 | 0.248320278 | -2.009726015 | 1.09E-212 | 1.07E-210 | Down |
| KLF16 | 2406.646029 | 3486.986343 | 1326.305715 | 0.380316344 | -1.394728154 | 4.40E-65 | 6.37E-64 | Down |
| KLF2 | 802.8287227 | 255.7131069 | 1349.944339 | 5.282344963 | 2.40117852 | 7.69E-85 | 1.72E-83 | Up |
| KLF6 | 8038.134454 | 2961.197434 | 13115.07147 | 4.429148979 | 2.147029524 | 0 | 0 | Up |
| KLF9 | 343.0160609 | 466.6625269 | 219.3695948 | 0.469922166 | -1.089506274 | 7.06E-13 | 2.15E-12 | Down |
| KLHDC7A | 6.89752484 | 2.184455176 | 11.61059451 | 5.283254902 | 2.401427018 | 0.031784776 | 0.045754113 | Up |
| KLHDC9 | 16.89736662 | 32.29450768 | 1.500225546 | 0.046273462 | -4.433671145 | 5.52E-07 | 1.22E-06 | Down |
| KLHL14 | 40.10453474 | 1.543291942 | 78.66577754 | 52.96023594 | 5.726837644 | 9.19E-12 | 2.66E-11 | Up |
| KLHL17 | 700.2209595 | 969.4532766 | 430.9886425 | 0.44453129 | -1.169643123 | 1.35E-23 | 6.43E-23 | Down |
| KLHL41 | 9.978506449 | 0 | 19.9570129 | 97.99577917 | 6.614647706 | 2.68E-06 | 5.64E-06 | Up |
| KLK2 | 8.792581722 | 0 | 17.58516344 | 86.34381465 | 6.432020927 | 7.29E-06 | 1.50E-05 | Up |
| KLKB1 | 19.90780235 | 2.245090838 | 37.57051387 | 16.90990497 | 4.079796647 | 1.96E-07 | 4.48E-07 | Up |
| KLRF2 | 4.166787212 | 7.732835432 | 0.600738991 | 0.077303502 | -3.693322424 | 0.018637472 | 0.027714448 | Down |
| KLRG1 | 11.38021901 | 19.16936187 | 3.591076147 | 0.186994573 | -2.418931691 | 0.003095992 | 0.005073184 | Down |
| KMO | 112.6424182 | 38.57563309 | 186.7092033 | 4.849781015 | 2.277919606 | 1.10E-13 | 3.49E-13 | Up |
| KNL1 | 4345.282992 | 5859.930953 | 2830.635031 | 0.483061016 | -1.049722666 | 4.78E-48 | 4.77E-47 | Down |
| KNTC1 | 6288.12011 | 9425.681162 | 3150.559058 | 0.334256246 | -1.580973576 | 4.04E-122 | 1.53E-120 | Down |
| KPNA7 | 4.006877376 | 8.013754752 | 0 | 0.020494616 | -5.60861124 | 0.001036536 | 0.001787838 | Down |
| KPTN | 299.0952976 | 412.9098721 | 185.280723 | 0.448799379 | -1.155857417 | 1.25E-11 | 3.59E-11 | Down |
| KRBA2 | 770.2110739 | 1154.670316 | 385.7518313 | 0.334106702 | -1.58161917 | 7.27E-45 | 6.66E-44 | Down |
| KRCC1 | 1754.347903 | 1103.15092 | 2405.544886 | 2.180106195 | 1.124398412 | 8.16E-43 | 7.05E-42 | Up |
| KREMEN2 | 277.0539686 | 453.6625047 | 100.4454326 | 0.221311012 | -2.175852857 | 2.22E-36 | 1.60E-35 | Down |
| KRT17 | 11.93906456 | 21.18854382 | 2.68958531 | 0.126639539 | -2.981200191 | 0.001062437 | 0.001829328 | Down |
| KRT18 | 25.38712602 | 41.81650585 | 8.957746199 | 0.213892726 | -2.225040675 | 6.12E-05 | 0.000118008 | Down |
| KRT7 | 30.6733985 | 20.18892573 | 41.15787128 | 2.03530779 | 1.025246983 | 0.029210804 | 0.042316769 | Up |
| KRT8 | 34.36689877 | 51.43803243 | 17.29576512 | 0.335969546 | -1.573597629 | 0.000508957 | 0.000903357 | Down |
| KSR2 | 156.9159756 | 216.9803637 | 96.85158745 | 0.446156905 | -1.164376927 | 4.61E-08 | 1.09E-07 | Down |
| KYAT1 | 614.349212 | 978.4574553 | 250.2409686 | 0.255716321 | -1.967383849 | 4.03E-62 | 5.48E-61 | Down |
| L2HGDH | 729.8108532 | 997.2819341 | 462.3397724 | 0.463687362 | -1.108775689 | 1.17E-27 | 6.48E-27 | Down |
| L3HYPDH | 232.620996 | 311.3028362 | 153.9391559 | 0.494242438 | -1.016709202 | 2.20E-07 | 5.01E-07 | Down |
| L3MBTL1 | 327.8658547 | 497.7744946 | 157.9572149 | 0.317354902 | -1.655830966 | 6.69E-28 | 3.72E-27 | Down |
| LAIR1 | 4567.170159 | 7069.041604 | 2065.298713 | 0.292163107 | -1.775154082 | 4.94E-155 | 2.78E-153 | Down |
| LAIR2 | 12.47333327 | 22.28332195 | 2.663344593 | 0.119782927 | -3.061505806 | 0.000709202 | 0.001240835 | Down |
| LAMA2 | 224.2262452 | 56.11548828 | 392.337002 | 6.994548156 | 2.806230863 | 7.59E-45 | 6.94E-44 | Up |
| LAMA5 | 99.71824055 | 59.60809898 | 139.8283821 | 2.350667872 | 1.233070714 | 0.000305387 | 0.000554671 | Up |
| LAMP3 | 20.12438037 | 0.356399685 | 39.89236105 | 100.576889 | 6.652155024 | 2.72E-07 | 6.16E-07 | Up |
| LAMP5 | 84.35172792 | 138.0199729 | 30.68348293 | 0.222378237 | -2.168912488 | 5.33E-14 | 1.71E-13 | Down |
| LANCL1 | 3271.902994 | 4651.610813 | 1892.195176 | 0.406781702 | -1.297673309 | 8.52E-95 | 2.18E-93 | Down |
| LARS2 | 1862.186187 | 1238.55202 | 2485.820353 | 2.006975884 | 1.005023281 | 1.12E-36 | 8.14E-36 | Up |
| LAT2 | 11643.71913 | 17658.7447 | 5628.693549 | 0.318750136 | -1.64950214 | 3.51E-184 | 2.55E-182 | Down |
| LCA5L | 21.94013189 | 9.977926271 | 33.9023375 | 3.400862405 | 1.765900638 | 0.003055238 | 0.005011733 | Up |
| LCN10 | 51.72862398 | 81.73867113 | 21.71857683 | 0.265924523 | -1.910911268 | 1.13E-05 | 2.29E-05 | Down |
| LCN6 | 164.1989909 | 310.8325714 | 17.56541045 | 0.056552024 | -4.144277531 | 2.76E-54 | 3.22E-53 | Down |
| LCP2 | 1232.479586 | 597.7067317 | 1867.25244 | 3.122561459 | 1.642729968 | 2.26E-76 | 4.21E-75 | Up |
| LCT | 30.49900194 | 10.02756146 | 50.97044243 | 5.099145627 | 2.350255541 | 4.50E-06 | 9.35E-06 | Up |
| LDAH | 852.9386131 | 566.3409882 | 1139.536238 | 2.011565285 | 1.008318561 | 1.88E-25 | 9.62E-25 | Up |
| LDB3 | 55.48658946 | 92.76790584 | 18.20527308 | 0.196197446 | -2.349621831 | 3.10E-10 | 8.31E-10 | Down |
| LDLR | 2199.760534 | 3091.742466 | 1307.778603 | 0.422972693 | -1.241363569 | 1.93E-44 | 1.75E-43 | Down |
| LEF1 | 22020.94602 | 32675.8146 | 11366.07744 | 0.347844234 | -1.523486688 | 1.36E-187 | 1.03E-185 | Down |
| LENG8 | 6198.78895 | 8914.198939 | 3483.378962 | 0.390774959 | -1.355590072 | 6.08E-150 | 3.16E-148 | Down |
| LEPR | 91.75760256 | 146.2506912 | 37.26451388 | 0.254833034 | -1.972375786 | 3.87E-13 | 1.19E-12 | Down |
| LETM2 | 662.5596721 | 303.585562 | 1021.533782 | 3.363773354 | 1.750080502 | 5.08E-47 | 4.92E-46 | Up |
| LFNG | 472.26182 | 861.126837 | 83.39680306 | 0.096851683 | -3.368079067 | 3.70E-18 | 1.43E-17 | Down |
| LGALS1 | 277.8387223 | 68.41167254 | 487.265772 | 7.111459901 | 2.830145759 | 4.66E-54 | 5.40E-53 | Up |
| LGALS3BP | 2883.670699 | 4506.49024 | 1260.851158 | 0.279804405 | -1.837509421 | 1.32E-166 | 8.27E-165 | Down |
| LGALS4 | 45.18052862 | 21.24354619 | 69.11751105 | 3.245127553 | 1.698275186 | 3.78E-05 | 7.39E-05 | Up |
| LGALS9 | 1407.62475 | 2595.498981 | 219.7505179 | 0.084653554 | -3.562285545 | 7.82E-238 | 9.12E-236 | Down |
| LGMN | 18.71190948 | 2.570278161 | 34.85354079 | 13.51767147 | 3.756774752 | 1.52E-06 | 3.27E-06 | Up |
| LGR6 | 904.4963253 | 1433.045637 | 375.9470139 | 0.262278208 | -1.930830153 | 2.77E-86 | 6.23E-85 | Down |
| LGSN | 18.12483259 | 8.227140207 | 28.02252497 | 3.423267831 | 1.775374171 | 0.007116397 | 0.011191785 | Up |
| LHPP | 662.4075782 | 1072.783232 | 252.0319246 | 0.234904786 | -2.089851987 | 4.69E-72 | 7.99E-71 | Down |
| LHX3 | 31.96586094 | 7.622564582 | 56.3091573 | 7.333880627 | 2.874576784 | 2.07E-06 | 4.40E-06 | Up |
| LIG1 | 3173.786042 | 4872.739838 | 1474.832247 | 0.302689106 | -1.724091341 | 3.73E-170 | 2.47E-168 | Down |
| LIG3 | 1858.190862 | 2741.269182 | 975.1125422 | 0.35570503 | -1.49124672 | 1.30E-76 | 2.44E-75 | Down |
| LILRA2 | 665.2170023 | 1050.232529 | 280.2014759 | 0.266794362 | -1.906199916 | 4.81E-71 | 8.04E-70 | Down |
| LILRA4 | 42.13759217 | 67.8917887 | 16.38339564 | 0.241192645 | -2.051742183 | 2.61E-06 | 5.51E-06 | Down |
| LILRB4 | 1629.990207 | 897.0756343 | 2362.90478 | 2.634505484 | 1.397532183 | 6.88E-58 | 8.60E-57 | Up |
| LIMS2 | 109.3565465 | 64.1440958 | 154.5689971 | 2.405810932 | 1.266523269 | 4.17E-05 | 8.12E-05 | Up |
| LINC02210-CRHR1 | 246.8347589 | 330.3327377 | 163.3367801 | 0.494567783 | -1.015759831 | 3.36E-07 | 7.57E-07 | Down |
| LINS1 | 709.90358 | 467.2420556 | 952.5651044 | 2.037834581 | 1.027036947 | 1.27E-20 | 5.38E-20 | Up |
| LIPN | 23.56760699 | 13.12104354 | 34.01417045 | 2.582374861 | 1.368698439 | 0.018064248 | 0.026911093 | Up |
| LLGL2 | 971.6003601 | 1334.650567 | 608.5501526 | 0.455967213 | -1.132998007 | 4.40E-34 | 2.94E-33 | Down |
| LMAN2L | 1327.456574 | 791.316435 | 1863.596713 | 2.355837966 | 1.236240314 | 2.87E-44 | 2.58E-43 | Up |
| LMF1 | 668.5915499 | 1037.528787 | 299.6543132 | 0.288794861 | -1.791883024 | 2.52E-61 | 3.38E-60 | Down |
| LMLN | 318.6942987 | 193.5746294 | 443.813968 | 2.291402918 | 1.196231163 | 1.19E-13 | 3.76E-13 | Up |
| LMNA | 113.8395515 | 169.4233524 | 58.2557506 | 0.343694586 | -1.540800968 | 2.24E-07 | 5.10E-07 | Down |
| LMO7 | 286.1025942 | 124.5499601 | 447.6552283 | 3.593095678 | 1.845227353 | 1.95E-19 | 7.93E-19 | Up |
| LMTK3 | 74.24392802 | 131.8360546 | 16.6518014 | 0.126507629 | -2.982703705 | 9.18E-17 | 3.32E-16 | Down |
| LNX1 | 815.1771288 | 1106.720044 | 523.634214 | 0.473212675 | -1.079439379 | 1.41E-29 | 8.31E-29 | Down |
| LOC101059906 | 5.755407805 | 1.096833294 | 10.41398232 | 9.445809559 | 3.239674449 | 0.015899905 | 0.023861039 | Up |
| LOC102723750 | 29.26126632 | 11.70850045 | 46.81403219 | 3.98463639 | 1.99444808 | 0.000128977 | 0.000242681 | Up |
| LOC102724474 | 12.35549754 | 19.33669026 | 5.374304828 | 0.278138708 | -1.846123561 | 0.012559526 | 0.019104417 | Down |
| LOC105369591 | 15.0451035 | 24.71389789 | 5.376309109 | 0.217369891 | -2.201775975 | 0.001952915 | 0.003279923 | Down |
| LOC105369914 | 16.40664894 | 6.26475775 | 26.54854014 | 4.236357567 | 2.082824364 | 0.005769577 | 0.009188267 | Up |
| LOC105372704 | 34.67453992 | 47.61485111 | 21.73422873 | 0.45666207 | -1.13080113 | 0.008028833 | 0.012532967 | Down |
| LOC105379752 | 19.17360978 | 31.49854755 | 6.848672007 | 0.217284485 | -2.202342928 | 0.000473983 | 0.000843657 | Down |
| LOC107984124 | 3.576571297 | 0 | 7.153142594 | 35.12207425 | 5.134306145 | 0.004117089 | 0.00666606 | Up |
| LOC107985876 | 4.32899622 | 8.357622945 | 0.300369495 | 0.038452357 | -4.700784164 | 0.00698825 | 0.010997733 | Down |
| LOC107987373 | 87.93444787 | 20.24213904 | 155.6267567 | 7.681219099 | 2.941335302 | 4.22E-17 | 1.56E-16 | Up |
| LOC112268119 | 2.685721658 | 0 | 5.371443316 | 26.37092315 | 4.720876171 | 0.014543429 | 0.021920233 | Up |
| LOC112268131 | 58.04949453 | 30.51019605 | 85.58879302 | 2.801706487 | 1.486305824 | 2.94E-05 | 5.78E-05 | Up |
| LOC112268444 | 6.428611869 | 10.76799507 | 2.08922867 | 0.194093353 | -2.365177382 | 0.034293039 | 0.049140365 | Down |
| LOC645177 | 144.0217022 | 73.05818214 | 214.9852223 | 2.942783607 | 1.55718146 | 6.87E-09 | 1.71E-08 | Up |
| LOC728392 | 142.8840593 | 65.08100693 | 220.6871116 | 3.393951716 | 1.762966041 | 2.55E-14 | 8.29E-14 | Up |
| LOXL2 | 243.659483 | 94.18430123 | 393.1346648 | 4.169774204 | 2.059969263 | 2.01E-27 | 1.10E-26 | Up |
| LPAR5 | 3692.198425 | 2239.391154 | 5145.005695 | 2.297346379 | 1.199968393 | 5.62E-70 | 9.16E-69 | Up |
| LPCAT1 | 1694.101773 | 2351.221604 | 1036.981943 | 0.441097273 | -1.180831255 | 8.25E-55 | 9.70E-54 | Down |
| LPCAT4 | 1897.003161 | 2827.498517 | 966.5078055 | 0.341820282 | -1.548690093 | 2.61E-106 | 8.11E-105 | Down |
| LPXN | 4787.32328 | 1080.45519 | 8494.191371 | 7.860913079 | 2.974696897 | 0 | 0 | Up |
| LRFN1 | 314.8582247 | 445.9291865 | 183.7872628 | 0.41215418 | -1.278743969 | 3.63E-17 | 1.34E-16 | Down |
| LRP1 | 1963.740667 | 2993.721887 | 933.7594474 | 0.311900929 | -1.680840244 | 1.27E-102 | 3.67E-101 | Down |
| LRP2BP | 76.24901067 | 12.82938986 | 139.6686315 | 10.8781209 | 3.44335746 | 0.000596676 | 0.001050742 | Up |
| LRP4 | 470.4624648 | 758.188663 | 182.7362665 | 0.241028918 | -2.052721849 | 3.22E-57 | 3.96E-56 | Down |
| LRP8 | 2769.271047 | 4006.943494 | 1531.5986 | 0.382241729 | -1.387442811 | 1.29E-91 | 3.15E-90 | Down |
| LRRC10 | 10.28170585 | 20.26466414 | 0.298747564 | 0.015838652 | -5.980406673 | 3.01E-05 | 5.92E-05 | Down |
| LRRC14B | 3603.204519 | 5939.9578 | 1266.451237 | 0.213208011 | -2.229666447 | 1.23E-244 | 1.49E-242 | Down |
| LRRC20 | 930.4626564 | 1485.170067 | 375.7552457 | 0.252958071 | -1.983029822 | 1.19E-69 | 1.92E-68 | Down |
| LRRC26 | 504.4767212 | 951.3874642 | 57.56597823 | 0.060506495 | -4.046766184 | 1.24E-122 | 4.76E-121 | Down |
| LRRC32 | 8.046523777 | 0 | 16.09304755 | 79.01837414 | 6.304116257 | 1.35E-05 | 2.71E-05 | Up |
| LRRC37A2 | 151.1074932 | 65.95498531 | 236.2600011 | 3.576340746 | 1.8384842 | 8.84E-11 | 2.44E-10 | Up |
| LRRC37A3 | 108.3750647 | 50.28771972 | 166.4624097 | 3.30382145 | 1.72413572 | 1.27E-10 | 3.49E-10 | Up |
| LRRC39 | 86.06857425 | 55.00613156 | 117.1310169 | 2.129877961 | 1.090770768 | 0.000198758 | 0.000367302 | Up |
| LRRC43 | 37.08587873 | 9.801386467 | 64.37037099 | 6.518784458 | 2.704602974 | 2.10E-07 | 4.78E-07 | Up |
| LRRC45 | 1244.071782 | 1991.865378 | 496.278187 | 0.24912564 | -2.005054585 | 6.43E-113 | 2.20E-111 | Down |
| LRRC46 | 64.85488473 | 19.97606443 | 109.733705 | 5.500194591 | 2.459482661 | 9.02E-12 | 2.61E-11 | Up |
| LRRC4B | 23.68669082 | 9.80496459 | 37.56841706 | 3.808550665 | 1.929242088 | 0.001083047 | 0.001862733 | Up |
| LRRC56 | 152.4478895 | 91.21591858 | 213.6798605 | 2.347832883 | 1.231329722 | 1.43E-06 | 3.07E-06 | Up |
| LRRC66 | 40.99320353 | 15.8215464 | 66.16486066 | 4.153619335 | 2.054369004 | 0.000336381 | 0.000607623 | Up |
| LRRC69 | 22.37651027 | 34.29142258 | 10.46159796 | 0.304776434 | -1.71417674 | 0.002618595 | 0.004333397 | Down |
| LRRC7 | 154.805603 | 38.23382007 | 271.3773859 | 7.086638364 | 2.825101429 | 1.28E-28 | 7.29E-28 | Up |
| LRRC73 | 11.31826298 | 1.128045656 | 21.5084803 | 19.33200295 | 4.272919215 | 0.000123341 | 0.000232486 | Up |
| LRRC75A | 27.44643193 | 17.66470464 | 37.22815923 | 2.106064383 | 1.074549541 | 0.025951622 | 0.037864199 | Up |
| LRRCC1 | 585.7918685 | 809.7568706 | 361.8268664 | 0.446752604 | -1.162451957 | 3.85E-24 | 1.87E-23 | Down |
| LRRD1 | 26.15708523 | 9.411719253 | 42.9024512 | 4.526889532 | 2.178520101 | 0.000354671 | 0.000638914 | Up |
| LRRN4CL | 8.616566985 | 1.484445341 | 15.74868863 | 10.65289641 | 3.413173832 | 0.002077777 | 0.003483302 | Up |
| LRTM2 | 2.818325079 | 5.636650159 | 0 | 0.029240411 | -5.095892592 | 0.008410717 | 0.013089919 | Down |
| LSG1 | 3458.168364 | 1977.222528 | 4939.1142 | 2.498187712 | 1.320881884 | 3.47E-103 | 1.02E-101 | Up |
| LSM1 | 1450.866838 | 918.7589979 | 1982.974678 | 2.157376988 | 1.109278301 | 9.13E-36 | 6.43E-35 | Up |
| LSMEM1 | 74.91909992 | 36.30264997 | 113.5355499 | 3.121916509 | 1.642431955 | 2.38E-06 | 5.03E-06 | Up |
| LSMEM2 | 19.58800359 | 0.356399685 | 38.81960749 | 97.84252413 | 6.612389718 | 3.87E-07 | 8.66E-07 | Up |
| LSP1 | 40.48899155 | 4.453336024 | 76.52464708 | 17.25923639 | 4.109296731 | 1.28E-12 | 3.84E-12 | Up |
| LST1 | 51.33666795 | 28.97995975 | 73.69337616 | 2.537799036 | 1.343577829 | 0.000351244 | 0.000633069 | Up |
| LTA | 533.2800167 | 228.8820297 | 837.6780037 | 3.658800786 | 1.871370866 | 2.55E-53 | 2.88E-52 | Up |
| LTB | 97.07035021 | 14.60754405 | 179.5331564 | 12.2120994 | 3.610239333 | 1.16E-25 | 6.00E-25 | Up |
| LTBP1 | 3.568270467 | 0 | 7.136540934 | 35.04865071 | 5.131287 | 0.003395932 | 0.005541761 | Up |
| LTBP2 | 161.6677599 | 94.15897214 | 229.1765477 | 2.437778853 | 1.285567255 | 2.46E-09 | 6.27E-09 | Up |
| LTBP4 | 4015.098098 | 5601.624609 | 2428.571587 | 0.433547418 | -1.205738304 | 3.37E-99 | 9.08E-98 | Down |
| LTF | 5.351499148 | 0 | 10.7029983 | 52.56478459 | 5.716024694 | 0.000325091 | 0.000588533 | Up |
| LTO1 | 1453.86555 | 661.2815918 | 2246.449508 | 3.398688012 | 1.764977933 | 4.36E-102 | 1.25E-100 | Up |
| LUC7L3 | 6661.332043 | 9444.268086 | 3878.396 | 0.410659884 | -1.283984072 | 1.05E-46 | 1.01E-45 | Down |
| LURAP1 | 6.31887368 | 11.74713516 | 0.890612199 | 0.075951317 | -3.71878121 | 0.003580339 | 0.005831714 | Down |
| LXN | 82.80794305 | 113.945175 | 51.67071109 | 0.45345232 | -1.140977235 | 0.000449667 | 0.000801919 | Down |
| LY6E | 293.1806183 | 432.0989137 | 154.262323 | 0.35711002 | -1.48555948 | 1.83E-19 | 7.46E-19 | Down |
| LY6G5B | 339.9065913 | 462.7800324 | 217.0331503 | 0.468857764 | -1.092777771 | 2.10E-14 | 6.84E-14 | Down |
| LY6G5C | 39.53982937 | 5.540957905 | 73.53870084 | 13.28667571 | 3.731908286 | 4.38E-12 | 1.29E-11 | Up |
| LY96 | 351.4054528 | 187.6266217 | 515.1842839 | 2.748655481 | 1.458726089 | 8.93E-23 | 4.08E-22 | Up |
| LYG1 | 302.3134811 | 160.2257593 | 444.4012028 | 2.772354737 | 1.47111187 | 1.11E-18 | 4.40E-18 | Up |
| LYPD3 | 57.69305341 | 92.45450119 | 22.93160562 | 0.248383727 | -2.009357437 | 1.01E-06 | 2.20E-06 | Down |
| LYPLAL1 | 347.6095278 | 230.9178293 | 464.3012264 | 2.011674762 | 1.008397076 | 5.67E-12 | 1.66E-11 | Up |
| LYRM9 | 175.1192432 | 244.9190086 | 105.3194778 | 0.429901933 | -1.217920499 | 1.80E-09 | 4.62E-09 | Down |
| LYSMD1 | 169.9991224 | 240.6890183 | 99.30922642 | 0.412297492 | -1.27824241 | 1.18E-09 | 3.06E-09 | Down |
| LYSMD2 | 948.9719136 | 572.6435743 | 1325.300253 | 2.314140774 | 1.210476629 | 2.06E-33 | 1.35E-32 | Up |
| LYSMD4 | 647.7473763 | 923.7685634 | 371.7261891 | 0.402223394 | -1.313931103 | 3.64E-27 | 1.98E-26 | Down |
| LYST | 1499.368038 | 510.1702314 | 2488.565844 | 4.87627472 | 2.285779407 | 7.60E-104 | 2.25E-102 | Up |
| LZTS1 | 139.9054485 | 278.6304117 | 1.180485408 | 0.004278847 | -7.86856217 | 3.26E-25 | 1.65E-24 | Down |
| MACROD1 | 1984.879837 | 2816.978247 | 1152.781427 | 0.409223727 | -1.2890383 | 3.08E-75 | 5.59E-74 | Down |
| MACROD2 | 188.4346559 | 59.81713218 | 317.0521796 | 5.287982885 | 2.402717507 | 4.22E-28 | 2.36E-27 | Up |
| MAD2L1 | 3026.803195 | 4160.103033 | 1893.503357 | 0.455148858 | -1.135589633 | 8.31E-76 | 1.54E-74 | Down |
| MAD2L1BP | 1457.335322 | 960.9984396 | 1953.672204 | 2.033552102 | 1.024001955 | 3.34E-36 | 2.38E-35 | Up |
| MADCAM1 | 3.715448793 | 0 | 7.430897585 | 36.49520439 | 5.189634996 | 0.003128528 | 0.005122868 | Up |
| MAFF | 75.03068426 | 11.40173595 | 138.6596326 | 12.13776772 | 3.601431212 | 2.89E-20 | 1.21E-19 | Up |
| MAGEB17 | 4.009568004 | 0 | 8.019136008 | 39.38529361 | 5.299585126 | 0.003109459 | 0.005094651 | Up |
| MAGI1 | 198.7023815 | 336.0727272 | 61.33203584 | 0.182524487 | -2.453838071 | 6.57E-25 | 3.28E-24 | Down |
| MAIP1 | 626.0747192 | 398.7190812 | 853.4303573 | 2.141821992 | 1.098838582 | 2.18E-19 | 8.87E-19 | Up |
| MAJIN | 5.073124367 | 0 | 10.14624873 | 49.81229263 | 5.638429908 | 0.000408088 | 0.00073191 | Up |
| MAML3 | 1519.376321 | 682.7284965 | 2356.024145 | 3.448959503 | 1.786161189 | 6.45E-89 | 1.51E-87 | Up |
| MAN2B1 | 5288.709537 | 7354.226876 | 3223.192197 | 0.43830049 | -1.190007803 | 3.56E-91 | 8.61E-90 | Down |
| MAN2C1 | 2295.897992 | 3083.407908 | 1508.388077 | 0.489148896 | -1.031654409 | 2.66E-44 | 2.40E-43 | Down |
| MANEA | 434.6179531 | 682.1466879 | 187.0892182 | 0.274288044 | -1.866236357 | 3.20E-31 | 1.97E-30 | Down |
| MAP10 | 79.26059704 | 41.31861489 | 117.2025792 | 2.836481897 | 1.504102657 | 2.66E-07 | 6.02E-07 | Up |
| MAP1A | 894.0070386 | 1286.521416 | 501.4926608 | 0.389813888 | -1.359142603 | 2.30E-41 | 1.90E-40 | Down |
| MAP1LC3B | 8545.546549 | 5312.304734 | 11778.78836 | 2.217333138 | 1.148825541 | 1.56E-101 | 4.38E-100 | Up |
| MAP2 | 202.1215513 | 123.6160787 | 280.6270239 | 2.270232984 | 1.182840362 | 1.33E-08 | 3.25E-08 | Up |
| MAP2K6 | 477.9922003 | 686.1078077 | 269.876593 | 0.393511166 | -1.345523521 | 6.88E-23 | 3.17E-22 | Down |
| MAP3K14 | 1830.329394 | 678.3729493 | 2982.285839 | 4.396178501 | 2.136249966 | 3.56E-143 | 1.73E-141 | Up |
| MAP3K15 | 6.958147043 | 1.069199055 | 12.84709503 | 11.76441091 | 3.556357176 | 0.005973616 | 0.009497067 | Up |
| MAP3K5 | 275.9997427 | 379.0950803 | 172.904405 | 0.455938303 | -1.133089481 | 1.15E-10 | 3.15E-10 | Down |
| MAP3K6 | 507.5931199 | 771.1400649 | 244.046175 | 0.316489491 | -1.6597705 | 6.06E-40 | 4.82E-39 | Down |
| MAP3K7CL | 89.47796058 | 4.016088789 | 174.9398324 | 43.17932265 | 5.432268707 | 1.41E-26 | 7.53E-26 | Up |
| MAP4K2 | 1420.774111 | 2074.66224 | 766.8859823 | 0.369623891 | -1.435870083 | 1.08E-55 | 1.29E-54 | Down |
| MAP4K3 | 1166.967418 | 682.3542315 | 1651.580604 | 2.420937862 | 1.27556605 | 1.89E-36 | 1.36E-35 | Up |
| MAP6D1 | 93.74011856 | 132.9037388 | 54.5764983 | 0.410632353 | -1.284080796 | 9.04E-06 | 1.85E-05 | Down |
| MAPK13 | 2460.555981 | 3415.22897 | 1505.882993 | 0.440983398 | -1.181203752 | 1.92E-66 | 2.88E-65 | Down |
| MAPK4 | 5.070500295 | 0 | 10.14100059 | 49.78909002 | 5.637757743 | 0.000402053 | 0.000721739 | Up |
| MAPK8IP1 | 156.7060818 | 43.41685533 | 269.9953082 | 6.210914497 | 2.634805707 | 5.97E-28 | 3.33E-27 | Up |
| MARCHF2 | 587.7938612 | 822.0105921 | 353.5771304 | 0.429950804 | -1.217756504 | 3.67E-18 | 1.42E-17 | Down |
| MARCHF7 | 5980.15163 | 3582.384648 | 8377.918613 | 2.338639396 | 1.225669424 | 1.81E-91 | 4.42E-90 | Up |
| MARVELD1 | 2177.849917 | 3232.845146 | 1122.854689 | 0.347328601 | -1.525626878 | 6.58E-113 | 2.25E-111 | Down |
| MARVELD2 | 31.91356249 | 6.678214974 | 57.14891 | 8.587567919 | 3.102249604 | 1.58E-08 | 3.85E-08 | Up |
| MASP2 | 4.623770975 | 0.356399685 | 8.891142266 | 22.42562867 | 4.487076524 | 0.007289566 | 0.011439481 | Up |
| MAST4 | 500.2653003 | 310.7558261 | 689.7747745 | 2.219966575 | 1.150537955 | 4.98E-20 | 2.06E-19 | Up |
| MAT2A | 11917.79973 | 17250.32414 | 6585.275332 | 0.381754096 | -1.38928446 | 2.44E-179 | 1.72E-177 | Down |
| MAU2 | 4449.592189 | 2922.7637 | 5976.420678 | 2.044426675 | 1.03169632 | 1.16E-55 | 1.39E-54 | Up |
| MBLAC1 | 56.81262979 | 33.08073225 | 80.54452732 | 2.44022274 | 1.287012841 | 0.000557503 | 0.000984736 | Up |
| MBNL3 | 3493.529701 | 4904.384162 | 2082.675239 | 0.424667172 | -1.235595506 | 8.57E-54 | 9.82E-53 | Down |
| MBOAT1 | 193.5901875 | 337.4280122 | 49.75236279 | 0.147450423 | -2.761698134 | 4.66E-40 | 3.72E-39 | Down |
| MBOAT2 | 657.1042336 | 947.4667175 | 366.7417498 | 0.386944743 | -1.369800534 | 7.68E-31 | 4.69E-30 | Down |
| MC1R | 219.7028131 | 39.07762633 | 400.3279998 | 10.29257514 | 3.363532075 | 5.17E-46 | 4.87E-45 | Up |
| MC4R | 2.957890208 | 5.915780416 | 0 | 0.02781261 | -5.168117039 | 0.004925617 | 0.007903209 | Down |
| MCEMP1 | 20.34485615 | 0.731222196 | 39.9584901 | 54.20858417 | 5.760449422 | 5.46E-07 | 1.21E-06 | Up |
| MCM2 | 19687.54975 | 26269.2747 | 13105.82481 | 0.498906302 | -1.003159201 | 6.44E-80 | 1.30E-78 | Down |
| MCM5 | 12107.07421 | 18151.74347 | 6062.404953 | 0.333990781 | -1.582119813 | 4.33E-189 | 3.41E-187 | Down |
| MCM8 | 2976.126323 | 3979.129879 | 1973.122766 | 0.495878421 | -1.011941649 | 9.45E-38 | 7.09E-37 | Down |
| MCTP1 | 12.20021455 | 1.42559874 | 22.97483035 | 15.7410209 | 3.976457207 | 0.000106493 | 0.000201633 | Up |
| MCU | 6378.611415 | 2711.717325 | 10045.5055 | 3.705342238 | 1.889606801 | 3.02E-219 | 3.20E-217 | Up |
| MDFI | 911.1080636 | 1255.474784 | 566.7413428 | 0.451349094 | -1.147684383 | 2.71E-31 | 1.68E-30 | Down |
| MDGA1 | 237.5581039 | 358.2437726 | 116.8724353 | 0.326217819 | -1.616092505 | 5.05E-20 | 2.09E-19 | Down |
| MDM4 | 5305.607032 | 7397.523749 | 3213.690314 | 0.43444721 | -1.202747211 | 2.90E-63 | 4.03E-62 | Down |
| MDP1 | 53.80581997 | 30.06937069 | 77.54226924 | 2.573298635 | 1.363618893 | 0.000175807 | 0.000326499 | Up |
| MECOM | 3.891063292 | 0 | 7.782126584 | 38.19605865 | 5.255351873 | 0.00282896 | 0.004660392 | Up |
| MED10 | 1605.074471 | 841.7499891 | 2368.398954 | 2.812598174 | 1.491903454 | 1.61E-78 | 3.16E-77 | Up |
| MED26 | 810.1684426 | 476.4838925 | 1143.852993 | 2.401442709 | 1.263901391 | 3.74E-33 | 2.44E-32 | Up |
| MED27 | 2617.072265 | 1425.768189 | 3808.376342 | 2.670637719 | 1.417184283 | 1.15E-96 | 3.00E-95 | Up |
| MED6 | 1663.902621 | 1060.307162 | 2267.49808 | 2.138043988 | 1.096291535 | 6.51E-50 | 6.74E-49 | Up |
| MED9 | 1542.990621 | 2237.940341 | 848.0409019 | 0.378874355 | -1.400208604 | 6.62E-57 | 8.12E-56 | Down |
| MEF2C | 8468.129061 | 4568.156023 | 12368.1021 | 2.707361529 | 1.436887552 | 6.54E-115 | 2.26E-113 | Up |
| MEGF6 | 22.12641323 | 37.68477096 | 6.568055505 | 0.174212688 | -2.521078399 | 7.20E-05 | 0.000138335 | Down |
| MEI1 | 55.45442001 | 32.98862618 | 77.92021384 | 2.356954419 | 1.236923859 | 0.000883214 | 0.001531957 | Up |
| MEIG1 | 30.94823982 | 9.83438789 | 52.06209175 | 5.265415031 | 2.396547252 | 7.73E-06 | 1.59E-05 | Up |
| MEP1A | 2.086222248 | 0 | 4.172444495 | 20.48689697 | 4.356629579 | 0.032089492 | 0.046159218 | Up |
| MERTK | 153.5794115 | 228.1743475 | 78.98447539 | 0.345943392 | -1.531392112 | 1.69E-12 | 5.06E-12 | Down |
| METRN | 80.23297344 | 159.8668298 | 0.59911706 | 0.003730902 | -8.066259708 | 2.67E-14 | 8.68E-14 | Down |
| METTL3 | 2283.121908 | 3154.967332 | 1411.276484 | 0.447315347 | -1.160635839 | 1.42E-56 | 1.73E-55 | Down |
| METTL7A | 1200.276883 | 1708.75091 | 691.8028569 | 0.404814294 | -1.304667863 | 2.33E-55 | 2.77E-54 | Down |
| MEX3A | 1759.417676 | 2677.531169 | 841.3041827 | 0.314216428 | -1.670169484 | 2.01E-90 | 4.82E-89 | Down |
| MEX3B | 301.1957512 | 450.0287102 | 152.3627921 | 0.338486653 | -1.562829146 | 4.18E-22 | 1.87E-21 | Down |
| MFAP4 | 40.11602866 | 76.94173302 | 3.290324301 | 0.042643876 | -4.55151763 | 6.82E-15 | 2.28E-14 | Down |
| MFGE8 | 1170.645378 | 1990.05731 | 351.2334457 | 0.176444502 | -2.50271362 | 3.80E-99 | 1.02E-97 | Down |
| MFSD12 | 1235.413124 | 635.4626722 | 1835.363576 | 2.887639949 | 1.529890868 | 6.29E-65 | 9.08E-64 | Up |
| MFSD2A | 890.6077123 | 401.3950199 | 1379.820405 | 3.436626257 | 1.780992963 | 7.91E-68 | 1.22E-66 | Up |
| MFSD4B | 887.0013649 | 1250.406122 | 523.5966075 | 0.418738093 | -1.255879927 | 4.01E-34 | 2.69E-33 | Down |
| MGAM | 45.99132925 | 1.543291942 | 90.43936655 | 60.93015909 | 5.929084602 | 6.12E-13 | 1.87E-12 | Up |
| MGAM2 | 5.282017729 | 0.385822986 | 10.17821247 | 25.64786289 | 4.680766713 | 0.003979422 | 0.006452954 | Up |
| MGARP | 9.710181356 | 1.791209838 | 17.62915287 | 9.676538074 | 3.274490994 | 0.001804956 | 0.003039522 | Up |
| MGAT3 | 24.6240086 | 0.356399685 | 48.89161752 | 123.248139 | 6.945422053 | 4.54E-08 | 1.07E-07 | Up |
| MGST3 | 661.4929764 | 958.7507775 | 364.2351753 | 0.379932162 | -1.396186253 | 3.32E-39 | 2.59E-38 | Down |
| MICALCL | 35.70835991 | 13.3198504 | 58.09686943 | 4.367401484 | 2.12677516 | 6.13E-06 | 1.27E-05 | Up |
| MICOS10-NBL1 | 11.28386208 | 4.392700361 | 18.1750238 | 4.123388661 | 2.043830453 | 0.009817663 | 0.015124479 | Up |
| MID1IP1 | 6874.045205 | 9794.557789 | 3953.532621 | 0.403647218 | -1.308833145 | 3.16E-98 | 8.40E-97 | Down |
| MILR1 | 1698.873826 | 2477.223988 | 920.5236632 | 0.371555763 | -1.428349347 | 2.87E-63 | 4.00E-62 | Down |
| MINDY1 | 604.3108872 | 810.5175575 | 398.1042169 | 0.491244023 | -1.025488242 | 7.48E-19 | 2.98E-18 | Down |
| MINPP1 | 51.87999227 | 22.34421565 | 81.41576888 | 3.656939909 | 1.870636921 | 1.64E-06 | 3.51E-06 | Up |
| MISP3 | 35.6273343 | 57.289591 | 13.9650776 | 0.244103625 | -2.034434378 | 1.41E-05 | 2.83E-05 | Down |
| MITD1 | 1060.524361 | 592.7977913 | 1528.25093 | 2.577343292 | 1.365884711 | 2.40E-36 | 1.72E-35 | Up |
| MITF | 14.76292782 | 0.385822986 | 29.14003265 | 73.47528171 | 6.19918708 | 4.50E-06 | 9.35E-06 | Up |
| MIX23 | 711.6257871 | 368.9093858 | 1054.342188 | 2.857268249 | 1.514636487 | 3.67E-44 | 3.29E-43 | Up |
| MKI67 | 30227.18766 | 40363.99319 | 20090.38212 | 0.497732584 | -1.006557259 | 2.43E-74 | 4.32E-73 | Down |
| MKRN2OS | 95.50092917 | 2.888043133 | 188.1138152 | 63.87776091 | 5.997241838 | 3.97E-26 | 2.09E-25 | Up |
| MKRN3 | 904.4199744 | 1267.773466 | 541.0664826 | 0.426838647 | -1.228237288 | 1.47E-26 | 7.84E-26 | Down |
| MLXIP | 9460.081408 | 13743.03617 | 5177.126648 | 0.376711582 | -1.408467705 | 1.01E-138 | 4.70E-137 | Down |
| MMADHC | 1756.072285 | 1055.99303 | 2456.151541 | 2.32630351 | 1.218039336 | 2.62E-64 | 3.71E-63 | Up |
| MME | 34493.94757 | 59525.27288 | 9462.622249 | 0.158967981 | -2.653191885 | 0 | 0 | Down |
| MMP10 | 38.84354466 | 0.742222671 | 76.94486664 | 104.2540003 | 6.703958931 | 7.38E-10 | 1.94E-09 | Up |
| MMP11 | 373.7331742 | 568.0756566 | 179.3906918 | 0.315812976 | -1.662857646 | 4.37E-34 | 2.92E-33 | Down |
| MMP14 | 445.5543397 | 595.2183414 | 295.8903381 | 0.497182989 | -1.008151161 | 9.79E-16 | 3.39E-15 | Down |
| MMP24OS | 747.4407764 | 1145.376034 | 349.5055192 | 0.305259012 | -1.711894206 | 2.12E-54 | 2.47E-53 | Down |
| MMP28 | 27.15581987 | 44.75802034 | 9.553619397 | 0.213400598 | -2.228363874 | 1.35E-05 | 2.73E-05 | Down |
| MMP9 | 2.241655139 | 0 | 4.483310277 | 22.00698452 | 4.45988957 | 0.026231626 | 0.038236537 | Up |
| MNS1 | 322.2692517 | 554.9472229 | 89.59128041 | 0.161473367 | -2.630631865 | 3.40E-33 | 2.22E-32 | Down |
| MNT | 1566.027346 | 2125.559693 | 1006.494999 | 0.473533817 | -1.078460637 | 8.30E-39 | 6.42E-38 | Down |
| MOB3A | 1535.394686 | 303.8705917 | 2766.918781 | 9.100575012 | 3.185957704 | 1.28E-196 | 1.07E-194 | Up |
| MOB4 | 2.691207241 | 0 | 5.382414482 | 26.41976662 | 4.723545818 | 0.014885246 | 0.022418377 | Up |
| MOBP | 4.676542746 | 0.751434084 | 8.601651408 | 11.5839016 | 3.534049348 | 0.021430135 | 0.031613038 | Up |
| MOCS3 | 573.6405488 | 380.1435848 | 767.1375128 | 2.018107257 | 1.013002852 | 3.62E-15 | 1.22E-14 | Up |
| MOGS | 2464.097751 | 3364.282112 | 1563.91339 | 0.464823689 | -1.105244498 | 1.09E-53 | 1.25E-52 | Down |
| MOK | 292.9434609 | 142.5495301 | 443.3373917 | 3.106031958 | 1.635072674 | 4.39E-21 | 1.89E-20 | Up |
| MORN1 | 70.51015427 | 31.0891926 | 109.9311159 | 3.541147267 | 1.824216843 | 1.00E-08 | 2.46E-08 | Up |
| MORN2 | 142.4657644 | 53.61890143 | 231.3126273 | 4.318775207 | 2.110622226 | 2.09E-20 | 8.77E-20 | Up |
| MOSPD1 | 510.7217305 | 329.406561 | 692.0369001 | 2.100895046 | 1.071004092 | 4.80E-20 | 1.99E-19 | Up |
| MOV10 | 3722.75486 | 5182.873513 | 2262.636208 | 0.436564721 | -1.195732544 | 2.08E-67 | 3.17E-66 | Down |
| MOXD1 | 10.34307963 | 3.700112879 | 16.98604638 | 4.597714119 | 2.200916763 | 0.009089447 | 0.014069827 | Up |
| MPDU1 | 1762.390635 | 924.5666627 | 2600.214607 | 2.814127032 | 1.492687454 | 1.16E-53 | 1.32E-52 | Up |
| MPEG1 | 47.40120482 | 65.32202663 | 29.48038302 | 0.451916111 | -1.145873103 | 0.00252308 | 0.004184819 | Down |
| MPHOSPH9 | 3347.242812 | 5063.275962 | 1631.209662 | 0.322176054 | -1.634078827 | 8.95E-84 | 1.96E-82 | Down |
| MPND | 853.0634773 | 1186.273568 | 519.8533862 | 0.438320827 | -1.189940864 | 4.77E-23 | 2.22E-22 | Down |
| MPO | 81.40952849 | 142.2980421 | 20.52101489 | 0.144248853 | -2.793368246 | 1.44E-14 | 4.73E-14 | Down |
| MPP1 | 4414.241154 | 7021.321224 | 1807.161084 | 0.257396345 | -1.957936525 | 3.62E-225 | 3.99E-223 | Down |
| MPP4 | 4.034140526 | 0 | 8.068281051 | 39.60614436 | 5.307652357 | 0.002150824 | 0.003596635 | Up |
| MPP7 | 1190.490501 | 1753.306611 | 627.6743905 | 0.358004425 | -1.481950675 | 7.78E-30 | 4.60E-29 | Down |
| MPPED1 | 11.05662414 | 17.95330435 | 4.159943927 | 0.232442835 | -2.105052139 | 0.011970717 | 0.018250804 | Down |
| MPPED2 | 3611.253558 | 5104.042867 | 2118.464248 | 0.415063175 | -1.268597156 | 1.86E-109 | 6.04E-108 | Down |
| MPST | 1695.918936 | 2410.152042 | 981.6858296 | 0.407357826 | -1.295631469 | 5.20E-44 | 4.64E-43 | Down |
| MPZ | 447.6243586 | 163.4704521 | 731.778265 | 4.48038228 | 2.163621833 | 1.06E-43 | 9.40E-43 | Up |
| MRAP2 | 972.0953278 | 1543.908347 | 400.2823082 | 0.259244365 | -1.947615462 | 3.83E-63 | 5.30E-62 | Down |
| MRC1 | 7.183904025 | 0.365611098 | 14.00219695 | 35.29993772 | 5.141593733 | 0.000450233 | 0.000802826 | Up |
| MRC2 | 508.5038036 | 805.4838691 | 211.5237381 | 0.26256877 | -1.929232762 | 3.76E-43 | 3.28E-42 | Down |
| MRE11 | 1923.185845 | 2663.090331 | 1183.281359 | 0.444356265 | -1.170211266 | 1.65E-42 | 1.42E-41 | Down |
| MRI1 | 1806.198257 | 2427.234903 | 1185.161611 | 0.48822502 | -1.034381865 | 5.06E-42 | 4.29E-41 | Down |
| MRM3 | 1327.884239 | 685.3274572 | 1970.441021 | 2.874255298 | 1.523188211 | 8.71E-69 | 1.37E-67 | Up |
| MROH1 | 529.9202691 | 793.3084654 | 266.5320728 | 0.336042669 | -1.573283665 | 4.88E-33 | 3.16E-32 | Down |
| MROH8 | 330.9032977 | 189.3513126 | 472.4552827 | 2.494297221 | 1.318633387 | 7.36E-17 | 2.68E-16 | Up |
| MRPL1 | 992.7645161 | 301.3592177 | 1684.169815 | 5.590629472 | 2.483010731 | 3.61E-105 | 1.10E-103 | Up |
| MRPL15 | 2642.867253 | 1655.866019 | 3629.868487 | 2.192390054 | 1.132504495 | 1.29E-44 | 1.17E-43 | Up |
| MRPL32 | 1417.145918 | 765.8298589 | 2068.461977 | 2.700677623 | 1.433321437 | 5.02E-71 | 8.37E-70 | Up |
| MRPL39 | 1570.987902 | 969.2442354 | 2172.731569 | 2.241399443 | 1.164399776 | 3.91E-51 | 4.17E-50 | Up |
| MRPL46 | 1344.513708 | 872.5587531 | 1816.468663 | 2.080616195 | 1.05701086 | 4.33E-30 | 2.59E-29 | Up |
| MRPL47 | 2160.937206 | 1291.071443 | 3030.802968 | 2.347255152 | 1.230974675 | 1.93E-64 | 2.75E-63 | Up |
| MRPL58 | 1637.268825 | 1062.904367 | 2211.633284 | 2.081405419 | 1.057558003 | 1.56E-38 | 1.20E-37 | Up |
| MRPS11 | 2379.963311 | 1538.375278 | 3221.551343 | 2.094269449 | 1.066447071 | 6.27E-42 | 5.28E-41 | Up |
| MRPS18A | 1556.003933 | 860.4885111 | 2251.519354 | 2.616486226 | 1.387630664 | 5.37E-69 | 8.53E-68 | Up |
| MRPS18B | 4132.148684 | 2635.588983 | 5628.708384 | 2.135775403 | 1.094759942 | 1.99E-58 | 2.52E-57 | Up |
| MRPS18C | 1289.487246 | 715.286807 | 1863.687685 | 2.605165901 | 1.381375248 | 6.81E-66 | 1.01E-64 | Up |
| MRPS22 | 2056.761612 | 1293.64311 | 2819.880115 | 2.179753981 | 1.124165314 | 2.16E-52 | 2.39E-51 | Up |
| MRPS31 | 1116.679264 | 680.3890612 | 1552.969467 | 2.283243683 | 1.191084842 | 2.31E-35 | 1.61E-34 | Up |
| MS4A1 | 259.5549657 | 4.697675797 | 514.4122557 | 107.4205696 | 6.747126466 | 2.14E-55 | 2.55E-54 | Up |
| MS4A15 | 8.762567008 | 1.42559874 | 16.09953528 | 11.05857808 | 3.46709399 | 0.001945366 | 0.003268827 | Up |
| MS4A7 | 4.506865548 | 0.365611098 | 8.648119997 | 21.79985286 | 4.446246493 | 0.007170241 | 0.011264969 | Up |
| MSANTD3 | 445.299911 | 612.2265139 | 278.373308 | 0.454686983 | -1.137054393 | 6.03E-19 | 2.41E-18 | Down |
| MSGN1 | 6.932274119 | 0.751434084 | 13.11311416 | 17.67677815 | 4.143783441 | 0.003091809 | 0.005067527 | Up |
| MSH6 | 4523.965814 | 6818.739103 | 2229.192526 | 0.326933382 | -1.612931404 | 2.05E-178 | 1.44E-176 | Down |
| MSI2 | 14789.11392 | 20780.65998 | 8797.567852 | 0.423356287 | -1.240055783 | 9.46E-81 | 1.94E-79 | Down |
| MSR1 | 548.8785643 | 768.4697756 | 329.287353 | 0.428560286 | -1.22242993 | 4.18E-25 | 2.11E-24 | Down |
| MSS51 | 146.1313108 | 220.2143222 | 72.0482994 | 0.327283701 | -1.61138634 | 2.90E-12 | 8.61E-12 | Down |
| MT1F | 147.829291 | 213.3685127 | 82.29006924 | 0.385571415 | -1.374929997 | 1.05E-10 | 2.88E-10 | Down |
| MT1G | 2621.500452 | 4280.041598 | 962.9593064 | 0.224978157 | -2.152143159 | 7.74E-197 | 6.47E-195 | Down |
| MT1H | 934.3463537 | 1433.634643 | 435.0580644 | 0.303389587 | -1.720756527 | 6.54E-66 | 9.69E-65 | Down |
| MT1X | 9818.746048 | 16390.26157 | 3247.230525 | 0.19811371 | -2.335599371 | 0 | 0 | Down |
| MT2A | 1099.667226 | 1712.61359 | 486.7208631 | 0.284144809 | -1.815301736 | 4.20E-66 | 6.26E-65 | Down |
| MTCP1 | 90.45915042 | 123.6068835 | 57.31141739 | 0.463221929 | -1.110224543 | 0.000936122 | 0.001620688 | Down |
| MTFP1 | 1556.780021 | 869.3353539 | 2244.224688 | 2.5811696 | 1.368024939 | 8.70E-47 | 8.38E-46 | Up |
| MTG2 | 1997.276718 | 1213.611108 | 2780.942328 | 2.290862144 | 1.195890645 | 1.30E-45 | 1.21E-44 | Up |
| MTHFD2 | 12535.04864 | 19927.32097 | 5142.776304 | 0.258076015 | -1.954132026 | 0 | 0 | Down |
| MTMR6 | 3558.006921 | 1704.522775 | 5411.491067 | 3.175139234 | 1.666819857 | 2.01E-93 | 5.10E-92 | Up |
| MTO1 | 2443.302451 | 1463.656118 | 3422.948784 | 2.33772537 | 1.225105456 | 8.08E-63 | 1.11E-61 | Up |
| MTR | 2549.969554 | 3665.287894 | 1434.651214 | 0.391417003 | -1.353221667 | 3.48E-95 | 8.94E-94 | Down |
| MTRF1L | 1239.15125 | 568.5594355 | 1909.743065 | 3.35794898 | 1.747580311 | 2.94E-69 | 4.70E-68 | Up |
| MTRNR2L2 | 11.12726476 | 20.77892276 | 1.47560676 | 0.071470625 | -3.806505777 | 0.000217065 | 0.000399537 | Down |
| MTSS1 | 3679.837352 | 41.40509573 | 7318.269609 | 177.1072852 | 7.468479747 | 0 | 0 | Up |
| MTSS2 | 131.7172843 | 70.55901596 | 192.8755526 | 2.730467352 | 1.449147907 | 1.25E-08 | 3.06E-08 | Up |
| MTTP | 24.35818921 | 8.768766958 | 39.94761147 | 4.539706788 | 2.182599119 | 0.000539108 | 0.000953579 | Up |
| MTURN | 145.6921101 | 220.5955107 | 70.78870949 | 0.32100017 | -1.639354034 | 9.06E-10 | 2.37E-09 | Down |
| MTUS2 | 12.9847897 | 2.968890683 | 23.00068872 | 7.77125405 | 2.958147426 | 0.000630951 | 0.001108167 | Up |
| MUC1 | 25.33917125 | 41.12033218 | 9.558010311 | 0.232407448 | -2.10527179 | 0.000158072 | 0.000294984 | Down |
| MUL1 | 1907.951441 | 1050.92921 | 2764.973672 | 2.631375004 | 1.395816865 | 2.30E-77 | 4.37E-76 | Up |
| MUSTN1 | 15.72626436 | 25.79436353 | 5.658165191 | 0.219393854 | -2.18840498 | 0.002425995 | 0.004034405 | Down |
| MVK | 977.0964597 | 568.1859355 | 1386.006984 | 2.438950451 | 1.286260449 | 2.19E-40 | 1.77E-39 | Up |
| MX2 | 800.5825104 | 1225.458828 | 375.7061932 | 0.306519236 | -1.70595048 | 1.91E-58 | 2.43E-57 | Down |
| MXD3 | 2871.385057 | 5222.718933 | 520.0511815 | 0.099566235 | -3.328199608 | 0 | 0 | Down |
| MXRA7 | 57.25008574 | 5.884568054 | 108.6156034 | 18.42127449 | 4.203300974 | 1.46E-17 | 5.47E-17 | Up |
| MYBL1 | 403.4837955 | 567.0259367 | 239.9416543 | 0.42319878 | -1.240592626 | 4.99E-17 | 1.83E-16 | Down |
| MYBPC2 | 46.70162275 | 11.10955005 | 82.29369545 | 7.423706981 | 2.892139767 | 1.92E-11 | 5.47E-11 | Up |
| MYBPC3 | 35.70040322 | 16.20762743 | 55.19317902 | 3.403584478 | 1.767054918 | 0.000305787 | 0.000555325 | Up |
| MYBPH | 173.7015039 | 326.2306842 | 21.17232356 | 0.064907626 | -3.9454682 | 2.04E-47 | 2.00E-46 | Down |
| MYBPHL | 5.719222167 | 0.365611098 | 11.07283324 | 27.9025905 | 4.802327164 | 0.002347352 | 0.003909486 | Up |
| MYC | 16401.23076 | 26020.18452 | 6782.277009 | 0.260660307 | -1.939757185 | 0 | 0 | Down |
| MYCL | 98.51021667 | 168.7149052 | 28.3055281 | 0.167944204 | -2.573946088 | 5.86E-17 | 2.14E-16 | Down |
| MYL3 | 2.547081602 | 0 | 5.094163204 | 25.00116717 | 4.643923543 | 0.023163436 | 0.033996404 | Up |
| MYL5 | 154.8463318 | 83.03431935 | 226.6583442 | 2.729890014 | 1.448842827 | 2.39E-11 | 6.79E-11 | Up |
| MYL6 | 20620.15941 | 13177.55308 | 28062.76574 | 2.129630571 | 1.090603186 | 4.85E-73 | 8.43E-72 | Up |
| MYLK3 | 104.171968 | 184.7672197 | 23.57671639 | 0.127526013 | -2.971136532 | 1.56E-22 | 7.08E-22 | Down |
| MYLK4 | 63.95207394 | 42.12863753 | 85.77551035 | 2.03203787 | 1.022927289 | 0.002186406 | 0.003653492 | Up |
| MYLPF | 15.60843071 | 6.726061101 | 24.49080033 | 3.659252881 | 1.87154912 | 0.007085695 | 0.011144765 | Up |
| MYMX | 3.062210464 | 5.829299576 | 0.295121352 | 0.054877965 | -4.187629197 | 0.02417712 | 0.03541297 | Down |
| MYNN | 1793.630052 | 877.5135513 | 2709.746553 | 3.088795254 | 1.627044243 | 1.07E-70 | 1.78E-69 | Up |
| MYO10 | 1076.393035 | 1474.938929 | 677.8471408 | 0.459532316 | -1.121761775 | 4.67E-33 | 3.03E-32 | Down |
| MYO15A | 16.4899201 | 4.076724451 | 28.90311576 | 7.108756717 | 2.829597262 | 0.000101997 | 0.00019349 | Up |
| MYO18A | 13592.38893 | 21248.33935 | 5936.438521 | 0.279380283 | -1.839697886 | 6.09E-219 | 6.36E-217 | Down |
| MYO19 | 2670.382994 | 3573.900827 | 1766.865161 | 0.494351682 | -1.016390353 | 3.91E-53 | 4.41E-52 | Down |
| MYO1A | 57.4685962 | 19.9645398 | 94.9726526 | 4.729281736 | 2.24162109 | 9.65E-08 | 2.25E-07 | Up |
| MYO1C | 159.4880596 | 74.61220845 | 244.3639108 | 3.276777427 | 1.712277682 | 1.17E-13 | 3.70E-13 | Up |
| MYO1E | 34.20277291 | 11.46416067 | 56.94138516 | 4.97306631 | 2.314135668 | 5.91E-07 | 1.30E-06 | Up |
| MYO1F | 435.8840141 | 711.5603168 | 160.2077114 | 0.225075848 | -2.151516841 | 1.27E-45 | 1.19E-44 | Down |
| MYO5C | 1526.439768 | 2104.34623 | 948.5333073 | 0.450730994 | -1.149661437 | 2.36E-31 | 1.47E-30 | Down |
| MYO7A | 294.5819964 | 534.4136479 | 54.75034495 | 0.102543878 | -3.285686732 | 3.02E-58 | 3.81E-57 | Down |
| MYO7B | 262.2656374 | 75.54914377 | 448.982131 | 5.928602663 | 2.56769211 | 8.16E-37 | 5.94E-36 | Up |
| MYOM1 | 53.71925374 | 10.518022 | 96.92048548 | 9.296646329 | 3.216710372 | 4.19E-11 | 1.17E-10 | Up |
| MYORG | 1196.699325 | 1686.778238 | 706.6204116 | 0.41890134 | -1.255317595 | 7.24E-40 | 5.74E-39 | Down |
| MYOZ3 | 42.45536243 | 73.02161065 | 11.8891142 | 0.162889382 | -2.618035529 | 1.62E-07 | 3.71E-07 | Down |
| MYT1 | 61.80365829 | 28.04661061 | 95.56070597 | 3.408338434 | 1.769068597 | 1.14E-06 | 2.46E-06 | Up |
| MZB1 | 6550.370115 | 10237.02732 | 2863.712907 | 0.279745269 | -1.837814361 | 1.46E-280 | 2.26E-278 | Down |
| MZT2A | 1960.817713 | 2722.163403 | 1199.472022 | 0.440652643 | -1.182286236 | 1.64E-45 | 1.52E-44 | Down |
| N6AMT1 | 153.7249786 | 205.9892891 | 101.4606681 | 0.492473407 | -1.021882273 | 3.30E-06 | 6.92E-06 | Down |
| NAAA | 1531.840475 | 2290.877639 | 772.8033108 | 0.337302504 | -1.567885067 | 4.12E-71 | 6.89E-70 | Down |
| NAALAD2 | 3.861769888 | 0 | 7.723539777 | 37.93412574 | 5.245424383 | 0.002866951 | 0.004721857 | Up |
| NAALADL2 | 725.6340323 | 154.9537215 | 1296.314343 | 8.366334118 | 3.064595615 | 1.58E-103 | 4.66E-102 | Up |
| NAB1 | 1132.354621 | 1532.096457 | 732.6127846 | 0.478158589 | -1.064438905 | 1.70E-23 | 8.07E-23 | Down |
| NAIP | 30.91610104 | 43.96795154 | 17.86425054 | 0.406533771 | -1.298552891 | 0.007174759 | 0.011270791 | Down |
| NANP | 993.3103868 | 1515.182795 | 471.4379789 | 0.311155339 | -1.684293095 | 8.62E-60 | 1.12E-58 | Down |
| NAP1L1 | 22502.56305 | 30374.27881 | 14630.84729 | 0.481692031 | -1.053817038 | 6.63E-123 | 2.56E-121 | Down |
| NAP1L5 | 37.6844582 | 6.582788827 | 68.78612757 | 10.40299812 | 3.378927465 | 1.52E-08 | 3.70E-08 | Up |
| NAPEPLD | 42.58214911 | 65.49267509 | 19.67162313 | 0.300531983 | -1.734409563 | 1.00E-05 | 2.04E-05 | Down |
| NAPSA | 89.60345145 | 18.47498532 | 160.7319176 | 8.708024136 | 3.122345406 | 3.02E-22 | 1.36E-21 | Up |
| NARF | 4278.04338 | 6304.742546 | 2251.344214 | 0.357080142 | -1.485680192 | 7.34E-112 | 2.46E-110 | Down |
| NAT1 | 628.813534 | 350.1813895 | 907.4456784 | 2.590072677 | 1.37299258 | 2.18E-29 | 1.27E-28 | Up |
| NAT16 | 46.69348814 | 13.70567339 | 79.68130289 | 5.824067978 | 2.542027195 | 7.27E-09 | 1.80E-08 | Up |
| NATD1 | 724.5981698 | 1053.947445 | 395.2488946 | 0.374942453 | -1.415258912 | 2.48E-41 | 2.05E-40 | Down |
| NAV2 | 1079.457219 | 2039.078763 | 119.8356745 | 0.058759686 | -4.089029511 | 3.83E-299 | 6.32E-297 | Down |
| NBEAL1 | 441.4833001 | 594.5843505 | 288.3822498 | 0.484909237 | -1.04421336 | 2.86E-14 | 9.29E-14 | Down |
| NBEAL2 | 2814.449847 | 3778.124244 | 1850.77545 | 0.489810642 | -1.029703974 | 2.40E-56 | 2.90E-55 | Down |
| NBPF15 | 574.8851896 | 806.5208904 | 343.2494888 | 0.425634751 | -1.232312149 | 4.03E-22 | 1.80E-21 | Down |
| NCCRP1 | 6.569532525 | 0 | 13.13906505 | 64.50121789 | 6.011254496 | 7.24E-05 | 0.000139063 | Up |
| NCF4 | 4978.70127 | 2651.71087 | 7305.691669 | 2.755212538 | 1.462163613 | 6.09E-165 | 3.77E-163 | Up |
| NCOA5 | 3470.541383 | 2064.825105 | 4876.25766 | 2.361665578 | 1.239804687 | 1.29E-75 | 2.37E-74 | Up |
| NCOA7 | 3398.404876 | 1666.777286 | 5130.032466 | 3.077454824 | 1.621737679 | 4.56E-83 | 9.85E-82 | Up |
| NCR3LG1 | 893.3654361 | 1401.251351 | 385.4795217 | 0.27515497 | -1.861683709 | 1.14E-75 | 2.09E-74 | Down |
| NDE1 | 4740.845721 | 6532.181071 | 2949.510371 | 0.451544041 | -1.14706139 | 1.04E-71 | 1.76E-70 | Down |
| NDRG2 | 120.6693361 | 169.4939816 | 71.84469059 | 0.424213931 | -1.237136095 | 8.70E-07 | 1.90E-06 | Down |
| NDRG3 | 1952.095953 | 2935.664417 | 968.5274885 | 0.329932719 | -1.599756239 | 5.49E-89 | 1.29E-87 | Down |
| NDST2 | 967.5130604 | 1346.937 | 588.0891207 | 0.43669546 | -1.195300562 | 3.15E-34 | 2.12E-33 | Down |
| NDUFA4L2 | 144.899939 | 230.4938957 | 59.30598218 | 0.257434917 | -1.957720351 | 1.85E-18 | 7.26E-18 | Down |
| NDUFAF4 | 706.3110684 | 440.3995033 | 972.2226335 | 2.206625571 | 1.141841848 | 1.00E-21 | 4.41E-21 | Up |
| NDUFAF8 | 502.6991243 | 303.5251763 | 701.8730722 | 2.312937097 | 1.209726031 | 4.49E-15 | 1.51E-14 | Up |
| NEB | 338.6627968 | 131.1649601 | 546.1606336 | 4.159324332 | 2.056349187 | 1.95E-27 | 1.07E-26 | Up |
| NECAB3 | 314.8762047 | 425.4403864 | 204.3120229 | 0.4801223 | -1.058526149 | 2.84E-11 | 8.02E-11 | Down |
| NECTIN2 | 1000.404365 | 621.564816 | 1379.243914 | 2.218345916 | 1.149484349 | 6.86E-30 | 4.07E-29 | Up |
| NEGR1 | 2.663344593 | 0 | 5.326689186 | 26.16749738 | 4.709704049 | 0.026498859 | 0.03860984 | Up |
| NEK10 | 41.72843285 | 23.30493292 | 60.15193278 | 2.5838488 | 1.36952165 | 0.001165108 | 0.001997678 | Up |
| NEK11 | 50.48073448 | 13.93695752 | 87.02451144 | 6.225099993 | 2.638097011 | 4.08E-11 | 1.14E-10 | Up |
| NEK3 | 153.0011869 | 224.0431207 | 81.95925317 | 0.365942392 | -1.450311544 | 6.84E-10 | 1.80E-09 | Down |
| NEK6 | 1540.225764 | 2219.772836 | 860.6786924 | 0.387704046 | -1.366972303 | 6.66E-69 | 1.05E-67 | Down |
| NEK8 | 654.3181738 | 430.8718799 | 877.7644678 | 2.036314393 | 1.025960321 | 1.36E-17 | 5.11E-17 | Up |
| NEU1 | 1764.118642 | 833.6773716 | 2694.559912 | 3.231711628 | 1.692298469 | 1.30E-112 | 4.44E-111 | Up |
| NEU3 | 2788.856005 | 4265.865659 | 1311.846352 | 0.307491768 | -1.701380305 | 4.05E-132 | 1.73E-130 | Down |
| NEU4 | 138.2857296 | 8.662598381 | 267.9088608 | 31.11536962 | 4.959555479 | 7.19E-12 | 2.10E-11 | Up |
| NEURL1B | 3155.704005 | 5155.169534 | 1156.238477 | 0.224281806 | -2.156615503 | 1.34E-197 | 1.14E-195 | Down |
| NEURL3 | 9.809379674 | 0 | 19.61875935 | 96.35324062 | 6.590261284 | 3.61E-06 | 7.55E-06 | Up |
| NEXN | 359.226529 | 611.3735457 | 107.0795124 | 0.175119575 | -2.51358774 | 3.33E-47 | 3.25E-46 | Down |
| NFAM1 | 25.88283235 | 1.078410468 | 50.68725424 | 45.95798126 | 5.522243523 | 9.32E-09 | 2.30E-08 | Up |
| NFATC4 | 3546.771584 | 4822.127389 | 2271.415778 | 0.471069747 | -1.085987411 | 2.20E-72 | 3.78E-71 | Down |
| NFIB | 4.284861269 | 8.269353044 | 0.300369495 | 0.038833565 | -4.686552027 | 0.004993386 | 0.008006388 | Down |
| NFKB1 | 9686.323421 | 3593.749233 | 15778.89761 | 4.39142128 | 2.134687942 | 1.55E-307 | 2.72E-305 | Up |
| NFKB2 | 10540.223 | 2461.935108 | 18618.5109 | 7.562800177 | 2.918920501 | 0 | 0 | Up |
| NFKBIB | 2414.210464 | 924.3701024 | 3904.050825 | 4.223407825 | 2.078407565 | 2.02E-161 | 1.20E-159 | Up |
| NFKBID | 595.2402099 | 394.3701569 | 796.1102628 | 2.017818117 | 1.012796138 | 1.91E-16 | 6.81E-16 | Up |
| NFKBIE | 1188.88533 | 518.8095225 | 1858.961138 | 3.580444312 | 1.840138629 | 2.72E-64 | 3.84E-63 | Up |
| NFKBIZ | 277.07353 | 83.00078571 | 471.1462742 | 5.676468843 | 2.504993752 | 2.08E-36 | 1.49E-35 | Up |
| NFU1 | 1480.65356 | 785.1834168 | 2176.123704 | 2.772420006 | 1.471145834 | 1.36E-56 | 1.65E-55 | Up |
| NGDN | 1959.549721 | 938.6632015 | 2980.436241 | 3.174819081 | 1.666674382 | 5.91E-100 | 1.62E-98 | Up |
| NHLH1 | 19.73461818 | 8.23071833 | 31.23851803 | 3.815790533 | 1.931981977 | 0.003768166 | 0.006125422 | Up |
| NHLRC3 | 973.3270915 | 1429.001157 | 517.6530261 | 0.362283899 | -1.464807406 | 1.00E-35 | 7.06E-35 | Down |
| NHSL1 | 3576.194869 | 5645.519872 | 1506.869867 | 0.266906214 | -1.905595203 | 1.50E-201 | 1.34E-199 | Down |
| NIBAN3 | 11321.09257 | 20911.61293 | 1730.57222 | 0.082758285 | -3.594952443 | 0 | 0 | Down |
| NICN1 | 705.4549556 | 961.8031031 | 449.1068082 | 0.467004204 | -1.098492557 | 2.32E-27 | 1.27E-26 | Down |
| NID1 | 40.5918419 | 0.385822986 | 80.79786082 | 203.6770844 | 7.670139863 | 5.70E-10 | 1.51E-09 | Up |
| NID2 | 36.58517015 | 51.49097964 | 21.67936067 | 0.421223977 | -1.247340535 | 0.009389731 | 0.014505508 | Down |
| NIFK | 2642.611252 | 1462.450846 | 3822.771659 | 2.614658218 | 1.386622373 | 1.17E-99 | 3.18E-98 | Up |
| NINJ2 | 138.3614361 | 44.70122084 | 232.0216513 | 5.211481303 | 2.3816935 | 5.50E-19 | 2.20E-18 | Up |
| NINL | 52.89017211 | 77.16996318 | 28.61038104 | 0.37082747 | -1.431179977 | 3.29E-05 | 6.45E-05 | Down |
| NIPAL1 | 12.80466718 | 4.785945699 | 20.82338866 | 4.348073581 | 2.120376355 | 0.008082296 | 0.01260791 | Up |
| NIPAL2 | 383.802784 | 584.1277463 | 183.4778217 | 0.314177592 | -1.670347809 | 5.39E-28 | 3.01E-27 | Down |
| NIPSNAP1 | 7239.844443 | 10812.55873 | 3667.130156 | 0.33915263 | -1.559993415 | 1.72E-133 | 7.49E-132 | Down |
| NKAIN1 | 4.351432656 | 0.365611098 | 8.337254215 | 21.01948537 | 4.393655442 | 0.008204733 | 0.012788837 | Up |
| NKAIN4 | 1471.009337 | 2430.365523 | 511.6531511 | 0.210535542 | -2.247864291 | 1.27E-115 | 4.45E-114 | Down |
| NKD2 | 70.70043631 | 110.6802703 | 30.72060228 | 0.277351833 | -1.850210836 | 4.39E-09 | 1.10E-08 | Down |
| NKIRAS1 | 347.1288713 | 215.0356472 | 479.2220955 | 2.230804144 | 1.157563856 | 1.13E-14 | 3.73E-14 | Up |
| NKPD1 | 107.0330804 | 44.59632524 | 169.4698356 | 3.798370706 | 1.925380714 | 5.27E-12 | 1.55E-11 | Up |
| NKTR | 6637.818359 | 9499.277336 | 3776.359382 | 0.397541061 | -1.330824214 | 2.88E-17 | 1.07E-16 | Down |
| NLGN2 | 2226.834598 | 2998.077476 | 1455.59172 | 0.485532925 | -1.042358963 | 1.59E-46 | 1.53E-45 | Down |
| NLRC4 | 8.422834729 | 2.256091312 | 14.58957815 | 6.53119781 | 2.707347604 | 0.00856416 | 0.013313175 | Up |
| NLRC5 | 2279.302971 | 3049.055035 | 1509.550906 | 0.495050176 | -1.014353338 | 4.73E-47 | 4.59E-46 | Down |
| NLRP1 | 2138.446023 | 1318.637137 | 2958.25491 | 2.243308787 | 1.165628218 | 3.18E-59 | 4.09E-58 | Up |
| NLRP3 | 117.1868177 | 48.23169212 | 186.1419432 | 3.857341962 | 1.94760705 | 1.53E-11 | 4.38E-11 | Up |
| NLRP4 | 7.30752708 | 0 | 14.61505416 | 71.75515833 | 6.165010643 | 2.89E-05 | 5.69E-05 | Up |
| NME3 | 277.8245088 | 437.1041442 | 118.5448733 | 0.2712693 | -1.882202308 | 3.04E-20 | 1.27E-19 | Down |
| NME4 | 3704.854805 | 5436.72763 | 1972.98198 | 0.362907223 | -1.462327323 | 3.86E-75 | 7.00E-74 | Down |
| NME7 | 456.1719048 | 277.8572094 | 634.4866002 | 2.282125578 | 1.190378181 | 8.41E-17 | 3.05E-16 | Up |
| NMU | 3.584443512 | 0 | 7.168887024 | 35.19248084 | 5.137195313 | 0.004313469 | 0.006961428 | Up |
| NOC4L | 1197.648554 | 647.2758194 | 1748.021289 | 2.702450692 | 1.434268295 | 1.34E-38 | 1.03E-37 | Up |
| NOD1 | 1072.713232 | 1550.036199 | 595.3902658 | 0.384162205 | -1.380212504 | 4.87E-54 | 5.63E-53 | Down |
| NOG | 17.77211068 | 8.754188361 | 26.790033 | 3.047679911 | 1.607711389 | 0.010779989 | 0.016537186 | Up |
| NOL10 | 1584.681423 | 970.6445626 | 2198.718284 | 2.265680746 | 1.179944588 | 3.29E-48 | 3.29E-47 | Up |
| NOL11 | 3510.560598 | 2098.264243 | 4922.856952 | 2.346117303 | 1.230275148 | 5.54E-78 | 1.07E-76 | Up |
| NOL4 | 14.86266624 | 24.3413886 | 5.383943885 | 0.221293105 | -2.175969592 | 0.011615502 | 0.017740434 | Down |
| NOL6 | 3358.935449 | 2204.817597 | 4513.053301 | 2.046971976 | 1.033491351 | 7.31E-45 | 6.69E-44 | Up |
| NOL7 | 2229.309639 | 1333.683427 | 3124.935852 | 2.343347092 | 1.228570659 | 4.49E-63 | 6.21E-62 | Up |
| NOP2 | 5243.542574 | 3453.964451 | 7033.120697 | 2.036497284 | 1.02608989 | 1.32E-58 | 1.69E-57 | Up |
| NOP58 | 4679.826681 | 2812.418718 | 6547.234644 | 2.327866303 | 1.219008202 | 1.05E-53 | 1.20E-52 | Up |
| NOS2 | 86.1999801 | 123.2653204 | 49.13463979 | 0.398547489 | -1.327176455 | 5.38E-06 | 1.11E-05 | Down |
| NOTCH3 | 100.9601323 | 10.12119854 | 191.7990661 | 19.14888453 | 4.259188449 | 1.57E-25 | 8.05E-25 | Up |
| NOXO1 | 14.07782596 | 7.031036537 | 21.12461539 | 3.008755146 | 1.589166704 | 0.024389176 | 0.035719803 | Up |
| NOXRED1 | 102.7991261 | 60.43297438 | 145.1652778 | 2.401171942 | 1.263738715 | 7.22E-07 | 1.58E-06 | Up |
| NPAS1 | 43.83900885 | 26.08807238 | 61.58994531 | 2.358649568 | 1.237961089 | 0.003233981 | 0.005290547 | Up |
| NPAS2 | 113.6199872 | 4.115359165 | 223.1246152 | 54.89020978 | 5.778476948 | 1.84E-31 | 1.15E-30 | Up |
| NPC2 | 1970.689298 | 2707.306274 | 1234.072322 | 0.455820705 | -1.133461637 | 9.01E-53 | 1.01E-51 | Down |
| NPIPB11 | 223.2733976 | 310.4672356 | 136.0795596 | 0.438722607 | -1.188619047 | 1.00E-06 | 2.18E-06 | Down |
| NPIPB6 | 201.0701419 | 113.6975232 | 288.4427606 | 2.539786153 | 1.344707029 | 1.85E-11 | 5.29E-11 | Up |
| NPW | 2.384778637 | 0 | 4.769557274 | 23.4182253 | 4.549559843 | 0.020354357 | 0.03013505 | Up |
| NPY | 369.012132 | 63.75647569 | 674.2677883 | 10.56945157 | 3.401828614 | 1.70E-91 | 4.15E-90 | Up |
| NQO2 | 305.3602111 | 422.5582266 | 188.1621956 | 0.445340895 | -1.167017996 | 6.01E-14 | 1.92E-13 | Down |
| NR0B2 | 2.845400552 | 0 | 5.690801104 | 27.93183041 | 4.803838213 | 0.019703342 | 0.029221112 | Up |
| NR1D1 | 591.8846938 | 368.7727951 | 814.9965925 | 2.210559646 | 1.144411662 | 1.00E-23 | 4.81E-23 | Up |
| NR1H3 | 602.574814 | 333.0908384 | 872.0587897 | 2.618069273 | 1.388503271 | 3.69E-30 | 2.21E-29 | Up |
| NR1H4 | 4.014005182 | 0 | 8.028010364 | 39.4260617 | 5.301077701 | 0.002423311 | 0.004030425 | Up |
| NR2F6 | 384.9652305 | 590.4490214 | 179.4814396 | 0.303998218 | -1.717865228 | 2.57E-34 | 1.74E-33 | Down |
| NR3C2 | 172.9853581 | 283.6473915 | 62.32332476 | 0.21972473 | -2.186230841 | 9.18E-26 | 4.75E-25 | Down |
| NR4A1 | 4433.056817 | 1003.052661 | 7863.060973 | 7.84084129 | 2.971008458 | 0 | 0 | Up |
| NR4A2 | 151.0519399 | 93.85652653 | 208.2473532 | 2.217378508 | 1.14885506 | 2.80E-06 | 5.88E-06 | Up |
| NR4A3 | 1660.493946 | 32.11386561 | 3288.874027 | 101.9216966 | 6.671317388 | 3.33E-118 | 1.21E-116 | Up |
| NRAP | 108.6038747 | 0 | 217.2077494 | 1066.530346 | 10.0587093 | 5.94E-17 | 2.17E-16 | Up |
| NRBP1 | 6031.018046 | 3632.972152 | 8429.06394 | 2.320172899 | 1.214232319 | 1.07E-95 | 2.77E-94 | Up |
| NRBP2 | 52.4020985 | 85.74171234 | 19.06248466 | 0.222312356 | -2.169339962 | 6.62E-08 | 1.55E-07 | Down |
| NRCAM | 27.35124237 | 39.54403882 | 15.15844592 | 0.38375044 | -1.381759691 | 0.007568266 | 0.011856722 | Down |
| NRG3 | 146.0785242 | 224.1933073 | 67.96374124 | 0.303036074 | -1.722438551 | 1.24E-15 | 4.28E-15 | Down |
| NRG4 | 10.64972186 | 4.30443046 | 16.99501327 | 3.907704505 | 1.966321377 | 0.031117219 | 0.044876996 | Up |
| NRGN | 1741.902671 | 2625.038394 | 858.7669488 | 0.327192884 | -1.611786721 | 1.15E-87 | 2.62E-86 | Down |
| NRIP3 | 4.453825699 | 0 | 8.907651397 | 43.75118608 | 5.451250223 | 0.001433508 | 0.002436191 | Up |
| NRM | 1878.00309 | 2695.379271 | 1060.626909 | 0.393508731 | -1.345532448 | 4.87E-53 | 5.47E-52 | Down |
| NRROS | 1194.549722 | 685.5011769 | 1703.598267 | 2.486199686 | 1.313942175 | 1.11E-44 | 1.01E-43 | Up |
| NRXN2 | 106.8832765 | 163.1199518 | 50.6466012 | 0.310682085 | -1.686489042 | 2.49E-11 | 7.05E-11 | Down |
| NRXN3 | 82.32905368 | 156.3148403 | 8.343267059 | 0.053381613 | -4.227513301 | 4.19E-29 | 2.42E-28 | Down |
| NSD2 | 15314.14885 | 21366.46026 | 9261.837436 | 0.433479672 | -1.205963754 | 7.24E-136 | 3.29E-134 | Down |
| NSF | 2533.347717 | 1668.291728 | 3398.403705 | 2.037031435 | 1.026468244 | 1.47E-48 | 1.48E-47 | Up |
| NSG1 | 2598.208663 | 4185.656627 | 1010.760699 | 0.241465727 | -2.050109662 | 6.32E-194 | 5.16E-192 | Down |
| NSRP1 | 1405.391813 | 804.6111971 | 2006.17243 | 2.49323751 | 1.318020323 | 7.51E-36 | 5.30E-35 | Up |
| NSUN5 | 2933.295941 | 1732.485526 | 4134.106356 | 2.38682883 | 1.255095108 | 1.44E-58 | 1.83E-57 | Up |
| NT5C | 539.9698714 | 261.0404294 | 818.8993134 | 3.134921967 | 1.648429533 | 5.91E-42 | 4.98E-41 | Up |
| NT5DC3 | 203.1925484 | 283.0397447 | 123.345352 | 0.435476111 | -1.199334516 | 2.43E-07 | 5.52E-07 | Down |
| NT5DC4 | 16.82425283 | 2.96531256 | 30.68319311 | 10.37458994 | 3.374982409 | 4.77E-05 | 9.27E-05 | Up |
| NT5E | 5.388174776 | 0.365611098 | 10.41073845 | 26.25018056 | 4.714255441 | 0.002496333 | 0.004143429 | Up |
| NT5M | 452.8971727 | 663.3179398 | 242.4764057 | 0.365551531 | -1.451853302 | 5.15E-24 | 2.49E-23 | Down |
| NTAN1 | 1055.380465 | 1426.950006 | 683.8109236 | 0.479260562 | -1.061117869 | 1.26E-30 | 7.67E-30 | Down |
| NTMT1 | 1440.603085 | 949.3009719 | 1931.905197 | 2.034913628 | 1.024967561 | 2.17E-34 | 1.47E-33 | Up |
| NTN3 | 2.414079268 | 4.828158535 | 0 | 0.034096384 | -4.874237444 | 0.013591755 | 0.020546123 | Down |
| NTN5 | 83.08405933 | 151.5639431 | 14.60417552 | 0.096388776 | -3.374991034 | 3.86E-24 | 1.88E-23 | Down |
| NUDT1 | 1312.875068 | 1756.990623 | 868.7595128 | 0.494512236 | -1.015921875 | 1.68E-30 | 1.02E-29 | Down |
| NUDT11 | 1121.720684 | 1506.835275 | 736.6060928 | 0.488811208 | -1.032650729 | 1.66E-34 | 1.13E-33 | Down |
| NUDT13 | 42.46537819 | 64.96153273 | 19.96922364 | 0.307165226 | -1.702913196 | 5.05E-05 | 9.79E-05 | Down |
| NUDT17 | 273.5047022 | 178.4889236 | 368.5204807 | 2.067703217 | 1.048029127 | 1.20E-08 | 2.95E-08 | Up |
| NUDT3 | 5008.619952 | 6833.469116 | 3183.770788 | 0.465893701 | -1.101927269 | 1.09E-68 | 1.71E-67 | Down |
| NUDT8 | 70.80843388 | 96.64455853 | 44.97230923 | 0.465758891 | -1.102344784 | 0.000330731 | 0.000598118 | Down |
| NUF2 | 2177.717671 | 3002.190661 | 1353.244681 | 0.450750074 | -1.149600367 | 1.58E-30 | 9.57E-30 | Down |
| NUFIP1 | 1211.863047 | 661.486191 | 1762.239902 | 2.663515028 | 1.413331421 | 1.04E-45 | 9.67E-45 | Up |
| NUGGC | 22.04287395 | 36.0271059 | 8.058641994 | 0.223166319 | -2.163808788 | 0.00042095 | 0.000753809 | Down |
| NUMBL | 248.0003947 | 340.9631019 | 155.0376875 | 0.454697217 | -1.137021922 | 1.22E-10 | 3.33E-10 | Down |
| NUP210 | 12242.12704 | 16800.01799 | 7684.236087 | 0.457399011 | -1.128474847 | 5.70E-93 | 1.43E-91 | Down |
| NUP35 | 686.0122406 | 415.957281 | 956.0672002 | 2.29939578 | 1.201254809 | 2.15E-28 | 1.22E-27 | Up |
| NUP88 | 7080.473559 | 4711.259637 | 9449.687481 | 2.006107391 | 1.004398838 | 8.69E-69 | 1.37E-67 | Up |
| NUPR2 | 5.16196663 | 0.771645971 | 9.552287288 | 12.70044617 | 3.666807275 | 0.023069155 | 0.033861613 | Up |
| NUTM2B | 13.62401923 | 2.197244711 | 25.05079375 | 11.34879832 | 3.504467639 | 6.63E-05 | 0.000127746 | Up |
| NUTM2E | 182.211677 | 120.7515756 | 243.6717783 | 2.015225051 | 1.010940961 | 3.03E-06 | 6.37E-06 | Up |
| NVL | 2739.25551 | 1560.073586 | 3918.437435 | 2.511821078 | 1.328733702 | 5.61E-78 | 1.08E-76 | Up |
| NXF1 | 5492.973101 | 3207.598881 | 7778.347321 | 2.424777458 | 1.277852345 | 4.47E-123 | 1.73E-121 | Up |
| NXF5 | 5.04812323 | 0 | 10.09624646 | 49.59012056 | 5.631980828 | 0.000517432 | 0.000917344 | Up |
| NXPE3 | 1461.906173 | 2054.025889 | 869.7864577 | 0.423389585 | -1.239942315 | 4.51E-52 | 4.95E-51 | Down |
| NXPH4 | 78.09197583 | 115.6739601 | 40.50999152 | 0.350399398 | -1.512927799 | 2.32E-07 | 5.27E-07 | Down |
| NXT2 | 562.7366396 | 750.9496001 | 374.5236791 | 0.498748713 | -1.003614976 | 3.63E-14 | 1.17E-13 | Down |
| NYNRIN | 3490.493104 | 5508.794747 | 1472.191461 | 0.267251641 | -1.90372929 | 6.41E-137 | 2.93E-135 | Down |
| OAF | 487.9514986 | 671.0049106 | 304.8980866 | 0.454483713 | -1.137699501 | 2.26E-20 | 9.48E-20 | Down |
| OAS2 | 1643.803324 | 2436.740486 | 850.866162 | 0.349196267 | -1.517889958 | 8.22E-66 | 1.21E-64 | Down |
| OAZ3 | 22.98492684 | 6.951978048 | 39.01787563 | 5.590153659 | 2.48288794 | 3.04E-05 | 5.98E-05 | Up |
| OBSCN | 772.1655538 | 1156.55052 | 387.7805879 | 0.335165735 | -1.57705343 | 8.02E-43 | 6.93E-42 | Down |
| OCEL1 | 669.6435872 | 993.7847222 | 345.5024522 | 0.347755081 | -1.5238565 | 4.77E-41 | 3.91E-40 | Down |
| OCIAD1 | 3130.971711 | 1677.336014 | 4584.607408 | 2.732600908 | 1.450274772 | 5.23E-102 | 1.49E-100 | Up |
| OCSTAMP | 3.414650682 | 0 | 6.829301365 | 33.54287418 | 5.067934409 | 0.006097499 | 0.009677163 | Up |
| ODAD1 | 78.85004394 | 119.5066029 | 38.19348501 | 0.319771323 | -1.644887532 | 2.59E-07 | 5.88E-07 | Down |
| ODAD2 | 11.41880962 | 21.9417589 | 0.895860343 | 0.0408155 | -4.61473906 | 2.84E-05 | 5.61E-05 | Down |
| ODAD3 | 32.05259231 | 17.08238801 | 47.02279661 | 2.759833391 | 1.464581175 | 0.001993838 | 0.003346628 | Up |
| ODAD4 | 7.684273572 | 2.524221096 | 12.84432605 | 5.038379227 | 2.332959714 | 0.030550583 | 0.04412862 | Up |
| ODF3L1 | 7.10205886 | 1.078410468 | 13.12570725 | 11.96165457 | 3.580345056 | 0.003005708 | 0.004939247 | Up |
| OFD1 | 2481.678052 | 3326.151408 | 1637.204695 | 0.492254898 | -1.022522534 | 9.30E-24 | 4.46E-23 | Down |
| OGFOD1 | 4191.55478 | 2708.707428 | 5674.402132 | 2.094822477 | 1.06682799 | 9.42E-68 | 1.45E-66 | Up |
| OGFOD3 | 767.8735359 | 1101.716152 | 434.0309201 | 0.393959015 | -1.343882544 | 2.46E-32 | 1.57E-31 | Down |
| OGG1 | 335.6255587 | 520.2932271 | 150.9578904 | 0.289992277 | -1.785913613 | 1.62E-29 | 9.51E-29 | Down |
| OGN | 3.675295285 | 7.35059057 | 0 | 0.02236716 | -5.482474136 | 0.001547533 | 0.002620661 | Down |
| OGT | 13911.80546 | 19319.59858 | 8504.012338 | 0.440179877 | -1.183834901 | 7.80E-81 | 1.61E-79 | Down |
| OIT3 | 20.21420068 | 2.204667063 | 38.22373429 | 17.28487258 | 4.111438064 | 3.34E-07 | 7.51E-07 | Up |
| OLIG3 | 3.851511041 | 0 | 7.703022082 | 37.83969303 | 5.24182848 | 0.004238664 | 0.006846921 | Up |
| OPN1SW | 2.68190427 | 0 | 5.363808541 | 26.33698359 | 4.719018216 | 0.012047959 | 0.018360496 | Up |
| OPTN | 4529.418525 | 2868.992856 | 6189.844194 | 2.157281434 | 1.109214399 | 5.73E-88 | 1.32E-86 | Up |
| OR13A1 | 126.7971041 | 190.0549006 | 63.53930759 | 0.334153731 | -1.58141611 | 3.11E-11 | 8.76E-11 | Down |
| OR1L8 | 5.800477724 | 0 | 11.60095545 | 56.96997093 | 5.832129765 | 0.000353064 | 0.000636267 | Up |
| OR2B6 | 8.729278958 | 2.285514613 | 15.1730433 | 6.744108896 | 2.753627832 | 0.00913058 | 0.014128732 | Up |
| OR2L13 | 3.32458968 | 0.356399685 | 6.292779675 | 15.85529255 | 3.986892593 | 0.029099469 | 0.042168693 | Up |
| ORAI2 | 8704.129121 | 14464.63626 | 2943.621986 | 0.203504852 | -2.2968649 | 5.06E-275 | 7.38E-273 | Down |
| ORC5 | 1234.391933 | 744.7536573 | 1724.030208 | 2.314340742 | 1.210601289 | 1.32E-44 | 1.20E-43 | Up |
| OSBP2 | 11.26275575 | 19.26505412 | 3.260457371 | 0.169732978 | -2.558661195 | 0.003273505 | 0.005351091 | Down |
| OSBPL7 | 539.8438034 | 722.9883967 | 356.6992101 | 0.493181833 | -1.019808439 | 1.10E-15 | 3.82E-15 | Down |
| OSCP1 | 62.75426571 | 21.73937392 | 103.7691575 | 4.771472839 | 2.25443466 | 1.58E-08 | 3.84E-08 | Up |
| OSGEPL1 | 283.6551435 | 444.4718674 | 122.8384197 | 0.276222588 | -1.856096793 | 1.37E-26 | 7.30E-26 | Down |
| OSM | 145.3125923 | 75.71494114 | 214.9102434 | 2.83972893 | 1.505753222 | 3.79E-12 | 1.12E-11 | Up |
| OTOP2 | 5.098706565 | 0.356399685 | 9.841013445 | 24.80686607 | 4.632667582 | 0.003529116 | 0.005751659 | Up |
| OTUD7A | 199.0634368 | 281.1932675 | 116.933606 | 0.416015059 | -1.265292342 | 1.98E-09 | 5.07E-09 | Down |
| OTUD7B | 182.1386577 | 94.54095896 | 269.7363565 | 2.854155034 | 1.513063702 | 5.71E-13 | 1.75E-12 | Up |
| OTX1 | 380.3585723 | 527.4278863 | 233.2892584 | 0.442408114 | -1.176550249 | 3.06E-18 | 1.19E-17 | Down |
| OVCA2 | 55.08444068 | 32.93567092 | 77.23321045 | 2.347353342 | 1.231035024 | 0.000447601 | 0.000798851 | Up |
| OVGP1 | 557.0822895 | 357.5261141 | 756.6384649 | 2.118216857 | 1.082850296 | 1.67E-18 | 6.55E-18 | Up |
| OVOL3 | 12.12689306 | 2.184455176 | 22.06933095 | 10.02222642 | 3.325131132 | 0.000336086 | 0.000607169 | Up |
| P2RX5 | 66.3226157 | 88.78250527 | 43.86272614 | 0.493839342 | -1.01788632 | 0.003806748 | 0.006185241 | Down |
| P2RX7 | 572.4276021 | 912.674092 | 232.1811122 | 0.254347149 | -1.975129172 | 5.74E-50 | 5.94E-49 | Down |
| P2RY10 | 83.84045538 | 23.01122406 | 144.6696867 | 6.303816541 | 2.656225549 | 1.14E-14 | 3.76E-14 | Up |
| P2RY8-2 | 82.18823829 | 150.6451645 | 13.73131204 | 0.091138728 | -3.455791948 | 1.44E-20 | 6.07E-20 | Down |
| P3H4 | 291.7684492 | 421.6653335 | 161.8715649 | 0.383769985 | -1.381686213 | 5.05E-18 | 1.94E-17 | Down |
| P4HA1 | 2580.075314 | 1406.712627 | 3753.438001 | 2.667523463 | 1.415500961 | 9.55E-78 | 1.83E-76 | Up |
| PABIR2 | 1833.270717 | 2523.361835 | 1143.1796 | 0.453073454 | -1.142183129 | 4.46E-52 | 4.89E-51 | Down |
| PABPC1 | 45501.46461 | 62364.03429 | 28638.89492 | 0.459225397 | -1.122725663 | 3.88E-125 | 1.55E-123 | Down |
| PABPC1L | 940.8068539 | 1307.202665 | 574.4110425 | 0.439440771 | -1.186259367 | 4.77E-34 | 3.18E-33 | Down |
| PAFAH1B3 | 1228.643682 | 1684.872414 | 772.4149502 | 0.45849836 | -1.12501152 | 3.87E-39 | 3.01E-38 | Down |
| PALD1 | 3053.09796 | 5191.807438 | 914.3884815 | 0.176128526 | -2.505299507 | 4.95E-186 | 3.71E-184 | Down |
| PALM | 84.82060013 | 120.5041738 | 49.13702642 | 0.408004715 | -1.293342272 | 7.66E-06 | 1.57E-05 | Down |
| PAM | 3095.491371 | 1118.109439 | 5072.873303 | 4.534933984 | 2.181081549 | 1.76E-199 | 1.52E-197 | Up |
| PANO1 | 45.51745258 | 24.58341516 | 66.45149 | 2.698099713 | 1.431943667 | 0.000262846 | 0.000480677 | Up |
| PAOX | 136.1916894 | 185.5565558 | 86.82682295 | 0.46794403 | -1.095592113 | 8.33E-05 | 0.000159179 | Down |
| PAPPA2 | 4.559277614 | 0.751434084 | 8.367121144 | 11.26206612 | 3.49339962 | 0.020971896 | 0.03098319 | Up |
| PAQR4 | 2337.969886 | 3856.831892 | 819.1078805 | 0.212385844 | -2.235240484 | 2.90E-243 | 3.47E-241 | Down |
| PARD6B | 40.55875142 | 65.27596956 | 15.84153328 | 0.242635966 | -2.043134677 | 6.01E-06 | 1.24E-05 | Down |
| PARD6G | 121.6099476 | 170.4802941 | 72.73960118 | 0.426672608 | -1.228798601 | 3.67E-06 | 7.66E-06 | Down |
| PARL | 2734.993934 | 1763.620692 | 3706.367175 | 2.101614532 | 1.071498081 | 1.12E-54 | 1.31E-53 | Up |
| PARP11 | 642.8184342 | 862.5664982 | 423.0703702 | 0.490320167 | -1.028203993 | 2.98E-18 | 1.16E-17 | Down |
| PARP16 | 1004.114443 | 1422.816257 | 585.4126298 | 0.411552571 | -1.280851366 | 3.26E-40 | 2.62E-39 | Down |
| PASK | 1482.336598 | 2139.649635 | 825.0235606 | 0.385610472 | -1.374783865 | 1.57E-67 | 2.41E-66 | Down |
| PATJ | 840.3832617 | 1326.783429 | 353.9830942 | 0.266812238 | -1.906103253 | 2.74E-73 | 4.77E-72 | Down |
| PCBP4 | 2984.48663 | 4353.315315 | 1615.657945 | 0.371128483 | -1.430009368 | 1.29E-98 | 3.45E-97 | Down |
| PCDH12 | 10.86288503 | 19.04066819 | 2.685101868 | 0.140849415 | -2.827774527 | 0.003266356 | 0.005340991 | Down |
| PCDH18 | 23.8473409 | 33.9564997 | 13.73818211 | 0.403762022 | -1.308422877 | 0.022181449 | 0.032644973 | Down |
| PCDHGA4 | 13.62498764 | 20.9810497 | 6.26892559 | 0.298547987 | -1.743965255 | 0.022442421 | 0.03300454 | Down |
| PCDHGA5 | 18.88754956 | 29.42667644 | 8.348422674 | 0.283959004 | -1.816245438 | 0.016920608 | 0.02531066 | Down |
| PCED1A | 787.0301283 | 1138.229808 | 435.8304484 | 0.382864443 | -1.385094412 | 8.52E-44 | 7.56E-43 | Down |
| PCED1B | 197.0606133 | 121.5961641 | 272.5250626 | 2.244004149 | 1.166075344 | 1.74E-07 | 3.98E-07 | Up |
| PCF11 | 4918.856149 | 3009.301955 | 6828.410343 | 2.269139813 | 1.182145504 | 1.13E-39 | 8.86E-39 | Up |
| PCK2 | 2782.088817 | 4346.241689 | 1217.935945 | 0.280217151 | -1.835382837 | 8.20E-170 | 5.36E-168 | Down |
| PCLAF | 2242.729292 | 3269.186913 | 1216.27167 | 0.372020265 | -1.426546883 | 1.97E-93 | 4.99E-92 | Down |
| PCMTD2 | 1638.882971 | 2238.583094 | 1039.182849 | 0.464250167 | -1.107025664 | 1.41E-35 | 9.90E-35 | Down |
| PCNX2 | 208.9000208 | 6.525731288 | 411.2743102 | 62.73626976 | 5.971227845 | 2.76E-11 | 7.80E-11 | Up |
| PCOLCE | 432.2392299 | 605.4386017 | 259.0398581 | 0.427930515 | -1.224551537 | 2.71E-13 | 8.43E-13 | Down |
| PCOTH | 22.11758883 | 9.636105183 | 34.59907248 | 3.597256419 | 1.846897001 | 0.001442875 | 0.002450909 | Up |
| PCSK4 | 135.4857657 | 203.9184329 | 67.05309846 | 0.328758837 | -1.604898421 | 7.29E-09 | 1.81E-08 | Down |
| PCSK6 | 339.0167052 | 468.4924209 | 209.5409894 | 0.44726693 | -1.160792004 | 6.99E-16 | 2.44E-15 | Down |
| PCYOX1L | 333.6050063 | 477.5449273 | 189.6650853 | 0.396933066 | -1.333032347 | 1.47E-14 | 4.84E-14 | Down |
| PCYT2 | 2431.295474 | 3594.482632 | 1268.108315 | 0.352811276 | -1.503031426 | 1.01E-108 | 3.26E-107 | Down |
| PDCD1 | 93.26211192 | 11.91061932 | 174.6136045 | 14.74798868 | 3.882446309 | 9.38E-26 | 4.85E-25 | Up |
| PDE2A | 70.23053334 | 34.38684873 | 106.074218 | 3.087434707 | 1.626408627 | 1.74E-06 | 3.73E-06 | Up |
| PDE3B | 2132.352482 | 1111.066179 | 3153.638784 | 2.838244448 | 1.504998849 | 5.26E-72 | 8.91E-71 | Up |
| PDE4B | 1737.300998 | 465.1504554 | 3009.45154 | 6.46257401 | 2.692108898 | 2.14E-200 | 1.88E-198 | Up |
| PDE4C | 10.60164567 | 0.385822986 | 20.81746835 | 52.48993641 | 5.713968945 | 4.84E-05 | 9.40E-05 | Up |
| PDE4D | 1431.27003 | 520.784994 | 2341.755066 | 4.497357161 | 2.169077461 | 6.16E-112 | 2.07E-110 | Up |
| PDE6B | 54.95088119 | 27.64595099 | 82.2558114 | 2.962794977 | 1.566958796 | 0.000117634 | 0.000222273 | Up |
| PDE6G | 77.3518117 | 104.3441264 | 50.35949697 | 0.482942906 | -1.050075451 | 0.00030687 | 0.000557146 | Down |
| PDE7A | 2367.802859 | 3323.065031 | 1412.540687 | 0.425055445 | -1.234277053 | 3.56E-72 | 6.07E-71 | Down |
| PDE9A | 102.6814117 | 29.72960477 | 175.6332187 | 5.892915687 | 2.558981625 | 1.79E-18 | 7.02E-18 | Up |
| PDGFA | 134.060991 | 69.34448946 | 198.7774926 | 2.862289395 | 1.517169545 | 1.15E-08 | 2.82E-08 | Up |
| PDGFRA | 5912.110613 | 9306.219057 | 2518.002168 | 0.270573147 | -1.88590943 | 1.54E-168 | 9.97E-167 | Down |
| PDIA4 | 4077.468082 | 5799.892876 | 2355.043288 | 0.406077404 | -1.300173342 | 1.20E-91 | 2.96E-90 | Down |
| PDIA6 | 15521.94603 | 21596.1778 | 9447.714253 | 0.437473902 | -1.192731141 | 1.63E-106 | 5.07E-105 | Down |
| PDK3 | 1511.929999 | 2060.190386 | 963.6696116 | 0.46779809 | -1.096042122 | 2.40E-38 | 1.83E-37 | Down |
| PDLIM1 | 18542.67408 | 27017.35276 | 10067.99541 | 0.372651349 | -1.424101614 | 4.04E-149 | 2.08E-147 | Down |
| PDLIM2 | 118.0421177 | 20.06612339 | 216.018112 | 10.81373667 | 3.434793225 | 5.05E-30 | 3.02E-29 | Up |
| PDLIM7 | 2351.211587 | 3205.840374 | 1496.5828 | 0.466828864 | -1.099034331 | 1.33E-62 | 1.82E-61 | Down |
| PDP2 | 2030.227183 | 2991.173059 | 1069.281308 | 0.357418637 | -1.484313231 | 1.01E-81 | 2.11E-80 | Down |
| PDPR | 9225.891887 | 12532.21803 | 5919.565744 | 0.472344825 | -1.082087643 | 1.03E-102 | 2.97E-101 | Down |
| PDRG1 | 2887.083759 | 1598.448414 | 4175.719104 | 2.612642974 | 1.385509989 | 2.54E-74 | 4.50E-73 | Up |
| PDZD7 | 828.567706 | 1461.977808 | 195.157604 | 0.133496182 | -2.905129611 | 2.26E-148 | 1.15E-146 | Down |
| PDZK1 | 14.06369084 | 4.905427962 | 23.22195371 | 4.781793583 | 2.257551854 | 0.003343567 | 0.005461448 | Up |
| PDZRN4 | 63.09500504 | 5.485955533 | 120.7040545 | 21.86517885 | 4.450563244 | 1.42E-18 | 5.57E-18 | Up |
| PEA15 | 3245.679283 | 1736.397426 | 4754.961139 | 2.738388642 | 1.453327214 | 4.60E-110 | 1.51E-108 | Up |
| PEAR1 | 18.75184989 | 28.53707922 | 8.966620555 | 0.313536112 | -1.673296476 | 0.007754426 | 0.012126479 | Down |
| PECR | 156.938529 | 221.984804 | 91.89225404 | 0.413631383 | -1.273582447 | 1.45E-08 | 3.53E-08 | Down |
| PELI3 | 714.0230878 | 1124.36452 | 303.6816554 | 0.270074843 | -1.888568832 | 4.27E-46 | 4.03E-45 | Down |
| PER1 | 3073.551697 | 1661.884182 | 4485.219212 | 2.699466673 | 1.432674406 | 4.83E-96 | 1.26E-94 | Up |
| PER2 | 3486.537585 | 2033.140683 | 4939.934486 | 2.429658465 | 1.28075353 | 1.68E-80 | 3.42E-79 | Up |
| PEX11G | 2.245985369 | 4.491970737 | 0 | 0.036679757 | -4.76887211 | 0.020571106 | 0.030442955 | Down |
| PFKFB3 | 1572.288964 | 774.002787 | 2370.575141 | 3.062800082 | 1.614851201 | 7.84E-80 | 1.58E-78 | Up |
| PFKL | 8002.408274 | 10911.96339 | 5092.853162 | 0.466726581 | -1.099350462 | 1.12E-101 | 3.18E-100 | Down |
| PFKM | 3962.170915 | 5303.945752 | 2620.396077 | 0.494059081 | -1.01724452 | 2.00E-59 | 2.59E-58 | Down |
| PGAM2 | 39.75006347 | 55.07955676 | 24.42057018 | 0.443647839 | -1.172513153 | 0.003736862 | 0.006077384 | Down |
| PGAP3 | 252.9967708 | 341.1897515 | 164.8037901 | 0.482952499 | -1.050046795 | 2.64E-10 | 7.11E-10 | Down |
| PGBD5 | 3574.140366 | 4804.883224 | 2343.397508 | 0.48774009 | -1.035815535 | 5.59E-42 | 4.72E-41 | Down |
| PGC | 16.4411898 | 2.13839811 | 30.74398149 | 14.07051634 | 3.814603366 | 2.08E-05 | 4.13E-05 | Up |
| PGGHG | 1901.459909 | 3082.868779 | 720.0510398 | 0.233540827 | -2.098253317 | 4.83E-170 | 3.19E-168 | Down |
| PHACTR3 | 1962.744499 | 3636.431785 | 289.0572132 | 0.079497279 | -3.652950708 | 0 | 0 | Down |
| PHAX | 1822.87866 | 1185.792511 | 2459.96481 | 2.074337507 | 1.052650648 | 2.04E-43 | 1.79E-42 | Up |
| PHC1 | 1232.370657 | 1755.660626 | 709.0806883 | 0.403879101 | -1.308004599 | 7.04E-55 | 8.30E-54 | Down |
| PHEX | 23.74819634 | 33.79943102 | 13.69696166 | 0.405759773 | -1.301302251 | 0.02289468 | 0.033626864 | Down |
| PHF7 | 604.6182295 | 922.2626344 | 286.9738246 | 0.311208984 | -1.684044385 | 2.45E-38 | 1.87E-37 | Down |
| PHGDH | 11294.09982 | 20955.25759 | 1632.942053 | 0.077924501 | -3.681779183 | 0 | 0 | Down |
| PHKA2 | 2099.7937 | 3051.92017 | 1147.667231 | 0.376076653 | -1.410901349 | 6.11E-68 | 9.48E-67 | Down |
| PHLDA1 | 1001.211778 | 129.7567773 | 1872.666778 | 14.44881205 | 3.852878977 | 3.56E-195 | 2.94E-193 | Up |
| PHLDA3 | 368.2475376 | 17.30267166 | 719.1924036 | 41.4951546 | 5.374870977 | 3.30E-103 | 9.69E-102 | Up |
| PHLDB1 | 377.0876072 | 565.2080914 | 188.967123 | 0.33434262 | -1.580600823 | 8.03E-29 | 4.61E-28 | Down |
| PHYH | 1789.626782 | 2451.818237 | 1127.435327 | 0.459803418 | -1.120910903 | 4.62E-55 | 5.46E-54 | Down |
| PI16 | 8.098489244 | 2.177032824 | 14.01994566 | 6.382957098 | 2.674224951 | 0.007803581 | 0.012200558 | Up |
| PI4K2B | 1746.010345 | 2477.463629 | 1014.557062 | 0.409483109 | -1.288124152 | 3.02E-45 | 2.79E-44 | Down |
| PICK1 | 110.3962744 | 211.8275501 | 8.964998624 | 0.042231782 | -4.565527059 | 5.84E-36 | 4.14E-35 | Down |
| PID1 | 6.581030953 | 0 | 13.16206191 | 64.60488029 | 6.013571246 | 9.69E-05 | 0.000184191 | Up |
| PIDD1 | 1315.276831 | 1768.598464 | 861.9551983 | 0.487306954 | -1.037097286 | 7.94E-32 | 5.01E-31 | Down |
| PIEZO2 | 2.826221085 | 0 | 5.652442169 | 27.75818512 | 4.79484134 | 0.013292724 | 0.020140182 | Up |
| PIF1 | 1146.488124 | 1697.377574 | 595.5986744 | 0.350889072 | -1.510913079 | 6.82E-63 | 9.39E-62 | Down |
| PIH1D2 | 62.23839788 | 39.32912234 | 85.14767341 | 2.169282285 | 1.117217801 | 0.000992347 | 0.001714392 | Up |
| PIK3C3 | 1886.353679 | 1168.432573 | 2604.274786 | 2.228949028 | 1.156363625 | 1.66E-52 | 1.85E-51 | Up |
| PIK3CD | 16378.03966 | 24499.36098 | 8256.718338 | 0.337020713 | -1.569090833 | 5.74E-186 | 4.28E-184 | Down |
| PIK3IP1 | 212.7244534 | 114.1237781 | 311.3251287 | 2.728819553 | 1.448276998 | 1.53E-13 | 4.81E-13 | Up |
| PIK3R6 | 71.88380646 | 23.38014718 | 120.3874657 | 5.161878026 | 2.367896051 | 2.60E-10 | 7.02E-10 | Up |
| PILRA | 150.6059211 | 49.01665984 | 252.1951825 | 5.142794892 | 2.362552617 | 1.26E-23 | 5.99E-23 | Up |
| PIMREG | 1693.86161 | 2558.348174 | 829.375046 | 0.324191217 | -1.625083089 | 2.11E-89 | 4.97E-88 | Down |
| PINLYP | 59.42105186 | 0 | 118.8421037 | 583.5587362 | 9.188734063 | 4.84E-14 | 1.55E-13 | Up |
| PIP5KL1 | 96.56969164 | 149.9454369 | 43.19394634 | 0.28818175 | -1.794949121 | 1.62E-11 | 4.63E-11 | Down |
| PIPOX | 124.6481673 | 64.21188771 | 185.0844469 | 2.87708994 | 1.524610321 | 6.59E-10 | 1.74E-09 | Up |
| PITPNM2 | 5386.595645 | 8736.165718 | 2037.025572 | 0.233164229 | -2.100581618 | 1.13E-199 | 9.80E-198 | Down |
| PIWIL4 | 21.60122096 | 3.207597165 | 39.99484476 | 12.39704184 | 3.631924003 | 0.02598915 | 0.037914967 | Up |
| PJVK | 78.908351 | 139.3578178 | 18.45888416 | 0.132615308 | -2.914680778 | 1.71E-18 | 6.71E-18 | Down |
| PKIG | 3345.181551 | 1543.478997 | 5146.884105 | 3.334736272 | 1.73757267 | 9.44E-128 | 3.86E-126 | Up |
| PKMYT1 | 3761.78431 | 5317.065722 | 2206.502897 | 0.414984343 | -1.26887119 | 1.89E-82 | 4.02E-81 | Down |
| PKN3 | 8661.902771 | 12903.37394 | 4420.431604 | 0.342574188 | -1.545511644 | 1.29E-185 | 9.51E-184 | Down |
| PLA2G12B | 8.490445385 | 0 | 16.98089077 | 83.38063944 | 6.381640531 | 1.02E-05 | 2.07E-05 | Up |
| PLA2G1B | 8.418173683 | 14.14113156 | 2.695215804 | 0.190464837 | -2.392403418 | 0.017917124 | 0.026706256 | Down |
| PLA2G2D | 25.62054925 | 49.75299117 | 1.488107328 | 0.029914449 | -5.063013702 | 4.20E-10 | 1.12E-09 | Down |
| PLA2G4C | 494.5478551 | 11.56522011 | 977.5304902 | 85.37817039 | 6.415795342 | 3.17E-108 | 1.01E-106 | Up |
| PLAAT4 | 204.2316468 | 301.1230905 | 107.3402031 | 0.356533435 | -1.487890719 | 1.06E-09 | 2.75E-09 | Down |
| PLAC1 | 8.199242027 | 14.62775594 | 1.770728112 | 0.12146824 | -3.041348951 | 0.007395236 | 0.011597435 | Down |
| PLAC8 | 132.7433141 | 177.0400337 | 88.44659448 | 0.499419765 | -1.001675177 | 0.000502213 | 0.000891616 | Down |
| PLAUR | 701.1892192 | 1078.921675 | 323.4567637 | 0.299888977 | -1.737499601 | 1.90E-45 | 1.76E-44 | Down |
| PLB1 | 82.86541961 | 23.37452195 | 142.3563173 | 6.062110288 | 2.599820099 | 5.78E-15 | 1.93E-14 | Up |
| PLCB3 | 1522.76779 | 2384.760576 | 660.7750028 | 0.277090794 | -1.851569313 | 4.47E-126 | 1.81E-124 | Down |
| PLCD1 | 243.8304213 | 369.8081944 | 117.8526483 | 0.318622147 | -1.650081547 | 1.28E-18 | 5.03E-18 | Down |
| PLCG1 | 5597.116229 | 7943.052941 | 3251.179516 | 0.409330989 | -1.288660202 | 2.34E-123 | 9.07E-122 | Down |
| PLCH2 | 41.95783076 | 73.17793051 | 10.73773102 | 0.146725328 | -2.76881016 | 2.23E-10 | 6.03E-10 | Down |
| PLD1 | 51.23878403 | 1.484445341 | 100.9931227 | 68.4133457 | 6.096205881 | 2.17E-14 | 7.07E-14 | Up |
| PLD2 | 467.7626063 | 629.8628516 | 305.662361 | 0.485367149 | -1.042851628 | 9.34E-17 | 3.37E-16 | Down |
| PLD4 | 5124.808985 | 9924.827826 | 324.790144 | 0.03271689 | -4.933820592 | 0 | 0 | Down |
| PLEK | 1041.43888 | 21.78722005 | 2061.09054 | 94.69481658 | 6.565213552 | 4.53E-219 | 4.76E-217 | Up |
| PLEKHA1 | 267.6659169 | 151.5782718 | 383.753562 | 2.529490004 | 1.338846538 | 4.72E-15 | 1.58E-14 | Up |
| PLEKHA4 | 43.86845104 | 28.99864062 | 58.73826146 | 2.030255604 | 1.02166137 | 0.010673809 | 0.016385171 | Up |
| PLEKHA5 | 8.665378719 | 0.365611098 | 16.96514634 | 42.77369299 | 5.418651867 | 0.000157165 | 0.000293409 | Up |
| PLEKHA6 | 51.28601752 | 16.17641507 | 86.39561998 | 5.333006863 | 2.414949185 | 2.10E-09 | 5.38E-09 | Up |
| PLEKHA7 | 63.61107814 | 10.33817018 | 116.8839861 | 11.30633547 | 3.499059504 | 0.007056894 | 0.011103241 | Up |
| PLEKHB1 | 289.264997 | 536.829878 | 41.70011599 | 0.077698972 | -3.685960682 | 8.50E-81 | 1.74E-79 | Down |
| PLEKHG4B | 1174.650631 | 1854.979294 | 494.3219689 | 0.266475326 | -1.907926141 | 6.33E-99 | 1.69E-97 | Down |
| PLEKHG5 | 56.73404556 | 88.47779594 | 24.99029519 | 0.282598065 | -1.823176508 | 5.68E-07 | 1.26E-06 | Down |
| PLEKHH3 | 167.9955226 | 277.0500322 | 58.94101304 | 0.212851418 | -2.232081391 | 1.41E-24 | 6.96E-24 | Down |
| PLEKHM2 | 5922.241029 | 3277.949348 | 8566.532709 | 2.613493375 | 1.385979502 | 1.44E-118 | 5.23E-117 | Up |
| PLEKHS1 | 4.048439089 | 0.365611098 | 7.731267081 | 19.49446491 | 4.284992651 | 0.01115136 | 0.017082332 | Up |
| PLG | 20.78232619 | 6.360450003 | 35.20420238 | 5.571174146 | 2.477981413 | 0.000128712 | 0.000242215 | Up |
| PLGLB1 | 104.4154953 | 57.47713128 | 151.3538593 | 2.638071161 | 1.399483481 | 1.58E-07 | 3.64E-07 | Up |
| PLIN4 | 18.47839862 | 30.73100385 | 6.225793391 | 0.203070252 | -2.299949178 | 0.000949579 | 0.001643369 | Down |
| PLK3 | 729.488055 | 447.2025516 | 1011.773558 | 2.262568159 | 1.177961253 | 3.48E-23 | 1.63E-22 | Up |
| PLPP5 | 872.820873 | 1178.694607 | 566.9471389 | 0.480992776 | -1.055912869 | 1.20E-22 | 5.45E-22 | Down |
| PLPPR2 | 42.76636554 | 67.33299597 | 18.19973511 | 0.270127906 | -1.888285409 | 2.44E-05 | 4.83E-05 | Down |
| PLSCR1 | 954.8484704 | 562.1371183 | 1347.559823 | 2.398750486 | 1.262283099 | 5.75E-36 | 4.08E-35 | Up |
| PLSCR3 | 1102.622122 | 1525.526475 | 679.7177687 | 0.445583097 | -1.166233589 | 4.33E-40 | 3.47E-39 | Down |
| PLTP | 496.1055572 | 688.6381529 | 303.5729615 | 0.440773596 | -1.181890292 | 1.13E-21 | 5.00E-21 | Down |
| PLXNB1 | 1856.844638 | 2898.633476 | 815.0557997 | 0.281165264 | -1.830509725 | 3.93E-99 | 1.05E-97 | Down |
| PLXNB2 | 815.7312132 | 122.5819363 | 1508.88049 | 12.31620062 | 3.622485368 | 6.18E-197 | 5.20E-195 | Up |
| PLXNB3 | 5.765804571 | 0.771645971 | 10.75996317 | 14.36284182 | 3.844269322 | 0.008206147 | 0.012789603 | Up |
| PLXND1 | 1644.656058 | 2792.04703 | 497.265085 | 0.178097034 | -2.489264603 | 5.35E-176 | 3.73E-174 | Down |
| PMAIP1 | 5787.433552 | 3155.56304 | 8419.304063 | 2.667892167 | 1.415700356 | 1.77E-78 | 3.45E-77 | Up |
| PMCH | 11.74646271 | 1.42559874 | 22.06732667 | 15.12323559 | 3.91869493 | 0.000149921 | 0.000280339 | Up |
| PMEL | 79.56664637 | 134.1123659 | 25.02092682 | 0.186666714 | -2.421463399 | 1.73E-15 | 5.93E-15 | Down |
| PMEPA1 | 438.6379828 | 191.9456308 | 685.3303348 | 3.573580899 | 1.837370448 | 1.95E-37 | 1.45E-36 | Up |
| PMFBP1 | 137.2546596 | 198.6353209 | 75.87399834 | 0.382071236 | -1.388086446 | 1.11E-06 | 2.41E-06 | Down |
| PMP22 | 474.3180406 | 683.7739551 | 264.8621262 | 0.387356118 | -1.368267568 | 4.27E-20 | 1.77E-19 | Down |
| PMS1 | 958.4685758 | 1315.482753 | 601.4543988 | 0.457206158 | -1.12908326 | 1.22E-22 | 5.54E-22 | Down |
| PNO1 | 1649.047546 | 878.1493796 | 2419.945711 | 2.755259702 | 1.462188309 | 1.33E-81 | 2.77E-80 | Up |
| PNPLA2 | 5240.780777 | 7218.144934 | 3263.41662 | 0.45211153 | -1.145249385 | 1.54E-94 | 3.94E-93 | Down |
| PNRC1 | 5736.395194 | 2261.730908 | 9211.05948 | 4.07286219 | 2.026043 | 4.69E-280 | 7.15E-278 | Up |
| POC5 | 1359.993976 | 753.0805425 | 1966.90741 | 2.610803726 | 1.384494003 | 7.17E-57 | 8.79E-56 | Up |
| PODXL | 880.1159213 | 1535.520752 | 224.7110909 | 0.14633519 | -2.772651351 | 7.48E-125 | 2.97E-123 | Down |
| PODXL2 | 15.94890302 | 8.019388042 | 23.878418 | 2.964874641 | 1.567971107 | 0.02645175 | 0.038545249 | Up |
| POF1B | 6.256662461 | 0 | 12.51332492 | 61.44312857 | 5.941179772 | 0.000105536 | 0.000199875 | Up |
| POLD1 | 6333.634426 | 8449.189515 | 4218.079337 | 0.499214643 | -1.002267841 | 1.07E-74 | 1.92E-73 | Down |
| POLD4 | 526.3164484 | 335.3814702 | 717.2514265 | 2.137614686 | 1.096001825 | 1.34E-16 | 4.82E-16 | Up |
| POLE | 10633.14184 | 14645.99086 | 6620.292812 | 0.452012432 | -1.145565642 | 7.54E-101 | 2.09E-99 | Down |
| POLE2 | 819.6304614 | 1166.825194 | 472.435729 | 0.404877286 | -1.304443386 | 1.37E-36 | 9.91E-36 | Down |
| POLH | 4623.670263 | 6223.632547 | 3023.707978 | 0.485821836 | -1.041500758 | 8.55E-76 | 1.58E-74 | Down |
| POLI | 301.7096501 | 432.0328694 | 171.3864308 | 0.396542843 | -1.33445135 | 4.83E-14 | 1.55E-13 | Down |
| POLL | 64.85969452 | 37.99001251 | 91.72937654 | 2.414569273 | 1.271765855 | 0.000911089 | 0.001578531 | Up |
| POLN | 29.89761735 | 16.01829809 | 43.77693662 | 2.721479044 | 1.444390927 | 0.004583296 | 0.007378702 | Up |
| POLR1F | 3175.645073 | 1875.546345 | 4475.743802 | 2.386105589 | 1.254657886 | 4.74E-43 | 4.13E-42 | Up |
| POLR1G | 794.886313 | 1091.746406 | 498.0262203 | 0.456261628 | -1.132066767 | 1.44E-19 | 5.90E-19 | Down |
| POLR3C | 3641.854264 | 2170.975477 | 5112.73305 | 2.35453754 | 1.235443725 | 1.13E-93 | 2.89E-92 | Up |
| POLR3G | 413.3192967 | 632.0731439 | 194.5654496 | 0.30789621 | -1.699483984 | 7.59E-36 | 5.35E-35 | Down |
| POMGNT2 | 413.4737508 | 560.3507101 | 266.5967915 | 0.475679298 | -1.071938856 | 4.11E-14 | 1.33E-13 | Down |
| POMP | 3424.993975 | 2144.806011 | 4705.181938 | 2.194321475 | 1.133774901 | 4.90E-46 | 4.62E-45 | Up |
| POPDC2 | 51.67627188 | 20.79350136 | 82.55904241 | 3.981780643 | 1.993413745 | 1.53E-07 | 3.52E-07 | Up |
| POU2F1 | 2717.102951 | 4010.582069 | 1423.623834 | 0.354969496 | -1.49423304 | 1.59E-67 | 2.44E-66 | Down |
| POU3F3 | 448.3535679 | 620.9139667 | 275.793169 | 0.444147336 | -1.170889757 | 6.31E-21 | 2.70E-20 | Down |
| POU4F3 | 2.675653986 | 0 | 5.351307972 | 26.28171083 | 4.715987287 | 0.012732117 | 0.019339377 | Up |
| PPARA | 469.8030289 | 709.2577018 | 230.3483561 | 0.324789669 | -1.622422349 | 2.45E-35 | 1.71E-34 | Down |
| PPARD | 2117.957742 | 1392.976622 | 2842.938862 | 2.04119251 | 1.029412253 | 3.09E-53 | 3.49E-52 | Up |
| PPARGC1A | 12.52616911 | 0.356399685 | 24.69593854 | 62.26711655 | 5.960398568 | 1.16E-05 | 2.36E-05 | Up |
| PPEF1 | 241.0277163 | 160.6246299 | 321.4308026 | 2.001894649 | 1.001366054 | 1.05E-08 | 2.57E-08 | Up |
| PPFIA4 | 1378.796074 | 2447.334552 | 310.2575967 | 0.126775118 | -2.979656482 | 1.62E-247 | 2.01E-245 | Down |
| PPIF | 4595.019426 | 2217.210779 | 6972.828073 | 3.144379298 | 1.652775257 | 1.29E-153 | 7.03E-152 | Up |
| PPIL4 | 1672.013473 | 1112.130511 | 2231.896436 | 2.007170684 | 1.005163305 | 1.48E-24 | 7.26E-24 | Up |
| PPIP5K1 | 1282.657912 | 1738.626935 | 826.6888897 | 0.475463216 | -1.072594363 | 4.37E-22 | 1.95E-21 | Down |
| PPM1D | 2533.339304 | 1524.989674 | 3541.688935 | 2.321848356 | 1.21527375 | 7.62E-75 | 1.38E-73 | Up |
| PPM1L | 1095.207547 | 1698.174058 | 492.2410349 | 0.289863031 | -1.786556751 | 4.13E-88 | 9.56E-87 | Down |
| PPM1M | 362.0761389 | 543.010202 | 181.1420758 | 0.333481511 | -1.584321318 | 4.14E-20 | 1.72E-19 | Down |
| PPM1N | 43.13055394 | 64.46543083 | 21.79567705 | 0.337877705 | -1.565426939 | 0.000331279 | 0.000598876 | Down |
| PPP1R13L | 391.479331 | 555.0390134 | 227.9196485 | 0.410384858 | -1.284950594 | 1.76E-16 | 6.29E-16 | Down |
| PPP1R14B | 4303.9511 | 6181.143845 | 2426.758355 | 0.392631516 | -1.348752116 | 1.45E-75 | 2.66E-74 | Down |
| PPP1R15A | 21050.46323 | 3611.305047 | 38489.62142 | 10.65648524 | 3.413659777 | 0 | 0 | Up |
| PPP1R16B | 8803.30408 | 4900.488877 | 12706.11928 | 2.592702269 | 1.374456545 | 2.46E-133 | 1.07E-131 | Up |
| PPP1R1B | 27.46243698 | 51.05041233 | 3.874461632 | 0.07592576 | -3.719266752 | 3.86E-09 | 9.74E-09 | Down |
| PPP1R32 | 110.9575753 | 165.5520749 | 56.36307561 | 0.340293413 | -1.555148871 | 3.94E-08 | 9.36E-08 | Down |
| PPP1R3E | 517.6295234 | 867.7708271 | 167.4882198 | 0.193047062 | -2.372975494 | 6.84E-78 | 1.31E-76 | Down |
| PPP1R8 | 5428.194676 | 3232.431689 | 7623.957663 | 2.358630203 | 1.237949244 | 4.84E-108 | 1.54E-106 | Up |
| PPP2R2C | 30.36606718 | 0.385822986 | 60.34631138 | 152.0964359 | 7.248842537 | 1.14E-08 | 2.81E-08 | Up |
| PPP2R3B-2 | 419.8813862 | 744.3577303 | 95.40504216 | 0.128179652 | -2.963760839 | 0.007269838 | 0.011411103 | Down |
| PPP2R5B | 1063.741947 | 469.4065903 | 1658.077304 | 3.534937453 | 1.821684688 | 2.64E-69 | 4.23E-68 | Up |
| PPP4R4 | 66.02326285 | 106.9727351 | 25.07379061 | 0.234143968 | -2.094532221 | 5.84E-10 | 1.54E-09 | Down |
| PRAG1 | 1750.636633 | 835.5318045 | 2665.741461 | 3.190128433 | 1.673614507 | 3.36E-93 | 8.48E-92 | Up |
| PRAME | 31.49541988 | 58.81677333 | 4.174066426 | 0.071032596 | -3.815374977 | 1.47E-10 | 4.02E-10 | Down |
| PRCD | 9.832185353 | 0 | 19.66437071 | 96.55639864 | 6.593299962 | 2.57E-06 | 5.42E-06 | Up |
| PRDM11 | 200.4725857 | 66.14126068 | 334.8039108 | 5.066206861 | 2.340905986 | 3.08E-30 | 1.85E-29 | Up |
| PRELID2 | 111.3395254 | 174.1401917 | 48.53885912 | 0.278658984 | -1.84342743 | 5.15E-12 | 1.51E-11 | Down |
| PREX1 | 3538.978998 | 5132.720913 | 1945.237083 | 0.378979473 | -1.399808386 | 1.72E-115 | 6.03E-114 | Down |
| PREX2 | 3.133078304 | 0 | 6.266156607 | 30.76357656 | 4.943151335 | 0.006803317 | 0.010720074 | Up |
| PRH1 | 79.4843991 | 44.13502189 | 114.8337763 | 2.599250052 | 1.37809543 | 1.36E-05 | 2.73E-05 | Up |
| PRICKLE4 | 178.2462728 | 248.8854784 | 107.6070672 | 0.432120837 | -1.210493296 | 5.70E-09 | 1.42E-08 | Down |
| PRKAR1A | 20223.36235 | 11087.85946 | 29358.86525 | 2.647795442 | 1.404791669 | 1.10E-127 | 4.47E-126 | Up |
| PRKAR1B | 620.1364584 | 247.5317496 | 992.7411673 | 4.014620355 | 2.005263565 | 1.29E-53 | 1.46E-52 | Up |
| PRKAR2B | 153.6041389 | 230.6292376 | 76.57904027 | 0.332007689 | -1.590711443 | 5.87E-14 | 1.88E-13 | Down |
| PRKCH | 262.3717668 | 63.74931138 | 460.9942222 | 7.248904044 | 2.857762892 | 5.53E-51 | 5.87E-50 | Up |
| PRKCZ | 4717.30154 | 7478.41507 | 1956.18801 | 0.261569966 | -1.934731198 | 1.50E-174 | 1.02E-172 | Down |
| PRKD2 | 2539.561419 | 1411.14481 | 3667.978029 | 2.598968043 | 1.377938895 | 5.18E-84 | 1.14E-82 | Up |
| PRKD3 | 1943.755995 | 2942.676648 | 944.8353422 | 0.321078854 | -1.63900044 | 3.88E-71 | 6.50E-70 | Down |
| PRKG1 | 4.474818272 | 0 | 8.949636544 | 43.93897476 | 5.457429302 | 0.000856541 | 0.001487367 | Up |
| PRL | 32.48244421 | 3.272077057 | 61.69281137 | 18.65799085 | 4.221721736 | 1.32E-10 | 3.61E-10 | Up |
| PRMT5 | 9433.991411 | 5489.629744 | 13378.35308 | 2.43704887 | 1.285135181 | 5.93E-102 | 1.69E-100 | Up |
| PROC | 50.16639074 | 75.66146172 | 24.67131975 | 0.326367434 | -1.615430986 | 4.12E-05 | 8.04E-05 | Down |
| PROCA1 | 565.4569054 | 849.8763935 | 281.0374173 | 0.330670856 | -1.596532198 | 6.72E-36 | 4.75E-35 | Down |
| PROKR1 | 24.67694502 | 3.246231879 | 46.10765815 | 14.00468146 | 3.807837264 | 1.13E-07 | 2.62E-07 | Up |
| PROP1 | 2.377366668 | 4.754733337 | 0 | 0.034559953 | -4.854754937 | 0.018474554 | 0.027478076 | Down |
| PROSER3 | 1364.486782 | 1917.266242 | 811.7073227 | 0.423405823 | -1.239886983 | 4.98E-49 | 5.04E-48 | Down |
| PRPF3 | 3446.982061 | 2280.757272 | 4613.206849 | 2.022744002 | 1.016313744 | 1.32E-61 | 1.78E-60 | Up |
| PRPS2 | 4.81911805 | 9.04074097 | 0.597495129 | 0.06585503 | -3.924562554 | 0.01347148 | 0.020381273 | Down |
| PRR11 | 6667.376896 | 9187.0632 | 4147.690593 | 0.45146889 | -1.147301518 | 1.86E-105 | 5.69E-104 | Down |
| PRR22 | 95.05051087 | 42.00045994 | 148.1005618 | 3.535669971 | 1.821983616 | 1.73E-09 | 4.45E-09 | Up |
| PRR5 | 492.8377373 | 783.5350288 | 202.1404459 | 0.258011563 | -1.954492371 | 2.21E-48 | 2.22E-47 | Down |
| PRRT2 | 19.63980267 | 35.39541212 | 3.884193218 | 0.109586266 | -3.189861088 | 1.78E-06 | 3.81E-06 | Down |
| PRRT4 | 679.7213025 | 973.9455054 | 385.4970996 | 0.395699541 | -1.337522705 | 6.87E-36 | 4.85E-35 | Down |
| PRSS27 | 58.98081717 | 34.25483497 | 83.70679937 | 2.451204375 | 1.293490778 | 0.000267877 | 0.00048949 | Up |
| PRSS53 | 209.461191 | 319.7026639 | 99.21971816 | 0.310368591 | -1.687945528 | 9.74E-21 | 4.14E-20 | Down |
| PRSS57 | 13.09177501 | 23.20771771 | 2.975832306 | 0.128298904 | -2.962419249 | 0.000353491 | 0.000636955 | Down |
| PRX | 207.0134429 | 316.0366007 | 97.990285 | 0.310209792 | -1.688683867 | 4.10E-17 | 1.51E-16 | Down |
| PSAT1 | 6242.809861 | 11189.18378 | 1296.435942 | 0.11587913 | -3.10930734 | 0 | 0 | Down |
| PSD2 | 33.96433202 | 45.31832796 | 22.61033608 | 0.499729369 | -1.000781087 | 0.032351987 | 0.046517509 | Down |
| PSEN2 | 18.1324175 | 29.44278605 | 6.82204894 | 0.232007009 | -2.107759706 | 0.003027392 | 0.004971345 | Down |
| PSIP1 | 3841.926492 | 5613.781685 | 2070.0713 | 0.368748604 | -1.439290509 | 2.44E-77 | 4.64E-76 | Down |
| PSMC1 | 4750.333184 | 2873.722077 | 6626.94429 | 2.306225369 | 1.205533503 | 8.08E-80 | 1.62E-78 | Up |
| PSMC6 | 2909.383106 | 1745.994631 | 4072.771581 | 2.332693484 | 1.22199675 | 5.29E-87 | 1.20E-85 | Up |
| PSMD12 | 2403.529071 | 1591.727838 | 3215.330304 | 2.019617159 | 1.01408184 | 8.75E-37 | 6.36E-36 | Up |
| PSMD6 | 3326.554369 | 1994.906172 | 4658.202565 | 2.335338887 | 1.223631918 | 1.07E-79 | 2.14E-78 | Up |
| PSME1 | 5723.797258 | 8032.877482 | 3414.717033 | 0.425096355 | -1.234138207 | 5.62E-82 | 1.17E-80 | Down |
| PSPH | 1966.634188 | 2900.884265 | 1032.384111 | 0.355914403 | -1.490397778 | 5.01E-96 | 1.30E-94 | Down |
| PSPN | 43.67637149 | 24.72542252 | 62.62732047 | 2.543945883 | 1.347067981 | 0.002486923 | 0.004129109 | Up |
| PSRC1 | 1883.768915 | 2660.143658 | 1107.394171 | 0.416283291 | -1.264362442 | 3.32E-58 | 4.18E-57 | Down |
| PSTK | 300.7674109 | 194.0694503 | 407.4653715 | 2.100910901 | 1.071014979 | 6.00E-12 | 1.76E-11 | Up |
| PSTPIP1 | 23.13628945 | 1.543291942 | 44.72928696 | 30.04406695 | 4.909008213 | 3.00E-08 | 7.18E-08 | Up |
| PSTPIP2 | 11.30484806 | 4.418545539 | 18.19115058 | 4.11327077 | 2.040286045 | 0.017822046 | 0.02657025 | Up |
| PTAFR | 134.0282404 | 29.96472507 | 238.0917556 | 7.957101631 | 2.992243026 | 1.62E-27 | 8.93E-27 | Up |
| PTCRA | 361.9864518 | 525.5819402 | 198.3909634 | 0.377564967 | -1.405203186 | 1.69E-22 | 7.67E-22 | Down |
| PTGER1 | 13.48901293 | 22.5407093 | 4.437316567 | 0.19736754 | -2.341043362 | 0.007586527 | 0.011881304 | Down |
| PTGER3 | 14.64612764 | 2.213878476 | 27.07837681 | 12.23306766 | 3.612714325 | 8.09E-05 | 0.000154703 | Up |
| PTGER4 | 544.9672505 | 209.0843102 | 880.8501907 | 4.207978853 | 2.073127455 | 5.77E-47 | 5.58E-46 | Up |
| PTGES3L-AARSD1 | 22.10327082 | 12.05031347 | 32.15622818 | 2.684558791 | 1.424685 | 0.026121215 | 0.038095613 | Up |
| PTGFRN | 56.58851358 | 28.07066673 | 85.10636044 | 3.033711378 | 1.601083837 | 2.90E-05 | 5.72E-05 | Up |
| PTGR2 | 419.1374953 | 600.9071726 | 237.3678179 | 0.394872621 | -1.340540755 | 3.27E-21 | 1.42E-20 | Down |
| PTGS1 | 395.58872 | 37.75103992 | 753.4264002 | 20.00554852 | 4.32232828 | 1.03E-110 | 3.41E-109 | Up |
| PTH1R | 3.275582012 | 0 | 6.551164023 | 32.1693551 | 5.007615109 | 0.004777604 | 0.00767282 | Up |
| PTK7 | 3137.810475 | 4382.728277 | 1892.892673 | 0.431901151 | -1.211226934 | 1.75E-67 | 2.68E-66 | Down |
| PTMA | 96210.19081 | 139757.692 | 52662.68962 | 0.376814998 | -1.408071708 | 1.44E-244 | 1.74E-242 | Down |
| PTP4A3 | 509.1737012 | 874.4866537 | 143.8607487 | 0.164584304 | -2.60310134 | 7.53E-62 | 1.02E-60 | Down |
| PTPN12 | 1051.004478 | 1416.663492 | 685.3454628 | 0.483742344 | -1.047689267 | 6.71E-25 | 3.35E-24 | Down |
| PTPN14 | 14.23671552 | 2.601490523 | 25.87194051 | 9.993270385 | 3.32095689 | 7.29E-05 | 0.000140078 | Up |
| PTPN22 | 1798.288513 | 703.5486586 | 2893.028368 | 4.11329562 | 2.040294761 | 5.96E-79 | 1.17E-77 | Up |
| PTPN4 | 704.9974374 | 975.1502964 | 434.8445784 | 0.445903718 | -1.165195866 | 6.92E-22 | 3.07E-21 | Down |
| PTPN7 | 3647.981645 | 2317.630158 | 4978.333133 | 2.148130225 | 1.103081456 | 8.04E-60 | 1.05E-58 | Up |
| PTPRB | 9.05384079 | 1.078410468 | 17.02927111 | 15.50254474 | 3.954433148 | 0.000626157 | 0.001100397 | Up |
| PTPRC | 178.2947129 | 25.40854054 | 331.1808852 | 13.01895033 | 3.702541229 | 3.94E-49 | 4.02E-48 | Up |
| PTPRF | 434.9099655 | 665.5972313 | 204.2226997 | 0.306815099 | -1.704558614 | 6.26E-37 | 4.60E-36 | Down |
| PTPRH | 25.83997416 | 1.929114928 | 49.75083338 | 26.6923393 | 4.738353843 | 8.48E-09 | 2.10E-08 | Up |
| PTPRM | 13.9735327 | 23.18723972 | 4.759825688 | 0.205369449 | -2.283706511 | 0.002532608 | 0.004198614 | Down |
| PTPRO | 538.0297753 | 2.215667538 | 1073.843883 | 485.1707339 | 8.922348718 | 4.99E-50 | 5.17E-49 | Up |
| PTPRR | 64.40774082 | 10.09330626 | 118.7221754 | 11.61712072 | 3.538180639 | 1.48E-14 | 4.86E-14 | Up |
| PTPRS | 4147.42489 | 6587.459058 | 1707.390722 | 0.259175213 | -1.948000347 | 1.52E-201 | 1.34E-199 | Down |
| PTS | 461.7866787 | 281.3690573 | 642.2043 | 2.280564394 | 1.189390907 | 5.25E-18 | 2.01E-17 | Up |
| PTX3 | 5.101116815 | 0.365611098 | 9.836622531 | 24.79692523 | 4.632089335 | 0.003050611 | 0.005005325 | Up |
| PURG | 28.28797874 | 50.32277631 | 6.25318116 | 0.12425178 | -3.00866157 | 1.05E-07 | 2.44E-07 | Down |
| PWWP2B | 220.9412356 | 374.1981069 | 67.68436432 | 0.180888746 | -2.466825442 | 4.64E-40 | 3.71E-39 | Down |
| PWWP3A | 4157.493364 | 6711.859673 | 1603.127054 | 0.238841647 | -2.06587367 | 5.31E-233 | 5.98E-231 | Down |
| PXMP2 | 642.0632003 | 883.2996726 | 400.826728 | 0.453973081 | -1.139321342 | 2.39E-18 | 9.32E-18 | Down |
| PXN | 558.8519319 | 805.8124513 | 311.8914125 | 0.387080566 | -1.369294217 | 1.04E-31 | 6.51E-31 | Down |
| PYCARD | 777.1633717 | 1090.590917 | 463.7358263 | 0.425111445 | -1.234086995 | 5.76E-28 | 3.21E-27 | Down |
| PYCR1 | 3114.828386 | 4849.841905 | 1379.814867 | 0.284498373 | -1.813507691 | 1.43E-166 | 8.93E-165 | Down |
| PYGM | 83.15788644 | 52.86388922 | 113.4518837 | 2.146917811 | 1.102266962 | 0.000447982 | 0.000799296 | Up |
| PYROXD2 | 255.9305293 | 349.0861034 | 162.7749552 | 0.465985844 | -1.101641967 | 1.45E-08 | 3.54E-08 | Down |
| QARS1 | 11965.42127 | 16830.35241 | 7100.490121 | 0.421895134 | -1.245043648 | 6.40E-104 | 1.90E-102 | Down |
| QRICH2 | 290.2320982 | 109.3191515 | 471.1450448 | 4.306247846 | 2.106431356 | 1.23E-32 | 7.92E-32 | Up |
| QRSL1 | 6344.012939 | 8921.536064 | 3766.489813 | 0.42217876 | -1.244074096 | 1.55E-121 | 5.87E-120 | Down |
| QSOX2 | 5381.60257 | 2587.168904 | 8176.036236 | 3.160914673 | 1.660342091 | 1.14E-163 | 6.97E-162 | Up |
| RAB11FIP4 | 22.69244701 | 0 | 45.38489402 | 222.8038086 | 7.799630084 | 1.43E-09 | 3.70E-09 | Up |
| RAB15 | 85.34394479 | 43.09192605 | 127.5959635 | 2.967153484 | 1.569079556 | 1.70E-06 | 3.64E-06 | Up |
| RAB17 | 112.2825083 | 35.00422195 | 189.5607946 | 5.412304556 | 2.436243023 | 1.44E-20 | 6.06E-20 | Up |
| RAB20 | 442.1702521 | 238.860214 | 645.4802902 | 2.703152304 | 1.4346428 | 1.15E-27 | 6.34E-27 | Up |
| RAB26 | 77.48369903 | 149.2914842 | 5.675913903 | 0.037963943 | -4.719226347 | 4.87E-27 | 2.64E-26 | Down |
| RAB27B | 44.13531162 | 25.40086015 | 62.86976309 | 2.465487847 | 1.301873141 | 0.007476992 | 0.011719023 | Up |
| RAB30 | 2974.652792 | 1049.889467 | 4899.416116 | 4.665823413 | 2.222131707 | 4.23E-223 | 4.62E-221 | Up |
| RAB37 | 19.79824336 | 30.36871283 | 9.227773886 | 0.303623308 | -1.719645551 | 0.006120277 | 0.009710856 | Down |
| RAB3B | 4.925953577 | 0.356399685 | 9.495507469 | 23.94785948 | 4.581824805 | 0.004680798 | 0.007528681 | Up |
| RAB3D | 336.8193716 | 605.0210169 | 68.61772636 | 0.113332691 | -3.141364025 | 6.21E-74 | 1.09E-72 | Down |
| RAB3GAP2 | 3080.615444 | 1992.165776 | 4169.065111 | 2.092578987 | 1.06528208 | 7.66E-34 | 5.09E-33 | Up |
| RAB40B | 96.62820024 | 137.7400524 | 55.51634808 | 0.402824352 | -1.311777193 | 6.12E-06 | 1.26E-05 | Down |
| RAB43 | 87.10854429 | 135.8326804 | 38.3844082 | 0.282555615 | -1.823393235 | 6.48E-09 | 1.61E-08 | Down |
| RAB4B | 1068.32437 | 1449.072141 | 687.5765989 | 0.474590738 | -1.07524415 | 3.65E-32 | 2.32E-31 | Down |
| RAB6C | 16.67028057 | 7.784259682 | 25.55630146 | 3.290431184 | 1.71827665 | 0.010418447 | 0.016012663 | Up |
| RAB9A | 1145.255331 | 687.9276172 | 1602.583044 | 2.328976057 | 1.219695808 | 1.53E-40 | 1.24E-39 | Up |
| RAB9B | 41.53296908 | 76.23177101 | 6.834167157 | 0.089876143 | -3.475917979 | 1.34E-11 | 3.86E-11 | Down |
| RAC3 | 285.9661734 | 390.9404629 | 180.9918839 | 0.46303789 | -1.110797842 | 2.27E-10 | 6.13E-10 | Down |
| RAD51 | 2260.91317 | 3135.346404 | 1386.479936 | 0.442212914 | -1.177186937 | 1.99E-58 | 2.52E-57 | Down |
| RAD51AP1 | 2288.272126 | 3428.054497 | 1148.489756 | 0.335014209 | -1.577705809 | 2.42E-69 | 3.89E-68 | Down |
| RAD51C | 1102.174249 | 715.1890515 | 1489.159446 | 2.082100446 | 1.058039669 | 2.79E-33 | 1.82E-32 | Up |
| RAD54L | 2229.084539 | 3653.268311 | 804.9007671 | 0.220316581 | -2.182350016 | 1.18E-140 | 5.60E-139 | Down |
| RAD9A | 1047.537968 | 1402.381451 | 692.6944849 | 0.494012894 | -1.017379396 | 3.08E-27 | 1.68E-26 | Down |
| RADIL | 4.907432628 | 0 | 9.814865256 | 48.20094495 | 5.590989525 | 0.000596691 | 0.001050742 | Up |
| RAE1 | 5033.100729 | 3131.673343 | 6934.528114 | 2.214387515 | 1.146907714 | 1.91E-83 | 4.16E-82 | Up |
| RAG1 | 28675.31364 | 51774.52005 | 5576.107241 | 0.107699451 | -3.214917195 | 0 | 0 | Down |
| RALGAPA1 | 1530.377649 | 867.1940781 | 2193.561219 | 2.53005998 | 1.339171587 | 1.31E-56 | 1.60E-55 | Up |
| RALGAPA2 | 3252.308237 | 1798.205316 | 4706.411158 | 2.61710281 | 1.3879706 | 5.58E-101 | 1.56E-99 | Up |
| RALGPS1 | 663.655223 | 1077.305841 | 250.0046049 | 0.232072248 | -2.107354082 | 1.81E-72 | 3.10E-71 | Down |
| RAMAC | 1287.79798 | 816.2708779 | 1759.325082 | 2.156470149 | 1.108671745 | 1.93E-33 | 1.27E-32 | Up |
| RANBP3L | 29.31704538 | 47.63711816 | 10.9969726 | 0.231083201 | -2.11351571 | 0.000165092 | 0.000307587 | Down |
| RAP1GAP | 17.43269807 | 6.002261257 | 28.86313489 | 4.844092156 | 2.276226312 | 0.001260573 | 0.00215364 | Up |
| RAP1GAP2 | 1013.344482 | 1679.266951 | 347.4220135 | 0.206910805 | -2.272919108 | 3.17E-130 | 1.32E-128 | Down |
| RAPGEF1 | 12197.10751 | 7304.412609 | 17089.8024 | 2.339652568 | 1.226294309 | 1.52E-90 | 3.65E-89 | Up |
| RAPGEF3 | 146.6090373 | 214.060318 | 79.15775665 | 0.36946711 | -1.436482152 | 2.12E-07 | 4.83E-07 | Down |
| RAPSN | 26.71175139 | 11.72128998 | 41.7022128 | 3.547547874 | 1.826822154 | 0.000354765 | 0.000639001 | Up |
| RARA | 3452.897739 | 1564.833122 | 5340.962356 | 3.413785944 | 1.771372599 | 8.44E-124 | 3.31E-122 | Up |
| RASA3 | 1588.392305 | 2509.325315 | 667.4592944 | 0.26597935 | -1.910613851 | 1.41E-135 | 6.39E-134 | Down |
| RASA4B | 178.8746052 | 269.7034771 | 88.04573332 | 0.326298179 | -1.61573716 | 1.45E-07 | 3.34E-07 | Down |
| RASAL1 | 6126.276244 | 10692.98326 | 1559.569232 | 0.145843685 | -2.77750518 | 6.12E-291 | 9.99E-289 | Down |
| RASD1 | 3571.265528 | 5953.47733 | 1189.053725 | 0.199734552 | -2.323844166 | 6.51E-194 | 5.28E-192 | Down |
| RASD2 | 63.62420138 | 21.65879248 | 105.5896103 | 4.865515828 | 2.282592764 | 6.44E-09 | 1.60E-08 | Up |
| RASGRF1 | 20.50780155 | 0.722010783 | 40.29359232 | 54.73759113 | 5.774460042 | 5.14E-07 | 1.14E-06 | Up |
| RASGRF2 | 14.48849135 | 6.636002138 | 22.34098057 | 3.366028472 | 1.75104738 | 0.011287393 | 0.017271641 | Up |
| RASGRP2 | 1868.557021 | 2536.897907 | 1200.216134 | 0.473104316 | -1.079769774 | 1.69E-49 | 1.74E-48 | Down |
| RASGRP3 | 97.15399961 | 28.81646752 | 165.4915317 | 5.747963884 | 2.523050997 | 2.08E-16 | 7.40E-16 | Up |
| RASL10A | 64.82646879 | 13.48334263 | 116.1695949 | 8.604658937 | 3.105118009 | 0.002802303 | 0.004620868 | Up |
| RASL11A | 32.61073284 | 21.70816156 | 43.51330413 | 2.003100886 | 1.002235084 | 0.034259377 | 0.049097202 | Up |
| RASSF1 | 2877.666086 | 1132.276491 | 4623.055681 | 4.084155085 | 2.03003765 | 2.55E-186 | 1.92E-184 | Up |
| RASSF10 | 36.19045837 | 51.22490502 | 21.15601172 | 0.412973536 | -1.275878761 | 0.005520396 | 0.008809643 | Down |
| RASSF4 | 109.445199 | 190.2066354 | 28.68376252 | 0.150746531 | -2.729803288 | 7.49E-17 | 2.72E-16 | Down |
| RASSF6 | 2.979649694 | 0 | 5.959299388 | 29.26117555 | 4.870915825 | 0.00744956 | 0.011677348 | Up |
| RAVER2 | 1303.156293 | 1784.629827 | 821.6827591 | 0.460457357 | -1.118860542 | 6.58E-41 | 5.37E-40 | Down |
| RB1 | 10594.86826 | 5617.440108 | 15572.29641 | 2.772085812 | 1.470971918 | 2.68E-72 | 4.58E-71 | Up |
| RBBP4 | 15686.86734 | 21589.51629 | 9784.218387 | 0.453191501 | -1.141807289 | 2.32E-151 | 1.24E-149 | Down |
| RBBP7 | 5723.460457 | 7661.959077 | 3784.961838 | 0.493989731 | -1.017447042 | 8.39E-80 | 1.68E-78 | Down |
| RBFA | 1320.310262 | 1974.460973 | 666.1595508 | 0.337422358 | -1.567372525 | 2.13E-53 | 2.42E-52 | Down |
| RBKS | 500.8007286 | 109.3170883 | 892.2843689 | 8.167763302 | 3.029941058 | 8.73E-101 | 2.41E-99 | Up |
| RBL2 | 5275.599801 | 7358.355656 | 3192.843945 | 0.433902679 | -1.204556602 | 2.31E-59 | 2.98E-58 | Down |
| RBM11 | 3.694612642 | 7.389225283 | 0 | 0.022264449 | -5.489114277 | 0.001446986 | 0.002456989 | Down |
| RBM12B | 3394.087637 | 4579.446667 | 2208.728608 | 0.482346103 | -1.051859386 | 2.03E-46 | 1.94E-45 | Down |
| RBM18 | 2088.700723 | 1292.222628 | 2885.178818 | 2.232493084 | 1.158655706 | 1.45E-41 | 1.20E-40 | Up |
| RBM19 | 1631.672209 | 1040.169228 | 2223.17519 | 2.137857553 | 1.096165728 | 2.18E-36 | 1.57E-35 | Up |
| RBM20 | 13.91806398 | 25.14577795 | 2.690350011 | 0.106873437 | -3.22602478 | 0.000229344 | 0.000421299 | Down |
| RBM38 | 6257.947266 | 4154.029878 | 8361.864654 | 2.013065092 | 1.009393822 | 3.25E-61 | 4.35E-60 | Up |
| RBM43 | 226.707106 | 304.7832462 | 148.6309659 | 0.48787244 | -1.035424107 | 4.58E-09 | 1.15E-08 | Down |
| RBM44 | 29.3645426 | 46.51775171 | 12.2113335 | 0.262617401 | -1.928965584 | 0.000135506 | 0.000254447 | Down |
| RBM5 | 9185.276846 | 5616.542306 | 12754.01139 | 2.270609595 | 1.183079673 | 1.43E-130 | 6.00E-129 | Up |
| RBP1 | 1081.508279 | 1577.769679 | 585.2468786 | 0.370995466 | -1.430526541 | 6.85E-59 | 8.78E-58 | Down |
| RBSN | 1162.691141 | 1608.732424 | 716.6498568 | 0.445390264 | -1.166858074 | 1.85E-37 | 1.38E-36 | Down |
| RCAN1 | 4113.944908 | 6769.797535 | 1458.092282 | 0.215383726 | -2.215018847 | 0 | 0 | Down |
| RCAN2 | 14.83747202 | 5.531746492 | 24.14319754 | 4.36441019 | 2.1257867 | 0.003846924 | 0.00624613 | Up |
| RCBTB2 | 739.3704534 | 473.7455118 | 1004.995395 | 2.120891483 | 1.084670806 | 6.41E-21 | 2.75E-20 | Up |
| RCHY1 | 1533.155941 | 977.1999499 | 2089.111932 | 2.137270303 | 1.095769379 | 9.03E-42 | 7.56E-41 | Up |
| RCOR1 | 5674.378706 | 3723.605986 | 7625.151426 | 2.047776373 | 1.034058175 | 1.62E-65 | 2.36E-64 | Up |
| RCOR2 | 257.4597976 | 472.2416529 | 42.67794234 | 0.090295141 | -3.469207829 | 3.60E-66 | 5.38E-65 | Down |
| RCVRN | 32.51857712 | 16.81936736 | 48.21778687 | 2.858898516 | 1.515459409 | 0.002003306 | 0.003361706 | Up |
| RDH10 | 676.7453514 | 411.8547275 | 941.6359753 | 2.286111202 | 1.192895581 | 2.81E-28 | 1.59E-27 | Up |
| REC8 | 104.8331923 | 146.4423338 | 63.22405089 | 0.432025494 | -1.210811647 | 4.20E-06 | 8.75E-06 | Down |
| RECQL4 | 2156.662062 | 3020.617186 | 1292.706938 | 0.427983105 | -1.224374247 | 3.68E-69 | 5.87E-68 | Down |
| REELD1 | 29.50723067 | 8.676918934 | 50.3375424 | 5.7551263 | 2.52484759 | 3.10E-05 | 6.10E-05 | Up |
| REEP2 | 275.6385912 | 423.6671086 | 127.6100738 | 0.301311945 | -1.730670229 | 4.16E-25 | 2.09E-24 | Down |
| REEP6 | 122.1427962 | 185.3134729 | 58.97211955 | 0.318451246 | -1.650855577 | 5.58E-12 | 1.64E-11 | Down |
| RELA | 6323.605431 | 3997.163699 | 8650.047163 | 2.164100133 | 1.113767254 | 8.32E-81 | 1.71E-79 | Up |
| RELB | 3671.312644 | 808.842518 | 6533.782769 | 8.079487927 | 3.014263859 | 0 | 0 | Up |
| REP15 | 22.90205315 | 10.6190895 | 35.1850168 | 3.304490472 | 1.724427836 | 0.004284091 | 0.00691547 | Up |
| REPS1 | 4169.59181 | 2030.714218 | 6308.469403 | 3.106352112 | 1.635221372 | 6.41E-136 | 2.92E-134 | Up |
| REPS2 | 55.23603378 | 96.80573754 | 13.66633003 | 0.141339889 | -2.822759413 | 3.82E-12 | 1.13E-11 | Down |
| RESF1 | 2200.776326 | 2970.539138 | 1431.013515 | 0.481736152 | -1.053684898 | 1.13E-24 | 5.56E-24 | Down |
| REXO4 | 2555.909914 | 1313.004282 | 3798.815546 | 2.894001617 | 1.533065728 | 7.00E-102 | 1.99E-100 | Up |
| RFLNB | 17.72978727 | 4.480970263 | 30.97860428 | 6.961140634 | 2.799323722 | 4.58E-05 | 8.90E-05 | Up |
| RFPL1 | 7.448798744 | 0.356399685 | 14.5411978 | 36.67182459 | 5.196600143 | 0.000541404 | 0.000957396 | Up |
| RFPL4A | 4.638743169 | 0 | 9.277486337 | 45.535817 | 5.508929864 | 0.000980348 | 0.001695562 | Up |
| RFX2 | 393.9756349 | 573.3873775 | 214.5638924 | 0.374257175 | -1.41789812 | 2.52E-18 | 9.81E-18 | Down |
| RGMA | 623.7366701 | 1015.288127 | 232.1852132 | 0.228634253 | -2.128886535 | 2.78E-61 | 3.72E-60 | Down |
| RGMB | 1257.716303 | 1713.193521 | 802.239086 | 0.468351093 | -1.094337662 | 1.01E-39 | 7.95E-39 | Down |
| RGS1 | 2565.384897 | 8.932517226 | 5121.837276 | 578.4072811 | 9.175941903 | 3.82E-202 | 3.44E-200 | Up |
| RGS12 | 1297.081933 | 1753.20933 | 840.9545361 | 0.479571441 | -1.060182347 | 8.13E-34 | 5.39E-33 | Down |
| RGS13 | 166.7853689 | 3.066371997 | 330.5043658 | 109.5037736 | 6.774836777 | 7.52E-12 | 2.19E-11 | Up |
| RGS2 | 877.360848 | 138.6519328 | 1616.069763 | 11.63837604 | 3.540817861 | 2.75E-154 | 1.52E-152 | Up |
| RGS3 | 333.9880468 | 219.5202117 | 448.4558818 | 2.043072773 | 1.030740593 | 1.51E-12 | 4.55E-12 | Up |
| RGS8 | 9.665639242 | 2.919255495 | 16.41202299 | 5.591330994 | 2.483191752 | 0.006505385 | 0.010283328 | Up |
| RGS9 | 1146.918917 | 1552.85437 | 740.9834639 | 0.477150858 | -1.067482628 | 7.38E-33 | 4.76E-32 | Down |
| RGS9BP | 19.09021092 | 33.10810845 | 5.072313401 | 0.153063439 | -2.707798372 | 1.93E-05 | 3.85E-05 | Down |
| RHBDD1 | 1116.937148 | 1554.282772 | 679.5915234 | 0.437138606 | -1.1938373 | 9.52E-40 | 7.51E-39 | Down |
| RHBDD2 | 2306.01367 | 1333.141559 | 3278.885781 | 2.46044495 | 1.298919238 | 2.97E-58 | 3.75E-57 | Up |
| RHBDF1 | 39.03690101 | 10.3563269 | 67.71747511 | 6.547638355 | 2.71097464 | 3.31E-09 | 8.39E-09 | Up |
| RHBDL1 | 61.36634405 | 88.21272012 | 34.51996798 | 0.391289776 | -1.353690679 | 0.000219458 | 0.000403781 | Down |
| RHO | 2.376095456 | 0 | 4.752190912 | 23.34136942 | 4.5448173 | 0.021371252 | 0.031536239 | Up |
| RHOB | 1858.563958 | 637.9894312 | 3079.138485 | 4.822744993 | 2.269854528 | 2.65E-147 | 1.34E-145 | Up |
| RHOBTB3 | 1237.777437 | 1826.257875 | 649.2969978 | 0.355550752 | -1.491872589 | 6.85E-37 | 5.02E-36 | Down |
| RHOD | 3.879564864 | 0 | 7.759129728 | 38.09317865 | 5.251460772 | 0.002894432 | 0.004764855 | Up |
| RHOG | 2429.292579 | 1290.187038 | 3568.398119 | 2.766455442 | 1.468038687 | 3.26E-101 | 9.15E-100 | Up |
| RHOH | 3721.324067 | 1867.748379 | 5574.899755 | 2.985096943 | 1.577777785 | 2.92E-157 | 1.67E-155 | Up |
| RHOXF1 | 14.71802954 | 23.50500469 | 5.93105439 | 0.252614289 | -1.984991849 | 0.007181345 | 0.01127986 | Down |
| RHPN1 | 1450.934477 | 2419.139753 | 482.7292013 | 0.199561105 | -2.325097532 | 2.36E-175 | 1.63E-173 | Down |
| RILPL1 | 47.82465881 | 2.621702411 | 93.0276152 | 35.93125251 | 5.167167321 | 3.44E-15 | 1.16E-14 | Up |
| RIMBP2 | 11.05724125 | 0.356399685 | 21.75808282 | 54.84995943 | 5.777418648 | 2.65E-05 | 5.23E-05 | Up |
| RIMS3 | 25935.70485 | 49585.5273 | 2285.882406 | 0.046099095 | -4.439117752 | 0 | 0 | Down |
| RIN1 | 94.1427629 | 134.0402137 | 54.24531212 | 0.404401344 | -1.306140304 | 2.48E-06 | 5.24E-06 | Down |
| RIN2 | 244.5180906 | 38.53163119 | 450.50455 | 11.72393791 | 3.551385328 | 3.57E-60 | 4.67E-59 | Up |
| RIN3 | 7.074242573 | 0.771645971 | 13.37683918 | 17.90573745 | 4.162350033 | 0.00248711 | 0.004129109 | Up |
| RINL | 206.006503 | 64.90831942 | 347.1046865 | 5.335203612 | 2.415543331 | 4.06E-26 | 2.14E-25 | Up |
| RIOK2 | 1582.769291 | 919.1166797 | 2246.421902 | 2.444215007 | 1.289371199 | 1.72E-50 | 1.81E-49 | Up |
| RIOK3 | 4153.943438 | 2676.025383 | 5631.861493 | 2.104811092 | 1.073690756 | 6.54E-49 | 6.61E-48 | Up |
| RIPOR1 | 4785.238917 | 6540.739008 | 3029.738826 | 0.463234005 | -1.110186932 | 7.95E-87 | 1.79E-85 | Down |
| RIPOR2 | 2129.261034 | 3195.332223 | 1063.189845 | 0.332707885 | -1.587672038 | 3.93E-104 | 1.18E-102 | Down |
| RMI2 | 1996.106415 | 2864.767959 | 1127.444872 | 0.393537474 | -1.345427075 | 3.01E-63 | 4.17E-62 | Down |
| RNASE6 | 336.1087819 | 89.36712705 | 582.8504368 | 6.536713484 | 2.708565463 | 1.97E-50 | 2.07E-49 | Up |
| RNASEH2C | 1338.560693 | 1874.388949 | 802.7324359 | 0.428171318 | -1.223739937 | 1.78E-41 | 1.47E-40 | Down |
| RND1 | 2025.736569 | 576.6525149 | 3474.820623 | 6.023522838 | 2.590607489 | 3.00E-230 | 3.35E-228 | Up |
| RNF103-CHMP3 | 18.58162827 | 4.370699413 | 32.79255712 | 7.450385609 | 2.897315097 | 3.50E-05 | 6.86E-05 | Up |
| RNF112 | 18.56641344 | 27.59657384 | 9.536253036 | 0.345418071 | -1.533584532 | 0.008154719 | 0.012712306 | Down |
| RNF121 | 1185.783594 | 532.6689859 | 1838.898202 | 3.451459757 | 1.787206663 | 2.44E-83 | 5.30E-82 | Up |
| RNF144B | 509.7354067 | 864.2858219 | 155.1849914 | 0.179538158 | -2.4776376 | 9.00E-69 | 1.42E-67 | Down |
| RNF146 | 1208.708877 | 775.7013747 | 1641.716379 | 2.115663085 | 1.0811099 | 5.69E-35 | 3.91E-34 | Up |
| RNF165 | 96.03880707 | 164.0678796 | 28.00973458 | 0.170779855 | -2.549790292 | 1.91E-17 | 7.13E-17 | Down |
| RNF182 | 49.71956602 | 77.71005891 | 21.72907312 | 0.279855021 | -1.837248461 | 6.82E-06 | 1.40E-05 | Down |
| RNF186 | 8.564988044 | 17.12997609 | 0 | 0.009589115 | -6.70438661 | 5.13E-06 | 1.06E-05 | Down |
| RNF187 | 7807.609235 | 11038.58304 | 4576.635431 | 0.414605683 | -1.270188204 | 6.55E-132 | 2.78E-130 | Down |
| RNF2 | 484.691449 | 232.6918973 | 736.6910006 | 3.162459582 | 1.661047042 | 1.15E-33 | 7.60E-33 | Up |
| RNF208 | 60.85905722 | 85.37484439 | 36.34327005 | 0.425574773 | -1.23251546 | 0.000226168 | 0.000415685 | Down |
| RNF223 | 3.89488068 | 0 | 7.789761359 | 38.23161965 | 5.256694415 | 0.003552673 | 0.005788692 | Up |
| RNF227 | 287.7490466 | 430.3824354 | 145.1156578 | 0.337363079 | -1.567626 | 2.37E-21 | 1.03E-20 | Down |
| RNF25 | 2121.836609 | 864.802403 | 3378.870816 | 3.907792106 | 1.966353718 | 1.78E-153 | 9.64E-152 | Up |
| RNF40 | 12385.91617 | 7300.603323 | 17471.22902 | 2.393146495 | 1.258908713 | 2.52E-104 | 7.56E-103 | Up |
| RNF6 | 2588.639665 | 1510.238076 | 3667.041255 | 2.427748178 | 1.279618784 | 7.96E-37 | 5.80E-36 | Up |
| RNFT2 | 247.6436132 | 346.746811 | 148.5404153 | 0.428444033 | -1.222821336 | 9.74E-10 | 2.54E-09 | Down |
| ROGDI | 759.4595635 | 1169.941533 | 348.9775943 | 0.298307742 | -1.745126676 | 1.21E-60 | 1.60E-59 | Down |
| ROM1 | 80.93200066 | 32.48921227 | 129.3747891 | 3.983411445 | 1.994004503 | 4.98E-11 | 1.39E-10 | Up |
| ROR1 | 190.3179023 | 38.19365434 | 342.4421502 | 8.99094486 | 3.168472737 | 4.66E-39 | 3.62E-38 | Up |
| ROR2 | 171.5769411 | 271.5962959 | 71.55758637 | 0.26335079 | -1.924942309 | 3.64E-20 | 1.52E-19 | Down |
| RP1 | 2.968962232 | 0 | 5.937924464 | 29.16623532 | 4.866227275 | 0.008766295 | 0.013598443 | Up |
| RP1L1 | 457.8070178 | 860.8426056 | 54.77143005 | 0.063669132 | -3.973262085 | 4.02E-129 | 1.65E-127 | Down |
| RP2 | 783.1164221 | 358.3233219 | 1207.909522 | 3.368625715 | 1.752160141 | 3.08E-34 | 2.07E-33 | Up |
| RP9 | 1339.578025 | 570.1966468 | 2108.959404 | 3.696183938 | 1.886036553 | 8.62E-105 | 2.60E-103 | Up |
| RPAP1 | 2518.82127 | 1279.589908 | 3758.052632 | 2.936857641 | 1.554273335 | 5.35E-109 | 1.72E-107 | Up |
| RPF1 | 2787.885885 | 1810.570783 | 3765.200987 | 2.079651727 | 1.056341945 | 3.39E-58 | 4.26E-57 | Up |
| RPF2 | 984.9387977 | 627.0479676 | 1342.829628 | 2.141768541 | 1.098802578 | 6.87E-24 | 3.32E-23 | Up |
[truncated: 111,398 more chars]
