# Supplementary figures and images for: Transcription factor 12‐mediated self‐feedback regulatory mechanism is required in DUX4 fusion leukaemia
Source: Clin Transl Med. 2023 Dec 19;13(12):e1514. doi: 10.1002/ctm2.1514 (PMC10731121; doi:10.1002/ctm2.1514)

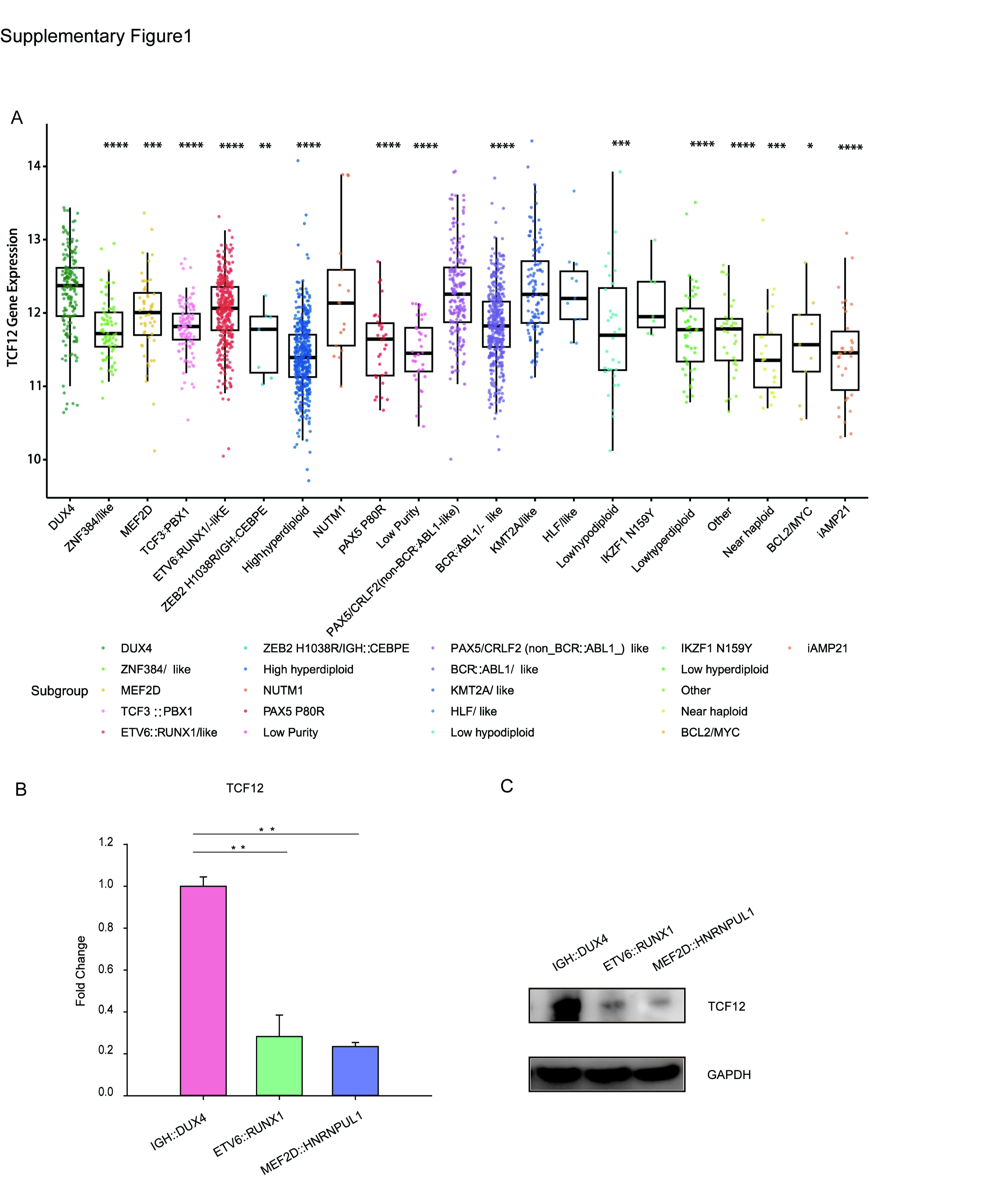

Supplement: Supplementary file 2 — Supporting Information [file CTM2-13-e1514-s001.tif]

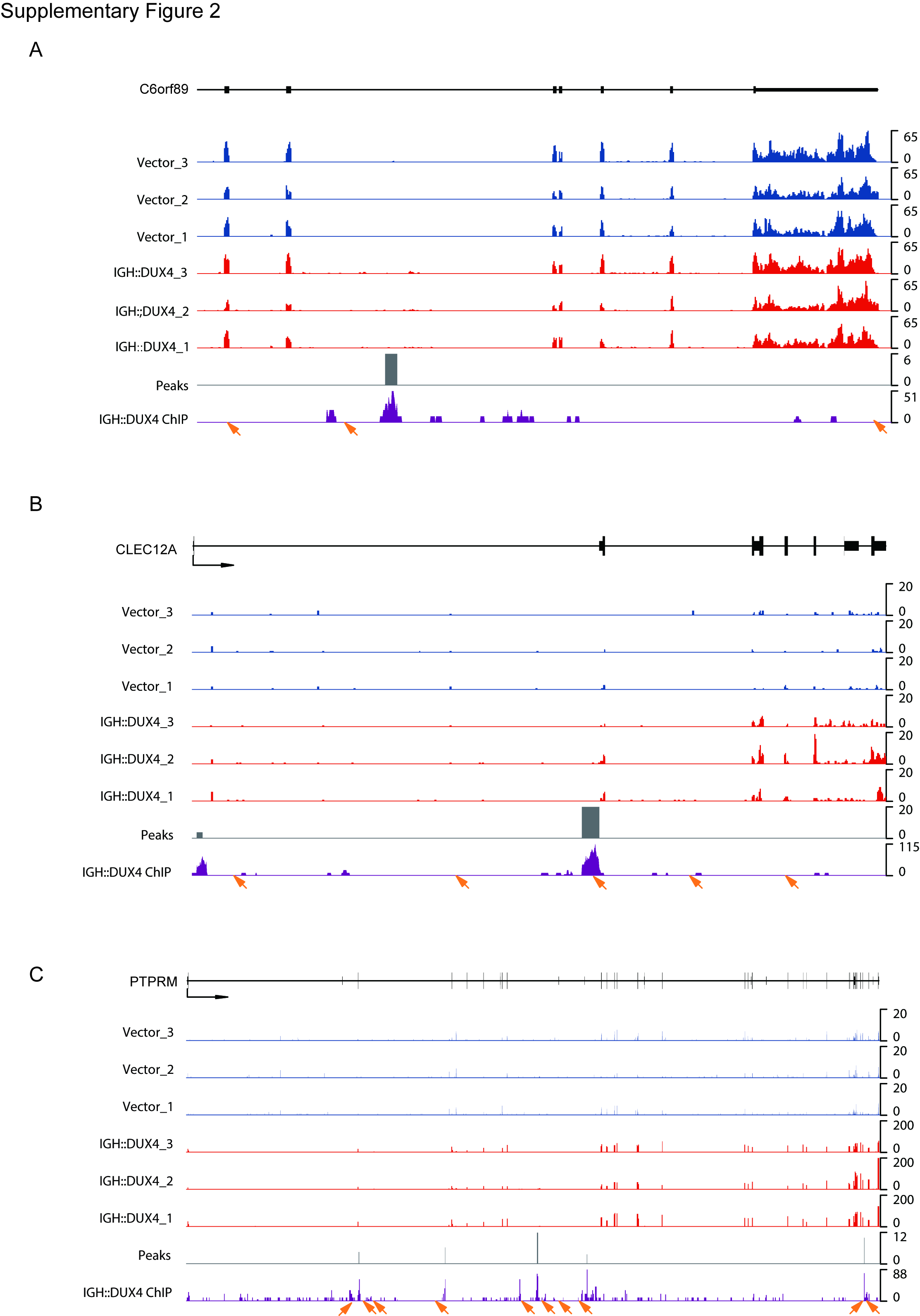

Supplement: Supplementary file 3 — Supporting Information [file CTM2-13-e1514-s008.tif]

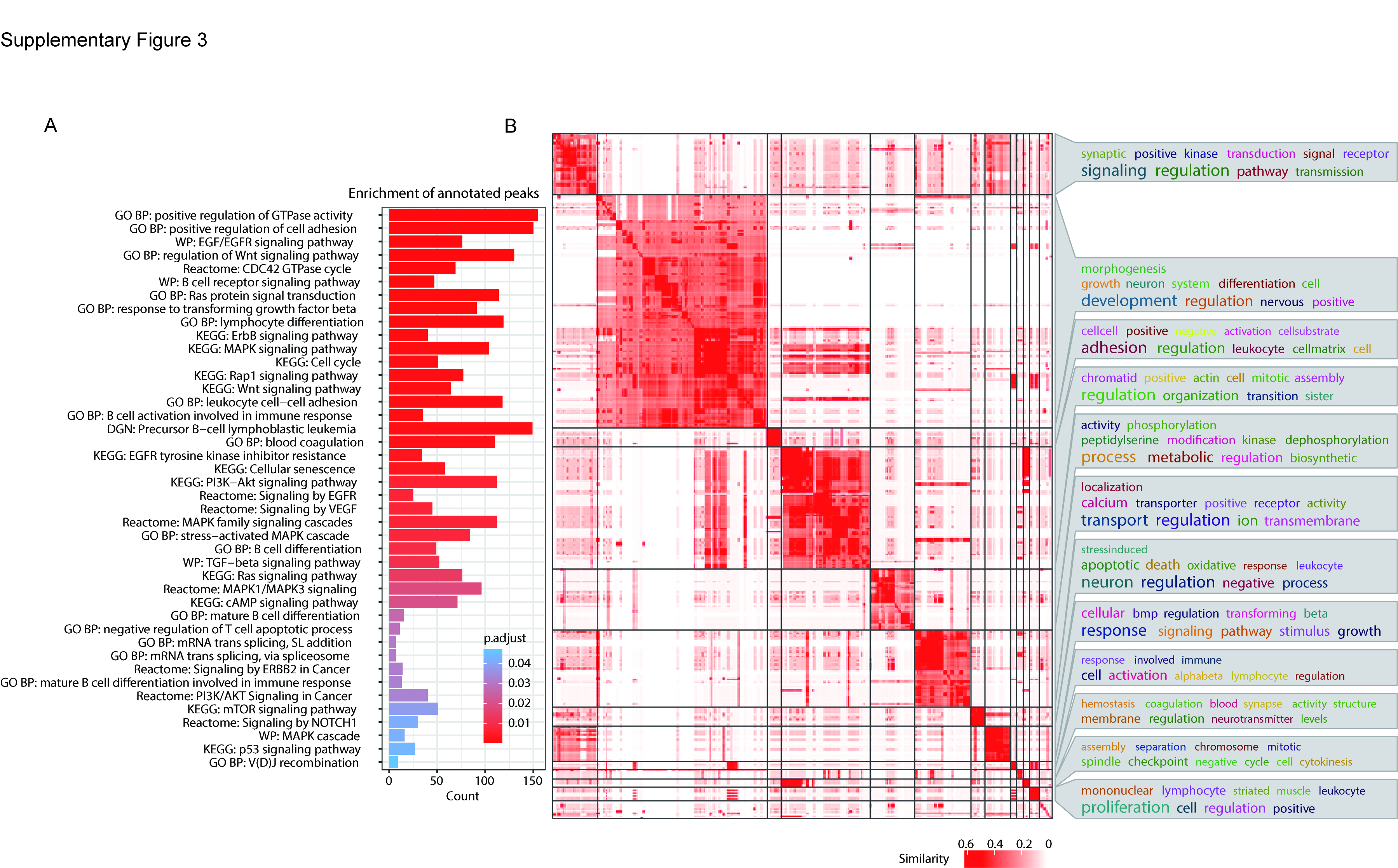

Supplement: Supplementary file 4 — Supporting Information [file CTM2-13-e1514-s006.tif]

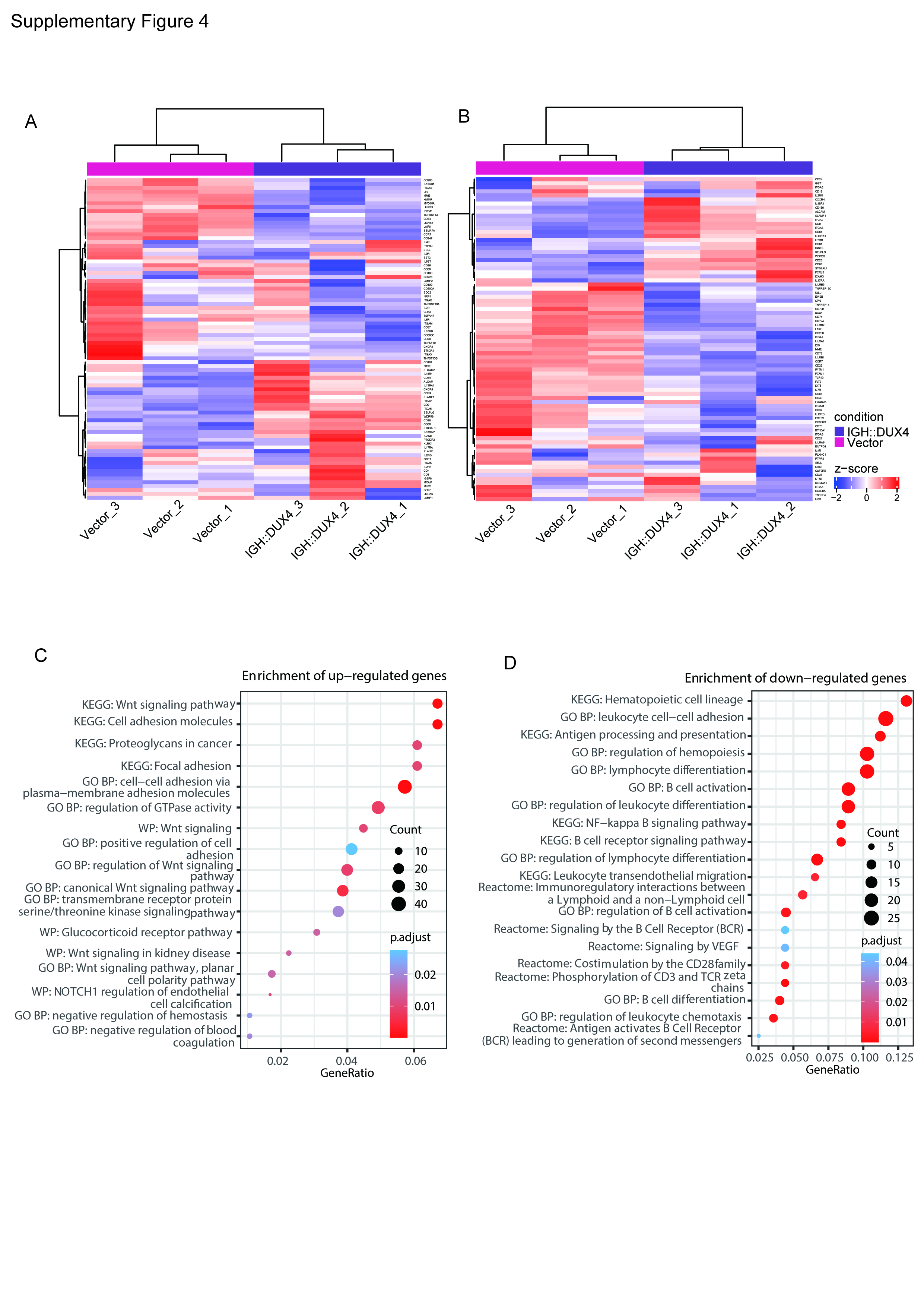

Supplement: Supplementary file 5 — Supporting Information [file CTM2-13-e1514-s010.tif]

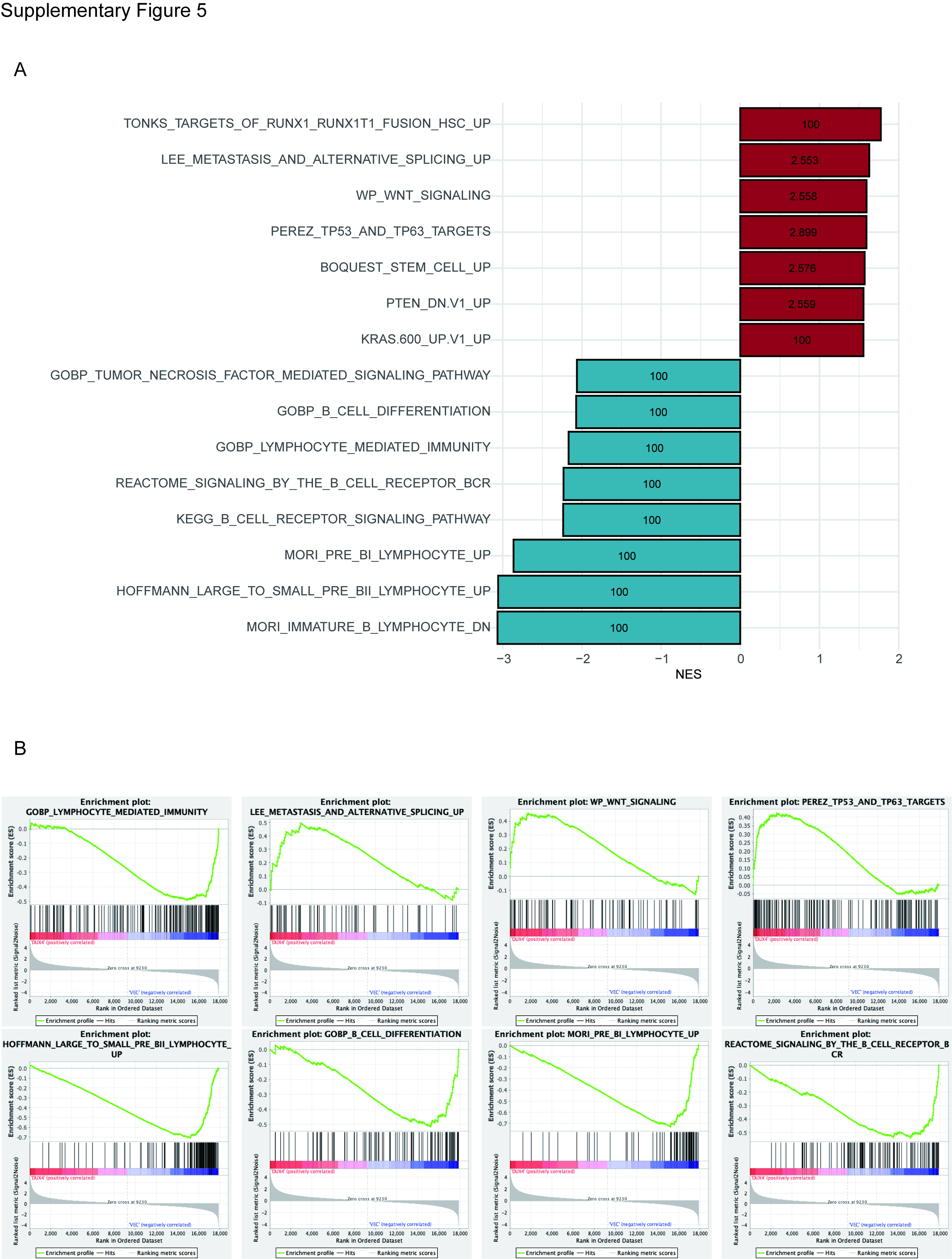

Supplement: Supplementary file 6 — Supporting Information [file CTM2-13-e1514-s007.tif]

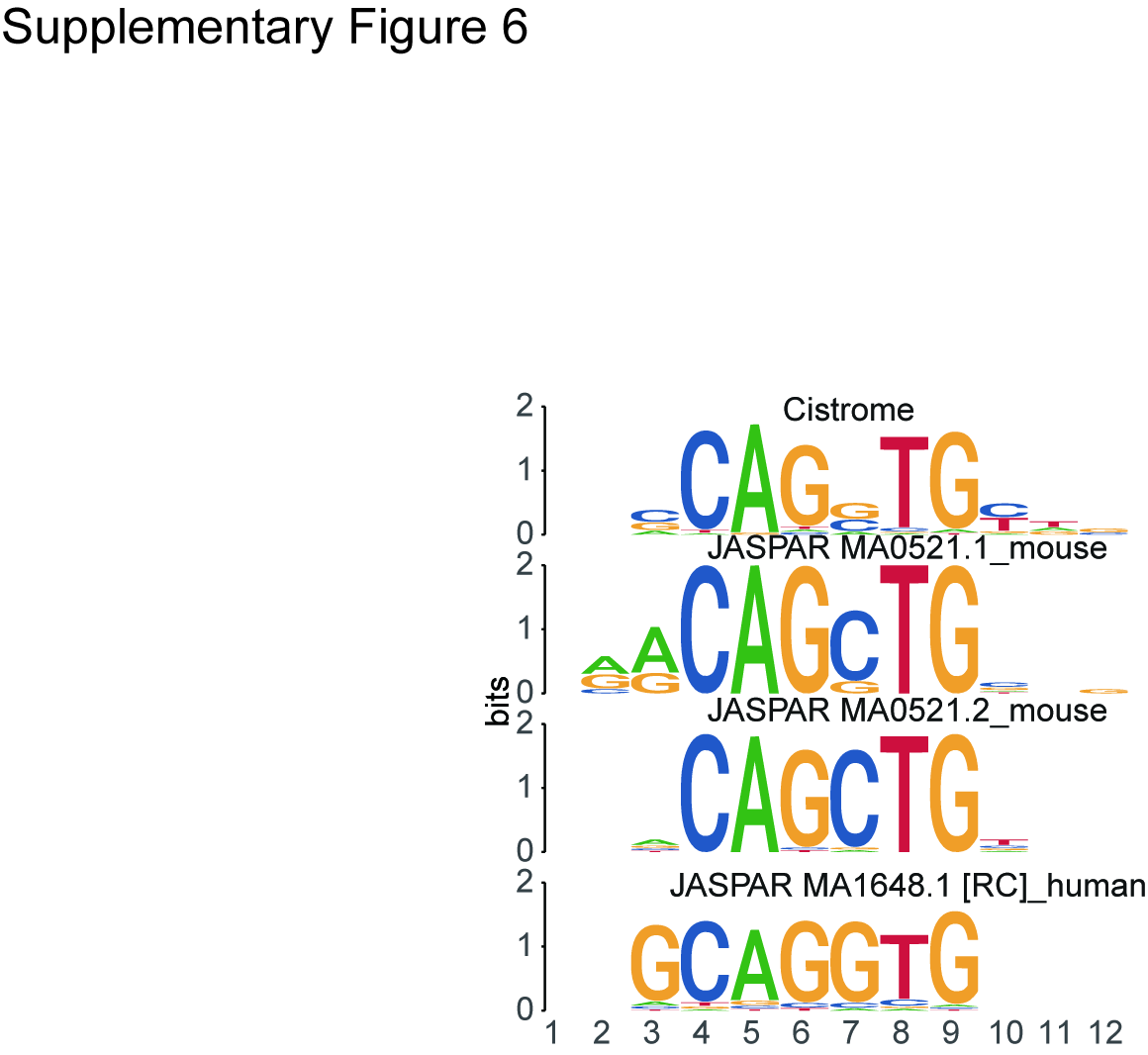

Supplement: Supplementary file 7 — Supporting Information [file CTM2-13-e1514-s002.tif]

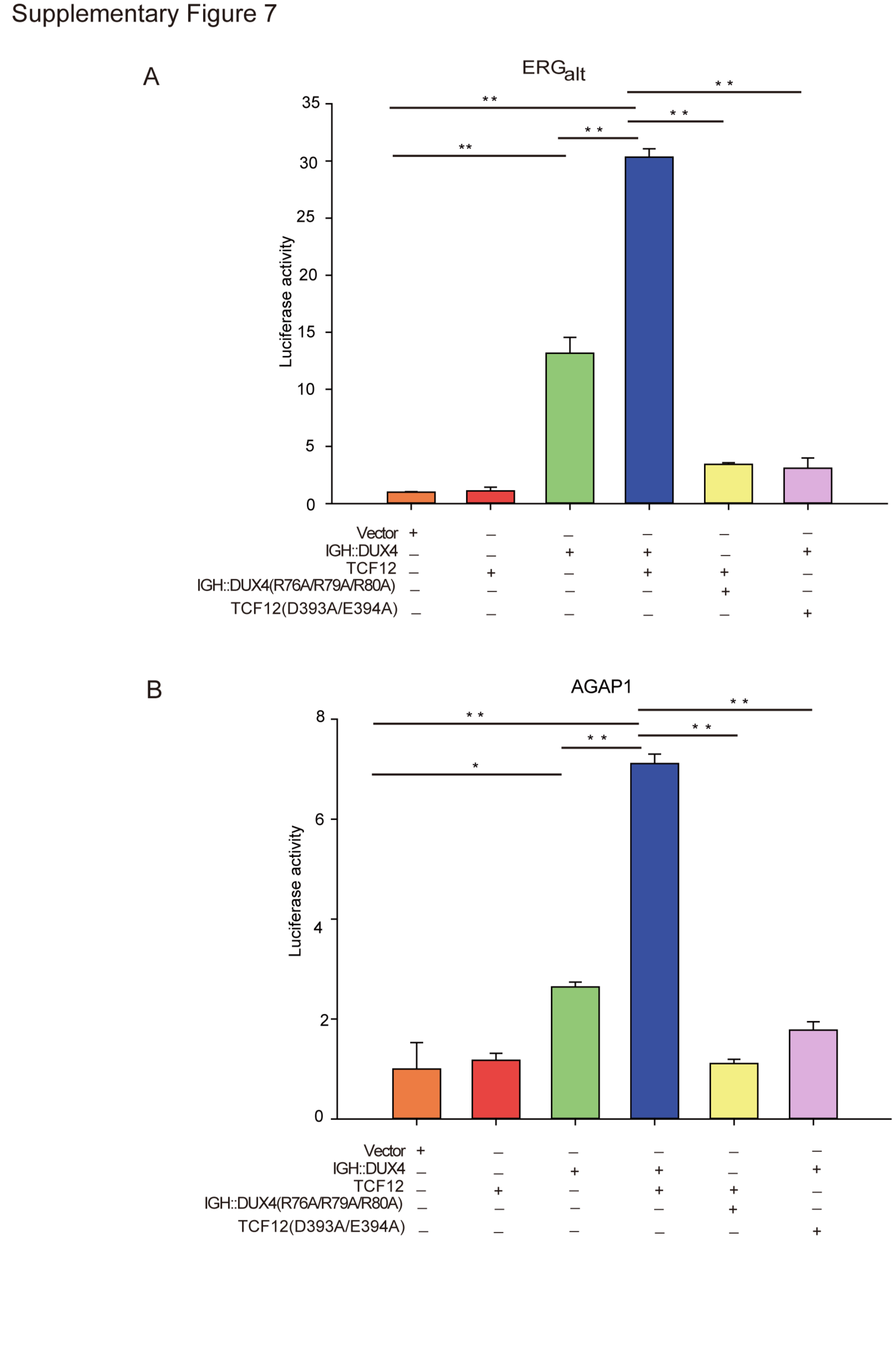

Supplement: Supplementary file 8 — Supporting Information [file CTM2-13-e1514-s004.tif]

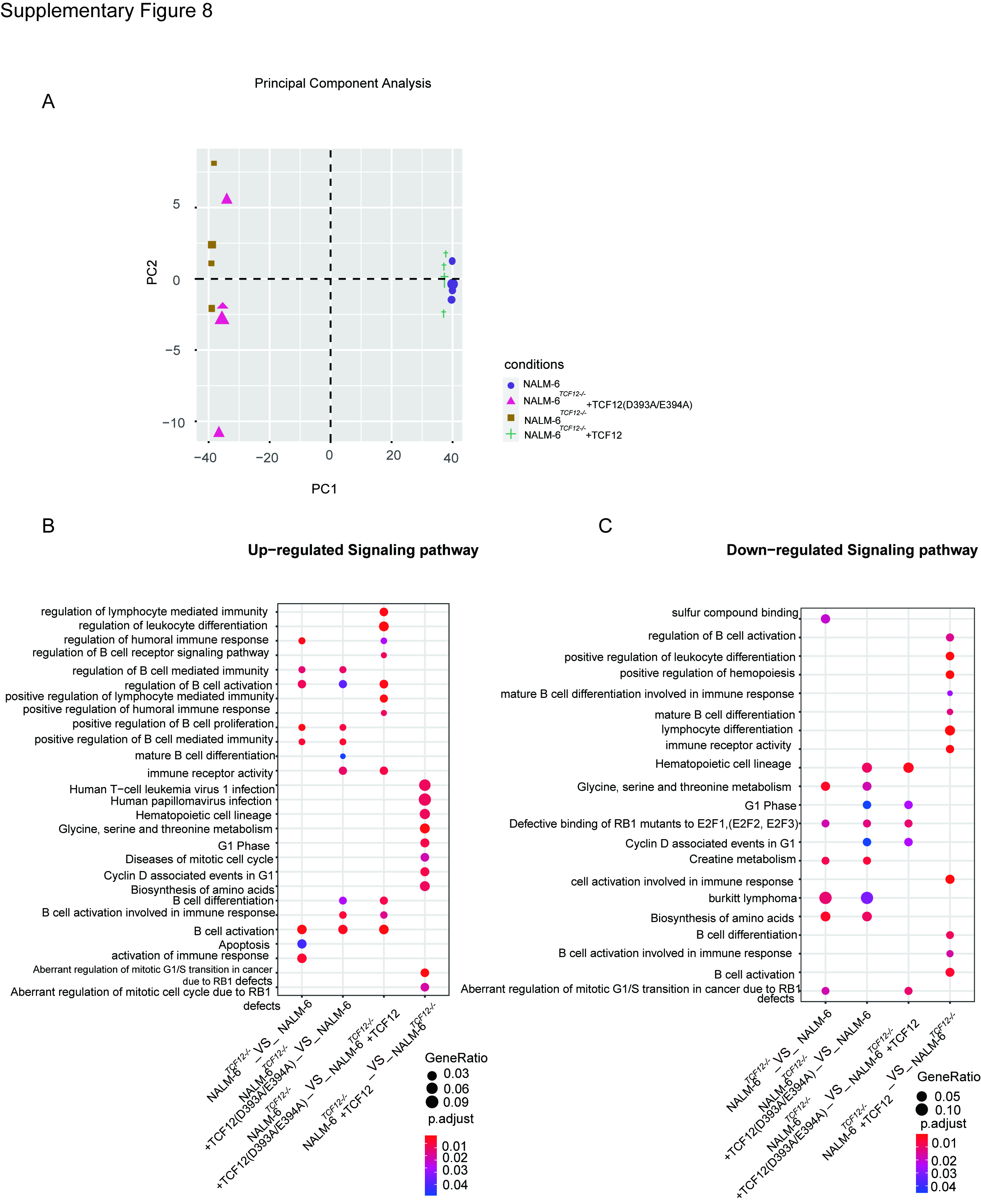

Supplement: Supplementary file 9 — Supporting Information [file CTM2-13-e1514-s003.tif]

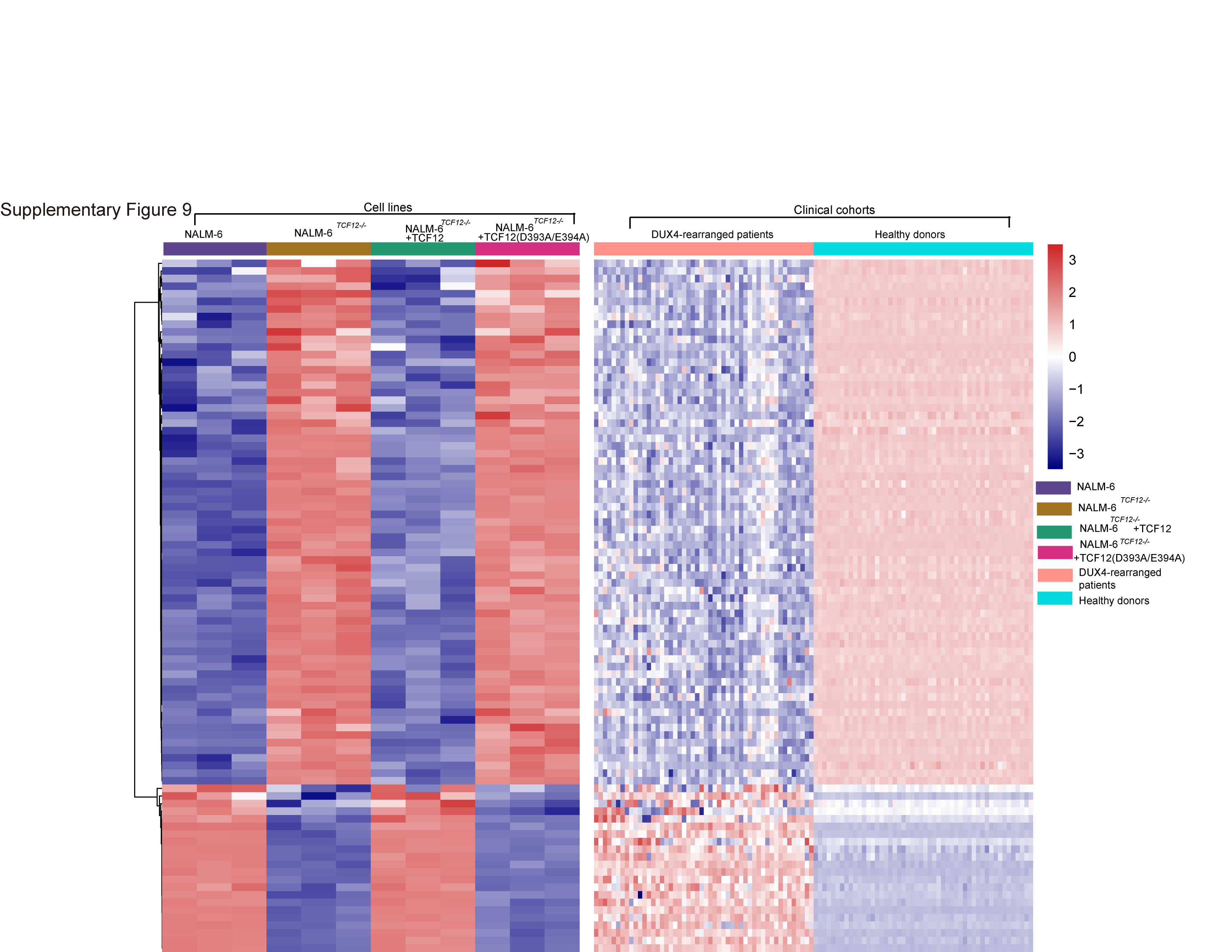

Supplement: Supplementary file 10 — Supporting Information [file CTM2-13-e1514-s009.tif]
